# Exploring translator’s style in children’s literature: A case study of Nicky Harman’s English translations of Huang Beijia’s two works

**DOI:** 10.1371/journal.pone.0350245

**Published:** 2026-06-02

**Authors:** He He, Hongtao Wang

**Affiliations:** School of English and International Studies, Beijing Foreign Studies University, Beijing, China; Bahir Dar University, ETHIOPIA

## Abstract

Huang Beijia’s contemporary Chinese novels *Wo Yao Zuo Hao Hai Zi* (a contemporary realistic children’s novel, 1996) and *Ye Feng Fei Wu* (a historical novel for children, 2018), translated into English by Nicky Harman as *I Want to Be Good* (2020) and *Flight of the Bumblebee* (2023), offer valuable insights into translator’s style in rendering Chinese children’s literature for English readers. Building on Baker’s (2000) translator’s style in corpus-based translation studies (CTS) that manifests translator’s recurrent patterns, this study investigates Harman’s stylistic choices at the lexical, syntactic, and textual level to reveal how Chinese specific elements are represented for target readers. A self-built bilingual corpus (parallel and comparable; 398,531 words total) enables a mixed-methods approach, integrating quantitative metrics (such as keyword analysis, readability index by Qt Readability, etc.) with qualitative close reading. By analyzing typical translation examples and the para-texts of Harman’s translated works, this study aims to explore the reasons behind Harman’s translation style and the relationship between the translator’s views on translation and translation choices. The results reveal the existence of a distinctive translator’s style and show that vividness, purity and joy reflected in the original children’s literature as the ST style has been reproduced in Harman’s translation. A preference for contracted forms (’s,’ve, ’d, etc.) and more strategic and habitual use of certain collocations (such as the employment of “keep an eye on…”, “as far as the eye could see”, etc.), tentative efforts in balancing domestication and foreignization in the translation of Chinese four-character idioms, the multimodal interaction employed by Harman as her special consideration for the young readership, etc. have all contributed to Harman’s translator’s style in translating children’s literature. These findings will contribute to the study of translator’s style and deepen the research of the English translation and international dissemination of Chinese children’s literature under the methodology of corpus-based research.

## Introduction

Existing research on the translation of children’s literature has diversified in terms of its focus, covering works like the world-renowned *Andersen’s Fairy Tales*, *Alice’s Wonderland*, and J.K. Rowling’s *Harry Potter*. These researches have focused on the micro level, such as the translation of proper nouns in *Harry Potter* [1], function and methods in the translation of names [2,3] and respect for the original culture [4]. Macro-level research has viewed the translation of children’s literature as a whole, such as reproducing style in the target text [5], translation strategies, including functional equivalence, equivalent reproduction and effective compensation [6], the translation and dissemination of English children’s literature in the Soviet Union [7], and the influence of ideology on translation of children’s literature [8,9].

Scholarship on children’s literature translation has consistently shown that translators must negotiate readability, cultural specificity, and the expectations of both child readers and adult mediators. Recent corpus-based research has further demonstrated that such stylistic patterning can be identified through recurrent lexical, grammatical, and discoursal choices, while corpus stylistics has also proved increasingly valuable for the study of translated children’s literature. Translator’s style is defined as the fingerprints of a translator or a group of translators in their preferences for certain words, phrases, and syntactic structures, which their regulated behaviors demonstrate. Despite this growing body of research, translator’s style in the English translation of contemporary Chinese children’s literature remains underexplored.

Existing studies on children’s literature translation have paid considerable attention to adaptation, cultural mediation, and readership, yet comparatively less attention has been given to how individual translators construct distinct voices when translating for young readers in the English-speaking countries. Translator’s style is especially worth examining in the case of Huang Beijia with her two children’s works translated into English by Nicky Harman. In *I Want to Be Good*, Harman’s translator’s style is most clearly manifest in the way she constructs and sustains the protagonist Ling’s narrative voice for children readers in the English-speaking countries, a voice that is consistently colloquial, humorous, and emotionally transparent. In *Flight of the Bumblebee*, Nicky Harman’s translator’s style is marked by a consistent re-creation of Orange’s first-person, oral narrative voice in idiomatic contemporary English.

Drawing on a corpus-based approach to translator’s style, this study examines how Harman handles colloquiality, culture-specific expressions, and readability for target children, and how these choices shape the English representation of Huang Beijia’s two children’s works. By focusing on the English translation of contemporary Chinese children’s literature, it helps broaden the empirical base of corpus-based translation studies of translator’s style, which has so far been more heavily concentrated on adult literary texts. This study explores the stylistic features of Harman’s translations of contemporary Chinese children’s literature, examining (i) how her recurrent lexical, syntactic, and textual-level choices reveal her translator’s style and (ii) factors that contribute to her translator’s style.

## Literature review

### Studies on translation of children’s literature

Research on the translation of children’s literature has grown substantially since the 1980s, shifting from prescriptive fidelity to descriptive analysis of cultural mediation, ideological manipulation, and focus on the reader. The prevailing literature primarily focuses on established Western canons for analyzing stylistic shifts in children’s literature translation, such as *Alice’s Adventures in Wonderland* [10], *Pippi Longstocking* [11], *The Wonderful Wizard of Oz* [12], and multimodal formats, such as picturebooks [13].

These researches focus on challenges like cultural references (such as food, folklore, proper names), dual readership (both children and adult), illustrations-text interplay, humor or nonsense, and ideological adaptations. Theoretical frameworks in this field often draw on Even-Zohar’s polysystem theory, translation norms [14], and Venuti’s concepts of domestication and foreignization [15,16], dialogic approach and the “child image” [10], and Lefevere’s work on rewriting and manipulation [1]. Findings reveal systematic domestication for readability and cultural acceptability, including explicitation of foreign elements [17], and prefaces as metatexts revealing translators’ child images [18].

Other perspectives in translation studies of children’s literature include translation history and translation agents, such as the translation and reception history of *Little Women* in Sweden, Denmark, and Norway [19], the role of Protestant missionaries in the evolution of Chinese children’s literature between the late 19th and early 20th centuries [20], and the role of children translators [21]. These studies have revealed the role of translators as invisible storytellers in children’s literature [22] and the relations between translated children’s literature and historical and cultural contexts [23].

Despite substantial advances in studies on the translation of children’s literature, several gaps remain. These studies predominantly examine European language pairs, including English, German, Dutch, Swedish, Finnish, and Spanish, while non-Western contexts remain comparatively underrepresented, with limited sustained attention to Chinese children’s literature in English. Despite the burgeoning interest in children’s literature translation, the individual style of the translator remains an underexplored dimension within the field, often overshadowed by a predominant focus on pedagogical functions and source-text fidelity.

For contemporary Chinese children’s literature, there is some research on the successful English translation of Cao Wenxuan’s *Qingtong Kuihua* (*Bronze and Sunflower*, translated by Helen Wang), such as the genre variation from Bildungsroman to historical fiction [24], narrative space [25], translation and dissemination modes [26] and gender representation [27]. However, research on the translation of Chinese children’s literature in the English-speaking world still needs to be further explored.

Current research on Harman’s translation of children’s literature is relatively scarce, leaving ample room for studying Harman’s translation from more practical approaches. Among the existing research, the examination by Zhong [28] of the translation of humor in Huang Beijia’s *I Want to Be Good* under the General Theory of Verbal Humor (GTVH) and the Variation Theory of Comparative Literature is the most representative. Zhong [28] finds that the use of Chinese humor in colloquial usage has been achieved through Harman’s strategies of both domestication and foreignization, and Harman has preserved the humor through rhetorical devices, metaphors, and, in particular, similes.

### Studies on translator’s style and its reasons

Translator’s style has become an important topic in corpus-based translation studies as it offers empirical and statistical evidence for identifying and interpreting recurring patterns of translator’s choices. In literary translation, style of a translation is therefore often regarded as the outcome of interaction between source-text stylistic traits and the translator’s own preferences, habits, and interpretive decisions [29–31]. From this perspective, translator’s style is conceived as a regular pattern of linguistic behavior that distinguishes one translator’s work from another, which is why Baker [32] famously described it as the translator’s “thumbprint”.

There are basically two types of research on translator’s style: ST-oriented (Source-Text) and TT-oriented (Target-Text). ST-oriented studies treat the style of the target text largely as a response to the stylistic properties of the source text and examines how particular source-text features are carried over, transformed, or weakened in translation [31]. Studies in this tradition have investigated such issues as point of view, speech representation, repetition, cohesion, and narratorial patterning, as illustrated in works represented by [33–44]. Although this approach has generated valuable insights into the relation between source style and target style, it tends to foreground textual response to the original and may leave insufficient room for explaining the translator’s independent responsibility for the stylistic profile of the translation [31,45].

TT-oriented approach aims to identify stylistic regularities that can be attributed more directly to the translator. To make up for the deficiencies in Baker [32]’s model, parallel model in which two or more translations of the same source text are compared with one another and then related back to the original [46–49]. This design offers a stronger basis for distinguishing translators’ stylistic choices because the source-text variable is relatively controlled, but its application is restricted by the fact that multiple translations of the same literary text into the same language are comparatively uncommon.

In corpus-based studies of translator’s style, scholars have investigated a wide range of features, including reporting verbs, modal particles, verbs of speech and cognition, conjunctions, clusters, and other recurring lexical or functional items that shape characterization, narrative stance, and textual cohesion [34,41,42,46,50].

As Leech [51] argues, no linguistic feature can be considered stylistically distinctive unless it departs from some norm of comparison. For that reason, more recent studies have increasingly relied on comparable and parallel corpora, using reference corpora to establish norms of the target texts and aligned source and target texts to trace how stylistic features are transferred, modified, or neutralized in translation. Corpus-based translation studies of translator’s style has increasingly moved toward mixed models that combine parallel and comparable corpora in order to distinguish more carefully between source-induced patterning and genuinely translator-specific preferences [45].

As for reasons for translator’s style, there’re three factors that may influence translator’s style, including (1) the translator, (2) the culture of the target language, and (3) cultural differences between the source and target language [49]. Reasons for translator’s style are usually explored through extra-textual variables such as translator background, translation purpose, translator preference, interpretation of the source text, and patronage or publishing context [49,52]. Differences in the reconstruction of themes, lexical chains, and semantic fields, with the exploration of functional translator’s style, can be explained by factors including translator background, motivation, personal preference, interpretation, and patron influence [52].

Berry and Egan’s translation of *The Song of Everlasting Sorrow* is interpreted as more colloquial and explicit partly because the translators sought to reduce ambiguity and improve accessibility for target readers from a different cultural background [45], while Goldblatt’s relatively stable style is linked to his long-term engagement with specific authors, sustained translational practice, and cooperative author-translator relationships [53]. The causes of translator’s style can now be investigated through an integrated explanatory framework in which quantitative corpus findings are systematically connected to cognitive, functional, biographical, and socio-institutional factors.

### Studies on translator’s style in children’s literature

In children’s literature, corpus stylistics is particularly valuable because it allows scholars to trace repeated textual choices and to relate them to broader questions of style, narration, and reader positioning, such as through inspectable evidence from parallel corpora [43]. Recent corpus-based research demonstrates that translators of children’s literature actively negotiate complex constraints, as evidenced by shifts in multimodal verbal-visual relations [54] and extensive ideological adaptations through cultural filtering [55]. Computational stylistics reveals that such textual variations correlate fundamentally with the age of the target readership [56], collectively highlighting the necessity of interpreting quantitative linguistic patterns through the qualitative lenses of style, ideology, and audience design.

Translator’s style in corpus-based translation studies, are only beginning to be applied to children’s literature. Despite the growing use of corpus methods in children’s literature translation studies, research specifically devoted to translator’s style in this field remains limited. Existing studies tend to rely on a relatively narrow set of indicators, most commonly lexical measures such as tokens, types, TTR, and STTR, sometimes supplemented by sentence length or selected rhetorical features. Such designs are useful, but they may not be sufficient to capture translator’s style as a more complex and multi-level textual phenomenon. Many studies continue to follow a traditional comparison model in which style is identified by contrasting two or more translators, rather than by examining how one translator’s stylistic profile operates across a carefully controlled corpus of texts, such as one translator’s translation of children’s literature.

With the inclusion of a diversified parameters at the lexical, syntactical and textual levels, this study adopts the mixed model for the exploration of Harman’s translator’s style by first comparing Harman’s translation of children’s literature with the reference corpora and then referring to the two source texts for a cross-comparison of translator’s response to the original texts.

## Theoretical framework

The paper titled “Towards a Methodology for Investigating the Style of a Literary Translator” by Baker [32] has been a milestone in establishing the study of translator’s style under the corpus-based methodologies, in which translator’s style has been identified by comparing two translators’ English translations in terms of their preferred choices of certain words, sentence structures, connective words, and punctuation. Based on interviews with the two translators and empirical data obtained from the corpus, Baker [32] points out that Peter Clark, whose translations are characterized by a lower type/token ratio and a shorter average sentence length, is probably influenced by the source language. He also caters to the language abilities of the receptors. Due to his personal experience working for the British Consulate in the Middle East, which would influence his cognitive process, Peter Bush tends to move the original Arabic culture towards the reader, creating an easy text for comprehension. In contrast, Peter Clark tends to move the readers towards the original Spanish context, thus presenting texts with challenges for the target readers.

Following Baker [32]’s line, Saldanha [57] refines Baker’s original conception of translator’s style through insights from literary and forensic stylistics and treats style as both habitual linguistic choices and motivated rhetorical strategies, linking micro‑level textual tendencies to broader sociocultural and ideological factors. Saldanha’s [31,58] research on the differences in the loan words and italics and connective words after verbs like say/tell between the two translators, Peter Bush and Margaret Jull Costa, and Olohan’s [59,60] research on the differences in the use of contracted forms between the two translators, Peter Bush and Dorothy Blair, has advanced research on translator’s style, introducing more feasible parameters into the study of translator’s style. Olohan [59,60] relates translators’ use of contractions and optional syntactic elements to broader tendencies such as explicitation and normalization in large electronic corpora. In addition, Saldanha [58,61], for example, examine the systematic use and discourse functions of foreign words and emphatic italics across an individual translator’s works, showing that stable stylistic traits can be identified even when source texts and paratextual norms vary. More recent works in this field have further consolidated this line of inquiry by combining quantitative patterning (e.g., frequency, keyword and cluster analyses) with qualitative interpretation, and by situating translators’ characteristics either within monolingual comparable corpora that are composed of translated versus non-translated texts, or within parallel corpora that control for source-text effects [62–64].

From this as the starting point, besides the comparable model, a parallel corpus has been built by scholars to identify the influence of the source text on the representation of textual and linguistic features of the translated texts.

Some studies focus on one parameter for identifying translator’s style, such as nominalization in Hou [65]’s research on the two English versions of the Chinese classical novel *Hong Lou Meng*, which are represented by Joly’s formal style and Yang and Yang’s concise style.

A recent development of translator’s style can be found in the studies of translation history. Based on the AHRC-founded Genealogies of Knowledge (GOK) project [66], a list of keywords related to participants involved in governing the city-states of “Thucydides” Greece has been generated in the three translations of Thucydides’ History of the Peloponnesian War to identify the chosen keywords and to be compared in Joweet’s and Crawley’s versions. A further exploration of concordance of “leaders” from the three translations has been used to verify that Jowett’s personal politics featured by the ideology of leadership and developed during his career as Master of Balliol College, Oxford have shaped his representation. An investigation of the use of the term “fact(s)” has been employed to reveal Crawley’s (1874) History’s tendency to present a significantly more objective and empirical tone to Thucydides in English [67].

Children’s literature is also a highly constrained genre, as translators must balance the author’s style, the expectations of child readers and gatekeepers (parents, teachers, publishers), and often strong ideological or didactic norms. These constraints mean that small but systematic shifts in, for example, repetition, sociolect, or evaluative stance can substantially reshape how a child reader experiences the story. Corpus-based translation studies of children’s literature have in fact followed Baker’s line, by using keyword, cluster and collocation analysis to uncover distinctive translator behaviour. Malmkjær [68] demonstrates how corpus stylistics can reveal translational choices in *Harry Potte*r and *Winnie-the-Pooh* that both respond to child-directed constraints and instantiate specific translational styles. Similarly, Borodo [69] applies corpus-based techniques to Adam Czasak’s translation of Korczak’s *Little King Matty*, showing how the translator’s style interacts with sociolectal representation in translated children’s fiction.

Work on translator’s style since Baker’s corpus‑based methodology has shown that translators leave recurrent, measurable linguistic traces across texts (e.g., wordings, phraseology, and syntactic routines), and that such traces can be characterized through keywords, clusters and concordances. In children’s literature, classic accounts of genre constraints and gatekeeping [10,70] and early quantitative studies of readability or ideology [71] explain why relatively small but systematic choices, such as repetition, evaluative stance, and colloquiality, can materially affect reception, making this a promising domain for translator’s style. More recent applications confirm the potential of the methodology applied in children’s literature. Corpus stylistics can be applied to identify translators’ recurring stylistic habits and preferred patterns in canonical children’s texts [68], and sociolectal representation through enhancing emotional expression, adopting non-formal grammatical structures, and integrating social dialects into the target text, has demonstrated stable and specific translator’s style [69].

Yet there are three gaps that remain in the corpus-based translation studies of children’s literature. First, the existing body of evidence remains disproportionately focused on European language pairs and authors, with relatively scant corpus-based research devoted to Chinese-to-English translations of children’s literature. Second, many studies examine single titles or single phenomena, such as Zhong [28]’s study of humour in Huang Beijia’s *I Want To Be Good*, which limits our ability to separate authorial voice from translator‑specific style. Third, despite the pivotal role of paratextual devices (e.g., series branding and blurbs) in children’s publishing, examinations of these features are frequently intuitive, failing to substantiate claims through close reading of the translated text. Addressing these gaps requires a design that (i) controls for author effects across multiple works by the same author, (ii) combines corpus stylistics with paratextual analysis, and (iii) extends the geographical scope of the field.

We therefore propose to examine Nicky Harman’s translator’s style in Chinese children’s literature using two English translations of works by the same author, Huang Beijia, namely *I Want To Be Good* (New Classic Press, 2021) and *Flight of the Bumblebee* (Balestier Press, 2022). This choice controls for authorial voice while varying title, publisher, and target positioning, allowing us to test whether Harman exhibits recurrent stylistic routines across books. In this study, we put forward the hypothesis that the translator maintains a stable translator’s style across the translation of two works of children’s literature, and we verify this hypothesis through empirical analysis.

With corpus-based translation studies as the theoretical framework, this study intends to explore if the two translated works of the same author by the same translator, show the existence of translator’s style and how translator’s style is reflected at the lexical, syntactical and textual level as well as in the choices of cultural-specific Chinese items when rendered into English. In addition, the reasons for translator’s style will be further demonstrated from the perspective of translator’s view of translation and translator’s ethics.

This study aims to answer the following questions:

1. Do Harman’s translated versions of the two children’s literature demonstrate the existence of translator’s style at the lexical, syntactic and textual level? Is her translator’s style stable?
2. What factors may contribute to the formation of translator’s style?

## Research methodology

Corpus Linguistics and Descriptive Translation Studies (DTS) are the two main sources of influence and inspiration to Corpus-based Translation Studies [72]. Corpus Linguistics typically follows a staged research design that moves from question formation to corpus construction, quantitative pattern detection, and qualitative interpretation. Researchers define stylistically motivated questions, compile and annotate an appropriate corpus when needed, run statistical and distributional analyses (e.g., keywords, frequencies, collocations, concordances) to identify salient patterns, and then return to close reading to interpret how these patterns function aesthetically and pragmatically in context [73].

There are two steps for the descriptive and explanatory methodology grounded in Descriptive Translation Studies and Corpus Linguistics. First, the researcher constructs an adequately sized corpus of the translator’s output (ideally across multiple works) and identifies “preferred or recurring patterns of linguistic behavior” [32] by comparing the translator’s distributional choices against relevant benchmarks (e.g., other translators and/or a reference corpus), thereby producing empirically testable descriptive generalizations about the translator’s “patterns of choice” rather than isolated interventions [32]. Second, these quantified regularities are interpreted in relation to translation norms and extra-textual conditioning, i.e., the translator’s position, project, and socio-cultural horizon, so that stylistic patterning is not treated as idiosyncratic but as socially situated behavior that can be reconstructed from textual evidence and then explained with reference to contextual constraints.

Translator’s style is a representative and hot discussed topic in CTS, as its emerging trend can be shown in book volumes and article publication in recent translation studies [49,72,74–77]. Building on Baker [32]’s definition of translator’s style as a translator’s “characteristic use of language” and “preferred or recurring patterns of linguistic behaviour”, a substantial body of corpus-driven work now investigates how such patterns can be revealed by comparing an individual’s translations with reference corpora of non-translated texts or with other translators’ versions, using techniques such as keywords, lexical bundles, collocations, and multi-dimensional stylometric analysis [32,58,78].

This study is based on a bilingual parallel corpus, with the original texts available for reference, thus filtering out disturbance that results from influences from the unknown original texts. This differs from Baker [32]’s translator’s style in our advancement of the inclusion of both the source text and the target text and the consideration of parameters that are typical of Chinese children’s literature.

Translator’s style can be reflected through basic statistics generated by WordSmith Tools and Sketch Engine and AntConc 3.5.4, including Standard Type/Token Ratio (STTR), mean word length (MWL) and lexical density for lexical richness and difficulty, keywords analysis for translation strategy employed by the translator in the tentative and skillful choice of words, phrases targeted at the young readership at the lexical level. In addition, average sentence length (ASL) and sentence translation ratio offer statistic support for translator’s style at the textual level. Moreover, paragraph translation ratio and readability stand at the textual level. The parallel corpus generated by tmxmall.com offer the efficiency to locate the corresponding English translation of culture-loaded terms, slangs and idioms and compare the methods employed by the translator.

In this study, we’ve modified parameters based on Baker [32] and identified 11 parameters at the lexical (7 parameters), syntactic (2 parameters) and textual (2 parameters) level in revealing translator’s style as shown in Table 1.

**Table 1:** Parameters for translator’s style at different levels.

Level	Parameters for translator’s style	Tools
Lexical level	(1) STTR (Standard Type/Token Ratio) (2) MWL (Mean Word Length) (3) Lexical Density (4) Keywords analysis (translation of character names, frequent use of contracted forms, collocation of high-frequency words or keywords) (5) translation of four-character Chinese idioms (6) onomatopoeia (7) flexible treatment of culturally-loaded terms, slang and dialects	(1) WordSmith 8.0 (Wordlist Function) (2) WordSmith 8.0 (Wordlist Function) (3) AntConc 3.5.4 (Search Term: *_AJ* *_N* *_AV* *_VV*, for content words) (4) WordSmith 8.0 (Keywords Function); Sketch Engine (word sketch function) CUC_ParaConc V03 (for parallel corpus) (5)(6)(7) AntConc 3.5.4 (Regular expression) (5) \b[^\x00-\xff]{4}\b (6)\S + /o (7) \S + /(nz|[naval]l) CUC_ParaConc V03 (for parallel corpus)
Syntactic level	(1) ASL (Average Sentence Length) (2) Sentence Translation Ratio	(1) WordSmith 8.0 (2) Readability Statistics tool within Microsoft Word
Textual level	(1) Paragraph Translation Ratio (2) Readability	(1) Word count tool within Microsoft Word (2) Qt Readability

At the lexical level, we’ve expanded parameters common in studies on translator’s style, from STTR (Standard Type/Token Ratio), MWL (Mean Word Length), LD (Lexical Density), Keywords, to include (a) four-character Chinese idioms, (b) onomatopoeia, and (c) culturally-loaded terms, slang and dialects, which are typical and representative in English-Chinese translation. Thus, we aim to explore translator’s style in a relatively holistic way in this study, not limited in the basic statistics generated by corpus tools, such as WordSmith 8.0, AntConc and Sketch Engine, for more convincing evidence of translator’s style.

The examination of (a) four-character Chinese idioms can reveal translation strategies employed by the translator when dealing with expressions specific to Chinese, that are embedded in Chinese culture and history, and representative of Chinese narrative. We have categorized and calculated the proposition of seven translation methods employed by Harman, including (1) literal translation, (2) free translation, (3) simplification, (4) loan translation, (5) omission, (6) shift and (7) literal translation + free translation in both two translated children’s works. A brief summary of the result is also provided in the discussion section, to emphasize on the translator’s balance between domestication and foreignization in translating four-character Chinese idioms to achieve the balance between acceptability and adequacy.

In addition, (b) onomatopoeia reflects the rhythmic patterns in children’s literature, and the reproduction of it actively recreates the sensory environment of the narrative. By echoing similar onomatopoetic forms at key moments of action, emotion, or humour, the translator constructs a recognizable voice that both supports coherence and helps to shape a distinctive translator’s style oriented towards oral performance and read‑aloud enjoyment in the target culture.

(c) Flexible treatment of culturally-loaded terms, slangs and dialects are valuable because translators’ handling of such items (whether they neutralize, adapt, annotate, or preserve them) is strongly constrained by assumptions about child readers’ processing abilities and by the pedagogical or protective norms of the target culture, making this a test for the translator’s positioning as cultural mediator [10].

At the syntactic level, ASL (Average Sentence Length) and Sentence Translation Ratio have been chosen as the parameters. A shorter ASL often suggests a strategy of syntactic simplification, aimed at enhancing the narrative flow for younger readers. Sentence Translation Ratio (the ratio of the number of sentences in the target text to those in the source text) is a crucial metric for analyzing textual expansion or contraction. Long and complex source sentences are broken down into shorter, more digestible units for children [79]. The total sentence count is initially extracted using the Readability Statistics tool within Microsoft Word, followed by a manual verification.

At the textual level, Paragraph Translation Ratio serves as a quantitative indicator of a translator’s macro-structural intervention, revealing translator’s tendency toward either condensation or expansion. A high degree of segmentation often signifies a domesticating strategy aimed at enhancing readability and narrative flow for the target audience. Readability provides a quantifiable measure of how translators adapt the linguistic complexity of the source text to align with the cognitive constraints and reading abilities of the target child audience [10,79].

For the textual features of the translated texts, Qt Readability (downloaded from https://corpus.bfsu.edu.cn/TOOLS.htm) has been employed to calculate the readability of the texts. Flesch-Kincaid Readability Index (FKRI) and Flesch-Kincaid Grade Level have been examined to test the readability of the two translated children’s literature. As FKRI ranges from 0 to 100, the lower the index, the more difficult the text is.

Flesch-Kincaid Grade Level [80] has been a useful way to estimate the complexity of the English text. The Flesch Reading Ease Formula is,


$$\begin{array}{c} \text{Grade Level }(\text{GL})\;=\;\text{0}.\text{39 }\times \;(\text{total words}/\text{total sentences})\;+\text{ 11}.\text{8}\;\times \;(\text{total syllables}/\text{total words})-\text{15}.\text{59} \end{array}$$

The score roughly corresponds to a grade level (K-12) in the United States education system. The score indicates that the text can be understood by students at the corresponding grade level.

Reasons for Harman’s translator’s style have been summarized in both the macro-level and micro-level (as shown in Table 2), including socio-cultural factors, translator’s preferences and translator’s view on translation. Paratextual materials in the case of Harman’s translation have provided rich materials for further explanation of her translator’s style.

**Table 2:** Reasons for Harman’s translator’s style.

Level category	Different factors	Detailed explanation
Macro-level	Socio-cultural factors	Lower position of translated literature from non-Western authors
Micro-level	Translator’s preferences	Translator’s special consideration for the young readership: multimodal interaction
	Translator’s view on translation	(1) dual responsibility, (2) a balance between domestication and foreignization, (3) translator’s voice, (4) translator as an active rewriter

## Data processing and tools in a corpus-based methodology

### Material selection

We choose *I Want to Be Good* and *Flight of the Bumblebee* translated by Nicky Harman as the corpus, together with its original Chinese text as the parallel corpus. This follows the line of study on translator’s style, as Baker [32] defines translator style as “a matter of patterning” that “must attempt to capture the translator’s characteristic use of language, his or her individual profile of linguistic habits, compared to other translators”. Using BNC (British National Corpus) as a reference corpus in the keywords analysis, is methodologically appropriate for a study of Nicky Harman’s translations of Huang Beijia’s two children’s works because it provides a balanced baseline of general British English against which statistically salient, translator-specific regularities can be identified.

The selection of the two translations and their original texts as the corpus data follows the principles of representativeness and comparability. First, the two Chinese children’s works are both translated by Nicky Harman, and published in 2021 by New Classic Press and 2023 by Balestier Press respectively, at the same period with an interval of 2 years, and with publishers both located in London for the same publishing environment. This leaves enough space for the discovery of the fingerprints left by the same translator, Harman, as the genre set for children’s literature. Second, the original Chinese texts are from the same author, Huang Beijia, indicating that the two translations are highly comparable. The two Chinese children’s works were published at different periods, with *Wo Yao Zuo Hao Hai Zi* in 1998 and *Ye Feng Fei Wu* in 2021 in China. As part of the Jiangsu Literature Translation Series, an initiative sponsored by the Phoenix Publishing and Media Group, two children’s works by Huang Beijia have been selected for global distribution. This project, originating from the author’s home province of Jiangsu, aims to introduce influential local writers to an international audience, thereby enhancing the dissemination of China’s cultural discourse on the global stage.

### Tools: WordSmith 8.0, AntConc 3.5.4, Sketch Engine

WordSmith Tools has been the most widely applied and reliable software for corpus analysis with its key functions, such as word list, keywords list and concord, etc., providing statistical support for further analysis of how the translated texts represent or deviate from the original text based on keywords analysis, the collocation of high-frequency word or keywords, etc. Its application in corpus-based translation studies can be traced back to the early establishment and development of corpus-based translation studies as shown in Baker [32,81] to reveal recurrent features of translated language and translator-specific patterns and translator style, followed by Baker & Olohan [82], Winters [34,83] and Saldanha [31,58,61]. Two distinctive research topics have been widely explored under corpus-based translation studies: (i) features of translated language (explicitation, normalization, etc.) and (ii) translator’s style (stable patterns traceable across a translator’s works).

WordSmith Tools 8.0 [84] has been employed for text processing, to generate word list, keywords and concord, etc. After adding a lemma list (BNC_lemmafile5) and a stop list (English_stoplist) (advanced settings--lists--wordlist), we select two text files: *I Want to Be Good.*txt and *Flight of the Bumblebee.*txt in Wordlist interface of WordSmith 8.0 and click “make a word list” button to generate statistics. Then, we obtain the basic information on the vocabulary and sentences of Harman’s two translated children’s works, including STTR, MWL (Mean Word Length) and ASL, etc.

AntConc, introduced by Laurence Anthony in the early 2000s, offers a free, cross-platform alternative that quickly spread into teaching practices and small-scale research, centered on concordance, keywording and collocations. It appears routinely in corpus-based translation studies and readily works as an accessible complement to WordSmith. AntConc 3.4.5 has been used for searching the content word count, four-character Chinese idioms, onomatopoeia, and slangs, etc.

As a web-based corpus query and management system, Sketch Engine provides access to sizeable monolingual and parallel corpora across many languages and allows users to upload and analyse their own specialised corpora, which is crucial for investigating domain-specific translation choices and constructing translator-specific or genre-specific datasets [85]. Sketch Engine has been identified and highlighted as a “powerful suite of corpus tools for cross-linguistic analysis,” underlining its growing role in both research and advanced translator education [86,87]. Sketch Engine has been utilized for the examination of the collocation of specific words.

### Procedure

After determining the English and the original texts and corpus software needed for the research, the corpus is built after cleaning the texts, segmenting the original Chinese words and tagging part of speech of the English and Chinese texts.

Corpus construction. We scanned the English versions by the software Quark scanner, downloaded from the website https://scan.quark.cn/web/ and installed in the mobile phone, and the scanned texts were saved as PDF format. Then the scanned PDF documents were converted into editable doc. format by the tool 4Videosoft PDF Converter Ultimate, running on PCs, with a very good OCR technology, developed by 4Videosoft Studio. After manual checks of these wrongly converted letters and punctuations, such as “I’ll” as “I^ll”, “the” as “tie”, the documents were stored in TXT format for segmentation and POS tagging, after removing Translator’s note, cover, and other paratextual information, with the texts left alone. We downloaded the two original Chinese versions from the Internet and convert them into TXT format.

Alignment and processing. The parallel corpus was built based on the online platform https://www.tmxmall.com/, a website developed by Yizhe Technology company, distinguished by its efficiency in aligning texts according to its own algorithm and complemented by manual checkups to ensure that no blanks exist or unmatched sentences are found. Then, the saved text has been put in the retrieval software CUC_ParaConc V03 (developed by China’s Renmin University) for the retrieval of source and target parallel texts, with both the source and target retrieved vocabulary marked in color red for emphasis and easy recognition.

Word segmentation and corpus tagging. For Chinese, POS tagging has been implemented by NLPIR (a powerful natural language processing software/platform for Chinese with a high accuracy for the part-of-speech tagging, the most representative platform released by Chinese Academy of Sciences (CAS), developed by Dr. Zhang Huaping, http://www.nlpir.org/). For English, treetagger.com has been used for POS tagging. The tagged corpus can be searched in AntConc 3.5.4 for the calculation of content words, used for lexical density.

## Corpus-based analysis of Harman’s two translated children’s literature

Translator’s style in Harman’s translation of the two children’s works by the Chinese author Huang Beijia will be discussed at the lexical, syntactic, and textual levels.

### Translator’s style manifested at the lexical level

Descriptive statistics of Harman’s two English translated children’s literature generated by WordSmith 8.0 can be shown in Table 3.

**Table 3:** Descriptive statistics of Harman’s two English translated versions of children’s literature.

	*I Want to Be Good*	*Flight of the Bumblebee*
Tokens (running words) in text	79388	74265
Types (distinct words)	6324	7063
TTR	7.99	9.52
Standardized TTR (STTR)	42.58	44.43
STTR standard deviation	56.19	54.20
STTR basis	1000	1000
Mean word length (in characters)	4.22	4.18
Sentences	7608	5532
Mean (in words)	12.47	15.77
Standard deviation	9.21	11.10
Paragraphs	1657	1094

(1) Standard Type/Token Ratio (STTR)

TTR is the ratio of the number of different words (types) to the number of running tokens in a text [88]. It is the common standard for judging lexical richness. If the two translated texts are of the same length, the higher the TTR, the higher the lexical richness the text holds. But if the text length expands, function words such as the, a, of, and etc., occupy certain amounts of the word count, thus lowering the TTR as a whole. The Standard Type/Token Ratio (STTR), which computes the TTR based on every 1000 words, is adopted when texts of different lengths are used for comparison. STTR for Harman’s two translated literature is 43.47, higher than that of the British National Corpus (BNC) (41.20) and lower than that of the Translational English Corpus (TEC) (44.63), demonstrating a lexical richness comparable with TEC, and a revelation of certain universal features of the translated language in Harman’s two translated works.

(2) Mean Word Length (MWL)

As shown in Table 3, mean word length for *I Want to Be Good* is 4.22 and *Flight of the Bumblebee* is 4.18. The average mean word length of the two translated literature is 4.20, lower than that of the Translational English Corpus (TEC) (4.36) [60] and the British National Corpus (BNC) (4.54). This indicates that Harman’s translated children’s literature features relatively shorter words and lower lexical difficulty.

(3) Lexical Density

Lexical density refers to the ratio of lexical words (i.e., running words minus function words) to the number of running words in a text [89]. Lexical density can reflect the information load, associated with features including the use of technical vs. general vocabulary, the percentage of known versus unknown information, etc. [90]. Lexical density is an important indicator of the lexical richness of the text, i.e., the higher the lexical density, the more complex the text; the more information the text carries, thus the greater the cognitive load for information processing; the lower the lexical density, the more simplified the text is. Lexical density is calculated by the formula, (content words/ whole words) ×100% [91].

**Fig 1:**
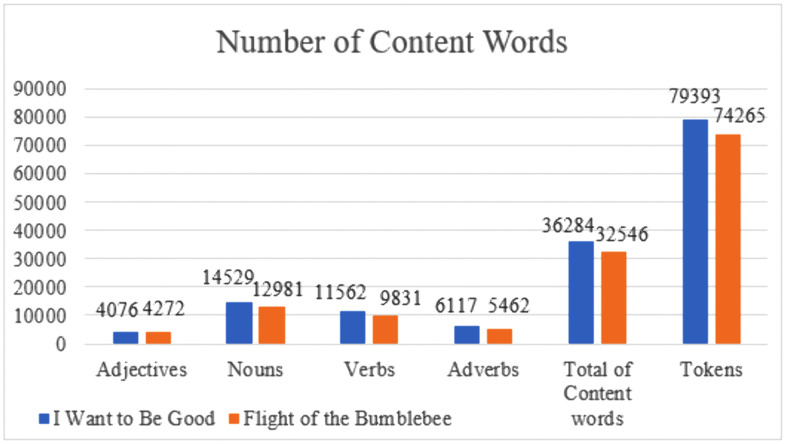
Numbers of Content Words in *I Want to Be Good* and *Flight of the Bumblebee.*

After POS (part-of-speech) tagging, the number of content words in *I Want to Be Good* and *Flight of the Bumblebee*, including adjectives, nouns, verbs, and adverbs, has been counted and calculated in AntConc 3.5.4, listed in parallel (as shown in Fig 1). Next, the lexical density of content words is calculated according to the above-mentioned formula, and the result is shown in Table 4. Lexical density in *I Want to Be Good* and *Flight of the Bumblebee* is 43.83% and 45.70%, respectively, located in the lower domain of the lexical density of English novels, i.e., 40–54% [92], indicating relatively low lexical difficulty and brevity in information load in the two translated children’s works. These characteristics are in line with the lexical features of children’s literature, featuring simple, accessible vocabulary and straightforward messages to “engage and offer a participatory experience for the reader” [93].

**Table 4:** Lexical Density in *I Want to Be Good* and *Flight of the Bumblebee.*

Parameters	Total of Content Words	Tokens	Lexical Density	Average Lexical Density
*I Want to Be Good*	32546	74265	43.83%	44.77%
*Flight of Bumblebee*	36284	79393	45.70%	

(4) Keywords analysis

Keywords are those that occur unusually frequently in comparison with a reference corpus [40]. The keyword tool in WordSmith 8.0 has been used to compare the frequencies of words in the word list in *I Want to Be Good* and *Flight of the Bumblebee* respectively with the frequencies of words in the word list in the reference corpus, BNC (British National Corpus).

Tables 5 and 6 present the top thirty keywords in Harman’s translation of two children’s literature, *I Want to Be Good* and *Flight of the Bumblebee*, with the frequency of the keyword, its frequency in the reference corpus with comparable non-translated texts, the British National Corpus (BNC), and its keyness score listed. BNC serves as the optimal normative baseline for contemporary British English, ensuring that the comparative analysis accurately reflects Harman’s idiosyncratic stylistic markers.

**Table 5:** Top thirty keywords in *I Want to Be Good.*

N	Key word	Freq.	RC. Freq.	BIC
1	Ling	1,196	243	15,736.12
2	Mom	965	262	12,475.15
3	Xing	341	2	4,821.41
4	Ling’s	160	0	2,263.99
5	didn’t	222	2,043	1,698.94
6	Dad	230	6,584	1,262.14
7	Math	84	34	1,038.19
8	Xiaoli	72	0	1,008.67
9	Exam	120	882	960.44
10	I’ll	66	0	923.08
11	couldn’t	106	552	913.58
12	school	315	38,483	876.60
13	Mom’s	59	0	823.22
14	wasn’t	101	873	774.82
15	student	142	7,793	595.64
16	silkworm	44	16	539.68
17	they’d	39	0	537.92
18	what’s	44	22	525.26
19	She’s	38	0	523.66
20	Yaru	38	0	523.66
21	They’re	35	0	480.86
22	grade	86	2,605	451.07
23	grandma	59	489	449.61
24	we’re	32	0	438.07
25	He’d	31	0	423.80
26	Yuan	58	580	421.17
27	Lin	47	184	418.95
28	wouldn’t	59	662	415.88
29	There’s	31	1	414.90
30	eye	120	10,021	405.99

**Table 6:** Top thirty keywords in *Flight of the Bumblebee.*

N	Key word	Freq.	RC. Freq.	BIC
1	Kejun	133	0	1,902.22
2	Sheona	96	0	1,367.91
3	couldn’t	101	552	878.39
4	Dad	164	6,584	816.32
5	He’s	49	1	679.38
6	Mum	150	8,188	657.03
7	Huaxi	46	0	645.86
8	I’ll	44	0	616.98
9	Kid	90	1,695	570.63
10	Chengdu	42	16	519.80
11	Pomegranate	38	30	437.06
12	Tianlu’s	29	0	400.37
13	She’d	27	0	371.49
14	We’d	27	0	371.49
15	You’ll	27	0	371.49
16	wasn’t	56	873	368.42
17	uncle	77	3,462	356.80
18	Tao	35	74	350.29
19	eye	105	10,021	344.16
20	piano	64	1,945	341.55
21	school	178	38,483	337.63
22	They’re	24	0	328.17
23	Yusheng	24	0	328.17
24	Xu	31	49	322.50
25	make	244	78,980	311.45
26	I’ve	21	0	284.84
27	There’s	21	1	276.71
28	You’ve	20	0	270.40
29	student	79	7,793	249.56
30	They’d	18	0	241.52

From the analysis of keywords, the following findings can be generated accordingly:

1) Translation of character names

Transliteration, the adoption of the standard Mandarin Chinese pinyin system, has been best employed in the translation of characters’ names in Harman’s translation practice of two children’s literature. This strategy retains the sound of the name, thus conveying the name’s foreignness to the target readers. This has been further explained in Harman’s Translator’s Foreword of *Flight of the Bumblebee*, “I’ve used their given names for all the children. So Shen (surname) Tianlu (given name) is referred to as Tianlu. [94]”, providing enough information for the cultural connotation for the naming conventions in Chinese for the target young readership. But there is one exception in the translation of the narrator’s name in *Flight of the Bumblebee*, as stated in the Translator’s Foreword: “Orange is the only name I actually translated,” and the reason is that the author has clarified that she chose this name because it suits a bright, sparky character [94].

2) Frequent use of contracted forms

Contracted forms (’s, ’d, ’ll, ’ve, etc.) has occupied certain positions in the keywords of Harman’s translated children’s literature. Contractions are generally regarded as important markers of informality, as demonstrated in various studies investigating formality and informality [59,95]. This demonstrates Harman’s tendency to preserve the informal style in children’s literature, and the choice of simple sentence structures, such as existential sentences and link verbs, has enhanced the clarity of the grammatical structures.

3) Collocation of high-frequency words or keywords

1. Ling and Tianlu

**Fig 2:**
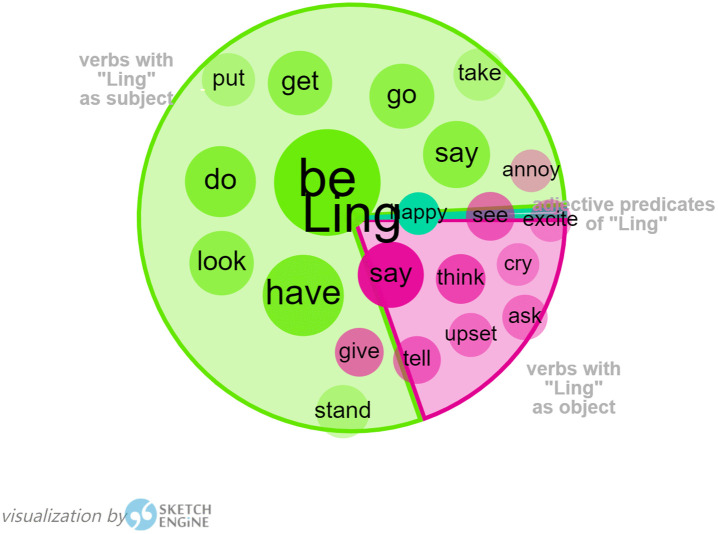
Visualization of Ling by Sketch Engine.

Word Sketch in Sketch Engine has been used to generate the collocations of “Ling”, in order to find out more about Harman’s portrayal of Ling in her translation. As Ling is the main character in *I Want to Be Good*, verbs with “Ling” as the subject, adjective predicates of “Ling”, and verbs with “Ling” as the object can all be used to map out Ling’s characterization (as shown in Fig 2). As shown in the graph generated in Sketch Engine, the closer a collocate is to the center, the more typical it is; the larger the circle size, the more frequent the word is; and the larger the segment size, the more collocations it contains compared to other visualized relations. These verbs following Ling as the subject, mainly include “be”, “have”, “say”, “do”, “look”, “get”, “take” and “put”, which are used to describe Ling’s action and state of mind. The adjective “happy” as active predicates of “Ling”, is a clear indication of Ling’s easy-going and optimistic characteristics, the same as the original portrayal of Ling. Verbs with “Ling” as object are composed of “think”, “give”, “tell”, “upset”, “ask”, “cry”, “see”, “excite”, and “annoy”, presenting a clever Ling with a keen observation and emotional disclosure in daily life.

**Fig 3:**
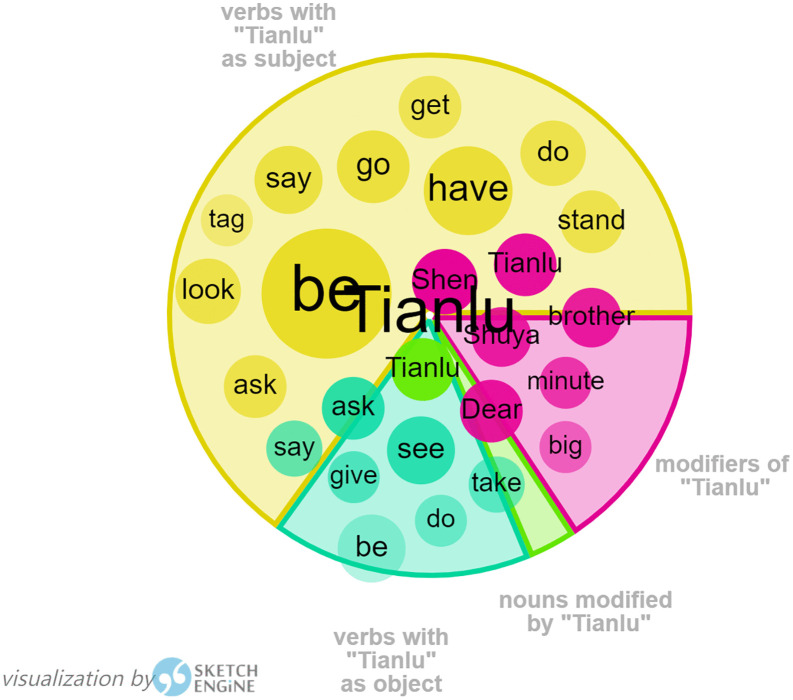
Visualization of Tianlu by Sketch Engine.

Similarly, Word Sketch in Sketch Engine has been used to generate collocation of Tianlu, in order to find out more about Harman’s portrayal of Tianlu in her translation, as shown in Fig 3. “Dear”, as a modifier of “Tianlu”, serves to express a close relationship with the word “Tianlu” and appears with relatively high frequency. The concordance analysis shows that this is the result of several letters to Tianlu, starting with “Dear Tianlu”, written by Orange, the narrator, to express her sincere wish for her brother’s good luck, as danger may befall at any time while her brother is piloting a plane to fight against the enemy.

From the above analysis, Harman’s representation of the characteristics of Ling can be the reproduction of Ling in the original Chinese. Tianlu in *Flight of the Bumblebee* has also been portrayed in the same way as in the original Chinese, who was a shy and sensitive boy when he first came to Orange’s family and became a member of her family, and then he has grown up to be a responsible and promising boy with his fast progress in study. This has shown the translator’s respect for the original text.

2. collocation of “look”

Consider the collocation of “look” as an example. Harman’s tendency to follow the original Chinese syntactic structure is clearly demonstrated in her preservation of the order and construction of Chinese serial verbs. This demonstrates Harman’s foreignization strategy of retaining the original flavor of Chinese syntax, particularly in daily conversation for communicative purposes. In the following example (Example 1), “来看” has been rendered as “Come and look! Come and look” repetitively to reinforce the appellative function of the original Chinese serial verbs. By changing the original descriptive phrase into two imperative sentences, Harman has increased the vividness and emotional appeal for children’s verbal expression.

Example 1

**Table:** 

Source Text: 还有人大喊大叫, 招呼更多的同学来看 [96]
**Literal Translation**: Some people were shouting loudly, calling out for more classmates to come and have a look.
**Target Text:** She could hear shouts, “Come and look! Come and look!” [97]

3. high-frequency word “good”

According to the word list function generated by WordSmith 8.0, the word “good” ranks 15^th^ in the wordlist of *I Want to Be Good,* and is the first adjective to appear in the list. As “good” is the main adjective used for the characterization of Ling, and “Being Good” is her ultimate goal in response to her mother’s sincere wish for her to get good grades and make progress in her studies, the collocation of “good” provides a useful test of Harman’s representation of the original text.

**Fig 4:**
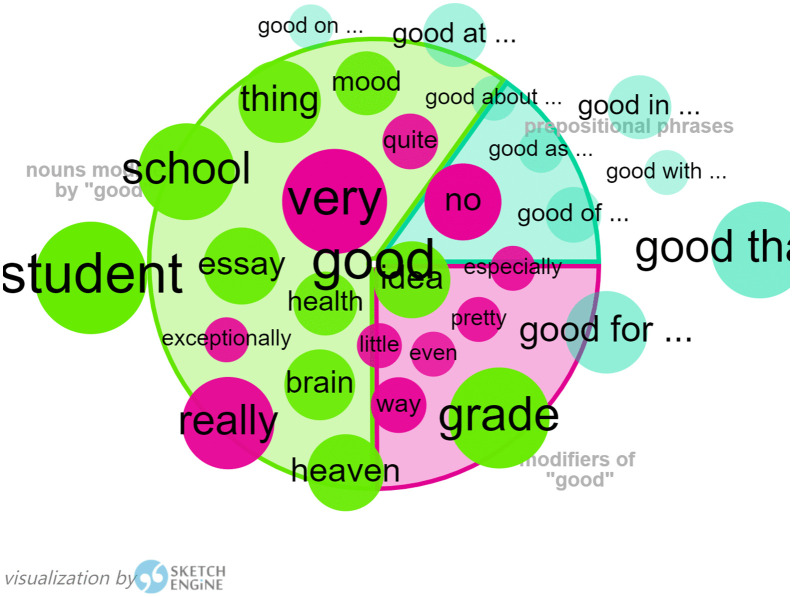
Visualization of “good” by Sketch Engine in *I Want to Be Good.*

As shown in Fig 4, nouns modified by “good” are the words for school, such as student, essay, school, grade, idea, etc. and gradable adjectives, such as exceptionally, really, very, quite, no, little, pretty, especially, even, etc. and prepositional phrases, such as good at/ on/ in/ with/ of/ about/ for/ that. In addition, there is a tendency for domestication for the emotional expression to interpret surprise “天哪 !(Tian Na)” as “Good heavens”, suggesting Harman’s adaptation to the reading habits of the target English speakers.

**Fig 5:**
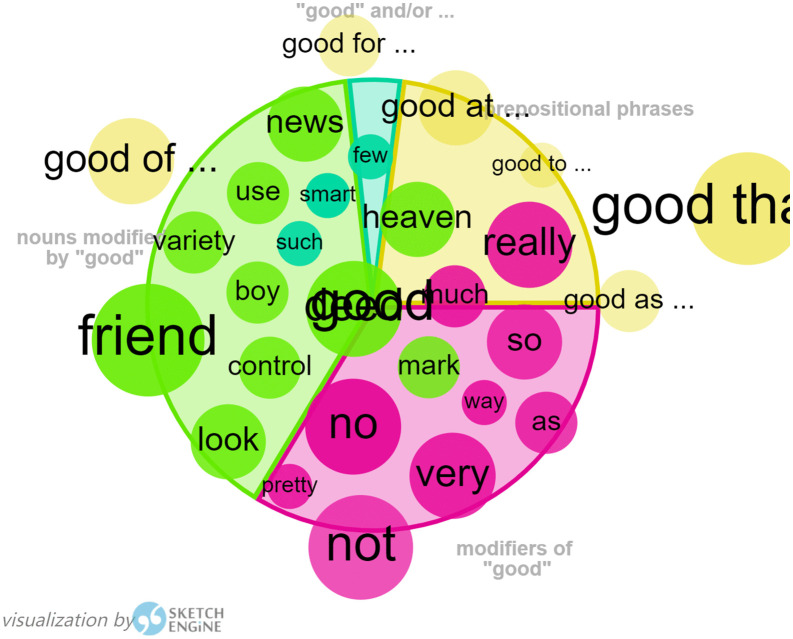
Visualization of “good” by Sketch Engine in *Flight of the Bumblebee.*

Similarly, the first adjective “good” ranks 17^th^ in the wordlist of *Flight of the Bumblebee*, demonstrating Harman’s tendency to use simple words effectively. This has two-fold meanings. On the one hand, translating children’s literature requires the language to be easy and vivid, lowering the reading difficulty. On the other hand, the diversified collocation of “good” as shown in Fig 5, functioning as infinitive objects in “good enough to” (take, read, offer, etc.), and modifiers of “good”, such as so, no, pretty, very, as…as, etc., have shown Harman’s tentative efforts in describing the minor psychological activities of characters.

In addition, a careful examination of the collocation of “good” reveals that the frequency of “good deed” (7 times, as shown in Table 7) is unusually high compared to other common collocations. By consulting the bilingual corpus, all instances of “good deed” refer to “行善” (to practice virtue), an activity of the Youth Militia in Chapter 6, aimed at encouraging children to make their own contributions during the anti-Japanese war, in which all four children from the narrator’s family participated. This helps to reinforce Harman’s tendency to simplify certain complicated expressions and phrases for the target young readers.

**Table 7:** Examples of “good deed” in Harman’s translation of Huang’s *Flight of the Bumblebee.*

No.	Harman (2023)
1	The Youth Militia had us doing ‘A **Good Deed** A Day’ [94].
2	But we primary school kids couldn’t compete with the high school students as far as **good deeds** went [94].
3	It was not easy to find a **good deed** to do, but that made me all the more determined [94].
4	Anyway, I just couldn’t find a **good deed** to do [94].
5	‘When you help someone in the family, that’s not a **good deed**, it’s your duty.’ [94]
6	She reminded me, ‘You could help your mother. That’s a **good deed**.’ [94]
7	I’m doing a **Good Deed** a Day, not looking for a husband! [94]

4. keyword “eye”

**Fig 6:**
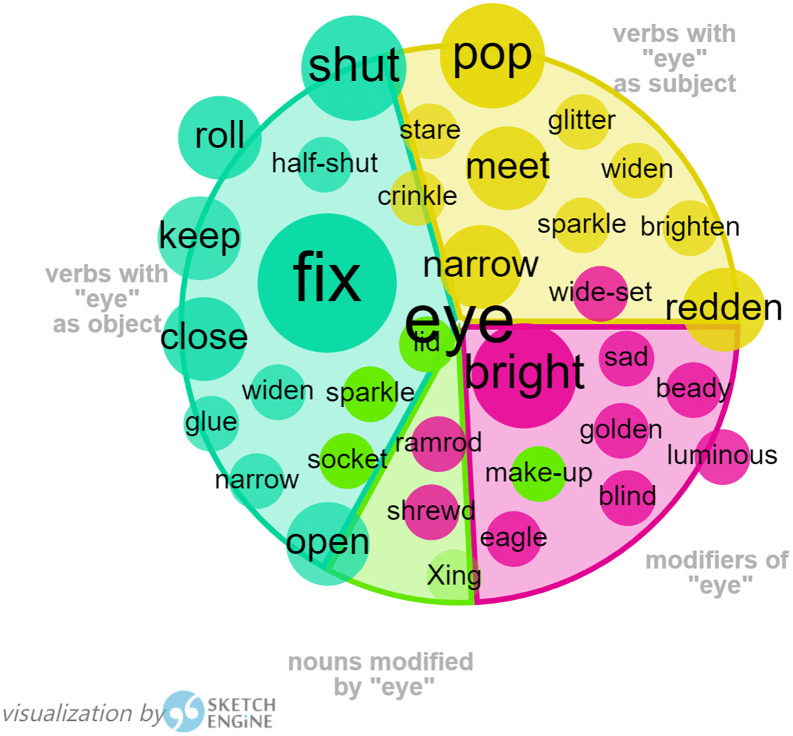
Visualization of Eye by Sketch Engine in *I Want to Be Good.*

**Fig 7:**
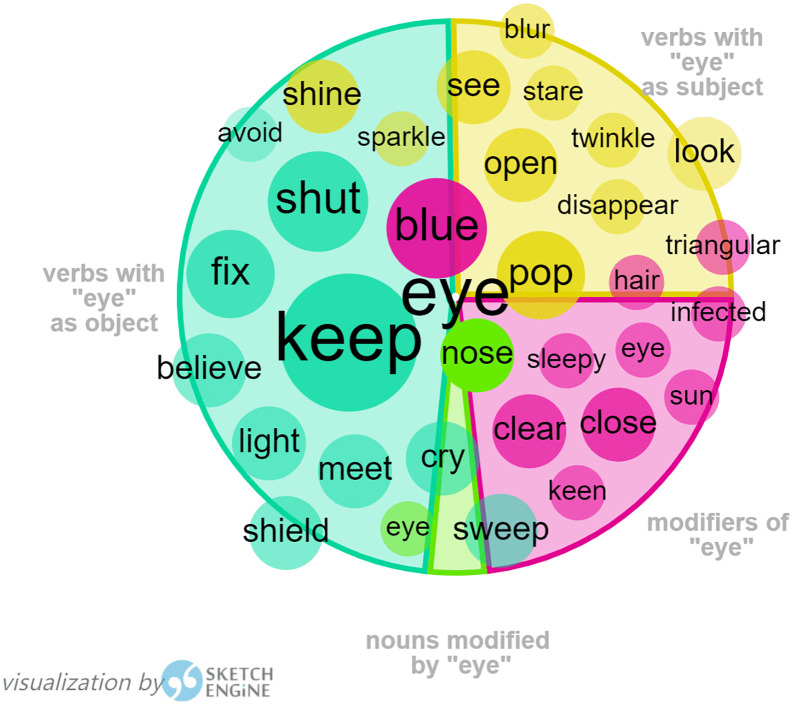
Visualization of Eye by Sketch Engine in *Flight of the Bumblebee.*

The abstract meaning of “eye” in certain collocations, as shown in Figs 6 and 7, has been frequently identified in both of Harman’s translations of the two children’s books, *I Want to Be Good* and *Flight of the Bumblebee*, such as “keep an eye on sb.”, “in the blink of an eye”, “as far as the eye could see”, etc. This shows Harman’s habitual use of collocations to realize its contextual meaning.

There is a famous line by the patriotic poet Lv You (陆游) in Southern Song Dynasty, in which the poet has expressed his profound longing for the unification of the nation and his undaunting attitude towards death, “死去元知万事空 但悲不见九州同” (Upon my death, I am aware that all things will become void, yet I lament that I shall not witness the unification of the nine provinces). Harman rendered this line into “My dying eyes see only dust/ My sole regret that China is divided”, in which she has conveyed the most intense emotion with the fewest words, highly concise yet rich in meaning, making it accessible and understandable to young readers. The tactical use of “my dying eyes” as the subject in the beginning and “my sole regret” as the subject in the next instance is parallel in structure and employs alliteration in its lyrical rhyme, resonating with the original poem both emotionally and aesthetically, representing a successful attempt at interpretive translation.

(5) Four-character Chinese idioms

Regular expression “\b[^\x00-\xff]{4}\b” has been entered as the Search Terms (Select Regex) in Concordance interface in AntConc 3.5.4, generating the total count of four-character expressions adopted in Harman’s translated versions *I Want to Be Good* and *Flight of the Bumblebee*.

Then, the retrieved four-character Chinese idioms were classified into two main categories: reduplicated four-character idioms and culturally specific Chinese idioms. Reduplicated four-character idioms are a typical feature of Chinese four-character idioms, with rhythmic paces and archaic styles.

**Fig 8:**
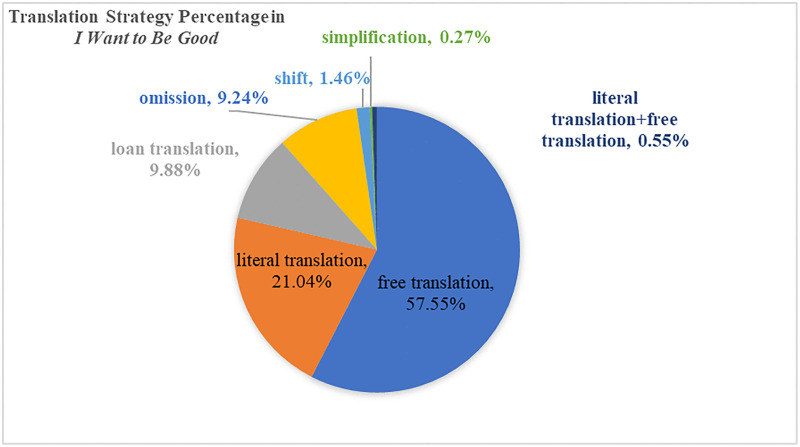
Translation Strategy Percentage in *I Want to Be Good.*

**Fig 9:**
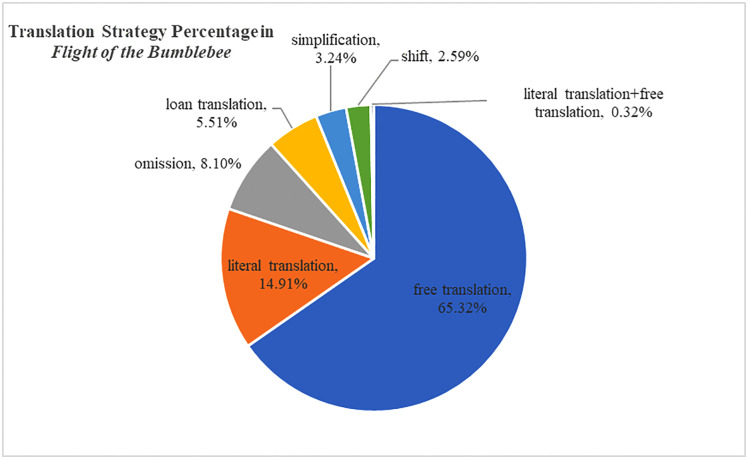
Translation Strategy Percentage in *Flight of the Bumblebee.*

Harman’s translation strategy for the four-character Chinese idioms can be basically categorized into seven ways: literal translation, free translation, simplification, loan translation, omission, shift and literal translation + free translation. The percentage for each strategy in Harman’s translated version *I Want to Be Good* and *Flight of the Bumblebee* can be demonstrated in Figs 8 and 9.

The two figures above show Harman’s stable tendency to achieve acceptability by catering to target readers’ reading habits and striving for adequacy, with literal translation as a supplementary method for free translation. Free translation and literal translation have been the major strategies for Harman in dealing with four-character Chinese idioms. Loan translation and omission rank as the third and fourth largest strategies in Harman’s choices. Shift, simplification, and literal and free translation occupy a relatively small percentage in Harman’s strategy for four-character Chinese idioms.

For literal translation, “满脸是泪(with tears all over her cheeks)”, “哭笑不得(not knowing to laugh or cry)” and “恶性循环” have been rendered as “tears running down her cheeks”, “didn’t know whether to laugh or cry” and “a vicious circle”. Literal translation has been employed when similar lexical features have been identified and the intended meaning is effectively conveyed without causing potential confusion. Literal translation ranks second in Harman’s translation strategy for four-character Chinese idioms, demonstrating her preference for accuracy in conveying meaning.

Phrases like “掉以轻心(literally, treat it with a light heart, i.e., be careless about something)”“压轴节目(literally, the anchor act)”“拿手绝活(literally, special skill)”“诅咒发誓 (literally, to curse and swear)” have been domesticated into “Be on your guard”, “a grand finale”, “have … in the palm of his hand” and “swearing that by hook or by crook”, in the pursuit of achieving similar effect of the original four-character idioms among the target international readership.

Domestication, as described by Venuti [98], involves “an ethnocentric reduction of the foreign text to target-language cultural values, bringing the author back home”, aiming for transparency and readability. The employment of domestication typically results in the use of existing English idioms, functional equivalents, or sense-for-sense translation, effectively obscuring the linguistic and cultural difference of the source text. In this sense, translator mediates culturally specific items by substituting a familiar cultural reference for an unfamiliar one, thereby ensuring the immediate rhetorical function of the four-character Chinese idioms is preserved in the target culture.

When translating four-character Chinese idioms, Harman’s nuanced approach to balancing domestication and foreignization is evident. Although she demonstrates a tendency towards domestication in her treatment of four-character Chinese idioms, in her two translated children’s literature, this balance is ultimately achieved through a strategic combination of literal translation and a literal-plus-liberal approach. This approach serves to both explain and preserve the original flavor of the culture-specific items for the target English young readers.

(6) Onomatopoeia

As one of the powerful literary devices, onomatopoeia carries rhythmic sound and represents the musicality of the text. In children’s literature, onomatopoeia stimulates their imagination and capture children’s attention. In Harman’s translation, she has represented onomatopoeia in the translated children’s literature, preserving the original rhythmic features.

There is a children’s war song “铲东铲东铲” in Chinese, commemorating the bravery and sacrifice of the anti-Japanese soldiers. The repetitive structure may evoke associations with a certain action or sound being reiterated, thereby generating a sense of rhythm and musicality in the auditory experience. The name of the song has been rendered as “Dig, Dig, Dig for victory”, enhancing the continuous action of shoveling, reproducing the original rhetorical features, such as repetition (dig for three times), onomatopoeia (imitating the sound of shoveling) and symbolism (the repetition of the action underscoring the resoluteness of the action or determination) contained in the original name. Harman’s rendering of this name has not only reproduced the rhythmic phonetic effect, but also made the implicit symbolic meaning (for victory) concise and straightforward.

In addition, Harman has adjusted the sound in the original context to one that is more contextually appropriate in the target language, for example *pa*, a sharp, snapping sound in “啪的一响”, rendered into “- ping! -”, imitating the sound of the chalk hitting Hai, a naughty boy in Ling’s class, which enhances the vividness of the scene.

“脚步跺得地面咚咚响” has been rendered as “Thud, thud, thud, went her feet.” The repetitive use of the onomatopoeic word “thud” in the phrase, has captured the sound of heavy footsteps pounding on the ground, just like the original Chinese phrase. By adopting an inverted sentence structure (… went her feet), the sound created by the action is emphasized. Harman skillfully represents the original onomatopoeia in the following example.

Example 2

**Table:** 

ST:邢老师骂人像鸡婆, 咕咕咕咕不停;数学张老师骂人像乌鸦,全班人鸦雀无声时,他冷不防嘎的一叫…… [96]
Literal Translation: Mrs. Xing scolds like a hen, clucking away non-stop; Mr. Zhang, the math teacher, scolds like a crow, and when the whole class is quiet, he suddenly caws.
TT:Mrs. Xing scolds like a mother hen, *cluck*, *cluck*, *cluck*! The math teacher Mr. Zhang scolds us like a crow, he waits until we’re all quiet, and then he goes, *caw*, *caw*! [97]

In the above example (Example 2), the repetition of cluck three times and caw twice in Harman’s translation, which mimic the sound of a mother hen and a crow respectively, vividly reproduce the original rhetorical feature. Additionally, the italics for the sounds “cluck” and “caw” have been used to make the onomatopoeia prominent and attract children’s attention. When compared with the original text, Harman’s addition of the sound of the crow has shown her effort in imitating the sound of animals and creating joyful atmosphere for the target readership.

(7) Flexible treatment of culturally-loaded terms, slang and dialects

Harman has demonstrated a flexible and context-sensitive approach to the English translation of culturally-loaded terms, slang, and dialects through the coordinated use of (1) adaptation, (2) omission due to cultural vacancy, (3) intra-textual amplification, (4) paraphrasing some slangs and dialects, and (5) translator’s subjectivity.

Adaptation allows Harman to replace culturally specific images or allusions with functionally equivalent expressions in English. For example, “去年的皇历今年能翻吗？ (literally, can we turn the pages of last year’s almanac this year?)” has been rendered as “Last year was last year”, without explaining the traditional Chinese almanac (皇历, *huangli*) to the target young readers, which was used to determine auspicious dates for daily activities. When confronted with cultural vacancies, Harman occasionally resorts to partial omission or condensation as a remedy, to reduce cultural bumps and lower the difficulty of understanding the translated text for the target young readership, which can be demonstrated by the deletion of certain cultural symbols, such as Yang Yuying (a renowned Chinese pop singer especially in the 1990s, known as the “Queen of Sweet Songs”).

Intra-textual amplification serves as a complementary strategy to make up for necessary cultural backgrounds or contextual information and layered meaning. For example, without mentioning the culture-specific term “小脚(*xiaojiao*)”, intra-textual amplification has taken the form of brackets, distinguishing from the original narration, to work out a proper explanation of “裹脚” (*guojiao*, bind feet) for the target young readers:

Mum sat on the ground and bemoaned her useless feet. (They’d been bound when she was a small girl, then unbound, but the bones hadn’t healed and she still had difficulties walking.) [94]

Paraphrasing has been regarded as “the translator’s last resort” [99], such as Harman’s rendering of “砸锅卖铁！”(literally, meant to smash one’s pots and pans and sell the iron) into “We’ll do whatever it takes!” to show the resolution of Ling’s mother to pave a good way for her daughter’s education.

Translator’s subjectivity can be shown in Harman’s agency in translating Sichuan dialect in children’s literature. Harman has shown creative interpretation of certain Sichuan dialect in representing the humorous effect conveyed by the homophones, such as the rendering of “要的” “摇堆” into “Awesome” and “Ow-sum” [94], extending the linguistic flexibility to allow for more vivid and expressive communication, and transfer the original wisdom in a new context.

Overall, at the lexical level, Harman’s tendency to reproduce the vividness and liveliness in the original children’s literature has been featured by a lower mean word length, a lower lexical density, the choice of contracted forms, and habitual use of collocations. In addition, a relatively high proportion of culture-bound lexical items to balance between domestication and foreignization, together with the reproduction of onomatopoeia and the flexible treatment of collocational expressions contribute to Harman’s translator’s style.

### Translator’s style manifested at the syntactic level

(1) ASL(Average sentence length)

Harman’s translations of children’s literature are characterized by concise and vigorous sentence structures. According to Table 8, the average sentence length in her translations is relatively short: 12.47 for *I Want to Be Good* and 15.77 for *Flight of the Bumblebee*, which aligns well with the syntactic features of children’s literature. This brevity not only facilitates comprehension for young readers but also mirrors the lively and dynamic nature of children’s language. By using shorter sentences, Harman effectively captures youthful expression, making her translations both engaging and accessible. Such a linguistic approach is crucial in children’s literature, where simplicity and clarity are often prioritized to resonate with the target audience.

**Table 8:** Average sentence length of Harman’s two translated children’s literature.

No	Text file	Mean (in words)	Std. dev
Overall		13.86	10.18
1	*I Want to Be Good*	12.47	9.21
2	*Flight of the Bumblebee*	15.77	11.10

The English translation of *Flight of the Bumblebee* presents a higher average sentence length (15.77) than *I Want to Be Good* (12.47). This syntactic feature can be attributed to the complexity of the sentences employed to depict its historical backdrop, which is set against the backdrop of the War of Resistance against Japanese Aggression. The intricate nature of the historical description necessitates the use of more elaborate, polysyllabic words to convey the multifaceted context accurately and vividly. This contributes to the higher average sentence length in the translation.

(2) Sentence translation ratio

The sentence translation ratio can be defined as the proportion of the number of translated sentences to the number of original sentences. It reflects the extent to which the translator has segmented or restructured the sentences of the original work.

According to Table 9, the average sentence translation ratio of Harman’s two children’s literature translations is 1.386:1. Moreover, the sentence translation ratios of the two works are quite similar. This indicates that Harman has carried out a certain degree of sentence segmentation in the translation of both works, and the extent of sentence segmentation is relatively stable. It is clear that Harman has considered the reading habits and comprehension levels of the target language children. She has aimed to segment long sentences as much as possible to ensure a relaxed and enjoyable reading experience for young readers.

**Table 9:** Sentence translation ratio of the two children’s literature.

Parameters	*I Want to Be Good*	*Flight of the Bumblebee*
Sentences in the translated text	7624	5548
Sentences in the original text	5330	4133
Sentence translation ratio	1.430:1	1.342:1
Sentence translation ratio in average	1.386:1	

Based on the above syntactic analysis, Harman’s two translated children’s literary works have a relatively low average sentence length. Harman’s translations are characterized by notable brevity and clarity, utilizing a simplified, non-complex syntactic structure to enhance readability and suit the reading habits of the target young audience. The sentence translation ratio has indicated that Harman has segmented sentences to a certain degree in her two translated works, thereby reducing the reading difficulty for the target young readers.

### Translator’s style manifested at the textual level

(1) Paragraph translation ratio

**Fig 10:**
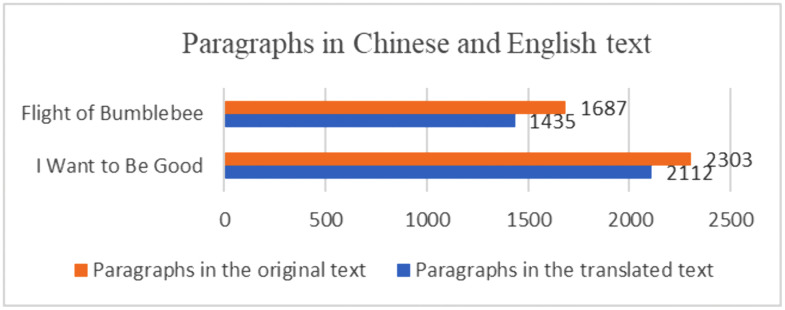
Paragraphs in the Chinese and English text.

**Fig 11:**
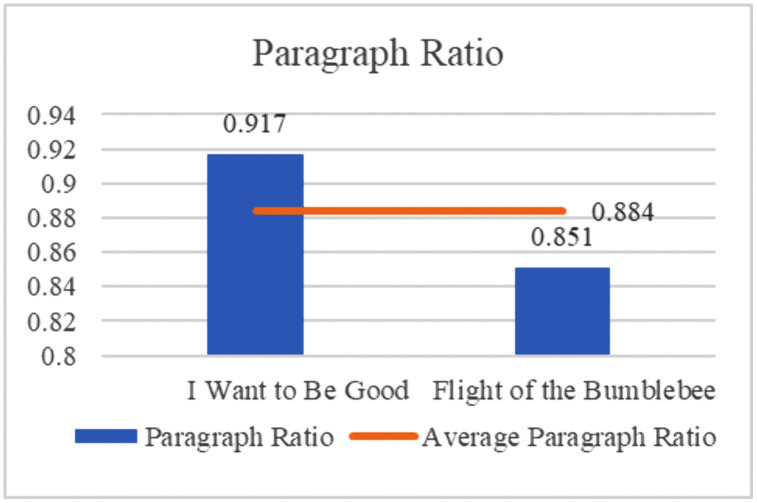
Paragraph translation ratio in the two translated texts.

Paragraph translation ratio is calculated by comparing the number of paragraphs in the translation to the number of paragraphs in the original text (as shown in Fig 10), i.e., the ratio of English paragraphs to Chinese paragraphs (see Fig 11). The English-to-Chinese paragraph ratio reflects how the original paragraphs are presented in the English translation, demonstrating the extent to which the translator has divided or restructured the original paragraphs.

The average paragraph translation ratio of Harman’s two translated children’s literature is 0.884 (as shown in Fig 11), a clear indication of the existence of combination and adjustments (including the deletion of certain paragraphs) in Harman’s translation. Based on a close reading and examination of the original and translated texts of *I Want to Be Good* and *Flight of the Bumblebee*, it has been found that some paragraphs containing significant cultural gaps or negative information, potentially impeding young readers’ understanding, have been omitted during the translation process, thereby alleviating potential cultural shocks for the younger generation. One example is the omission of an entire paragraph describing the household allocation at Ling’s father’s school in *I Want to Be Good*, which may be beyond the comprehension of young readers. The most representative omission is the deletion of one paragraph and three sentences, stating the fate of Orange’s, the narrator, sister and brothers in the Prologue of Huang’s *Flight of the Bumblebee*, not only creating suspense and arousing children’s curiosity in finding out the miserable fate of Orange’s sister and brothers, but also mitigating the emotional impact directly on the target children readers, instead of revealing the tragic endings of the main characters at the beginning of the narration.

These efforts are made for the readability and reception of children’s literature in translation. All these efforts in combining, adjusting, and deleting certain paragraphs result in a tightly knit framework for the translated text, thereby reducing the burden on young readers.

(2) Readability

Readability norms refer to measurable or qualitative expectations about how comprehensible a text should be for a given audience. Lexical simplification and syntactic simplification contribute to readability norms. In addition, oral readability, i.e., the capacity for texts to be effectively read aloud, holds significant value, ensuring engagement and comprehension among young audiences.

As for stylistic norms, the reason for the high style in children’s literature is connected with the didactic concept of literature and the attempt to enrich the child’s vocabulary [70]. This falls in line with the representation of theme (to be good not only in study but also in behaviors, and to be strong in the war time and cherish your current life) in Harman’s two translated children’s literature and explains Harman’s choice of balancing domestication and foreignization in her treatment of four-character Chinese idioms, with the intention of introducing more foreign flavor into children’s vocabulary.

According to Polysystem Theory, elaborated on for children’s literature by Zohar Shavit, children’s literature occupies a peripheral position in the literary canon [70]. Because it is often viewed primarily as an educational or socializing tool rather than “high art”, it is subject to much stricter constraints than adult literature. Translators are expected to manipulate the source text significantly to fit the target child’s perceived cognitive capabilities and reading level. Complexity, ambiguity, and excessive foreignness are often viewed as obstacles to readability.

In Toury’s initial norm, the decision regarding the translation strategy, which takes priority over operational norms, must be made between adequacy, i.e., adherence to source norms, and acceptability, i.e., adherence to norms originating in the target culture [100]. As stated by Huang [48], readability is taken as an indicator of translator’s style.

**Fig 12:**
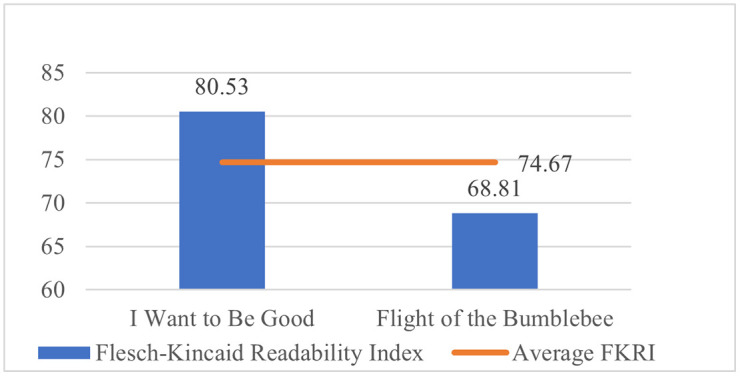
Flesch-Kincaid Readability Index of the two translated texts.

**Fig 13:**
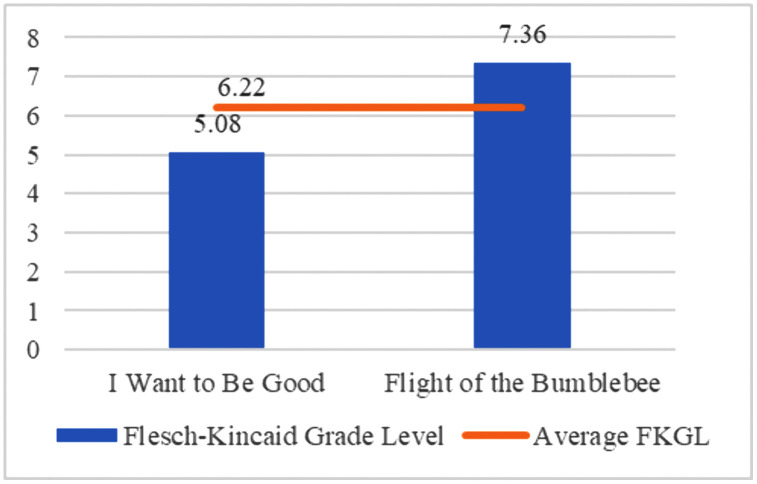
Flesch-Kincaid Grade Level of the two translated texts.

Qt Readability has been employed for the analysis of readability and the results of Flesch-Kincaid Readability Index and Flesch-Kincaid Grade Level have been shown in the following figures (Figs 12 and 13).

As shown in Fig 12, the average Flesch-Kincaid readability index of Harman’s two translated children’s literature is 74.67, which falls within the range of 70–79. According to Table 10, Harman’s translation of the two children’s literature can be categorized as “Fairly Easy” in terms of reading difficulty. This indicates that the overall difficulty of the two translated texts is relatively low. Harman’s translated works are simple and easy to understand, which is in line with the characteristics of children’s literature.

**Table 10:** Flesch-Kincaid Readability Index and Text Readability Difficulty.

Score	0-29	30-49	50-59	60-69	70-79	80-89	90-100
Level of Difficulty	Very Confusing	Difficult	Fairly Difficult	Standard	Fairly Easy	Easy	Very Easy

As shown in Fig 13, the average Flesch-Kincaid Grade Level of Harman’s two translated works is 6.22, an indication of a difficulty level for Grade 6–7 students. For *I Want to Be Good*, the Flesch-Kincaid Grade Level is 5.08, suitable for students in Grade 5–6. This rightly aligns with the difficulty level of the original Chinese version, marked as Grade 5–6 in the book cover. As for *Flight of the Bumblebee*, the score is 7.36, resulting in a slightly higher reading grade level, grades 7–8. This is due to its background in the War of Resistance against Japanese Aggression and the substantial amount of information contained in the text.

Overall, it remains in the category of works with relatively low reading difficulty. The readability level for Grade 6–7 students (approximately 11–13 years old) indicates an alignment with late elementary to early adolescent developmental stages. Learners within this age group, typically demonstrate automatized decoding, expanding vocabularies, and improved capacity for inference and critical evaluation of narrative texts. The well-chosen concise, dynamic words, phrases and sentences in Harman’s translation, achieved by means of sentence segmentation and careful rhythm, etc., support fluent reading and comprehension for young readers in this age group, while retaining sufficient linguistic complexity to provide appropriate challenges.

Standard theories of domestication would suggest that retaining foreign cultural terms reduces readability. However, Harman reinterprets the norm. Instead of erasing the foreign element to improve readability (domestication), she employs intra-textual paratexts as scaffolding devices.

Harman internalizes the linguistic norms of readability while simultaneously negotiating the cultural norms of readability. By reducing sentence length (segmentation) and choosing simpler vocabulary (low lexical density), Harman is aligning her translation with the target audience’s cognitive processing capacities.

At the textual level, Harman’s two translated children’s literature are generally easy for young readers to comprehend. Meanwhile, Harman has effectively integrated and adjusted the paragraphs from the original works, aligning with the characteristic of conciseness in children’s language.

## Results and discussion

In this part, Harman’s translator’s style at the lexical, syntactic and textual levels will be briefly summarized and followed by an exploration of reasons for her translator’s style.

### Summary of translator’s style

By addressing the psychological factors and readability, Harman’s translation of children’s literature reproduces the vividness, purity, and joy reflected in the original work. The analysis at the lexical, syntactic and textual level of Harman’s two translated children’s literature has demonstrated the existence of translator’s style and the stability in translator’s style.

At the lexical level, the richness of Harman’s translation is comparable to that of the Translated English Corpus (TEC). The lexical density of Harman’s translation is relatively low, indicating that her works feature low lexical difficulty and concise textual information. Through a detailed key words analysis, it is found that Harman tends to choose contracted forms and simple sentence patterns. In addition, transliteration is primarily used in Harman’s translation of proper nouns to best retain the original foreign flavor. Thus, the vividness and liveliness of the original children’s literature have been retained and represented in Harman’s translated works.

At the syntactic level, Harman’s two translated children’s literary works are characterized by clear and dynamic sentence structures, achieved through sentence segmentation, which reduces the reading difficulty for young readers.

At the textual level, Harman’s translations of children’s literature are easy for young readers to understand, with paragraph structures skillfully reorganized and adapted to the brevity typical of children’s language.

Harman’s translator’s style is stable across the two translated children’s works, with similar STTR, MWL, ASL, her tendency towards a balance between domestication and foreignization in four-character Chinese idioms and identical readability level of her translated works. Simplification in Harman’s translation of two children’s literature is characterized by her versatile application of high-frequency lexis and the evocative power of minimalist diction (such as the flexible collocation of “good”, “look”, and “eye”), thus bridging the cultural gap for young readers.

In *I Want to Be Good*, Ling’s development, from an ordinary, slightly “sloppy” girl to a self-conscious “good child” under China’s exam regime, is carried by a stable and recognisable translator’s voice that is accessible, conversational, empathetic, and gently ironic. In this way, the maintenance of a light-hearted tone creates a similar emotional atmosphere for English-speaking readers.

In *Flight of the Bumblebee*, Harman systematically employs contractions, direct conversational address and vivid similes to sustain the illusion of a spoken memoir, and at the same time, clarifies reference to keep dense historical details accessible to young English-speaking readers. Harman favors idiomatic, conversational English in dialogue and interior monologue, but she also infuses the descriptive passages with a touch of archaism and a slightly more literary tone. In this way, characters speak in a register intelligible to contemporary young readers, while the narrative voice carries a faintly historical sense that signals temporal distance from the Anti-Japanese War era. Harman mediates between Chinese socio-historical specificity and the norms of children’s fiction in the English-speaking countries, guiding young readers through unfamiliar cultural and historical story-telling.

Furthermore, the existence of the translator’s style is further confirmed by her understanding of the translator’s voice. Harman holds that “each translator needs to make choices, and those choices make up her/his own ‘voice’”, as she believes that “every translator’s choice of language is unique (and that’s without considering the differences between the different Englishes, Australian, British, American…) [101]”.

### Reasons for translator’s style

As Laviosa [102] points out, three factors influence the features of translated language: the constrained cognitive processing that occurs during translation, translation’s communicative role, and translators’ awareness of their sociocultural roles and positions. This holds true for the explanation of translator’s style, and reasons for translator’s style will be explored from the macro-level, such as the socio-cultural factors and the micro-level, including translator’s personal preferences and awareness of translator’s role in translating for children’s literature and translator’s view on translation.

#### Socio-cultural factors: Lower position of translated literature from non-Western authors in the target literary system.

When translated literature occupies a peripheral position within a polysystem, translated literature becomes a major factor of *conservatism* [103]. A sharp contrast of the position occupied by English literature and Chinese literature within the map of world literature may increase the difficulty of translated Chinese literature coming into English-speaking countries, and especially in the case of translated children’s literature, this inferiority has driven translators to adopt flexible translation strategies to make the translation more accessible and readable for the target young readers. Harman has employed cultural filtering of certain culture-specific expressions and chosen to omit or delete paragraphs that may be difficult for the target young readers’ comprehension. This helps explain Harman’s translator’s style at the syntactic and textual levels, with the choice of simpler cohesive devices, etc.

The “lower position of translated literature” emerges in the corpus as (a) lexical simplification (a lower mean word length, and a lower lexical density), (b) syntactic simplification (a lower average sentence length and relatively frequent sentence segmentation), and (c) alignment with translator-mediated stylistic tendencies (recurrent metaphorical and collocative use of eye, reduced four-character Chinese idioms and the reproduction of onomatopoeia with good rhythm). For example, the socio-cultural influence on Harman’s translation of children’s literature can be reflected on her translation strategy employed in her treatment of four-character Chinese idioms, as a balance between domestication and foreignization, has been her major translation strategy to cater to target readers’ cognitive capabilities and represent Chinese culture as much as possible. This has been identified with corpus evidence by the retrieval of four-character Chinese idioms in AntConc and the comparisons in the bilingual corpus. These features collectively support the qualitative claim that translated literature occupies a comparatively lower position in the target language system and it holds true especially for children’s literature as the readability norm for children constraints translators’ choice of words, phrases and sentence structures.

The English translation projects of Huang Beijia’s *I Want to Be Good* and *Flight of the Bumblebee* are both part of the Jiangsu Literature Translated series, with the first volume released in 2021 and the second in 2023. The sponsor is Phoenix Publishing and Media Group (PPMG), which has its headquarters located in Nanjing. PPMG stands as one of the largest and most influential publishing conglomerates in China. PPMG actively collaborates with international publishers to ensure high-quality translations and global distribution, and they are “seeking top translators to ensure the best quality translation”. The project of English translation of *I Want to Be Good* was done in partnership with UK-based prizewinning literary translator Nicky Harman, and Harman was chosen as the translator as she has accumulated rich symbolic, cultural and social capital in the field of contemporary Chinese literature translation, and she is eager to take part in translating for different genres and age groups.

#### Translator’s special consideration for the young readership: multimodal interaction.

Nicky Harman cares deeply about the young generation, which is reflected not only in her choice of children’s literature to translate, but also in her desire to encourage high school students to participate in Chinese-English translation. In a translation project called “Translation Exchange”, co-organized by Paper Republic, a non-profit platform dedicated to introducing contemporary Chinese literature, and one of the universities in Oxford, the subject of translation from Chinese was introduced to schoolchildren in England, who took part in the translation process. Harman was encouraged by the competition held in that project when their excellent translated stories and poems were submitted. In Harman’s mind, “young people are the new generation of translators” [104], and she hopes that more people will participate in translation and find ways to combine it with other professional pursuits.

A close paratextual analysis of the two translated works of children’s literature also provides insight into Harman’s consideration for the target young readers.

Harman has made full use of multimodal materials in translated children’s literature, incorporating graphics, illustrations, images, moving images, and audio in various combinations to enhance the language of children’s literature [105]. In the Translator’s Foreword of *Flight of the Bumblebee*, a QR code linking to a playlist has been provided to allow a better appreciation of the songs and musical works in the original work through a blog on Chinese Books for Young Readers. This has demonstrated the intrinsic “dynamic” feature, a feature of children’s literature [105], which has shown Harman’s intention to increase the interaction between texts and sound for a better reading experience for the target young readers.

Each chapter in *I Want to Be Good* begins with an illustration that closely aligns with the chapter’s theme, thereby enhancing the reader’s comprehension and engagement with the storytelling. Thus, the visual appeal has been added, and the universal themes of the story have been reinforced. There are mainly three types of relations between texts and images: the direct correspondence between the chapter title and the illustrations, the theme of the chapter, or a typical item in that specific chapter.

For the first type, texts and images are reciprocal, with images complementing the texts. For example, in Chapter 13, a simple sketch of a frustrated boy with his arm wrapped in a bandage for the treatment of a dislocation resonates with the chapter title “The Accident”, highlighting the tragic outcome for Ling’s classmate due to Ling’s carelessness in running. Such relations can also be observed in Chapter 14 (image of a cat corresponds to the tile “Cat or mouse”), Chapter 15 (image of big fish is closely related to the title “Dad’s giant fish”), etc., with the illustration equally the same as the name of the title.

The second type falls under the thematic summary of the chapter, such as the portrayal of a purse in Chapter 10, which symbolizes “running the household on a shoestring.” The purse, in its metaphorical use, symbolizes the hope for financial success and stability in family management. The person who is in charge of the money makes decisions to ensure the well-being of the family.

The third type involves choosing a typical item in the chapter, and this type accounts for the majority of the relationships between texts and images in *I Want to Be Good.* For example, in Chapter 1, a sketch of an eraser, ruler and pencil presents essential tools for students in class, as they are the basic elements of learning. In Chapter 2, a teddy bear appears at the beginning of the chapter to signal Ling’s sincere wish and pure heart in choosing a gift for her teacher abroad.

Nord describes text as “a communicative action which can be realized by a communication of verbal and non-verbal means [106].” By means of verbal (textual) and non-verbal (multimodal) materials, Harman has successfully achieved the communicative purpose, creating a joyful and interactive reading experience for the young readers.

In addition, multimodality can be demonstrated with the cover design and blurb of the two translated children’s literature. In the cover of *I Want to Be Good*, by combining visual softness (soft color design and rounded forms), and affirming verbal cues (“Life with Ling is always fun… but it’s never simple!”), the multimodal presentation frames the text as both entertaining and empathetic, signaling the translator’s and publisher’s concern for children’s affective needs, agency, and educational context.

**Fig 14:**
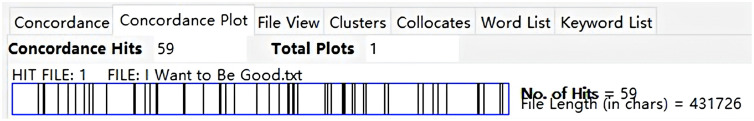
Concordance plot of the adjective “hard”.

The verbal information on the cover of *I Want to Be Good*, which reads, “Life with Ling is always fun… but it’s never simple!”, has foregrounded the story as a children’s literature with a heart lightening reading experience that is always filled with ups and downs in life. Harman’s translation depicts the challenging maturation process of Ling, a process underscored by the pressure she faces to succeed in the demanding middle school entrance examination. This can be proved by the corpus data as the word “hard” has been the second adjective (No. 196, frequency: 65), ranking after the first adjective “big” (No. 101, frequency: 93) in the keyword list of *I Want to Be Good*. In addition, the concordance plot in AntConc 3.3.0 of “hard” has shown the even display of the word over the whole story as shown in Fig 14.

The picture on the cover of *Flight of the Bumblebee* has portrayed a little girl in blue dress, looking far into the distant village, viewing the far-away peaceful and carefree life. The blue sky and the blue dress have cooperated to portray a life without war. In alignment with the cover design, the blurb on the cover of the translated work, *Flight of the Bumblebee,* which reads, “Life is good, until the war changes everything” functions as a crucial piece of paratextual evidence. This visual and verbal composition reframes the wartime narrative, rendering it emotionally accessible and psychologically safe for young readers.

By searching war related words (kangzhan, frequency: 48, zhanzheng 17, zhanqu 2, kangzhan 4, zhan 1, zhang 5, dazhang 9, kangri 2, dikang 4; 88 in total) in the Chinese original text and war related words (war, wartime, post-war) in the English translated text in AntConc 3.3.0, we obtain fewer words or expressions contain war (war, frequency 77, wartime 5, war-time 2; 84 in total) in the English target text.

Based on the corpus data, Harman reduces the frequency of war-related terms in the English target text, with fewer instances of “war” (84 occurrences) compared to the Chinese original (88 occurrences), reflecting a conscious effort to mitigate the harshness of the wartime context in the translation of children’s literature. This vocabulary adjustment demonstrates the translator’s strategy of making the content more accessible and age-appropriate for young readers, and underscores the translator’s protective role in guiding children through complex and challenging themes.

#### Translator’s view on translation.

Harman has articulated her views on translation in various online interviews with translators, in her blog series “On Translation”, and in research articles on Chinese–English translation published in leading international journals.

(1) Dual responsibility

In translating for children, Oittinen [10] attaches more importance to being “loyal” to the target readers than to being “faithful” to the source text. Van Coillie [2] argues that a balance between both should be sought, and this requires the translator to be creative enough to “make a more challenging translation, one that calls on the reader’s creative, intellectual, and aesthetic abilities”. Harman’s view on translation follows the same line as Van Coillie [2], as demonstrated in her academic article, namely, dual responsibility for translators reflected in Nord’s concept of “loyalty”, “both to the target audience, whose subjective theories have to be taken into account, and the source-text sender, whose communicative intentions must not be turned into their opposite [107].” Harman’s translation for children has captured the liveliness and joy in the original author’s work, achieving communicative effects that resonate with both the source-text author’s intention and the target audience’s expectations.

(2) A balance between domestication and foreignization

When discussing shifts and changes in the technique of translating, Bassnett & Lefevere [108] have pointed out the challenge posed by “the existence of the Other and the need to select from a number of possible strategies for dealing with that Other”. For Harman, interpreting the Other is a way to balance between domestication and foreignization in translation, even though she may not be conscious of this. In an interview titled “Translation is the art of communication and compromise,” when asked about how she deals with domestication and foreignization, Harman contends that when translating, domestication and foreignization are not what she cares about. What she is more concerned about is what the author is talking about, how he/she talks, and what effect the author intends to achieve, and she tries to match this in her translation [109]. In translating children’s literature, Harman’s translator’s style has shown her tactical balance between domestication and foreignization, in which leveling out certain culture-specific items and paragraphs serve the creation of a joyful and easy reading environment.

(3) Translator’s voice

Reading experiences in translating for children are described as “the dialogue with readers who do not yet exist for her/him, that is: imaginary projections of her/his own readerly self. [10]” In an interview with *The Chinese Weekly* [110], Harman expressed her concern for identifying a “voice” that attracts young readers before she translated *I Want to Be Good,* as this was her first time translating young adult literature. However, when she translates, the language is fluid, and she attributes this to Huang’s excellent writing. Engaging in different genres in her translation practice is one way Harman pursues high quality and diversity, as she believes that variety is the spice of life. In addition, Harman points out the difficulty in finding the corresponding school terms for American English, as grades and marks are used separately in American English and British English.

(4) Translator as an active rewriter

In Harman’s mind, to “gloss cultural references, re-order sentences within a paragraph or clauses within a sentence [101]” is a necessity for translators to achieve a natural effect in the translation. In this sense, the translator is an active rewriter. That’s why Harman has glossed some cultural references, such as the practice of foot-binding (*guojiao*), within the translated text. Harman has omitted certain phrases that may arouse potential ideological conflicts or bring complex historical facts before young readers, such as the day of Hong Kong’s Return to the Motherland.

(5) Translation as a form of self-expression and a form of authorship

Contrary to the common belief that translation is not, and can never be, a form of authorship, Harman has pointed out that “the better and richer the original work, the greater the obligation on the translator to ‘author’ its complexity into English [101]”. This arises from the differences between English and Chinese, not only in language but also in culture. Harman views translation as both self-expression and authorship, enabling greater flexibility in producing highly readable and acceptable translated works.

Across both works, Harman’s stylistic choices rest on a single, coherent ethical framework. Her central commitment that translation is an act of ethical interpretation, i.e., the dual responsibility both to the target audience and the source-text sender, shapes every decision and yields three interlocking stylistic effects that recur in both texts. First, she favors lower lexical density, lower word length, and contracted forms across her two translated children’s literature. Second, she tends to apply lower average sentence length, and simple sentence structures. Third, she keeps relatively less paragraphs in her translations compared with the original paragraphs, and text readability suitable for Grade 6–7 students. Across both translated works, Harman has identified a voice that attracts young readers, by portraying the protagonist Ling as a happy girl but with pressures from her middle school entrance exam, and representing the narrator Orange’s reflective story-telling with a deeply moving tone.

## Conclusion

This study has identified the existence of translator’s style and found a stable translator’s style in Harman’s translation of Huang’s two children’s literature (RQ 1), that is low lexical density, the choice of contracted forms, habitual use of collocations (such as the collocation of eye) and a strategic approach to retain or domesticate four-character Chinese idioms, the representation of onomatopoeia and flexible treatment of culturally-loaded terms, slang and dialects driven by translator’s agency at the lexical level. In addition, Harman’s translator’s style is characterized by her lower average sentence length and concise sentence structures realized by sentence segmentation at the syntactic level, readability suitable for young readers at the textual level. These findings suggest that several factors contribute to the shaping of Harman’s translator’s style (RQ2) in her English translations of the two children’s literature, including the relatively marginal position of translated works by non-Western authors within the target literary system, multimodal and paratextual cues, and Harman’s stated translation ethics or philosophy (e.g., dual responsibility and an effort to balance domestication with foreignization, etc.).

A corpus-based study has provided a feasible way to explore translator’s style for its reasonable explanation of the empirical data generated by corpus tools. This study has been one of these tentative ways to demonstrate the existence of translator’s style, in the translator’s treatment of two children’s literature by the same author, but in a way different from the majority studies with their focuses on the same work translated by different translators. This study has advanced the study of translator’s style by adding more parameters to the examination of lexical, syntactic and textual features of the translated texts, such as translation strategies employed in the translation of four-character Chinese idioms, translation of onomatopoeia and flexible treatment of culturally-loaded terms, slangs and dialects at the lexical level.

In addition, this study has explained the reason of translator’s style from the socio-cultural factors constrained the lower position of translated literature in the target literary system, to translator’s preferences and paratextual materials, such as the multimodal interaction employed by Harman for her special consideration for the targe young readers. Harman’s views on translation have been demonstrated in dual responsibility, a balance between domestication and foreignization, translator’s voice, translator as an active rewriter, and translation as a form of self-expression and a form of authorship. Drawing on her views on translation, Harman adapts her work to the reading habits and expectations of young target readers, with her distinctive style evident in the skillful rendering of the vividness, purity, and joy of the original children’s literature, reflecting her balanced loyalty to both the target audience and the source-text author.

As the study of translator’s style has been diversified in its scopes and ranges, such as the inclusion of multi-dimensional method and the introduction of functional approach to translator’s style [52], more systemic and multi-dimensional exploration of translator’s style will guide the future development of this potential branch in translation studies. As this study does not cover all aspects of translator’s style, it is hoped that future research will make significant progress in providing new and more valuable insights into the study of translator’s style.

Unlike studies comparing multiple translators or diverse authors, this research distinctly investigates one translator’s style across the consecutive translations of a single author. This study provides fresh insights into how to reveal translator’s style for one translator in translating two children’s literature by the same author, which is an area that has been scarcely discussed in corpus-based translation studies. Future studies can incorporate more children’s stories translated by the same translator to broaden the research scope and compare English translations against non-translated English texts to distinguish between individual translator idiosyncrasies and universal tendencies inherent in translating for younger readers.

## Supporting information

S1 Fig Readability result_ *I Want to Be Good.* (TIFF)

S2 Fig Readability result_ *Flight of the Bumblebee.* (TIFF)

S3 Fig Statistics list generated by WordSmith 8.0. (TIFF)

S1 File *I Want to Be Good* – WordSmith 8.0_Word list. (XLSX)

S2 File *I Want to Be Good* – WordSmith 8.0_Key word list. (XLSX)

S3 File *Flight of the Bumblebee* – WordSmith 8.0_Word list. (XLSX)

S4 File *Flight of the Bumblebee* – WordSmith 8.0_Key word list. (XLSX)

S5 File Four-character idioms in *Wo Yao Zuo Hao Hai Zi.* (PDF)

S6 File Four-character idioms in *Ye Feng Fei Wu.* (PDF)

S7 File Data. (ZIP)
