# Supplementary figures and images for: Exploring translator’s style in children’s literature: A case study of Nicky Harman’s English translations of Huang Beijia’s two works
Source: PLoS One. 2026 Jun 2;21(6):e0350245. doi: 10.1371/journal.pone.0350245 (PMC13229296; doi:10.1371/journal.pone.0350245)

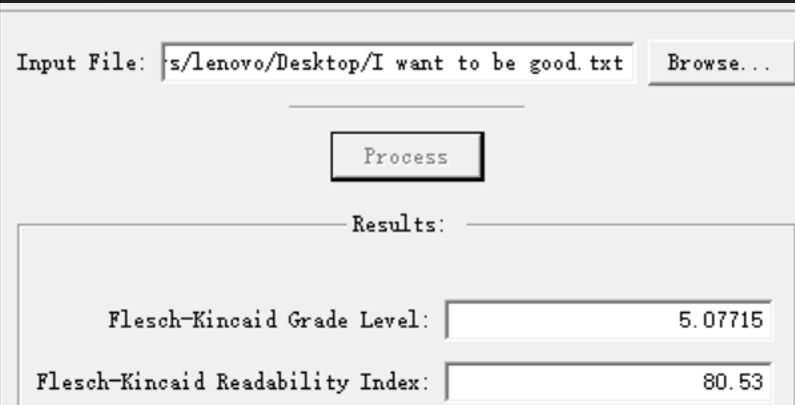

Supplement: S1 Fig — (TIFF) [file pone.0350245.s001.tiff]

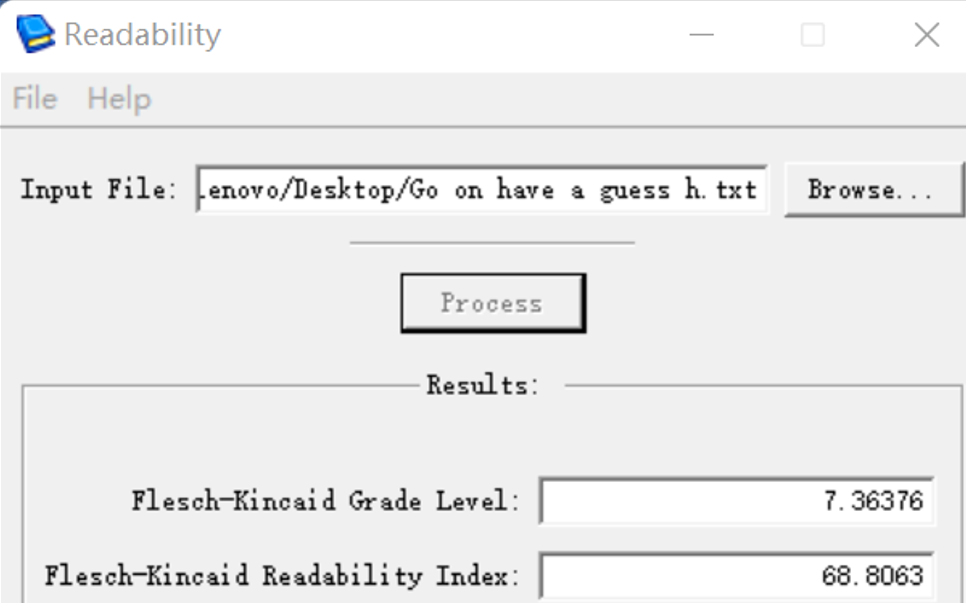

Supplement: S2 Fig — (TIFF) [file pone.0350245.s002.tiff]

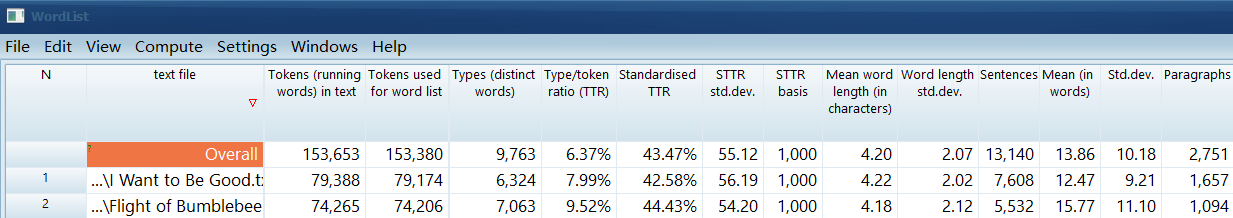

Supplement: S3 Fig — (TIFF) [file pone.0350245.s003.tiff]
