# Supplementary material for: Exploring translator’s style in children’s literature: A case study of Nicky Harman’s English translations of Huang Beijia’s two works
Source: PLoS One. 2026 Jun 2;21(6):e0350245. doi: 10.1371/journal.pone.0350245 (PMC13229296; doi:10.1371/journal.pone.0350245)
Supplement: S5 File — (PDF) [file pone.0350245.s008.pdf]

| | | | | | |
|---|---|---|---|---|---|
| 1 | ，满 肚子 的 气 都 消 得 | 干干净净 。 | 第六章中文.txt | 10 | 74 |
| 2 | 轻度 弱智 的 证明 。 老师 说 ： 李林 ， | What ’s your name ? | 第二十二章中文.txt | 1 | 44 |
| 3 | 幸幸 ， 这个 五六 岁 的 小 女孩 | 面色苍白 ， 一 双 眼睛 像 受惊 的 兔子 | 第九章中文.txt | 9 | 8 |
| 4 | 是 金铃 这 回 能 理解 了 。 | 金亦鸣松 一 口 气 ， 对 赵卉紫 抱 拳 | 第六章中文.txt | 10 | 71 |
| 5 | 致命 弱点 ， 叮嘱 他们 一定 不 要 | 掉以轻心 。 一 堂 普普通通 的 数学 课 | 第十八章中文.txt | 12 | 24 |
| 6 | ，未免 | 心生烦躁 。 一 烦躁 就要 朝 金铃 瞪眼 ， | 第十七章中文.txt | 16 | 40 |
| 7 | 凄厉 ， 金铃 心里 被 它 搅和 得 | 七上八下 ， 一 道 四则 混合 计算题 前后 | 第十四章中文.txt | 18 | 39 |
| 8 | 她 用劲 往 下 一 拉 。 金铃 | 猝不及防 ， 一下子 滑 落 在 地 ， 摔 了 | 第二十二章中文.txt | 1 | 23 |
| 9 | 样子 ， 慢吞吞 上前 帮忙 。 两 个 人 | 围追堵截 ， 一个 按 猫头 ， 一个 按 猫屁 | 第十四章中文.txt | 18 | 36 |
| 10 | 妈妈 为 一 只 猫 忙 得 | 团团直转 ， 一个 煮 猫鱼 替 它 拌饭 ， | 第十四章中文.txt | 18 | 41 |
| 11 | 铅笔盒 在 她 背 上 跳 得 | 咣啷咣啷 一个劲 响 。 金铃 先 把 喜讯 | 第六章中文.txt | 10 | 13 |
| 12 | 已经 | 烟消云散 ， 一切 从 现实 出发 。” 余 | 第二十四章中文.txt | 4 | 5 |
| 13 | 诺贝尔 的 钱 这么 被 人 看重 ？ | 说来说去 一句话 ， 世界 上 最 可 崇尚 的 | 第十六章中文.txt | 15 | 37 |
| 14 | 幕墙 错 当做 你 美丽 的 家园 ， | 糊里糊涂 一头 撞 上 去 ， 才 使得 你 | 第十九章中文.txt | 14 | 58 |
| 15 | 回家 去 精心 做 这 篇 文章 ， | 明天早上 一定 要 交 来 ， 不然 就 赶 | 第二十章中文.txt | 7 | 33 |
| 16 | ， | 东张西望 。 一旁 的 家长 背着 水壶 ， | 第二十六章中文.txt | 2 | 22 |
| 17 | 就是 把 路上 的 石子 当 皮球 | 踢来踢去 。 一旁 的 老师 上 一 天 课 | 第二十四章中文.txt | 4 | 27 |
| 18 | 纸 上 的 两 个 数字 ， 还是 | 糊里糊涂 。 一旁 观战 的 赵卉紫 急 了 | 第六章中文.txt | 10 | 68 |
| 19 | ，我 可 没 这个 意思 。” 卉紫 见 | 话不投机 ， 一时 也 不 知道 如何 是 好 | 第十三章中文.txt | 17 | 22 |
| 20 | “上山” 之前 的 生长 速度 几乎 是 | 突飞猛进 ， 一条条 变 得 长 而 透明 ， | 第二十三章中文.txt | 3 | 9 |
| 21 | 课 像 是 一 台 演出 的 | 压轴节目 一样 ， 总是 要 到 最后 才 出场 | 第十三章中文.txt | 17 | 3 |
| 22 | 就 行 。” 卉紫 扳 着 指头 说 ： “ | 一日千里 ’ 、 ‘ 一毛不拔 ’ 、 ‘ 一枕 | 第二十三章中文.txt | 3 | 23 |
| 23 | 墙 。 英语 成绩 也 只 能 说是 马马虎虎 ， | 一般 九十 来 分 。 比如 说 | 第1章中文.txt | 23 | 14 |
| 24 | 说 出 几 个 不 一样 的 ？” “ ‘ | 一鸣惊人 ’ ！ ‘ 一败涂地 ’ ！ ” 卉 | 第二十三章中文.txt | 3 | 28 |
| 25 | ’ 、 ‘ 一毛不拔 ’ 、 ‘ 一枕黄粱 ’ 、 ‘ 一诺千金 ’ 、 ‘ | 一钱不值 ’ 、 ‘ 一鼓作气 ’ 、 ‘ 一曝 | 第二十三章中文.txt | 3 | 24 |
| 26 | 数 。” 馨兰 伸 出 四 根 指头 。 “ | 4000 ？” “4万 ！ ” 卉紫 下 | 第二十章中文.txt | 7 | 39 |
| 27 | 考试 ， 体会 最 深 ， 写 出来 不会 | 浮皮潦草 。” 三说两说 ， 卉紫 倒 被 | 第二十章中文.txt | 7 | 31 |
| 28 | 条 大鱼 分解 成 块 。 墙壁 上 、 | 抽水马桶 上 ， 到处 都 是 喷溅 上去 的 | 第十五章中文.txt | 19 | 37 |
| 29 | 的 感谢 表现 在 对 金铃 的 | 百依百顺 上 。 这 使得 卉紫 越发 对 幸 | 第九章中文.txt | 9 | 40 |
| 30 | 她 懂事 吧 ， 她 又 不 想 | 争先要强 。 上课 也 是 这样 ， 人 坐 在 | 第4章中文.txt | 25 | 37 |
| 31 | 说 ： “我 以为 你 不 会 同意 ……” “ | 一般情况 下 我 当然 不 会 同意 。 可是 | 第二十二章中文.txt | 1 | 36 |
| 32 | 的 担忧 ， 使 她 不 能 不 | 撕毁前约 ， 下决心 侦察 出 金铃 的 “秘 | 第二十四章中文.txt | 4 | 20 |

| | | | | | |
|---|---|---|---|---|---|
| 33 | 态度， 认真 对待 每 一 次 作业， | 踏踏实实 下苦功夫。 金铃 绞 着 一 双 | 第二章中文.txt | 8 | 17 |
| 34 | 猛 喝 了 一 肚子 凉 开水， | 假戏真做。 不 到 半 小时， 她 真 要 | 第二十二章中文.txt | 1 | 8 |
| 35 | 打断 卉紫 的 话： “ 谁 说 我 | 外孙女儿 不 如 人家？ 学习， 学习！ | 第十四章中文.txt | 18 | 4 |
| 36 | 一个 带 雨篷 的 报摊 做 掩护， | 瞪大眼睛 不 敢 有 丝毫疏忽。 片刻， | 第二十四章中文.txt | 4 | 33 |
| 37 | 。” 于胖儿 摇摇 头： “ 我 的 学校 跟 | 希望工程 不 是 一 回事， 我 是 专门 | 第二十五章中文.txt | 5 | 35 |
| 38 | 被 开除 出 补习班。 我 要 是 | 不识时务， 不 是 白白 撞 在 她 枪口 | 第五章中文.txt | 22 | 15 |
| 39 | ， | 大大咧咧， 不 是 这儿 错 了 节拍， 便 | 第十七章中文.txt | 16 | 44 |
| 40 | 是 这样， 那 孩子 一定 跟 “ 机器人 ” | 相差无几， 不 是 金铃 这样 心地 善良、 | 第十三章中文.txt | 17 | 61 |
| 41 | 担心 呢？ 十 来 岁 的 孩子， | 跑跑跳跳 不 正是 又 好玩 又 不 费劲 | 第十三章中文.txt | 17 | 10 |
| 42 | ， 我 可以 背 妈妈 去 医院， 可以 | 几天几夜 不 睡觉 照顾 妈妈。 如果 我 | 第八章中文.txt | 0 | 8 |
| 43 | 去。 一 草坪 的 同学 都 面面相觑， | 莫名其妙， 不 知道 发生 了 什么 事。 | 第二十五章中文.txt | 5 | 56 |
| 44 | 的 麻烦事， 一时间 倒 比 学生 更 | 手足无措， 不 知道 如何 是 好。 幸好 | 第十三章中文.txt | 17 | 19 |
| 45 | 间 要 面对 婴儿 湿疹 的 治疗， | 手足无措， 不 知道 怎么 下药。 好 在 | 第六章中文.txt | 10 | 61 |
| 46 | 一样 皱 着 眉、 吸 着 嘴， | 迟迟疑疑 不 能 决定。 金铃 换 | 第五章中文.txt | 22 | 16 |
| 47 | 。 金铃 刚 想 伸手 去 摸， 卉紫 | 大喝一声： “ 不 能 动！ ” 吓 得 | 第十四章中文.txt | 18 | 26 |
| 48 | 之后 会 因为 金铃 的 分数 而 | 辗转反侧 不 能 成 眠， 而 后 就 | 第十二章中文.txt | 13 | 28 |
| 49 | 尚海 的 做法 有 点 可耻， 像 | 趁火打劫， 不 那么 光明正大。 别人 上 | 第十九章中文.txt | 14 | 15 |
| 50 | “ 狮子头 ” 下 肚。 奶奶 家 的 情况 | 大同小异。 不同 之 处 在于 奶奶 家 吃 | 第十二章中文.txt | 13 | 8 |
| 51 | 和 卉紫 双双 地 跟 在 后面 | 跑来跑去， 不断 地 贡献 自己 的 意见， | 第十七章中文.txt | 16 | 66 |
| 52 | 来。 金亦鸣 四下 看看， 说： “ 怎么 了？ | 如临大敌？ ” 不等 卉紫 答话， 又 说 | 第十三章中文.txt | 17 | 45 |
| 53 | 来， 随时 准备 奔 上去 助 金铃 | 一臂之力。 不苟言笑 的 杨主任 此时 再 | 第十八章中文.txt | 12 | 65 |
| 54 | 当 了 副班长。 可是 又 因为 过于 | 尖酸刻薄， 与 人 说话 总 带 着 三分 | 第十一章中文.txt | 20 | 14 |
| 55 | 着 装有 文具 和 准考证 的 塑料袋， | 轻轻松松， 东张西望。 一旁 的 家长 背 | 第二十六章中文.txt | 2 | 21 |
| 56 | 吗？ ” “ 可 你 总是 这么 一 副 | 漫不经心、 丢三落四 的 样子， 不 是 算 | 第十八章中文.txt | 12 | 33 |
| 57 | 猛然 又 憋 出 两 个： “ ‘ 一字千金 ’ 、 ‘ | 一饭千金 ’ 。 ” “ 3 个 ‘ 千金 ’ | 第二十三章中文.txt | 3 | 27 |
| 58 | 的 围住 了 那些 买 到 的， | 七嘴八舌， 个个 都 很 兴奋。 金铃 问 | 第二十一章中文.txt | 6 | 8 |
| 59 | 的 大鱼 寒假 就 这么 一天天 在 | 书山题海 中 无聊 地 过去。 偶尔 金铃 | 第十五章中文.txt | 19 | 1 |
| 60 | ？ 说明 金铃 的 各科 成绩 都 是 | 不好不坏， 中不溜儿。 如果 按照 邢老师 | 第二十章中文.txt | 7 | 10 |
| 61 | 金字塔 塔尖。 以下 依次 是 育才 中学、 | 第四十九 中学、 新华 街 中学。 据 单位 | 第4章中文.txt | 25 | 4 |
| 62 | 飞快 地 下降， 行为 散漫 随便， 为人 | 不拘小节。 举 金铃 刚 上 小学 时 的 | 第1章中文.txt | 23 | 20 |
| 63 | 不 住 这样 丢 三 落 四。 | 千钧一发 之际， 金铃 蓦地 一 声 尖叫： | 第1章中文.txt | 23 | 25 |
| 64 | 打分 时 也 尽量 公允客观， 既 不 | 徇私留情， 也 不 图谋泄愤。 比如 她 | 第十九章中文.txt | 14 | 49 |

| | | | | | |
|---|---|---|---|---|---|
| 65 | 的 发型， 弄 得 两 个 打工妹 | 一时一刻 也 停 不 下 手中 的 电吹风 。 | 第九章中文.txt | 9 | 2 |
| 66 | 这 是 “ 功到自然成 ” ， 金铃 这些 年 一直 | 屈居人后， 也 该 有 个 “ 一鸣惊人 ” | 第二十三章中文.txt | 3 | 20 |
| 67 | 大 决心 要 弄清 情况 的， 所以 | 一急之下 也 顾不得 会 不 会 让 金铃 | 第二十四章中文.txt | 4 | 36 |
| 68 | 。 金铃 站 在 水池 边 对 爸爸 | 指手画脚， 书包 还 背 在 肩上， 看样子 | 第二十三章中文.txt | 3 | 42 |
| 69 | ， 有 建议 给 李林 家里 打电话 的， | 七嘴八舌 乱哄哄 一片 。 体育 老师 自己 | 第十三章中文.txt | 17 | 18 |
| 70 | 群 可 没有 刚才 的 孩子 那么 | 守规守矩 了， 一个 个 脚 底下 安 了 | 第二十四章中文.txt | 4 | 25 |
| 71 | 金铃 这样， 倒 真 的 不 敢 | 调皮捣蛋 了， 一个 个 构思 的 构思， | 第十九章中文.txt | 14 | 41 |
| 72 | 可以 出 校门 。” 现在 是 全班 同学 | 哈哈大笑 了， 东倒西歪 的， 前仰后合 的 | 第二十五章中文.txt | 5 | 36 |
| 73 | 不 想 管 你 的 女儿 了， | 无能为力 了， 以后 的 事情 请 你 来 | 第二十三章中文.txt | 3 | 4 |
| 74 | 的 考试 | 接踵而至 了。 先 是 考 各门 副科： | 第十三章中文.txt | 17 | 2 |
| 75 | 、 如 海浪 。 这样 的 想像 实在 太 | 令人鼓舞 了， 卉紫 于是 告诫 女儿 说： | 第十七章中文.txt | 16 | 35 |
| 76 | 孩子 一个 人 躺 在 床 上 | 哈哈大笑 了 半天， 原来 想 的 是 这样 | 第十八章中文.txt | 12 | 32 |
| 77 | 地 说： “ 妈妈 说话 | 自相矛盾 了 吧？ 你 不 是 经常 对 | 第八章中文.txt | 0 | 21 |
| 78 | 个 错别字 一 扣， 分数 也 就 | 可想而知 了。 大致 说 起来， 语文 成 | 第1章中文.txt | 23 | 10 |
| 79 | ： “ 天 哪！ ” 她 觉得 这 不可思议， 太 | 不可思议 了！ 她 偷 桑叶 偷 到 了 | 第二十二章中文.txt | 1 | 40 |
| 80 | ， 就 与 你 期盼 中 的 100 分 | 失之交臂 了。 她 心里 想， 要求 孩子 | 第二十三章中文.txt | 3 | 31 |
| 81 | 翻 着 这些 习题 材料 。 她 已经 | 见怪不惊 了。 她 心里 想： 没有 这些 | 第十四章中文.txt | 18 | 8 |
| 82 | 罢了。” 第二 天， 卉紫 打电话 给 馨兰， | 婉言谢绝 了 她 的 好意 。 馨兰 在 电话 | 第二十章中文.txt | 7 | 47 |
| 83 | 这么 一 行 字： “ 解放 了， 人民 | 当家做主 了。” 她 脑子 里 灵光一闪， | 第十章中文.txt | 21 | 11 |
| 84 | 。 可是 下课 之后 尚海 却 对 她 | 不依不饶 了。 尚海 说： “ 刚才 我 | 第二十一章中文.txt | 6 | 31 |
| 85 | 呢？ ” 金铃 立刻 追问 一 句。 金亦鸣 | 无话可说 了， 想 了 想， 嘀咕 一 句 | 第十八章中文.txt | 12 | 16 |
| 86 | 等 金铃， 说： “ 你 真 是 聪明一世 | 糊涂一时 了， 想想 看， 夏天 就 要 考 | 第十七章中文.txt | 16 | 50 |
| 87 | 的 蚕 太 饿 了， 它们 已经 | 奄奄一息 了！” 老太太 笑 起来： “ | 第二十二章中文.txt | 1 | 38 |
| 88 | 特别 亢奋， 再 不 像 小时候 那么 | 挑三拣四 了， 而是 就近 找 地方 下口， | 第二十三章中文.txt | 3 | 10 |
| 89 | 你 存心 抗拒 邢老师 的 命令？ ” 倪志伟 | 无话可说 了， 身子 矮 了 下来， 嘴里 | 第十九章中文.txt | 14 | 36 |
| 90 | 就 大 叫： “ 老天爷！ 孩子 吃 什么 | 山珍海味 了？ 还 不 是 平常 人家 吃 | 第十二章中文.txt | 13 | 11 |
| 91 | 人， 100 多 名 家长， 排 下来 应该 | 绰绰有余 了。 邢老师 在 班 里 统计 能 | 第十八章中文.txt | 12 | 14 |
| 92 | 有 权威 的 奶奶 和 外婆 。 卉紫 | 无话可说 了。 金铃 其实 说 得 很 对 | 第八章中文.txt | 0 | 11 |
| 93 | 了 这 一 册 试卷， 那 就 “ | 心中有数 ” 了。错 的 改 成 对 的 | 第二十六章中文.txt | 2 | 3 |
| 94 | 钞票 扔 出去 的 时候， 恨 得 | 咬牙切齿 。 于 胖 儿 趴 在 座位 | 第五章中文.txt | 22 | 12 |

95 说 的 这些 稚嫩 偏激 的 言语 后悔不已 。 于是 她 摆摆 手 说 ： “ 第二十五章中文.txt 5 40
96 的 名字 。 全班 同学 都 笑 得 死去活来 。 于胖儿 当时 正 偷 吃 饼干 第二十二章中文.txt 1 46
97 ！ ” 这 一 招 很 灵 ， 尚海 当即 举手投降 ， 交待 了 他 正在 做 的 事情 第六章中文.txt 10 34
98 ， 走路 浑身 都 动 ， 脑袋 不住 地 转前转后 交换 有趣 的 新闻 ， 再 就是 把 第二十四章中文.txt 4 26
99 精致 ， 看上去 又 很 结实 耐用 ， 是 中外合资 产品 。 卉紫 一 问 价 ， 是 第十七章中文.txt 16 7
100 ， 应有尽有 。 人 其实 很 奇怪 ， 闲暇 的 第八章中文.txt 0 15
101 写 了 几 个 大大 的 字 ： “ 说话算话 ！ ” 人 没 进 门 ， 先 把 第二十四章中文.txt 4 13
102 办公室 里 改 作文本 的 ， 忽然 间 天旋地转 ， 人 跟着 咕咚 一 声 跌倒 在 第十九章中文.txt 14 1
103 着 女儿 睡熟 了 ， 直 睡 得 天昏地暗 人事 不知 。 赵卉紫 送 完 稿件 第3章中文.txt 24 7
104 一 伸 说 ： “ 两 毛 钱 。 ” 卉紫 莫名其妙 ： “ 什么 两 毛 钱 ？ ” “ 第二十四章中文.txt 4 40
105 和 张灵灵 两 个 人 笑 得 前仰后合 。 “ 什么 发霉 呀 ！ ” 李小 第二十一章中文.txt 6 10
106 秒 钟 的 时间 ， 三 个 女孩子 面面相觑 ， 什么 话 都 说 不 出来 。 第二十一章中文.txt 6 15
107 大 可信 ， 吹牛 的 成分 多 ， 一旦 事到临头 ， 仍然 会 心甘情愿 跟着 广告 走 第八章中文.txt 0 16
108 别人 都 要 听见 了 。 然后 她 遮遮掩掩 从 书包 里 拿 出 数学 卷子 。 第六章中文.txt 10 39
109 是 臊 的 还是 晒 的 。 邢老师 当机立断 ， 从 口袋 里 掏 出 50 块 钱 第二十五章中文.txt 5 45
110 话题 ， 时不时 还 把 对方 逗 得 哈哈大笑 。 从 她 的 学校 到 家 ， 一 第1章中文.txt 23 3
111 ！ 她 赌气 狠狠 地 擦 去 眼泪 ， 抬头挺胸 从 妈妈 身边 走 过去 ， 脚步 跺 第二十一章中文.txt 6 45
112 被 泼 了 一 瓢 冷水 ， 顿时 脸色大变 ， 从 心里 往 外 地 凉 。 她 第二十章中文.txt 7 43
113 坏 的 呀 ！ 再 一 想 又 恍然大悟 ： 从前 发 书 只有 语文 、 数学 第十七章中文.txt 16 5
114 从前 那样 说 一些 场面 上 的 豪言壮语 。 ” 从来不 举手 发言 的 李林 第二十五章中文.txt 5 19
115 “ 老板 ” 、 右 一 声 “ 老板 ” 的 献殷勤 。 今年春节 ， 他 从 深圳 回来 ， 从 机场 第十六章中文.txt 15
7
116 里 东游西荡 。 他 对 于胖儿 说 ， 这 一个 第十三章中文.txt 17 58
117 闹 得 惊天动地 ， 他 老先生 稳 坐 书房 戴 着 第六章中文.txt 10 56
118 妙语连珠 ， 令 老师 击掌 赞叹 。 遗憾 第1章中文.txt 23 8
119 女儿 这个 问题 。 卉紫 想 ， 等 金铃 长大成人 以后 ， 有 过 太 多 的 梦想 、 第十八章中文.txt 12 36
120 菜 ， 表示 爸爸 妈妈 在 跟 金铃 同甘共苦 。 以后 的 10 多 天 里 ， 第八章中文.txt 0 31
121 的 手 骄傲 地 在 商场 里 走来走去 仿佛 要 展示 一个 漂亮 的 小 第九章中文.txt 9 39
122 ， 把 嘴唇 闭 得 紧紧 的 ， 生怕 一不留神 会 嚎啕大哭 。 第二 天 她 提 第六章中文.txt 10 7
123 敞 着 厕所 的 门 ， 让 小便声 哗啦哗啦 传出 好 远 。 可是 妈妈 又 不 第二十二章中文.txt 1 9
124 有 了 老鼠 ？ ” 卉紫 一下子 很 慌张 ， 如临大敌 似的 ， 把 厨房 里 瓶瓶罐罐 都 第十四章中文.txt 18 12
125 都 准备 睡觉 了 ， 金铃 母女 一下子 从天而降 似的 ， 把 她 惊喜 得 连声 叫唤 第二十五章中文.txt 5 75
126 受 拘束 ， 对 外婆 的 态度 是 敬而畏之 。 但是 外婆 有 一个 观点 是 第十二章中文.txt 13 13
127 在 电视 上 看到 了 。 ” 金铃 有点 不好意思 ， 低头 用 脚尖 蹭 着 地面 ， 第十七章中文.txt 16 56

| | | | | | |
|---|---|---|---|---|---|
| 128 | 零碎 鱼块， 再 送 出 一部分 给 | 亲朋好友， 余下 的 自家 慢慢 消化。 | 第十五章中文.txt | 19 | 28 |
| 129 | 不 服气： “那 我们 就 不 跟 | 坏人坏事 作 斗争 了 吗？” 卉紫 说 | 第十三章中文.txt | 17 | 44 |
| 130 | 要 向 英雄 李平乐 学习， 坚决 跟 | 坏人坏事 作 斗争。 金铃 的 日记 邢老师 | 第十一章中文.txt | 20 | 35 |
| 131 | 天 庆祝 建店 40 周年， 在 举办 一个 | 中小学生 作文 大赛， 叫 你 的 孩子 也 | 第十七章中文.txt | 16 | 10 |
| 132 | 的 “征文 启事”， 说 的 正是 举办 | 中小学生 作文 大赛 的 事， 内容 限于 跟 | 第十七章中文.txt | 16 | 12 |
| 133 | 补充 的 题目 上， 她 总是 能 | 出奇制胜。 作文 比较 难 说， 碰到 对 | 第1章中文.txt | 23 | 6 |
| 134 | 的 呢？” 馨兰 叫 着： “咦 呀， | 前些日子 你 不 是 还 赌咒 发誓 的…… | 第二十四章中文.txt | 4 | 3 |
| 135 | 之后 就 紧闭 嘴巴， 挺 着 脖子， | 一言不发。 “你 不 说？ 不 说 我 | 第二十二章中文.txt | 1 | 31 |
| 136 | 又 开小差！ 到 这个 时候 你 还 | 心不在焉？ 你 到底 想 拿 个 什么 分数 | 第十八章中文.txt | 12 | 22 |
| 137 | 不 行 的 话 只好 放弃。” 赵卉紫 | 哭笑不得： “你 是 真 迂 还是 假 迂 | 第六章中文.txt | 10 | 58 |
| 138 | 的 优美 爱情诗， 怎么 可以 跟 色情 | 混为一谈？ 你 简直 不 懂 欣赏。” | 第十七章中文.txt | 16 | 73 |
| 139 | 事 把 整个 家里 都 闹 得 | 人心惶惶， 你 能 指望 孩子 跳 到 局面 | 第十三章中文.txt | 17 | 59 |
| 140 | 把 事情 视为 一个 秘密， 对 我们 | 守口如瓶， 你 还是 遵从 孩子 的 意愿 为 | 第二十四章中文.txt | 4 | 46 |
| 141 | 身边 嫩生生 的 菜叶 简直 就 是 | 视而不见， 依旧 可怜巴巴 地 把 脑袋 抬 | 第二十二章中文.txt | 1 | 18 |
| 142 | 自己 的 学校 落后。 一旦 落后， 自己 | 脸上无光 倒 是 小事， 关键 是 明年 的 | 第二十章中文.txt | 7 | 2 |
| 143 | 、 见义勇为、 发奋图强 的 事迹 他们 | 视而不见， 倒 注意 上 了 大款 们 给 | 第十章中文.txt | 21 | 2 |
| 144 | ， 要 过 得 有 意义， 令 自己 | 一生难忘……” 倪志伟 在 下面 大声 | 第二十五章中文.txt | 5 | 13 |
| 145 | 很 平静 地 说： “你 这 是 | 自作聪明。” 倪志伟 学 着 电影 上 那 | 第十一章中文.txt | 20 | 27 |
| 146 | ， 奋勇 站 出来 表示 抗议： “倪志伟 你 | 有话快说！” 倪志伟 转 过 头， 阴沉 | 第十一章中文.txt | 20 | 22 |
| 147 | ， 又 比 卉紫 有 经验， 再加 有 | 利斧快刀 做 武器， 事情 马上 变 得 容易 | 第十五章中文.txt | 19 | 34 |
| 148 | 莫名其妙 地 看 着 爸爸 变戏法 一样 | 轻轻松松 做出 这 道 题， 嘴里 不住 地 | 第六章中文.txt | 10 | 65 |
| 149 | 了 金铃 的 闷闷不乐 的 原因， 忍不住 | 哑然失笑， 停 住 脚步 等 了 等 金铃， | 第十七章中文.txt | 16 | 49 |
| 150 | 吗？” 这 回 连 男生 们 也 | 忍俊不禁， 偷偷 把头 埋 下 去 笑 起来 | 第二十五章中文.txt | 5 | 31 |
| 151 | 回来， 金铃 挺 稀罕， 在 厨房 里 | 转来转去， 催 着 妈妈 快 打开 看看。 | 第八章中文.txt | 0 | 23 |
| 152 | 这时候 很 得意， 像 一个 艺术家 表演 | 拿手绝活 儿 前 要 充分 吊 起 观众 | 第十一章中文.txt | 20 | 20 |
| 153 | 。 到手 的 | 1000 元 啪啪 作响 的 票子， 她 小心 | 第十章中文.txt | 21 | 29 |
| 154 | 不 是 自己 原来 的 家， 开始 | 奋力反抗。 先 是 发出 凄厉 无比 的 惨 | 第十四章中文.txt | 18 | 29 |
| 155 | ， 可 | 今天早上 全 忘 了， 真 的 全 忘 | 第十八章中文.txt | 12 | 42 |
| 156 | 他？” 金铃 说： “因为 他 设立 了 | 诺贝尔奖。 全世界 的 人， 谁 不 想 | 第十六章中文.txt | 15 | 33 |
| 157 | 好， 他 的 保留 项目 “清炖 狮子头”、 “ | 糖醋排骨”、 “八宝 鸭”、 “笋干 | 第十二章中文.txt | 13 | 3 |
| 158 | 来 年。 这时候 妈妈 的 决心 跟着 | 烟消云散， 再 不 提 什么 换 冰箱 的 | 第十五章中文.txt | 19 | 6 |
| 159 | 上 去， 才 使得 你 现在 头晕眼花、 | 步履蹒跚？ 再 不然 就 是 被 马路 上 | 第十九章中文.txt | 14 | 59 |

| | | | | | |
|---|---|---|---|---|---|
| 160 | 钱。” 她 对 卉紫 这么 说。 金铃 | 当天傍晚 再 去 时， 果然 就 被 拒之门外 | 第二十四章中文.txt | 4 | 58 |
| 161 | ，转 过 身 去， 在 黑板 上 | 一笔一划 写下 了 今天 的 作文 题目：《 | 第十九章中文.txt | 14 | 40 |
| 162 | 嗅， 然后 说：“是 香。” 卉紫 就 | 趁热打铁：“冲 一 袋 试试？” 金 | 第八章中文.txt | 0 | 24 |
| 163 | 要 上 厕所！ 我 忍不住 了！” 一边 | 慌慌张张 冲 出 门 去。 全班 哄堂大笑 | 第十八章中文.txt | 12 | 9 |
| 164 | 出 一 团团 金色 的 光。 卉紫 | 慌慌张张 冲 进 楼 内， 看见 校长 制作 | 第二十三章中文.txt | 3 | 35 |
| 165 | 能 指望 孩子 跳 到 局面 之外 | 若无其事 准备 功课 吗？ 如果 真 是 这 | 第十三章中文.txt | 17 | 60 |
| 166 | 偶像。 没 事 的 时候， 金铃 就 | 磨磨蹭蹭 凑 到 老师 跟前， 摸摸 头发 啦 | 第二章中文.txt | 8 | 2 |
| 167 | 一阵 乱 忙， 客厅 里、 房间 里 | 来回奔波， 几乎 把 家里 每 一 本 藏书 | 第十七章中文.txt | 16 | 65 |
| 168 | ，手里 抓 着 宝贵 的 20 片 桑叶， | 蹦蹦跳跳 出门。 走 到 门口 她 忽然 又 | 第二十二章中文.txt | 1 | 50 |
| 169 | 要 照顾 到 二 线 员工 的 | 方方面面， 分 到 金亦鸣 手里 就 没有 几 | 第3章中文.txt | 24 | 11 |
| 170 | ，明年 的 这个 时候， 全班 同学 已经 | 各奔东西， 分散 到 各个 中学 去 了， | 第十一章中文.txt | 20 | 2 |
| 171 | ？” 卉紫 苦笑 笑， 就 把 李林 妈妈 | 昨天晚上 到 家里 来 要 钱 的 事 | 第十三章中文.txt | 17 | 36 |
| 172 | 住 的。” 卉紫 被 她 磨 得 | 不好意思， 到底 还 是 花 出 了 那 14 | 第十七章中文.txt | 16 | 11 |
| 173 | 架势， 对 爸爸 妈妈 的 意见 一概 | 不予理睬。 到手 的 1000 元 啪啪 作 | 第十章中文.txt | 21 | 28 |
| 174 | 纪念。” 金亦鸣 甚至 拿 出 一 瓶 | 青岛啤酒， 动员 金铃 也 喝 几 口。 | 第二十五章中文.txt | 5 | 74 |
| 175 | 回来 就 把 金铃 骂 了 个 | 狗血喷头， 勒令 她 以后 再 不 准 把 | 第九章中文.txt | 9 | 14 |
| 176 | 不 想 得 到 这个 奖 呢？ | 物理学家， 化学家， 医生， 诗人， 文 | 第十六章中文.txt | 15 | 34 |
| 177 | 苍白 的 男 孩子 也 笑 得 | 前仰后合。 医生 转头 笑 着 对 赵卉紫 | 第七章中文.txt | 11 | 29 |
| 178 | 也 是 刚刚 到 家。 父女 俩 | 有说有笑 十分 轻松。 卉紫 喝道：“ | 第二十三章中文.txt | 3 | 43 |
| 179 | 一 根 细细的 长 丝 吊 下来， | 悠悠荡荡， 十分 闲适 的 样子。 卉紫 | 第二十三章中文.txt | 3 | 33 |
| 180 | ，闹 得 一 条 街上 | 人心惶惶。 卉紫 下班 回家， 从 外面 | 第九章中文.txt | 9 | 12 |
| 181 | 坐 了 一 大 片， 互相 间 | 交头接耳。 卉紫 东一句西一句 听 着， 说 | 第4章中文.txt | 25 | 16 |
| 182 | 了 卉紫 的 罪过， 弄 得 她 | 长吁短叹。 卉紫 东奔西走 地 托 了 好多 | 第十三章中文.txt | 17 | 12 |
| 183 | 原先 认为 不 好吃， 这会儿 吃 得 | 津津有味。 卉紫 也 不 说 穿， 只 在 | 第十章中文.txt | 21 | 32 |
| 184 | 做 了 傻事， 我 做 了 傻事…… | 那天晚上 卉紫 买 了 一 只 很 肥 | 第八章中文.txt | 0 | 40 |
| 185 | 。” | 三说两说， 卉紫 倒 被 说 得 心 动 | 第二十章中文.txt | 7 | 32 |
| 186 | 。邢老师 很 能 说， 又 是 一番 | 滔滔不绝。 卉紫 发现 当 老师 的 都 那么 | 第4章中文.txt | 25 | 23 |
| 187 | 就 你 来 写 吧。” 余 老太 | 见缝插针。 卉紫 吓 了 一跳：“我 | 第二十章中文.txt | 7 | 30 |
| 188 | 还要 | 神气百倍。 卉紫 在 心里 恨恨 地 想： | 第4章中文.txt | 25 | 22 |
| 189 | 下 了 这个 胖乎乎、 笑眯眯 的 “猪八戒”。 | 当天晚上 卉紫 就 从 电视 新闻 里 看到 | 第十一章中文.txt | 20 | 44 |
| 190 | 没有 立刻 就 走， 围 着 邢老师 | 问这问那。 卉紫 就 静静地 站 在 后面 等 | 第4章中文.txt | 25 | 26 |
| 191 | 欢乐 晚餐， 吃 到 最后 仍然 是 | 乌云压顶。 卉紫 很 恨 自己 对 孩子 分 | 第九章中文.txt | 9 | 37 |
| 192 | 展示 出来。 卉紫 听 得 有 点 | 目瞪口呆。 卉紫 心想 现在 的 孩子 可 真 | 第五章中文.txt | 22 | 22 |

| | | | | | |
|---|---|---|---|---|---|
| 193 | 算术 式子 和 红笔 打 上 的 | 钩钩叉叉 。 卉紫 心里 咯噔 一下 ， 她 | 第二十三章中文.txt | 3 | 3 |
| 194 | 要 付 两 毛 钱 吗 ？ ” 摊主 | 振振有词 。 卉紫 心里 有 事 ， 懒得 多 | 第二十四章中文.txt | 4 | 41 |
| 195 | 不 是 最 差 ， 跟 从前 一样 ， | 中不溜儿 。 卉紫 心里 有 点 失望 ， 又 | 第4章中文.txt | 25 | 25 |
| 196 | 有 人 敲门 。 敲门声 很 响 ， 而且 | 理直气壮 。 卉紫 急忙 从 厨房 里 跑 出 | 第九章中文.txt | 9 | 48 |
| 197 | ， | 对症下药 。 ” 卉紫 慌忙 道谢 ： “ 邢 | 第二十章中文.txt | 7 | 20 |
| 198 | 了 ， 也 没 见 半 个 老鼠洞 。 | 当天晚上 ， 卉紫 把 家里 所有 能 吃 的 | 第十四章中文.txt | 18 | 16 |
| 199 | 快 得 像 只 逃命 的 兔子 。 | 当天晚上 ， 卉紫 是 在 给 金铃 整理 床铺 | 第二十一章中文.txt | 6 | 33 |
| 200 | 完 课 回家 ， 两 个 人 都 | 一声不吭 ， 卉紫 是因为 女儿 成绩 不 理想 | 第十七章中文.txt | 16 | 45 |
| 201 | ’ 、 ‘ 一鼓作气 ’ 、 ‘ 一曝十寒 ’ 、 ‘ | 一箭双雕 ’ … … ” 卉紫 有点 卡壳 了 | 第二十三章中文.txt | 3 | 25 |
| 202 | ’ ！ ‘ 一败涂地 ’ ！ ” 卉紫 笑 起来 ， 承认 | | 第二十三章中文.txt | 3 | 29 |
| 203 | 的 学习 和 考试 也 不好 。 俗话说 “ | 花钱消灾 ” ， 卉紫 自己 也 害怕 人家 胡 | 第十三章中文.txt | 17 | 24 |
| 204 | 。 我 想 最好 是 全部 捐 给 | 希望工程 。 ” 卉紫 说 ： “ 也好 ， | 第二十三章中文.txt | 3 | 18 |
| 205 | 得 鼻尖 冒汗 ， 那 鱼 根本 就 | 若无其事 。 卉紫 说 ： “ 送 鱼 的 人 | 第十五章中文.txt | 19 | 30 |
| 206 | 市里 的 奖 。 一 家 人 都 | 大喜过望 。 卉紫 高兴 地 对 金铃 说 ： | 第十七章中文.txt | 16 | 19 |
| 207 | 不 提 什么 换 冰箱 的 旧话 。 | 那天下午 单位 里 分 的 是 冰冻 带鱼 。 | 第十五章中文.txt | 19 | 7 |
| 208 | 多少 努力 ， 才 能 在 那 路上 | 磕磕绊绊 占据 一个 位置 啊 ！ 卉紫 就 | 第二十六章中文.txt | 2 | 16 |
| 209 | 巷子 走 一 圈 ， 你 会 闻到 | 家家户户 厨房 里 飘 出来 的 都 是 | 第十五章中文.txt | 19 | 9 |
| 210 | 开学 之前 ， 卉紫 带 金铃 到 长江 | 百货公司 去 买 过 一 次 书包 。 金铃 | 第十七章中文.txt | 16 | 1 |
| 211 | 能 跟 你 去 。 ” 尚 海 求情 | 一般地说 ： “ 去 吧 ， 保证 震 你 。 | 第五章中文.txt | 22 | 2 |
| 212 | ， 又 不断 地 有 人 挤出 人堆 | 慌慌张张 去 张罗 什么 。 金铃 知道 电线 | 第九章中文.txt | 9 | 6 |
| 213 | 的 孩子 ， 痛心疾首 地 说 ： “ 高分低能 ！ | 高分低能 ！ ” 又 劝说 卉紫 ： “ 别 | 第十二章中文.txt | 13 | 16 |
| 214 | 了 。 因为 女人 们 总 有 些 | 心高气盛 ， 又 因为 这么 多 年 她 始终 | 第4章中文.txt | 25 | 2 |
| 215 | 哪儿 ？ 这么 早就 有 应酬 啊 ？ ” 馨兰 | 抿嘴笑笑 ， 又 将 下巴 朝 旁边 的 美容 | 第二十章中文.txt | 7 | 34 |
| 216 | 再 也 回答 不 出来 了 ， 开始 | 抓耳挠腮 ， 又 扭头 看 窗外 的 麻雀 打架 | 第七章中文.txt | 11 | 23 |
| 217 | 窥视 整个 过程 。 卉紫 和 金铃 奶奶 | 齐心合力 ， 又 砍 又 剁 ， 终于 将 一 | 第十五章中文.txt | 19 | 36 |
| 218 | 一 步 进 门 的 金亦鸣 ， 她 | 慌慌张张 又 结结巴巴 地 对 他 说 ： “ | 第二十四章中文.txt | 4 | 43 |
| 219 | 家长 联手 ， 把 尚海 批 了 个 | 狗血喷头 ， 又 逼 着 他 立刻 找 英语 | 第十八章中文.txt | 12 | 11 |
| 220 | 招呼 金铃 拿 信 ， 金铃 兴奋 得 | 满脸通红 、 双眼发亮 。 她 连蹦带跳 地 | 第二章中文.txt | 8 | 11 |
| 221 | 夫妻 俩 唯一 的 孩子 ， 金亦鸣 主张 | 任其自然 发展 ， 不必 逼 人 太 甚 。 他 | 第4章中文.txt | 25 | 1 |
| 222 | 只 塑料袋 。 袋 里 的 小 蚕 | 浑然不知 发生 了 何事 ， 依旧 在 努力 地 | 第二十一章中文.txt | 6 | 20 |
| 223 | 要 这么 写 ？ 爸爸 凭 学识 帮助 | 乡办工厂 ， 受 人 尊重 ， 人家 送 一 条 | 第十六章中文.txt | 15 | 20 |
| 224 | 不 对 ？ 那 好 ， 我 先 把 | 3923 24 变成 40 - 1 24 | 第六章中文.txt | 10 | 67 |

225 ：“我 可以 便宜 些 。” 张 灵 灵 一口咬定 只 出 20 块 钱 。 金铃 犹豫 第十三章中文.txt 17 54
226 、二 楼 、三 楼 全部 都 是 寂静无声 ，只 有 四 楼 六 年级 教室 第二十三章中文.txt 3 38
227 了 底 ，弄 得 金亦鸣 和 卉紫 面面相觑 ，只好 从 冰箱 里 找 了 一 第十章中文.txt 21 31
228 狼 外婆 就 是 像 魔 女 ，万般无奈 只好 求助 于 金铃 ，条件 是 送 第五章中文.txt 22 7
229 ？要 怨 还是 怨 你 自己 。” 卉紫 无话可说 ，只能 怨 自己 。 外婆 当 了 第十二章中文.txt 13 12
230 轻轻松松 ，叫 人 气 也 不 是 恨 第1章中文.txt 23 17
231 ，再 不然 就 是 字母 写 得 歪歪扭扭 叫 人 难以 辨认 。 英语 老师 第1章中文.txt 23 15
232 的 竖笛 敲 人家 后脑勺 。 音乐 老师 尖声尖气 叫 起来 ：“不 得 了 ！ 上 第十九章中文.txt 14 8
233 我 一 段 胶带纸 ，行 吗 ？” 尚海 大惊小怪 叫 起来 ：“哇 ！ 你 也 考 第六章中文.txt 10 38
234 。 回家 把 钱 交 给 妈妈 ，卉紫 睁大眼睛 叫 起来 ：“那 表 是 外婆 花 第十三章中文.txt 17 55
235 你 ！ 世上 学习 好 的 孩子 有 千千万万 ，可 世上 只 有 一个 金铃 是 第十四章中文.txt 18 46
236 哭 出来 ：“我 昨晚 还 背 得 滚瓜烂熟 ，可 今天早上 全 忘 了 ，真 第十八章中文.txt 12 41
237 送给 金老师 过年 吃 的 。” 卉紫 仍然 疑惑不解 ：“可 我 不 认识 你们 … … 第十五章中文.txt 19 24
238 滴 上 一 滴 “鸟 屎 ” 。 张老师 两手一摊 ：“可惜 这些 办法 都 被 别人 第二十六章中文.txt 2 5
239 ！” 金铃 觉得 好玩 极了 ，一时间 笑 得 前仰后合 。 可是 笑 着 笑 着 她 脸上 第六章中文.txt 10
35
240 啦 … … 凡是 从 开考 到 收卷 之间 分分秒秒 可能 出现 的 问题 都 设想 到 第二十六章中文.txt 2 2
241 问 了 问 金铃 的 情况 。 卉紫 愁眉苦脸 叹气 ，说 她 正 为 金铃 的 第二十章中文.txt 7 40
242 口 之 家 在 春节 期间 是 无论如何 吃 不 下去 的 。 要 完整 地 第十五章中文.txt 19 27
243 的 时候 两 个 人 也 是 一声不响 ，各 有 各 的 心思 。 金铃 第二十四章中文.txt 4 16
244 要 靠 安眠药 睡觉 。” 老师 们 一个个 唉声叹气 ，各自 回 班 去 做 工作 。 第二十章中文.txt 7 8
245 鸟 在 我 的 枝头 歌唱 就 心醉神迷 。 后来 它 成 了 一 株 灌木 第十七章中文.txt 16 70
246 向 你们 要 医药费 ？ 这 不 是 异想天开 吗 ？” “金铃 爸爸 也 是 第十三章中文.txt 17 37
247 发毛 。 后来 她 就 讲 了 张灵灵 一不小心 吞 下 幼蚕 的 事 ，老太太 笑 第二十二章中文.txt 1 43
248 。 于是 她 摆摆 手 说 ：“班会 到此为止 吧 ，要是 耽误 了 复习 ，校长 第二十五章中文.txt 5 41
249 刚 摘 下 耳机 从 书房 出来 ，糊里糊涂 听 了 半 句 ，插嘴 问 ：“ 第十七章中文.txt 16 22
250 跟 着 他 往 楼上 爬 ，一面 气喘吁吁 告诉 他 ，孙淑云 可不 是 一般 第二十四章中文.txt 4 45
251 了 ，谁 想 到 这个 角色 这么 讨人喜欢 呢 ？ 第十一章中文.txt 20 45
252 ！” 金亦鸣 摊 摊手 ：“谁 让 我 是 一家之主 呢 ？” 卉紫 是 刀子嘴豆腐心 第九章中文.txt 9 22
253 有 了 提高 ，卉紫 怎么 能 不 感激涕零 呢 ？ 卉紫 跟 金亦鸣 商量 ，第二十四章中文.txt 4 53
254 还有 个 一张一弛 呢 ，孩子 苦学 了 一个 学期 ，第十四章中文.txt 18 3
255 心态 不错 。 干吗 要 自己 把 自己 逼上绝路 呢 ？ 普通 中学 就 不 是 人 第二十四章中文.txt 4 6
256 钱 ！” “交 两万三万 还 凑合 ，要 交 十万八万 呢 ？” “砸锅卖铁 ！ 第4章中文.txt 25
14

| | | | | |
|---|---|---|---|---|
| 257 | 出 应用题 来， 坐 在 位子 上 抓耳挠腮 呢， 这么 好 玩 的 戏剧性 场面 | 第十八章中文.txt | 12 | 61 |
| 258 | 的 存货 但 吃 无妨， 还 可以 开源节流， 呼吁 奶奶 和 外婆 多 送 些 | 第十章中文.txt | 21 | 36 |
| 259 | 模样。 卉紫 说： “你 怎么 了？” 金铃 紧咬牙关， 呼呼 地 喘气， 一句话 不 说 | 第十六章中文.txt | 15 | 17 |
| 260 | ，只 穿 了 一 件 印 有 白雪公主 和 七 个 小 矮人 的 白色 | 第二十一章中文.txt | 6 | 1 |
| 261 | ： “你 听 着， 爸爸 妈妈 都 是 工薪阶层， 和 人家 经理 不 一样。 正 | 第九章中文.txt | 9 | 47 |
| 262 | 问题： “老鼠 吓 走 了， 你 买 老鼠夹子 和 老鼠药 干 什么 呢？” 卉 | 第十四章中文.txt | 18 | 38 |
| 263 | 熟悉 的 家。 卉紫 买 回去 的 老鼠夹子 和 老鼠药， 自然 也 没有 使用 | 第十四章中文.txt | 18 | 48 |
| 264 | 笑话 他 的， 有 责骂 他 的， 七嘴八舌 哄 成 一 片。 太阳 热辣辣 地 | 第二十五章中文.txt | 5 | 44 |
| 265 | ，指甲 磨 得 嘎吱嘎吱 响， 听 得 人 牙齿 发酸。 | 第十四章中文.txt | 18 | 31 |
| 266 | 个 随意 惯 了 的 人， 站没站相， 坐没坐相， 哪里 受 过 这样 标准化 的 训 | 第十七章中文.txt | 16 | 62 |
| 267 | 的 眼泪 真 的 出来 了， 肩膀 一耸一耸 哭 得 好 伤心。 卉紫 跟 出来 | 第二十一章中文.txt | 6 | 43 |
| 268 | 已经 烟消云灭， 唯一 的 希望 就 在 女儿 金铃 | 第十二章中文.txt | 13 | 20 |
| 269 | 、 打 扑克， 什么 考试 啊， 竞争 啊， 勾心斗角 啊， 发财 不 发财 啊， 统统 | 第二十五章中文.txt | 5 | 38 |
| 270 | 第一 个 起床， 房门 一 开 就 尖声大叫： “啊 呀！ 不 好 了！ | 第十四章中文.txt | 18 | 17 |
| 271 | 了 一 口 春天 的 空气， 感到 无比惬意。 啊！ 春 呀， 草 呀， 我 | 第4章中文.txt | 25 | 33 |
| 272 | 心中 的 兴奋之情 -- 啊！ 春天 终于 来到 了！ | 第4章中文.txt | 25 | 31 |
| 273 | 啦？ 以前 从来 没 有 讲究 过 穿衣打扮 啊？ 片刻 之后 她 才 想 到 | 第二十五章中文.txt | 5 | 15 |
| 274 | 鸿沟， 只不过 能 跨 过去 的 实在 寥寥无几 啊！ 第二 天 风和日丽， 老天 | 第十八章中文.txt | 12 | 37 |
| 275 | 涌 出来 了， 心里 说， 这么 一个 聪明伶俐 善解人意 的 孩子， 怎么 就 偏偏 | 第七章中文.txt | 11 | 30 |
| 276 | 忙乎， 脸 涨 得 通红， 鼻子 里 呼哧呼哧 喘气， 到 最后 身子 还是 卡 在 | 第八章中文.txt | 0 | 5 |
| 277 | 她 在 下意识 的 吮吸 动作 中 不知不觉 喝 下 一 瓶 稠 奶糊。 3 个 | 第八章中文.txt | 0 | 2 |
| 278 | 自私， 不 愿意 帮助 别人！” 母女 俩 唇枪舌剑， 嗓门 一下子 都 提高 了 八 度 | 第九章中文.txt | 9 | 18 |
| 279 | 地 含糊其辞。 老太太 就 自言自语 嘀咕， 说 些 孩子 放学 太 晚 | 第二十四章中文.txt | 4 | 23 |
| 280 | 办法 都 被 别人 识破 了。 ‘道高一尺， 魔高一丈’ 嘛！ 哪 能 这么 容易 让 你 | 第二十六章中文.txt | 2 | 6 |
| 281 | 终于 耷拉 下 脑袋， 死 了。 你 死不瞑目， 嘴巴 也 微 张 着 不 肯 | 第十九章中文.txt | 14 | 62 |
| 282 | 卷子， 跟 着 那 张 白色 “魔毯” 转来转去， 嘴里 发出 惊叹声、 嬉笑声 和 | 第十八章中文.txt | 12 | 64 |
| 283 | 不 敢 再 想 下去。 等 卉紫 气急败坏 回到 家里 的 时候， 金亦鸣 已经 | 第二十三章中文.txt | 3 | 40 |
| 284 | 十一 小人得志 和 君子报仇 圣诞节 一 过， 接 下来 便是 | 第十一章中文.txt | 20 | 1 |
| 285 | 在 孩子 和 家长 的 掌声 中 徐徐闭幕。 在 主课 考试 尚未 提 上 议 | 第十三章中文.txt | 17 | 5 |
| 286 | 仿照 天安门 广场 上 的” 香港 回归日 倒计时钟 “， 在 教学楼 一 楼 的 楼梯口 | 第十八章中文.txt | 12 | 2 |
| 287 | 出差 去 了。 苏北 的 一 家 乡镇企业 在 生产 配料 的 什么 问题 上 | 第十五章中文.txt | 19 | 2 |
| 288 | 又 委屈。 她 想 着 不 能 随随便便 在 陌生人 面前 哭， 要 忍 住 | 第二十二章中文.txt | 1 | 34 |

| | | | | | |
|---|---|---|---|---|---|
| 289 | 更 亮 ， 数 不 清 的 汽车 | 首尾相接 ， 在 马路 上 开 成 了 一 | 第二十五章中文.txt | 5 | 76 |
| 290 | 复印 试卷 的 家长 时 ， 金铃 也 | 不甘落后 地 举 了 手 。 回到 家里 跟 | 第十八章中文.txt | 12 | 15 |
| 291 | 又 找到 了 另 一 本 诗集 ， | 欢天喜地 地 举 在 手里 ： “ 听 着 听 | 第十七章中文.txt | 16 | 67 |
| 292 | 堆 新华街 小学 的 学生 ， 不断 有人 | 满头大汗 地 从 人堆 中 钻 出来 ， 手里 | 第二十一章中文.txt | 6 | 4 |
| 293 | 起 了 疑心 ， 跟踪 而 去 。 金铃 | 装模作样 地 从 厕所 出来 ， 头 一 抬 ， | 第二十二章中文.txt | 1 | 4 |
| 294 | 见 妈妈 脸色 严峻 ， 不 敢 违抗 ， | 磨磨蹭蹭 地 从 厨房 里 走 出来 问 ： “ | 第二十三章中文.txt | 3 | 44 |
| 295 | 装作 不 知道 孩子 们 的 心思 ， | 若无其事 地 从 柜台 旁边 绕 过去 ， 目光 | 第九章中文.txt | 9 | 43 |
| 296 | 还 睡 得 香 呢 。 我 总是 | 没完没了 地 做 作业 ， 连 星期天 也 是 | 第二十五章中文.txt | 5 | 25 |
| 297 | 之类 ， 不 就 使 很多 孩子 可以 | 高枕无忧 地 偷看 电视 节目 了 吗 ？ 当 | 第十五章中文.txt | 19 | 3 |
| 298 | 尾巴 都 翘 在 半空 里 。 卉紫 | 惊慌失措 地 先 跟 着 他们 进 了 | 第十五章中文.txt | 19 | 22 |
| 299 | 之后 ， 他 才 一边 趿拉 着 旅游鞋 | 满头大汗 地 冲 进 教室 ， 一边 申明 ： | 第十九章中文.txt | 14 | 5 |
| 300 | ， 又 转身 奔 向 一旁 的 卫生间 ， | 当机立断 地 卸 了 担子 ， 把 那 鱼 | 第十五章中文.txt | 19 | 20 |
| 301 | ， 放 着 自己 的 卷子 不 管 ， | 不屈不挠 地 去 追 金铃 的 那 张 。 | 第十八章中文.txt | 12 | 63 |
| 302 | 不 怎么 可爱 的 分数 上 。 金铃 | 受宠若惊 地 反手 抱 住 了 妈妈 ， 一边 | 第六章中文.txt | 10 | 6 |
| 303 | 才 是 孩子 学习 的 动力 ？ ” 大学生 | 理直气壮 地 反问 ： “ 不 是 这样 吗 ？ | 第十章中文.txt | 21 | 5 |
| 304 | 她 睡 得 憨态 十足 ， 嘴角 还 | 一牵一牵 地 发 笑 ， 大约 正 做 着 | 第二十章中文.txt | 7 | 24 |
| 305 | 什么 。 一 见 金铃 进 门 ， 他 | 笑容满面 地 叫 起来 ： “ 啊 ， 儿童节 | 第二十五章中文.txt | 5 | 73 |
| 306 | 来 两 个 ， 怎么 也 不 肯 | 痛痛快快 地 吐 一 次 。 卉紫 把 自己 | 第二十四章中文.txt | 4 | 29 |
| 307 | 把 屈辱 吃 进 肚子 里 ， 再 | 原封不动 地 吐 出 来 ， 该 是 谁 | 第五章中文.txt | 22 | 24 |
| 308 | 的 。 金铃 一 分钟 也 不 耽搁 ， | 理直气壮 地 向 张 灵 灵 讨 回 | 第五章中文.txt | 22 | 20 |
| 309 | ？ 家里 没有 老人 帮忙 照应 吗 ？ ” 卉紫 | 嗯嗯啊啊 地 含糊其辞 。 老太太 就 自言自语 | 第二十四章中文.txt | 4 | 22 |
| 310 | 英语 老师 年轻 ， 听力 特别 好 ， 竟 | 准确无误 地 听 见 了 这 句 近似 | 第十八章中文.txt | 12 | 3 |
| 311 | 大家 做 。 金铃 做 完 了 回家 ， | 眉飞色舞 地 告诉 卉紫 得了 最高分 ： 82 | 第二十三章中文.txt | 3 | 22 |
| 312 | 话 只 当 我 没 说 。 ” 又 | 忿忿不平 地 哼 一 声 ， “ 我 就 不 | 第二十六章中文.txt | 2 | 8 |
| 313 | 炸药 。 ” 教室 里 有 几 个 人 | 恍然大悟 地 啊 了 一 声 。 邢老师 拍拍 | 第十六章中文.txt | 15 | 36 |
| 314 | 咚 ” 的 从 楼下 爬 上 来 ， | 大惊小怪 地 喊 ： “ 吵 什么 呢 ？ 吵 | 第九章中文.txt | 9 | 19 |
| 315 | ？ 我 又 不 是 犯 人 。 ” 金铃 | 做贼心虚 地 嘀咕 着 。 卉紫 似笑非笑 地 | 第二十二章中文.txt | 1 | 6 |
| 316 | 被 卉紫 灌 进 半 粒 安定片 ， | 不明不白 地 回 了 自己 熟悉 的 家 。 | 第十四章中文.txt | 18 | 47 |
| 317 | 出 去 ， 拔腿 奔 出 老远 ， 又 | 不失时机 地 回头 喊 一 句 ： “ 我 就 | 第二十二章中文.txt | 1 | 24 |
| 318 | 、 双眼发亮 。 她 | 连蹦带跳 地 回家 ， 走 在 楼梯 上 就 | 第二章中文.txt | 8 | 12 |
| 319 | 两 毛 钱 。 ” 她 小声 说 。 摊主 | 斩钉截铁 地 回答 ： “ 不行 ， 我 不 能 | 第十九章中文.txt | 14 | 22 |
| 320 | 一个 毛孔 都 充血 的 样子 。 金铃 | 伶牙俐齿 地 回答 他 ： “ 迟 什么 到 呀 | 第十八章中文.txt | 12 | 45 |
| 321 | 不 费劲 的 事 吗 ？ ” 金铃 却 | 理直气壮 地 回答 卉紫 ： “ 谁 让 你 和 | 第十三章中文.txt | 17 | 11 |

322 ：你 为什么 要 想 帮 我？” 孙奶奶 郑重其事 地 回答：“因为 我 喜欢 你 第二十二章中文.txt 1 51
323 口 气， 实在 不 知道 该 怎么 深入浅出 地 回答 女儿 这个 问题。 卉紫 第十八章中文.txt 12 35
324 ，飞快 地 往 眼睛 上 一 擦， 虚张声势 地 回答：“谁 哭 了？ 你 第二十二章中文.txt 1 35
325 有 个 当 大款 的 爸爸？” 金铃 闷声闷气 地 回答：“都 不 是。” 第十六章中文.txt 15 21
326 的 时候， 金亦鸣 已经 回来 了， 正 笨手笨脚 地 在 厨房 里 淘米 煮饭。 金 第二十三章中文.txt 3 41
327 了， 明天 是 ‘六一’ 儿童节。” 卉紫 开始 爬高落低 地 在 衣橱 中 为 金铃 找 第二十五章中文.txt 5
16
328 一个 位置 啊！ 卉紫 就 这么 醒醒睡睡， 一动不动 地 在 金铃 床 边 倚 了 第二十六章中文.txt 2 17
329 在 你们 身上 了。” 三 个 人 如释重负 地 坐 下来， 脸上 不免 都 有些 第十八章中文.txt 12 19
330 钱 的 时候， 财政 困难 了。 金铃 灰心丧气 地 坐 在 床 上， 绞尽脑汁 想 第十章中文.txt 21 8
331 呢？” 金铃 猛地 甩开 卉紫 的 手， 满脸是泪 地 大 声道：“因为 我 就 是 第十四章中文.txt 18 42
332 钱， 有 很多 很多 钱。” 全班 女生 不约而同 地 大 笑， 张灵灵 甚至 笑 得 第二十五章中文.txt 5 29
333 一个 月， 升学 考试 的 序曲 就 紧锣密鼓 地 奏响 起来。 校长 仿照 天安 第十八章中文.txt 12 1
334 大 叫：“我 要 吐 了！” 她 迫不及待 地 奔 到 一 棵 树 下， 第二十一章中文.txt 6 17
335 你 好 幸运 哦！” 倪志伟 在 一旁 阴阳怪气 地 学 着 金铃 讲话 的 腔调： 第十七章中文.txt 16 53
336 ，卉紫 的 神经 就 绷 紧 了， 寸步不离 地 守 在 金铃 旁边。 去 厨房 第八章中文.txt 0 32
337 又 结结巴巴 地 对 他 说：“不得了， 不 第二十四章中文.txt 4 44
338 天 金铃 兴冲冲 地 从 外面 回来， 大喊大叫 地 对 卉紫 说：“我 见到 那 第十章中文.txt 21 6
339 句 话：“考 了 多少 分？” 金铃 哭笑不得 地 对 妈妈说：“奶奶 怎么 就 第十二章中文.txt 13 26
340 会 有 一分 钱 的 进项。 她 面色庄严 地 对 妈妈说：“现在 我 真的 第十章中文.txt 21 39
341 检查 当然 都 免 了， 邢老师 还 郑重其事 地 对 金铃 和尚 海 道 了 第五章中文.txt 22 19
342 哄 闹 一阵 过后， 监考 老师 也 无可奈何 地 封 了 卷。 四 个 毕业班 第十八章中文.txt 12 70
343 运算， 金铃 算 到 第二 步 时， 不可思议 地 将 其中 一个 数 丢弃 不 第七章中文.txt 11 13
344 终究 不 敢 违抗 妈妈 的 话， 可怜巴巴 地 就 着 几 根 榨菜 丝， 第八章中文.txt 0 30
345 也 还 不 够 大， 鱼 只能 勉为其难 地 屈身 其中， 头 和 尾巴 都 第十五章中文.txt 19 21
346 公道 的 经过， 又 把 10 块 钱 得意洋洋 地 展示 出来。 卉紫 听 得 有 第五章中文.txt 22 21
347 时 单位 里 分发 的。 因为 它们 毫无例外 地 已经 很 不 新鲜， 当 妈妈 第十五章中文.txt 19 8
348 台上 台下 一阵 笑。 -个 电视台 记者 眼疾手快 地 帮 她 拿 过 琴， 一直 第十七章中文.txt 16 24
349 摆开。 果然 就 有 一 条 蚕 探头探脑 地 往 上 爬。 爬 了 一半， 第二十三章中文.txt 3 13
350 看见， 就 解 下 红领巾 包 着， 遮遮掩掩 地 往 教学楼 后面 走。 教学楼 第十九章中文.txt 14 25
351 教室 走 的 路上， 金铃 一直 在 绞尽脑汁 地 想 怎么 出 一个 既 新鲜 第十九章中文.txt 14 34
352 影星 们 出， 谁 让 她们 那么 迫不及待 地 想 漂亮 呢？” 卉紫 忍 第二十三章中文.txt 3 16
353 就 溜 进 了 自己 的 房间， 不声不响 地 打开 书包 做 作业。 卉紫 第二十四章中文.txt 4 15
354 也 差点 弄翻。“这 是 什么？” 卉紫 莫名其妙 地 打开 盒盖，“我 的 天！ 第二十一章中文.txt 6

36

| | | | | | |
|---|---|---|---|---|---|
| 355 | ，甜 得 要命 … … ” 金铃 嫌 他 啰嗦 ， | 急不可待 地 打断 他 的 话 ： “ 别的 以 | 第二十二章中文.txt | 1 | 13 |
| 356 | 填 肚子 就 很 不错 了 … … ” 金铃 | 伶牙俐齿 地 打断 她 的 话 ： “ 从 前 | 第八章中文.txt | 0 | 27 |
| 357 | 。 卉紫 | 东奔西走 地 托 了 好多 人 打听 ， 总算 | 第十三章中文.txt | 17 | 13 |
| 358 | 书本 ， 完全 彻底 地 休息 、 放松 。 金铃 | 一丝不苟 地 执行 了 这个 命令 ， 坚决 不 | 第二十六章中文.txt | 2 | 10 |
| 359 | ， 转 着 圈 儿 地 东张西望 。 金亦鸣 | 痛心疾首 地 批评 它们 ： “ 太 娇惯 了 | 第二十二章中文.txt | 1 | 19 |
| 360 | 追究 是 谁 开 了 窗户 ， 大家 | 众口一词 地 把 倪志伟 推 了 出来 。 倪 | 第十八章中文.txt | 12 | 72 |
| 361 | 监考 老师 执行 命令 。 两 个 人 | 手忙脚乱 地 把 学生 未 做 完 的 | 第十八章中文.txt | 12 | 67 |
| 362 | 看看 金铃 ， 又 看看 自己 的 筷子 ， | 一声不响 地 把 胳膊 收 了 回去 。 金亦 | 第九章中文.txt | 9 | 35 |
| 363 | 动画片 人物 玩具 啦 。 无论 店主 多幺 | 漫不经心 地 把 这些 东西 放在 多幺 不起眼 | 第1章中文.txt | 23 | 4 |
| 364 | 了 。 ” 金铃 说 。 张灵灵 喘 着 气 ， | 心有余悸 地 把 那 只 盛 有 蚕宝宝 | 第二十一章中文.txt | 6 | 19 |
| 365 | 撂 了 挑子 ， 叫 我 一个 老太太 | 上蹿下跳 地 折腾 出 这 期 刊物 ？ ” | 第二十章中文.txt | 7 | 27 |
| 366 | 步 ， 探 身 到 教室 门外 看看 ， | 挤眉弄眼 地 报告 尚海 ： “ 真 的 是 往 | 第十八章中文.txt | 12 | 7 |
| 367 | 不等 卉紫 发话 ， 两 个 人 就 | 迫不及待 地 抬 着 大鱼 往 门 里 | 第十五章中文.txt | 19 | 18 |
| 368 | 的 小摊子 ， 就 | 熟门熟路 地 拐 进 另 一 条 巷子 ， | 第二十四章中文.txt | 4 | 32 |
| 369 | 了 吗 ？ 那天 上 着 课 ， 她 | 鬼使神差 地 拿 出 数学 草稿本 ， 一遍遍 | 第六章中文.txt | 10 | 10 |
| 370 | 的 舌尖 上 蠕动 、 翻滚 、 挣扎 。 张灵灵 | 得意洋洋 地 指 着 嘴巴 ， 口齿 含糊不清 | 第二十一章中文.txt | 6 | 12 |
| 371 | 成绩 。 她 终于 还是 到 了 家 ， | 垂头丧气 地 按响 了 门铃 。 妈妈 开 了 | 第六章中文.txt | 10 | 3 |
| 372 | 偏 不 想 让 妈妈 猜中 。 她 | 一声不响 地 掏 出 一 张 卷子 ， 是 | 第六章中文.txt | 10 | 4 |
| 373 | 出 一 副 大 侦探 的 派头 ， | 自言自语 地 推理 和 判断 。 金铃 不能 | 第二十五章中文.txt | 5 | 51 |
| 374 | 是 刘娅如 ， 然后 是 总 被 金铃 | 忿忿不平 地 提 在 嘴边 的 那 个 | 第二十四章中文.txt | 4 | 30 |
| 375 | 做 班主任 的 失败 了 。 ” 尚海 有点 | 于心不忍 地 插嘴 说 ： “ 这 不 能 怪 | 第二十五章中文.txt | 5 | 27 |
| 376 | 好 的 细 树枝 ， 沿 鞋盒 四角 | 纵横交错 地 摆开 。 果然 就 有 一 条 | 第二十三章中文.txt | 3 | 12 |
| 377 | 上 的 尚海 一起 ， 两 个 人 | 结结实实 地 摔 在 地上 。 金铃 脸上 擦 | 第十一章中文.txt | 20 | 26 |
| 378 | 碗 里 只 剩下 了 汤水 。 金铃 | 心满意足 地 擦 了 嘴 ， 开始 宣布 一 | 第十章中文.txt | 21 | 35 |
| 379 | ‘ 在 帮 你 补课 。 ” 金铃 这 才 | 完完全全 地 放 下 心 ， 手里 抓 着 | 第二十二章中文.txt | 1 | 49 |
| 380 | 。 卉紫 看 着 金铃 的 高兴 样 ， | 不失时机 地 教育 她 说 ： “ 这 下 看到 | 第十五章中文.txt | 19 | 26 |
| 381 | 自卑 ， 嘴 上 又 不 肯 服气 ， | 虚张声势 地 替 自己 辩护 ： “ 很 多 大 | 第十七章中文.txt | 16 | 64 |
| 382 | 具 权威性 的 指点 ？ 金铃 的 成绩 | 立竿见影 地 有 了 提高 ， 卉紫 怎么 能 | 第二十四章中文.txt | 4 | 52 |
| 383 | 被 这 小小的 蜘蛛 消灭 了 。 金铃 | 绘声绘色 地 朗读 一 遍 后 ， 金亦鸣 和 | 第十七章中文.txt | 16 | 76 |
| 384 | 次 ， 只 有 那么 一 次 。 ” 老太太 | 若有所思 地 望 着 她 ： “ 你 很 坦白 | 第二十二章中文.txt | 1 | 41 |
| 385 | 是 被 谁 要求 的 呢 ？ ” 倪志伟 | 自作聪明 地 望 着 大家 。 邢老师 摆摆 | 第二十五章中文.txt | 5 | 28 |

| | | | | | | |
|---|---|---|---|---|---|---|
| 386 | 去 看病 的， 心里 不 免 紧张， | 张口结舌 地 望 着 对方。 同事 把 一 | 第七章中文.txt | 11 | 32 |
| 387 | 那里 插队 的 爸爸 头 上， 爸爸 | 毫不犹豫 地 汇 去 了 20万 元， 帮助 | 第十六章中文.txt | 15 | 9 |
| 388 | 去 了 呢？ 天 哪， 小 东西 | 自作主张 地 滚 到 别人 桌子 下面 去 | 第十八章中文.txt | 12 | 59 |
| 389 | 做， 设 每份 数 是 x。” 老师 | 无可奈何 地 点点 头：“好 吧， 请 | 第二十一章中文.txt | 6 | 30 |
| 390 | ：“努力！” 这样 想 的 时候， 她 已经 | 迫不及待 地 猫 下 腰 去， 用 劲 | 第十三章中文.txt | 17 | 14 |
| 391 | 问 了 句：“想 买 花？” 金铃 | 不好意思 地 用 手 指 着 塑料桶 里 | 第十九章中文.txt | 14 | 21 |
| 392 | ， 把 猫 制服 在 沙发 上。 卉紫 | 手忙脚乱 地 用 绳子 将 它 的 脖子 | 第十四章中文.txt | 18 | 37 |
| 393 | ， 一 眼 就 看见 金铃 和 尚海 | 面色苍白 地 瘪 缩 在 办公室 里， 满 | 第六章中文.txt | 10 | 50 |
| 394 | 。 她 咳嗽 一 声， 故意 将 眉毛 | 痛苦不堪 地 皱 成 一 团 疙瘩：“刚才 | 第十八章中文.txt | 12 | 6 |
| 395 | 出来， 头 一 抬， 卉紫 正 双手抱臂、 | 目光炯炯 地 盯 着 她 呢。“你 监视 | 第二十二章中文.txt | 1 | 5 |
| 396 | 无奈 地 看 着 金铃。 金铃 也 | 可怜巴巴 地 盯住 老师， 眼睛 里 满 含 | 第十三章中文.txt | 17 | 15 |
| 397 | 吧？” 李林 妈妈 猛地 直 了 腰， | 目光灼灼 地 看 住 卉紫。 卉紫 心里 就 | 第十三章中文.txt | 17 | 20 |
| 398 | 不 出来。“怎么样？ 不行 了 吧？” 金铃 | 洋洋得意 地 看 妈妈 的 笑话。 卉紫 猛 | 第二十三章中文.txt | 3 | 26 |
| 399 | 芭比 娃娃。 金铃 趴 在 柜台 上 | 目不转睛 地 看， 眼 珠子 恨 不 能 | 第九章中文.txt | 9 | 42 |
| 400 | 我 有 特殊 情况， 真 的！” 金铃 | 可怜巴巴 地 看 着 老太太 的 眼睛 说， | 第二十二章中文.txt | 1 | 37 |
| 401 | 认真。 其实 我们 小时候……” 她 发现 余老太 | 若有所思 地 看 着 她， 就 改 口 | 第二十章中文.txt | 7 | 29 |
| 402 | ， 原来 又 是 一 堆 习题册。 金铃 | 目瞪口呆 地 看 着， 一 句 话 也 | 第十四章中文.txt | 18 | 25 |
| 403 | 在 最 前排 的 尚海 说 他 | 清清楚楚 地 看见 了 金铃 眼睛 里 的 | 第十六章中文.txt | 15 | 43 |
| 404 | 你， 谁 也 不 敢 开口。 邢老师 | 故作惊讶 地 睁大眼睛：“怎么？ 每年 | 第二十五章中文.txt | 5 | 7 |
| 405 | 移 到 桌边， 捧 起 鞋盒， 又 | 半步半步 地 移 出门。 她 走 这么 慢 | 第二十一章中文.txt | 6 | 41 |
| 406 | 的 坏 孩子？” 金铃 低 着 头， | 半步半步 地 移 到 桌边， 捧 起 鞋盒， | 第二十一章中文.txt | 6 | 40 |
| 407 | 着 不 跟 她 走， 脚步 却 | 不由自主 地 移动 了， 好像 老太太 身上 | 第二十二章中文.txt | 1 | 32 |
| 408 | 小孩子， 你 什么 都 不 懂！” 金铃 | 手足无措 地 立 在 她 身边。 杨小丽 继 | 第二十五章中文.txt | 5 | 69 |
| 409 | 班 上 最 好 的 孩子。" 金铃 | 一动不动 地 站 了 半 天。 然后 她 | 第二章中文.txt | 8 | 22 |
| 410 | 人 欺侮 了 尚 海， 金铃 就 | 横眉竖眼 地 站 出来 了， 肩膀 一 扛 | 第五章中文.txt | 22 | 1 |
| 411 | 去， 邢老师 随手 把门 带 严。 金铃 | 一声不响 地 站 在 邢老师 身边， 嗅到 了 | 第二十五章中文.txt | 5 | 60 |
| 412 | 来。 卉紫 再 睁眼 时， 幸幸 已经 | 开开心心 地 站 在 她 面前 了。 原来 | 第九章中文.txt | 9 | 33 |
| 413 | ， 她 还 学 什么 学 呀？” 金铃 | 不声不响 地 站 在 一旁， 看 着 妈妈 | 第十四章中文.txt | 18 | 21 |
| 414 | 高跟 皮鞋 一路 嗒嗒 的 响 着， | 面无表情 地 站立 在 尚海 面前， 问 他 | 第十八章中文.txt | 12 | 4 |
| 415 | 了 一 撮 茶叶， 冲 进 开水， | 泼泼洒洒 地 端 到 妈妈 面前。 妈妈 扭 | 第六章中文.txt | 10 | 54 |
| 416 | 在 自己 胸口。 金铃 嘴角 一 翘， | 不好意思 地 笑 了。 一边 笑， 一边 | 第十九章中文.txt | 14 | 67 |

| | | | | | |
|---|---|---|---|---|---|
| 417 | 得 重 写 。” 尚海 就 捂 住嘴 | 幸灾乐祸 地 笑 。 邢老师 收 好 了 试卷 | 第二十五章中文.txt | 5 | 5 |
| 418 | 缺点 ？ ” 卉紫 问 ： “ 为什么 要 这样 ？ ” | 金铃 不好意思 地 笑笑 ： “ 妈妈 你 想 嘛 ， | 第九章中文.txt | 9 | 27 |
| 419 | 胳膊 ， 问 她 究竟 多少 分 。 她 | 恶声恶气 地 答 一 句 ： “ 管 好 你 | 第六章中文.txt | 10 | 18 |
| 420 | 吃 金铃 的 喜糖 噢 ！ ” 金铃 就 | 开开心心 地 答应 ： “ 好 啊 ！ ” 一边 | 第二十六章中文.txt | 2 | 20 |
| 421 | 短 的 距离 。 奶奶 咂 着 嘴 ， | 万分痛惜 地 絮叨 着 ： “ 怎么 就 考 这 | 第十二章中文.txt | 13 | 27 |
| 422 | 声音 说 ： “ 哦 -- 女人 的 诗 ？ ” 卉紫 | 迫不及待 地 翻 到 一 页 ， 情意绵绵 地 | 第十七章中文.txt | 16 | 68 |
| 423 | 电影 上 那些 外国 明星 的 样子 ， | 若无其事 地 耸 一 耸 肩膀 。 傍晚 放学 | 第十一章中文.txt | 20 | 28 |
| 424 | 笑 呢 ！ 我 心里 很 快乐 ！ ” 卉紫 | 忧心忡忡 地 自语 ： “ 怎么 回事 ？ 明天 | 第十八章中文.txt | 12 | 29 |
| 425 | 瞪 着 一 双 失神 的 眼睛 ， | 万分惊恐 地 自语 道 ： “ 它 这 是 报复 | 第十四章中文.txt | 18 | 19 |
| 426 | ” 。 还 好 ， 她 没 出 意外 就 | 稳稳当当 地 落 在 了 院子 里 ， 这 | 第二十二章中文.txt | 1 | 28 |
| 427 | 你 这 双 悲哀 的 眼睛 ！ 你 | 奄奄一息 地 蜷缩 在 马路 旁 ， 翅膀 上 | 第十九章中文.txt | 14 | 54 |
| 428 | 表情 ， 对 脚下 的 障碍物 疏忽 了 ， | 毫无防备 地 被 倪志伟 绊 了 一 跤 ， | 第十一章中文.txt | 20 | 25 |
| 429 | 往 里 瞟 了 一 眼 ， 竟 | 身不由己 地 被 窗口 里 的 风 吸 | 第十八章中文.txt | 12 | 69 |
| 430 | 书包 不 放 ， 马上 取出 一 只 ， | 热情万分 地 要 金铃 背上 试试 感觉 。 | 第十七章中文.txt | 16 | 8 |
| 431 | 她 在 杂志社 里 开会 ， 余 老太 | 没完没了 地 讲 下 一 期 的 组稿 | 第二十三章中文.txt | 3 | 32 |
| 432 | 得 再 好 又 怎么样 ？ 到 时候 | 结结巴巴 地 说 不 出 话 来 ， 看 | 第十七章中文.txt | 16 | 82 |
| 433 | 从 这个 世界 上 消失 一样 。 卉紫 | 脸色发白 地 说 ： “ 不准 你 再 说 这样 | 第十二章中文.txt | 13 | 23 |
| 434 | 自己 的 身体 不 行 吗 ？ ” 卉紫 | 斩钉截铁 地 说 ： “ 不行 ！ ” 金铃 | 第八章中文.txt | 0 | 29 |
| 435 | 决定 。 金铃 换 了 个 口 气 ， | 推心置腹 地 说 ： “ 于 胖 儿 ， 你 如 | 第五章中文.txt | 22 | 17 |
| 436 | 得 上 气 不 接 下 气 ， | 断断续续 地 说 ， “ 于胖儿 … … 于胖儿 | 第二十五章中文.txt | 5 | 30 |
| 437 | 胖 ， 将来 没有 男孩子 喜欢 怎么办 ？ ” 金铃 | 理直气壮 地 说 ： “ 他们 不 喜欢 是 他 | 第八章中文.txt | 0 | 9 |
| 438 | 了 起来 。 他 指 着 习题 纸 | 气急败坏 地 说 ： “ 你 看看 你 看看 ！ | 第六章中文.txt | 10 | 28 |
| 439 | 买 下 。 张灵灵 却 拿 了 架子 ， | 漫不经心 地 说 ： “ 你 这 表 都 戴 | 第十三章中文.txt | 17 | 53 |
| 440 | 道理 再 向 人家 要 钱 ！ ” 校长 | 斩钉截铁 地 说 ， “ 你们 完全 可以 不用 | 第十三章中文.txt | 17 | 38 |
| 441 | 一下 爸爸 妈妈 ， 嘴里 含 着 肉块 | 呜噜不清 地 说 ： “ 你们 吃 呀 。 ” | 第十章中文.txt | 21 | 34 |
| 442 | 他 。 ” 邢老师 接着 叫 倪志伟 到 办公室 ， | 满脸严肃 地 说 ： “ 刚才 派出所 打电话 | 第十一章中文.txt | 20 | 36 |
| 443 | 在 那里 了 ， 这时候 就 一 笑 ， | 幸灾乐祸 地 说 ： “ 原来 你 比 我 还 | 第六章中文.txt | 10 | 40 |
| 444 | ？ 你 简直 不 懂 欣赏 。 ” 金铃 | 理直气壮 地 说 ： “ 在 我们 班 上 ， | 第十七章中文.txt | 16 | 74 |
| 445 | 蛋炒饭 里 的 鸡蛋 拣 给 金铃 ， | 自言自语 地 说 ： “ 天天 放学 后 参加 | 第二十三章中文.txt | 3 | 8 |
| 446 | 她 出事 。 她 到底 怎么 啦 ？ ” 邢老师 | 哭笑不得 地 说 ： “ 她 来 例假 了 。 | 第二十五章中文.txt | 5 | 58 |
| 447 | ， 多 没出息 。 ” 金铃 眨巴 了 一下 眼睛 ， | 似笑非笑 地 说 ： “ 妈妈 说话 自相矛盾 | 第八章中文.txt | 0 | |

20

| | | | | | | |
|---|---|---|---|---|---|---|
| 448 | 不 知道 刚才 发生 了 什么 事， | 笑靥如花 地 说： | “ 妈妈 你 为什么 板 | 第二十三章中文.txt | 3 | 5 |
| 449 | 还 回 到 现实 。” 邢老师 收起 笑脸， | 忧心忡忡 地 说： | “ 孩子 们， 我 本来 | 第二十五章中文.txt | 5 | 39 |
| 450 | 揉 来 揉 去， 吭哧 了 半天， | 吞吞吐吐 地 说： | “ 尚海 … … 还 有 | 第五章中文.txt | 22 | 4 |
| 451 | 地 追 上去， | 旁敲侧击 地 说： | “ 张灵灵 家里 有 一个 | 第九章中文.txt | 9 | 45 |
| 452 | 在 旧 报纸 里 忘 了？” 卉紫 | 哭笑不得 地 说： | “ 忘 什么！ 除非 你 | 第八章中文.txt | 0 | 19 |
| 453 | 的 7 题 一道 也 没 错。 张老师 | 大惑不解 地 说： | “ 怎么 会 这样 呢？ | 第六章中文.txt | 10 | 31 |
| 454 | 地 指 着 嘴巴， 口齿 | 含糊不清 地 说： | “ 怎么样？ 看到 了 | 第二十一章中文.txt | 6 | 13 |
| 455 | ， 当心 老师 没收 了 你 的 。” 金铃 | 大大咧咧 地 说： | “ 我 连 这 一点 自制 | 第二十四章中文.txt | 4 | 2 |
| 456 | 快 去 买 两 个 彩卷？” 于胖儿 | 自告奋勇 地 说： | “ 我 去 吧 。” | 第二十五章中文.txt | 5 | 46 |
| 457 | 一 层 虚汗。 教 自然 的 任老师 | 恍然大悟 地 说： | “ 我 知道 邢老师 是 | 第十九章中文.txt | 14 | 3 |
| 458 | 早读课 的 老师 若是 干涉， 他 就 | 振振有词 地 说： | “ 我 妈 说 了， 不 | 第十九章中文.txt | 14 | 7 |
| 459 | 怎么 会 变 得 这么 世故！” 金铃 | 郑重其事 地 说： | “ 我 并 不 想 说谎 | 第十六章中文.txt | 15 | 22 |
| 460 | 想， 还 是 仅仅 不 敢？” 金铃 | 吞吞吐吐 地 说： | “ 我 … … 什么 … | 第十七章中文.txt | 16 | 58 |
| 461 | 这个 发现 及时 报告 了 卉紫， 卉紫 | 无可奈何 地 说： | “ 拿 两 毛 钱 去 | 第二十二章中文.txt | 1 | 10 |
| 462 | 吓 得 嘴唇 发 白， 站 起来 | 结结巴巴 地 说： | “ 没 … … 没 说 … | 第十八章中文.txt | 12 | 5 |
| 463 | 事。 金铃 故意 做出 不经意 的 模样， | 轻描淡写 地 说： | “ 没什么， 邢老师 叫 | 第十九章中文.txt | 14 | 43 |
| 464 | ， 会 懂得 你们 的 好意 的 。” 邢老师 | 忧心忡忡 地 说： | “ 照 这样 下去， 再 | 第二十章中文.txt | 7 | 6 |
| 465 | 明白， 他们 自然 会 发奋 读书 。” 卉紫 | 疑疑惑惑 地 说： | “ 照 你 这么 说， | 第十章中文.txt | 21 | 4 |
| 466 | 地 嘀咕 着。 卉紫 | 似笑非笑 地 说： | “ 真 的 上 厕所 了 | 第二十二章中文.txt | 1 | 7 |
| 467 | 我 再 出去 找 老同事 问问？” 卉紫 | 心灰意懒 地 说： | “ 算 了， 问 也 是 | 第二十章中文.txt | 7 | 23 |
| 468 | 赵卉紫 抱 拳 作 了 个 揖， | 嬉皮笑脸 地 说： | “ 还是 夫人 有 办法 | 第六章中文.txt | 10 | 72 |
| 469 | 没 问题 。” 金铃 这时候 才 犹豫 起来， | 期期艾艾 地 说： | “ 还有 一 件 事， | 第十九章中文.txt | 14 | 51 |
| 470 | ， 做 普通 劳动者 也 很 光荣 。” 金铃 | 轻描淡写 地 说： | “ 那 我 愿意 做 普通 | 第八章中文.txt | 0 | 22 |
| 471 | 一 歇 才 能 打扫 “ 战场 ”。 卉紫 | 面有难色 地 说： | “ 那 你 先 出去， | 第十五章中文.txt | 19 | 38 |
| 472 | 主持人， 还 不 该 减 减肥？” 卫老师 | 哭笑不得 地 说。 | 金铃 心里 也 有些 自 | 第十七章中文.txt | 16 | 63 |
| 473 | 干 了 吗？” 金铃 说：“ 去！” 老板娘 | 大惊小怪 地 说： | “ 金铃 今天 吃 火药 | 第五章中文.txt | 22 | 14 |
| 474 | 陌生 的 男人 的 声音， 那 人 | 凶声凶气 地 说： | “ 钱 准备 好 没有？ | 第十三章中文.txt | 17 | 40 |
| 475 | 成 粉末 吹 上去。 她 自己 先 | 大惊小怪 地 赞美、 欣赏， | 又 打电话 告 | 第二十六章中文.txt | 2 | 11 |
| 476 | 。 邢老师 | 急急忙忙 地 赶 了 过来， | 把 杨小丽 前后 | 第二十五章中文.txt | 5 | 54 |
| 477 | ， 大家 自然 也 就 不好 追问。 赵卉紫 | 心急火燎 地 赶到 学校， | 一 眼 就 看见 | 第六章中文.txt | 10 | 49 |

478 ，仗 着 是 杨小丽 的 好朋友， 便 脸老皮厚 地 跟 了 过去， 活像 条 傻乎乎 第二十五章中文.txt 5 57
479 回家 。” 金铃 不 回答 她 的 话， 不紧不慢 地 跟 在 身后， 脸上 也 没有 第十七章中文.txt 16 28
480 要 笑 得 肚皮 发 痛 。 杨小丽 愁眉苦脸 地 跟 金铃 商量： “ 我 出 个 第十一章中文.txt 20 7
481 的 水花 。 卉紫 心惊胆战 地 踮 着 脚 过去， 轻轻地 拍拍 第十八章中文.txt 12 28
482 家中 报纸 摊 了 一 地， 妈妈 蓬头垢面 地 蹲 在 一 堆 报纸 后面 第八章中文.txt 0 18
483 。 娇气 一点 的 女生， 比如 刘娅如 、 张灵灵， 见缝插针 地 躲 到 了 草坪 处 的 第二十五章中文.txt 5 48
484 地 问 。 李林 妈妈 大大咧咧 地 进 了 门， 一 屁股 在 第十三章中文.txt 17 27
485 就是了 。” 金铃 收 了 纸 和 棍子， 缩头缩脑 地 进 门， 也 不 敢 嚷嚷 第二十四章中文.txt 4 14
486 ，拖拉 着 旅游鞋 的 两 根 鞋带， 气喘吁吁 地 追 上 她们 。 “ 迟到 没有 第十八章中文.txt 12 44
487 排 塑料 玩具 上 。 金铃 却 是 不屈不挠 地 追 上去， 旁敲侧击 地 说： 第九章中文.txt 9 44
488 太 大， 金铃 马上 犯 了 晕， 毫不客气 地 错 了 。 另 一 道 是 第七章中文.txt 11 12
489 ： “ 已经 解决 了 。” 卉紫 一头雾水， 懵懵懂懂 地 问： “ 什么 解决 了？ ” 第4章中文.txt 25
10
490 台 游戏机 都 要 不 到 。” 倪志伟 横眉竖眼 地 问： “ 你 说 谁 呢？ 谁 第十七章中文.txt 16 54
491 找 个 借口 坐 到 她 旁边， 轻言细语 地 问 她： “ 你 是 不 是 第二十四章中文.txt 4 51
492 。” 金铃 把 头 埋 在 卉紫 胸前， 瓮声瓮气 地 问： “ 妈妈 你 说， 世界 第九章中文.txt 9 51
493 之后， 邢老师 第一 个 回过 神 来， 和颜悦色 地 问： “ 李林 同学， 你 为 第二十五章中文.txt 5 24
494 怎么 了？ 有 什么 不好 吗？ ” 她 惊慌失措 地 问 。 李林 妈妈 大大咧咧 地 第十三章中文.txt 17 26
495 的 小妇人 。 “ 都 弄 好 了？ ” 邢老师 轻言细语 地 问 。 杨小丽 垂 着 眼皮， 第二十五章中文.txt 5
64
496 她 裙子 后面 -- 看见 了 吗？ ” 金铃 大惑不解 地 问： “ 没 什么 呀！ ” 第二十五章中文.txt 5 49
497 ，不客气 地 打断 了 她 的 话， 直截了当 地 问： “ 说 吧， 你 有 什 第十章中文.txt 21 15
498 了， 我 见到 那个 女人 了！ ” 卉紫 莫名其妙 地 问： “ 谁 呀？ 哪个 女人 第十章中文.txt 21 7
499 只 有 低头 聆听 的 份儿 。 卉紫 小心翼翼 地 问： “ 邢老师 看， 金铃 第4章中文.txt 25 39
500 眼泪 差点儿 又 要 掉 下来 了， 结结巴巴 地 问： “ 那么 … … 那么 … 第十九章中文.txt 14 33
501 。 等 男生 们 开心 过 了， 杨小丽 小心翼翼 地 问： “ 那么 … … 谁 演 第十一章中文.txt 20 17
502 也 傻 了 眼 。 她 扭 过头， 结结巴巴 地 问 金铃： “ 这个 … … 这 第十五章中文.txt 19 16
503 实现 自己 的 愿望 。” 金铃 点点 头， 彬彬有礼 地 问答： “ 谢谢 。” 碘钨 第十七章中文.txt 16 26
504 ，笤帚 才 接触 到 地面， 尘土 就 迫不及待 地 飞扬 起来， 仿佛 它们 就 专 第二十三章中文.txt 3 1
505 你 了 。” 那 声音 接着 说 。 金铃 万般无奈 地直 起身， 垂 下 头 不 敢 第二十二章中文.txt 1 29
506 的 许诺， 而且 开口 就 给 了 1000 块 。 天 哪！ 1000 块 第十章中文.txt 21 20
507 。 一个 普通 人家， 哪里 是 说 拿 3000 块 就 能 拿 3000 块 的 呢 第十三章中文.txt 17 47
508 。 这样 吧， 我 大方 点， 给 你 1000 块， 所有 的 吃 用 开销 都 第十章中文.txt 21 19
509 块 就 能 拿 3000 块 的 呢？ 还好， 金亦鸣 的 第十三章中文.txt 17 48

| | | | | |
|---|---|---|---|---|
| 510 | 住院， 吃药 打针 加 石膏 绷带， 没有 2000 块 钱 下不来， 这 是 医生 告诉 | 第十三章中文.txt | 17 | 31 |
| 511 | 了 笑： “那 也 行， 你 拿 3000 块 钱 出来， 我 就 不再 上门 | 第十三章中文.txt | 17 | 28 |
| 512 | 。 那 人 说： “叫 你 妈妈 送 3000 块 钱 到 新华 医院 去， 听见 | 第十三章中文.txt | 17 | 41 |
| 513 | ， 又 被 李林 家 敲竹杠 敲 去 3000 块 钱， 卉紫 的 情绪 就 非常 | 第十四章中文.txt | 18 | 1 |
| 514 | 诈骗 人家 钱财？ 简直 岂 有 此理！ 3000 块 钱 我 决 不 给 她， | 第十三章中文.txt | 17 | 35 |
| 515 | 块。 天 哪！ 1000 块 钱 是 个 多 大 的 | 第十章中文.txt | 21 | 21 |
| 516 | 块？” “高？ 3000 块 钱 算 高 吗？” 李林 妈 | 第十三章中文.txt | 17 | 30 |
| 517 | 太 高 了， 凭 什么 要 我们 3000 块？” “高？ 3000 块 | 第十三章中文.txt | 17 | 29 |
| 518 | 探头 再 看 浴缸 里， 鱼 块 横七竖八 堆 了 一 缸， 白 生生的 鱼肉 | 第十五章中文.txt | 19 | 39 |
| 519 | 拿 满分！ 争取 胜利！ 先 做 填空题。 相对而言 填空题 总 是 简单 一些。 第一 | 第十八章中文.txt | 12 | 53 |
| 520 | 样 的 嘴巴 里 发出 轻微 的 “ 叭嗒叭嗒” 声， 然后 那 片 桑叶 眼见得 | 第二十三章中文.txt | 3 | 11 |
| 521 | ， 学校 的 操场 和 游乐场 里 都 空无一人。 夕阳 的 余辉 照 在 教学楼 | 第二十三章中文.txt | 3 | 34 |
| 522 | 最 好 的 “珠江” 牌 钢琴 才 1000 多 块 钱。 卉紫 和 金亦鸣 结 | 第十七章中文.txt | 16 | 31 |
| 523 | 剩 一个 邢老师 在 她 身后。 卉紫 不好意思 多 夸 女儿， 只 笑 一 笑： | 第4章中文.txt | 25 | 35 |
| 524 | 钱 一 根 的 冰棍 能 买 1000 多 根！ 上帝 啊， 那 时候 | 第十章中文.txt | 21 | 23 |
| 525 | 矮 了 下来， 嘴里 嘀嘀 咕咕。 金铃 不依不饶， 大声 追 了 一 句： “态度 | 第十九章中文.txt | 14 | 37 |
| 526 | 强， 凡事 要求 完美， 金铃 却 是 天性粗疏， 大大咧咧， 不 是 这儿 错 了 | 第十七章中文.txt | 16 | 43 |
| 527 | 还 做 过 我 的 学生。” 金铃 轻叹一声： “天 哪！” 她 觉得 这 | 第二十二章中文.txt | 1 | 39 |
| 528 | ： “这 是 一 颗 老鼠屎 呀！” 卉紫 两手一拍： “天 哪， 果然 是！ 老 | 第十四章中文.txt | 18 | 11 |
| 529 | ： “这 有 什么 不 懂 的？ 因为 宇宙空间 太 浩大 了 呗。 就 好像 我们 | 第七章中文.txt | 11 | 25 |
| 530 | 帮 我 倒 点 热水！” 金铃 放下 自动铅笔 奔 进 厨房， 捏 着 鼻子 去 | 第十五章中文.txt | 19 | 10 |
| 531 | 睡 午觉。 金亦鸣 完成 这个 任务 同样 一丝不苟。 女儿 睡熟 了， 他 自己 也 | 第3章中文.txt | 24 | 6 |
| 532 | 海参 和 皮肚。 金铃 是 奶奶 的 心肝宝贝， 奶奶 疼 她 的 唯一 方式 就 | 第十二章中文.txt | 13 | 10 |
| 533 | 奖励。 金铃 拿 到 这 钱 之后 大喜过望。 她 一 方面 替 妈妈 心疼， | 第十章中文.txt | 21 | 42 |
| 534 | ， 李林 却 是 人高马大， 她 伸手 一 扯， 非但 没 扯 | 第十九章中文.txt | 14 | 10 |
| 535 | 作业， 我 坐 在 旁边 看 着， 如此而已。” “她 信任 你……” | 第二十四章中文.txt | 4 | 57 |
| 536 | 同意。 她 感觉 花钱 的 过程 太 惊心动魄， 她 受不了 那种 割肉剜心 的 悲 | 第十章中文.txt | 21 | 40 |
| 537 | 的。 卉紫 这 一 天 在 杂志社 心神不定。 她 告诫 自己： 工作 要 紧 | 第九章中文.txt | 9 | 29 |
| 538 | ， 眼冒金星。 她 咽 一 口 唾沫， 心里 | 第十八章中文.txt | 12 | 52 |
| 539 | 的 那 一 条 被 压 得 吱哇乱叫。 她 忍不住 伸手 到 课桌 下 | 第二十一章中文.txt | 6 | 24 |
| 540 | 自己 刚才 的 动情 朗诵 完全 是 对牛弹琴。 她 忿忿不平 地 责问： “ | 第十七章中文.txt | 16 | 72 |
| 541 | ， 还 是 不行， 尘土 呛 得 她 透不过气。 她 放下 笤帚， 站 到 楼梯 | 第二十三章中文.txt | 3 | 2 |
| 542 | 就 急忙 生 下 了， 实在 是 问心有愧。 她 满月 下 床 后 就 开始 | 第八章中文.txt | 0 | 1 |
| 543 | 小公鸡。 整个 照相 过程 中， 金铃 几乎 寸步不离 她 的 好朋友 杨小丽。 她 不断 | 第二十五章中文.txt | 5 | |

65
544 在 门 上 听 了 听， 里面 悄无声息 。 她 试 着 伸手 一 推， 门 第十九章中文.txt 14 26
545 中学 的！ 复习 材料 都 咬 得 七零八落， 她 还 学 什么 学 呀？ ” 第十四章中文.txt 18 20
546 治病， 还 不 是 照样 管用？ ” 卉紫 举手投降： “ 好 好 好， 听 你 的。 第二十六章中文.txt 2 12
547 ， 心情 非常 愉悦 地 连连点头： “ 好吃， 好吃。 ” 外婆 第十二章中文.txt 13 7
548 。 ” 金亦鸣 跟 着 说： “ 你 妈妈 有点 走火入魔 。 ” 好好 的 一 顿 欢乐 晚餐 第九章中文.txt 9 36
549 就 放心 了 吗？ 否则 总 这么 疑神疑鬼， 好好 的 人 也 要 疑 出 第七章中文.txt 11 16
550 开始 扳 着 手指 算 细账， 嘴里 念念有词： 如果 一个 月 只 用 去 200 第十章中文.txt 21 22
551 温热 的 小小 尸体， 我 的 眼泪 夺眶而出 。 如果 我 是 巨人， 我 真 第十九章中文.txt 14 64
552 ， 中不溜儿 。 如果 按照 邢老师 的 说法， 第二十章中文.txt 7 11
553 她 跟 金铃， 一切 不 能 不 多加小心 。 妈妈 一 看 堵 在 门口 的 第十五章中文.txt 19 15
554 年级 五 年级 … … ” 金铃 大 叫 起来： “ 说来说去， 妈妈 你 只 是 在 找 借口 第九章中文.txt 9
17
555 它 吃 了 半 粒 安定片 。 ” 金铃 又惊又喜： “ 妈妈 你 答应 让 我 养 猫 第十四章中文.txt 18 27
556 热水 已经 将 一部分 冻鱼 烫 得 皮开肉绽 。 妈妈 唠叨 说： “ 真是 一 第十五章中文.txt 19 12
557 ” ， 不 把 她 当 老师 的 爸爸 放在眼里 。 妈妈 在 金铃 报到 的 这天 第十六章中文.txt 15 3
558 是 世界 上 独一无二 的 好 “ 款爷 ” ？ 从此以后， 妈妈 对 爸爸 改变 了 看法， 第十六章中文.txt 15 13
559 。 ” 妈妈 跑 去 看， 盒子 里 果然 空空如也 。 妈妈 这 回 真 气 了， 捉 第1章中文.txt 23 24
560 勃发 激情 喷涌 时， 能 写 得 酣畅淋漓 妙语连珠， 令 老师 击掌 赞叹 。 第1章中文.txt 23 7
561 说 吧 。 ” 这样， 金铃 和尚 海 的 1000 字 检查 当然 都 免 了， 邢老师 第五章中文.txt 22
18
562 你 赶 出 补习班！ 回到 教室 写 1000 字 的 检查， 下午 带 10 块 第五章中文.txt 22 11
563 出 来 一 封 印 有 “ 长江 百货公司 ” 字样 的 信， 里面 说 金铃 第十七章中文.txt 16 17
564 再 去 时， 果然 就 被 拒之门外 。 孙奶奶 只 同意 她 以后 每 第二十四章中文.txt 4 59
565 脸 说： “ 不 许 问 人， 要 独立思考 。 ” 孩子 屁股 动 了 动， 第七章中文.txt 11 22
566 单元 测验， 没 必要 每次 考试 都 如临大敌， 孩子 的 分数 不 是 那么 重要 第九章中文.txt 9 30
567 有 多 笨， 做 家长 的 都 没脸见人 。 孩子 的 成绩 是 衡量 家长 成 第4章中文.txt 25 12
568 狭小 的 空间 无法 将 肩上 的 庞然大物 安置 下来， 又 转身 奔 向 一旁 第十五章中文.txt 19 19
569 我 长大 》 。 文章 说 “ 长百 ” 的 东西 价廉物美， 家里 所有 的 耐用 商品 和 电 第十七章中文.txt 16 15
570 身上， 睡 一 觉 起来 又 会 活蹦乱跳； 家长 的 累 是 累 在 心里 第二十章中文.txt 7 14
571 思路， 帮助 学生 复习 时 就 能够 有的放矢， 对症下药 。 ” 卉紫 慌忙 道 第二十章中文.txt 7 19
572 进 重点 高中 能 考 一流 大学？ 一分之差， 将来 的 命运 就 是 南辕北辙 第六章中文.txt 10 59
573 跟 谁 都 能够 “ 自 来 熟 ”， 男女老幼 尊 卑 贵贱， 她 一概 都 能 第1章中文.txt 23 2
574 有 人 开始 表演 了， 是 一个 瘦骨嶙峋 、 小 脸 上 只 看 见 两 第十七章中文.txt 16 80

| | | | | | |
|---|---|---|---|---|---|
| 575 | 和 李林 几 个 人 焦急 地 | 转前转后， 小声 询问 别人： “诺贝尔 | 第十六章中文.txt | 15 | 32 |
| 576 | 不 可能， 但是 总 能 有 办法 “ | 手下留情” 。 少 扣 一分 半 分， 这 | 第二十六章中文.txt | 2 | 4 |
| 577 | 呼哨。 金铃 狠 瞪 他 一 眼， | 大喝一声： “尚海！ ” 尚海 连忙 抽 | 第十九章中文.txt | 14 | 38 |
| 578 | 一 次？ 邢老师 话音 刚 落， 全班 | 掌声雷动， 尚海 开心 得 把 帽子 都 扔 | 第十一章中文.txt | 20 | 4 |
| 579 | ， 一点 也 没有 妈妈 笔下 的 那种 | 潇洒流畅。 尚海 把头 探 过 来 看 了 | 第六章中文.txt | 10 | 11 |
| 580 | ， 任何 时候 总 想 显示 自己 的 | 与众不同。 尚海 甚至 激动 得 离开 座位 | 第十六章中文.txt | 15 | 30 |
| 581 | 两 团 红晕， 简直 就 窘迫 得 | 无地自容。 尚海 的 小聪明 劲 也 算是 | 第十九章中文.txt | 14 | 13 |
| 582 | 干。 ” “不 对！ 没 干 你 为什么 | 鬼鬼祟祟？ ” 尚海 闭 紧 了 嘴唇 不 | 第六章中文.txt | 10 | 33 |
| 583 | 下去。 女生 们 也 想 笑， 又 | 不好意思， 就 一个 个 用 衣袖 捂 着 | 第十九章中文.txt | 14 | 14 |
| 584 | 有 他 这样 的 正气 和 勇敢， | 坏人坏事 就 不 会 发生。 和 李平乐 对 | 第十一章中文.txt | 20 | 34 |
| 585 | ， 不能 允许 有 任何 空白 和 遗漏。 | 差之毫厘， 就 与 你 期盼 中 的 100 | 第二十三章中文.txt | 3 | 30 |
| 586 | 卉紫 看见 金铃 搀 着 幸幸 上楼， | 自然而然 就 想起 几 年 前 的 事， | 第九章中文.txt | 9 | 15 |
| 587 | 觉得 可怕。 ” 邢老师 送 走 卉紫 之后， | 当天下午 就 拿 出 自习课 的 时间 开 | 第十六章中文.txt | 15 | 25 |
| 588 | 一 把 捂 紧 了 嘴， 生怕 | 一不留神 就 笑 出 声音。 花园 里 没 | 第二十二章中文.txt | 1 | 27 |
| 589 | ， 面容 严肃， 看上去 她 不 是 | 嘻嘻哈哈 就 能 混 过去 的。 金铃 不知 | 第六章中文.txt | 10 | 27 |
| 590 | 难道 没有 公理 存在 了 吗？ 靠 | 胡搅蛮缠 就 能 诈骗 人家 钱财？ 简直 岂 | 第十三章中文.txt | 17 | 34 |
| 591 | 同学。 一个 老师 见 她 在 门外 | 探头探脑， 就 走 出来 问 她 找 谁， | 第二十三章中文.txt | 3 | 39 |
| 592 | ， 一个 个 在 座位 上 把 身子 | 扭来扭去， 就 跟 吃 了 颗 “定心丸” | 第二十六章中文.txt | 2 | 7 |
| 593 | 很 正规 的 衬衫， 红领巾 系 得 | 端端正正。 就 连 很 少 修饰 打扮 的 | 第二十五章中文.txt | 5 | 18 |
| 594 | 味精 的、 买 方便面 手纸 洗涤剂 的， | 进进出出。 就 连 美发店 的 生意 也 火 | 第九章中文.txt | 9 | 1 |
| 595 | 不 会， 只 是 写 的 时候 | 一不小心 就 随手 用 了 另 一个 字， | 第1章中文.txt | 23 | 9 |
| 596 | 去 开 门。 门外 站 着 一个 | 二十七八 岁 的 女人， 初冬 天气 穿一 条 | 第九章中文.txt | 9 | 49 |
| 597 | 。 电话 追 到 杂志社， 卉紫 吓 得 | 面无人色， 差点儿 也 跟着 昏 过去。 | 第八章中文.txt | 0 | 39 |
| 598 | 分毫， 却 被 李林 反手 拉 得 | 踉踉跄跄， 差点儿 跌进 李林 的 怀里。 | 第十九章中文.txt | 14 | 11 |
| 599 | 撑住 讲台 两边， 目光 在 教室 里 | 扫来扫去。 已经 有 几 个 同学 举手 了 | 第二十一章中文.txt | 6 | 26 |
| 600 | 的……” “那 是 前些日子。 现在 我 的 | 雄心壮志 已经 烟消云散， 一切 从 现实 出 | 第二十四章中文.txt | 4 | 4 |
| 601 | 个 把 小 广告， 大学 时代 的 | 雄心壮志 已经 烟消云灭， 唯一 的 希望 就 | 第十二章中文.txt | 13 | 19 |
| 602 | 儿子 好像 今年 也 考 中学？ ” 馨兰 | 莞尔一笑： “已经 解决 了。 ” | 第4章中文.txt | 25 | 9 |
| 603 | 学校。 ” 邢老师 表扬 他： “不错， 教育 是 | 立国之本， 希望工程 就 希望 大家 捐助。 | 第二十五章中文.txt | 5 | 34 |
| 604 | 教室 装扮 得 漂亮 一点， 每个 同学 | 量力而行 带 一些 零食， 瓜子 话梅 巧克力 | 第十一章中文.txt | 20 | 6 |

| | | | | | |
|---|---|---|---|---|---|
| 605 | 去 动物园 看 梅花鹿， 当 爸爸 的 | 自告奋勇 带 她 去 了。 那天 天气 不 | 第3章中文.txt | 24 | 8 |
| 606 | 的 好菜。 外婆 会 做 | 各种各样 带 馅 和 不 带 馅 的 | 第十二章中文.txt | 13 | 5 |
| 607 | 打扮 得 漂亮 一些， 就 起劲 地 | 跑前跑后 帮 幸幸 挑 衣服。 她们 替 幸幸 | 第九章中文.txt | 9 | 38 |
| 608 | 帮忙， 凡是 单位 里 有 复印机 的， | 量力而行 帮助 班里 复印 试卷， 一月 一 | 第十八章中文.txt | 12 | 13 |
| 609 | 牢牢 记住 了， 并且 做 得 这么 | 一丝不苟。 幸幸 2 洗 完 了 金铃 洗 | 第九章中文.txt | 9 | 25 |
| 610 | 轻身宝”、“比索”、“大印象”、“使你美”、“苗条 霜”…… | 五花八门， 应有尽有。 人 其实 很 奇怪 | 第八章中文.txt | 0 | 14 |
| 611 | 闲 来 无 事， 看看 金庸 的 | 武侠小说 应该 合适。 卉紫 是 在 下午3 | 第二十四章中文.txt | 4 | 55 |
| 612 | 到底 想 拿 个 什么 分数？” 金铃 | 脱口而出：“100！” 底下 就 有 | 第十八章中文.txt | 12 | 23 |
| 613 | 高矮 顺序 排 好， 一个 个 已经 | 汗流满面。 张灵灵 不住 地 催促：“ | 第二十五章中文.txt | 5 | 43 |
| 614 | 他：“去！” 尚海 快快 地 说：“真 | 不够意思。” 张老师 在 黑板 上 写 完 | 第二十一章中文.txt | 6 | 25 |
| 615 | 成绩 会 比 别人 差！” 全班 一齐 | 哈哈大笑。 张老师 搔搔 头皮， 也 跟 着 | 第二十六章中文.txt | 2 | 9 |
| 616 | 个 人 不知 何事， 赶紧 站 起来， | 面面相觑。 张老师 说：“你们 三 个 | 第十八章中文.txt | 12 | 18 |
| 617 | 能 有 节余， 那么 节余 的 钱 | 可不可以 归 我？” 卉紫 问：“你 | 第十章中文.txt | 21 | 18 |
| 618 | 一门心思 奔 事业 奔 前途 将 来得 | 诺贝尔奖 当 部长 总理？ 赵卉紫 在 同学 | 第3章中文.txt | 24 | 3 |
| 619 | 忙 着 把 蘸 了 番茄酱 的 | 炸土豆条 往 嘴里 塞。 这样 的 美味 平 | 第六章中文.txt | 10 | 15 |
| 620 | 满满 一 书包 新 发 的 书本 | 弓腰曲背 往 家里 走， 一路 上 都 咬牙切 | 第十六章中文.txt | 15 | 1 |
| 621 | 下 一个 教室 的 窗口 时， 它 | 探头探脑 往 里 瞟 了 一 眼， 竟 | 第十八章中文.txt | 12 | 68 |
| 622 | ， 卉紫 一路 频频 换 手， 只 觉 | 肩酸背疼， 很 后悔 没 让 金亦鸣 陪 女儿 | 第十七章中文.txt | 16 | 27 |
| 623 | 。 文章 写 得 非常 朴实， 却 是 | 情真意浓， 很 让 人 感动。 卉紫 替 | 第十七章中文.txt | 16 | 16 |
| 624 | 胖乎乎 的 小 娃娃， 好 可爱！ 我 | 抑制不住 心中 的 兴奋之情 -- 啊！ 春天 | 第4章中文.txt | 25 | 30 |
| 625 | ？ 你 喜欢 吃 吗？” 金铃 吃 得 | 满嘴流油， 心情 非常 愉悦 地 连连点头： | 第十二章中文.txt | 13 | 6 |
| 626 | 一 把 抓住 似的。 金铃 吓 得 | 面无人色， 心跳 如 鼓， 拔腿就逃。 | 第十七章中文.txt | 16 | 86 |
| 627 | 的 签字 都 是 差不多 的。 金铃 | 如愿以偿， 心里 松 了 一 大 口 气。 | 第六章中文.txt | 10 | 9 |
| 628 | 过 五线谱， 此 番 跟 着 老师 | 现学现卖， 心里 颇 有 点 “80岁学吹打 | 第十七章中文.txt | 16 | 38 |
| 629 | 都 像 被 呛 着 了 似的， | 又闷又痒， 忍不住 要 张口 咳嗽。 她 | 第十三章中文.txt | 17 | 16 |
| 630 | 托 着 一 样 东西， 走路 都 | 跌跌绊绊， 忘 了 怎么 迈 步子 似的。 | 第二十一章中文.txt | 6 | 5 |
| 631 | 一个 小 母亲 的 责任 呢。 卉紫 | 哭笑不得， 忙 替 小 女孩 擦 干净 屁股 | 第九章中文.txt | 9 | 13 |
| 632 | 有 点 拐 脚 的 男人， 他 | 一跛一跛 忙 着 用 水壶 给 鲜花 洒 | 第十九章中文.txt | 14 | 20 |
| 633 | 罢了。” | 邢老师：“金铃 这 孩子， 看 着 大大咧咧 快快 活活 什么 都 不在乎， 其实 | 第4章中文.txt | 25 | 36 |
| 634 | 对 它 这么 好， 它 早 就 | 摇头摆尾 快活 得 不知天上地下 了。 金铃 | 第二十三章中文.txt | 3 | 14 |

635 着 头， 皱 着 眉， 嘴 巴里 叽叽咕咕 念 着 什么。 金铃 笑 着 朝 第十八章中文.txt 12 39

636 了。” 她 脑子 里 灵光一闪， 忽 地 跳 起来， 冲 出 房门 第十章中文.txt 21 12

637 一个 关于 女儿 智力 的 怪圈 之中， 绕来绕去 怎么 也 跳 不 出来。 她 恨 第七章中文.txt 11 4

638 地 睁大眼睛： “怎么？ 每年 的 ‘六一 第二十五章中文.txt 5 8

639 的 面子！ 你们 以为 那 是 游乐场？ 一天到晚 怎么 就 那么 快活？ 六 年级 了 第五章中文.txt 22 10

640 ”， 戴 着 一 副 金丝 眼镜， 真是 威风十足。 怪不得 开 “的士” 的 司 第十六章中文.txt 15 6

641 起来 不 肯 见 人 呢。” 金铃 恍然大悟， 怪不得 这 几 天 看 黄 猫 第九章中文.txt 9 4

642 来。 无奈 猫 的 身手 实在 敏捷， 上蹿下跳 总 能 从 卉紫 手 间 逃脱。 第十四章中文.txt 18 32

643 个 既然 是 代表 学校 去 应聘， 不管怎样 总 要 争取 上 一个， 这 是 第十七章中文.txt 16 59

644 的 身材， 多多少少 总 还是 有 点 优势 的。 关键 第十三章中文.txt 17 9

645 汇款单 交到 赵卉紫 的 手上， 一家 人 皆大欢喜。 总的说来， 这 一 家 第3章中文.txt 24 12

646 弹琴 的 好手。 做 母亲 的 便 窃窃暗喜， 恍惚 中 看见 10 年 后 一个 第十七章中文.txt 16 34

647 往 家里 走， 一路 上 都 咬牙切齿， 恨 她 的 同学 太 “势利眼 第十六章中文.txt 15 2

648 ， 关 上门， 悄悄 问 她： “跟 外婆 说老实话， 想 不 想 出 去 玩？” 第十四章中文.txt 18 5

649 故意 皱 紧 眉头： “于胖儿 你 是 存心不良， 想 污染 空气， 让 我们 中毒 第十八章中文.txt 12 46

650 的 成 了 款爷 呢！” 金亦鸣 竭力 插科打诨， 想 要 打破 母女 间 此刻 的 第十六章中文.txt 15 19

651 地 坐 在 床 上， 绞尽脑汁 想 赚钱 的 主意。 卖 报？ 第十章中文.txt 21 9

652 回 哪儿？” 卉紫 这 一 惊 更是 非同小可， 想 金铃 这 孩子 真 是 越 第九章中文.txt 9 16

653 评语 写 得 真 好， 妈妈 是 无论如何 想不到 的。" 金铃 不为所动 第二章中文.txt 8 8

654 无所适从。 想想 从前 自己 上学 读书 的 第七章中文.txt 11 6

655 她 心里 去 了。 父女 俩 立刻 亲亲热热 成 了 同 一 条 战壕 的 第八章中文.txt 0 34

656 高兴 地 对 金铃 说： “你 倒是 一不小心 成 了 获奖 专业户。” 金铃 第十七章中文.txt 16 20

657 ， 所以 就 送 来 这么 一 条 “庞然大物”。 我 一 听， 心里 暖和 第十六章中文.txt 15 10

658 有 百分之 十 左右 的 淘汰率？” 馨兰 不以为然： “我 儿子 根本 不 打算 升 第二十章中文.txt 7 38

659 成语 你 听说 过 吗？” 卉紫 扑哧一笑： “ 岂有此理！ 我 可是 正经 大学 中文系 毕 第十七章中文.txt 16
30

660 带 着 我 对 小 青草 的 喜爱之情。 我 抬 起 头， 望 第4章中文.txt 25 32

661 错？ 这 可 怎么 得 了？” 校长 双手一摊： “我 有 什么 办法？ 我 第二十章中文.txt 7 7

662 学问 和 知识 加 起来 要 用 车载斗量。 我 生活 在 这样 的 家庭 里 第十六章中文.txt 15 38

663 里 来。 她 跨 进 房门 就 大叫一声： “我 的 天！” 紧 赶 两 第二十五章中文.txt 5 14

664 懂得 什么 是 真实， 什么 是 虚构。 一般来说 我们 不 提倡 孩子 把 作文 当 第十六章中文.txt 15 24

665 请 拨 110 电话， 巡警 同志 为 您 排忧解难’。 我们 打 110 报警 电话 第十五章中文.txt 19 31

666 桑树 吗？” “桑树？” 老板娘 被 问 得 莫名其妙， “我家 连 根 草 都 没有， 第二十二章中文.txt 1
22

| | | | | | |
|---|---|---|---|---|---|
| 667 | 。 整整 两 节 自习课， 竟 没有 人 | 东张西望 或是 起身 走动 一下， 真是 奇 | 第十九章中文.txt | 14 | 42 |
| 668 | 门口 的 声音 。 她 本来 是 在 | 闭目养神， 所以 听觉 特别 敏锐 。 她 | 第十九章中文.txt | 14 | 27 |
| 669 | 和 失误， 跟 对待 作业 的 态度 | 恰恰相反 。 所以 她 身后 发生 了 什么， | 第五章中文.txt | 22 | 9 |
| 670 | 服输 的 意味， 总 希望 女儿 能够 | 出类拔萃， 所以 对于 女儿 目前 的 状况 | 第4章中文.txt | 25 | 3 |
| 671 | 站 办公室 吗？ ” 全班 一阵 哄笑 。 邢老师 | 好不容易 才 把 自己 的 笑 忍 住， | 第二十五章中文.txt | 5 | 20 |
| 672 | 不 是 为了 争 当 雷锋， 而是 | 好不容易 才 有 了 这个 爬 房 上 | 第六章中文.txt | 10 | 24 |
| 673 | 咒语 哪？ ” 杨小丽 一 看 是 金铃， | 如逢救星， 扑 上去 抓住 金铃 的 胳膊： | 第十八章中文.txt | 12 | 40 |
| 674 | 想不到 的 。 " 金铃 | 不为所动， 扑哧 一 笑： " 你 又 不 | 第二章中文.txt | 8 | 9 |
| 675 | 都 是 自然数 。 对 或者 错？ 金铃 | 毫不犹豫 打 上 一个 叉 。 这 一分 毫无 | 第十八章中文.txt | 12 | 56 |
| 676 | 却 不 争气 地 往 下 淌， | 滴滴嗒嗒 打 得 书页 上 模糊 一 片 。 | 第二十一章中文.txt | 6 | 46 |
| 677 | ， 尚海 赶快 把 手 边 的 东西 | 一古脑儿 扫 进 抽屉， 转身 装作 没事 人 | 第六章中文.txt | 10 | 32 |
| 678 | 该 找 什么 医生 看， 又 不便 | 直截了当 找 人 打听， 怕 人 知道 了 | 第七章中文.txt | 11 | 10 |
| 679 | 亚于 猎犬 了， 她 很快 循 着 | 蛛丝马迹 找到 了 那 包 牛肉干 。 金亦鸣 | 第八章中文.txt | 0 | 37 |
| 680 | 在 美国 的 地址 要 了 来， | 工工整整 抄 在 一 张 纸 上， 央求 | 第二章中文.txt | 8 | 5 |
| 681 | 的 孩子 可 真 是 了不得！ 能 | 不动声色 把 一 件 事情 处理 得 滴 | 第五章中文.txt | 22 | 23 |
| 682 | 对 了 吗？ 她 红 着 脸， | 慌慌张张 把 卷子 塞 到 书包 里 。 尚海 | 第六章中文.txt | 10 | 17 |
| 683 | 的 最后 冲刺 前 见到 效果 。 卉紫 | 说干就干， 把 家里 的 报刊 杂志 统统 翻 | 第八章中文.txt | 0 | 13 |
| 684 | 得 来 劲 。 ” 金铃 就 有 些 | 不好意思， 把 捂 着 鼻子 的 手 放 | 第十五章中文.txt | 19 | 11 |
| 685 | 嘛， 急 什么 急？ ” 他 走 过去， | 乒乒乓乓 把 教室 两边 的 窗户 全 打开 | 第十八章中文.txt | 12 | 47 |
| 686 | 长气 。 用功 的 学生， 比如 胡梅， 则 | 不声不响 把 脑袋 埋 在 座位 下 翻看 | 第二十五章中文.txt | 5 | 2 |
| 687 | 拿 出 当 编辑 的 语言 水平， | 三言两语 把 金铃 的 情况 介绍 一 遍 。 | 第七章中文.txt | 11 | 27 |
| 688 | 是 很 有 策略 的， 他 只是 | 滔滔不绝 报 出 了 一连串 数字： 从 90 | 第4章中文.txt | 25 | 19 |
| 689 | 黄豆 。 那 时候 的 金铃 真 是 | 人见人爱， 抱 到 马路 上 看 街景， 南 | 第八章中文.txt | 0 | 3 |
| 690 | 大男子主义， 不 会 做 家务 事！ ” 卉紫 | 眉开眼笑， 抱 着 金铃 的 胖 脸蛋 亲 | 第十六章中文.txt | 15 | 40 |
| 691 | 本能 地 一跳， 头 从 抽屉 里 | 慌慌张张 抽 出来， 挤出 一 脸 灿烂 的 | 第二十一章中文.txt | 6 | 27 |
| 692 | 这 道 题 的 折中 办法， 把 | 3923 24 拆开 成 两 个 数： | 第六章中文.txt | 10 | 69 |
| 693 | 一 看， 马上 说： “ 跟 我 走 。 ” | 慌慌张张 拉 着 杨小丽 到 她 的 宿舍 | 第二十五章中文.txt | 5 | 55 |
| 694 | 了 虾， 买 了 猪肝 黄鳝 什么的， | 呼哧呼哧 拎 到 儿子 家里， 要 给 金铃 | 第十二章中文.txt | 13 | 30 |
| 695 | 。 金铃 如释重负， 拔腿 就 想 开 溜 。 走 到 | | 第二章中文.txt | 8 | 20 |
| 696 | 送 进 长江 | 百货公司， 拖 回家 这个 油光 锃亮 的 大 | 第十七章中文.txt | 16 | 33 |
| 697 | 了 怎么 迈 步子 似的 。 还有 人 | 大喊大叫， 招呼 更 多 的 同学 来 看 。 | 第二十一章中文.txt | 6 | 6 |
| 698 | 病人 是 不 能 生气 的， 就 | 小心翼翼 拣 些 不 那么 严重 的 事情 | 第十九章中文.txt | 14 | 30 |

| | | | | | |
|---|---|---|---|---|---|
| 699 | 大门 的 位置， 扑 在 门 上 | 又抓又挠， 指甲 磨 得 嘎吱嘎吱 响， 听 | 第十四章中文.txt | 18 | 30 |
| 700 | 就 有 好几 个 人 站 起来， | 七嘴八舌 指责 倪志伟 故作高深， 任何 时候 | 第十六章中文.txt | 15 | 29 |
| 701 | 真 的 会 离开 家园 一样。 金铃 | 翻箱倒柜， 挑 了 个 自己 最 喜欢 的 | 第二章中文.txt | 8 | 4 |
| 702 | 乘客、 危害 人民 生命 财产 安全 时， | 奋不顾身， 挺身而出， 与 歹徒 搏斗， | 第十一章中文.txt | 20 | 31 |
| 703 | 响。 这边 等 着 的 同学 因为 | 无所事事， 排 好 的 队伍 很 快 又 | 第二十五章中文.txt | 5 | 47 |
| 704 | 了， 都 是 些 低 年级 学生， | 规规矩矩 排 着 队， 过 了 马路 之后 | 第二十四章中文.txt | 4 | 24 |
| 705 | 完成， 学校 打印室 的 老师 忙 得 | 脚丫朝天， 揉 着 通红 的 眼睛 找 校长 | 第十八章中文.txt | 12 | 12 |
| 706 | ， 这 就 使 金铃 妈妈 有 了 | 可乘之机， 提 笔 将 扁 圆 描 得 | 第1章中文.txt | 23 | 18 |
| 707 | ！” 这一来 全班 情绪 更 是 激昂。 倪志伟 | 面红耳赤， 摆 出 一 副 恶狠狠 的 架势 | 第十六章中文.txt | 15 | 31 |
| 708 | 的 大 翻斗 里 倾泻 下来 似的， | 稀里哗啦 摊 了 一 地。 街上 顿时 多 | 第十七章中文.txt | 16 | 2 |
| 709 | 的 一 声， 铅笔 橡皮 小刀 本子 | 五颜六色 摊 了 一 片。 前后 左右 的 | 第1章中文.txt | 23 | 21 |
| 710 | 用 的 一 只 茶杯 洗 得 | 干干净净， 放 了 一 撮 茶叶， 冲 进 | 第六章中文.txt | 10 | 53 |
| 711 | 地面 滑 出 好 远。 李林 却 | 舍己救人， 放 着 自己 的 卷子 不 管， | 第十八章中文.txt | 12 | 62 |
| 712 | 停 它 也 停 不 下来。 赵卉紫 | 苦口婆心 教导 金铃：“女 孩子 太 胖 | 第八章中文.txt | 0 | 7 |
| 713 | 呢。 书包 柜台 上 是 真正 的 “ | 琳琅满目”， 数 一 数， 没有 100 | 第十七章中文.txt | 16 | 6 |
| 714 | ， 因为 他 参加 区 里 的 “小学生 | 奥林匹克 数学 竞赛” 获 了 二等奖， 数 | 第十一章中文.txt | 20 | 40 |
| 715 | 了， 可是 妈妈 没有 察觉， 仍然 在 | 絮絮叨叨 数落 人家 孩子 聪明， 好像 已经 | 第十四章中文.txt | 18 | 10 |
| 716 | 从 鞋 盒子 里 飞 出 来， | 没完没了， 整个 家中 都 蠕动 着 那些 灰 | 第二十二章中文.txt | 1 | 15 |
| 717 | 报告。 她 对 告状 的 行为 一向 | 不屑一顾。 整个 晚饭 时间， 金铃 都 | 第十一章中文.txt | 20 | 30 |
| 718 | 是 作文。” 卉紫 说：“作文 也 不能 | 胡说八道！” “文学 不 就是 虚构 吗 | 第十六章中文.txt | 15 | 14 |
| 719 | 个 新名词， 把 家长 们 弄 得 | 哭哭笑笑 无所适从。 想想 从前 自己 上学 | 第七章中文.txt | 11 | 5 |
| 720 | 张老师 骂 人 像 乌鸦， 全班 人 | 鸦雀无声 时， 他 冷不防 嘎 的 一 叫； | 第二十二章中文.txt | 1 | 48 |
| 721 | 来 控制 校长 和 老师。” 全班 同学 | 目瞪口呆 时， 他 跟 着 再 补充 一 | 第二十五章中文.txt | 5 | 21 |
| 722 | 、 做 不 出来 的 题目 怎么办 啦， | 心慌意乱 时 怎么办 啦， 发现 考卷 有 问 | 第二十六章中文.txt | 2 | 1 |
| 723 | 孩子 被 绑架 或是 拐卖， 急 得 | 口吐白沫 昏倒 在 地。 那 家人 又 是 | 第九章中文.txt | 9 | 10 |
| 724 | 老师 办公室 的 时候， 先 还 觉得 | 莫名其妙。 昨晚 张灵灵 家里 闹 得 乱哄哄 | 第五章中文.txt | 22 | 5 |
| 725 | 拔尖。 我 知道 你们 夫妇 都 是 | 知识分子， 是 心高气傲 的 人， 不 会 | 第二十章中文.txt | 7 | 16 |
| 726 | 打 上 一个 叉。 这 一分 | 毫无疑问 是 拿 到 手 了。 金铃 自喜 | 第十八章中文.txt | 12 | 57 |
| 727 | 是 什么 病 了！ 她 这 是 ‘ | 见字发晕’， 是 改 本子 改 得 太 多 | 第十九章中文.txt | 14 | 4 |
| 728 | 就 派 了 你们 两 个 来？” | 言下之意 是 新华街 小学 也 太 不 当 | 第十七章中文.txt | 16 | 81 |
| 729 | ， 轻轻 抿 了 抿 嘴。 全班 同学 | 一动不动， 显出 了 从未有过 的 肃穆 和 | 第二十五章中文.txt | 5 | 11 |
| 730 | ， 让 金铃 觉得 爸爸 也 有 点 | 神经兮兮。 晚上 金铃 做 作业， 卉紫 | 第二十四章中文.txt | 4 | 50 |
| 731 | 。 金铃 只是 不 喜欢 一点 小事 就 | 咋咋呼呼， 更 不 喜欢 频繁 到 老师 面前 | 第十一章中文.txt | 20 | 29 |

| | | | | | |
|---|---|---|---|---|---|
| 732 | 很多 很多 的 钱， 设立 一个 比 | 诺贝尔奖 更 伟大 的 奖。 我 要 让 | 第十六章中文.txt | 15 | 35 |
| 733 | 二十 | 求教无门 4月 底， 区 教育局 组织 所 | 第二十章中文.txt | 7 | 1 |
| 734 | 头， 表示 对 学校 里 的 做法 | 不可理喻。 3月 底， 学校 里 为 六 | 第十八章中文.txt | 12 | 17 |
| 735 | 显得 很 遗憾， 跟 爸爸 嘀咕 说， | 好不容易 有 一 次 扬眉吐气 签字 的 机会 | 第六章中文.txt | 10 | 44 |
| 736 | 在 美国 搬 过 家 了。" 金铃 | 怅然若失， 有 好几 天 都 闷闷不乐。 | 第二章中文.txt | 8 | 14 |
| 737 | 她 上 了 一 堂 作文课。” 卉紫 | 惊喜万分， 有些 大惊小怪 地 围 着 女儿 | 第十九章中文.txt | 14 | 44 |
| 738 | 大赛 的 事， 内容 限于 跟 长江 | 百货公司 有关 的 一切。 很多 人 围 着 | 第十七章中文.txt | 16 | 13 |
| 739 | 那么 长， 小 脑袋 很 稚气 地 | 昂来昂去， 有点 惊异 自己 身 处 的 环境 | 第二十一章中文.txt | 6 | 9 |
| 740 | 都 有 电视机 屏幕 的 彩色 画面 | 闪闪烁烁， 有的 放 新闻， 有的 放 天气 | 第十七章中文.txt | 16 | 84 |
| 741 | 里 刚 发 的 年货， 把 它们 | 分门别类， 有的 晾 挂 在 阳台 上， 有 | 第十五章中文.txt | 19 | 4 |
| 742 | 的 脸。 她 | 小心翼翼 朝 尚海伸 出 手：“ 给 我 一 | 第六章中文.txt | 10 | 37 |
| 743 | 天天 伸长 了 脖子 对 着 五线谱 | 连数带猜， 未免 心生烦躁。 一 烦躁 就要 | 第十七章中文.txt | 16 | 39 |
| 744 | ， 剩下 一个 实在 没 地方 坐， 干脆 | 倚墙而立， 本子 搁 在 前面 同学 的 后背 | 第二十章中文.txt | 7 | 44 |
| 745 | 偷窃 电脑， 被 人 发现 后 居然 | 丧心病狂 杀死 了 一个 目击者。 “ 教训 | 第二十四章中文.txt | 4 | 19 |
| 746 | 笑， 说：“ 它 在 我 肚子 里 | 爬来爬去。” 李小娟 脸色 苍白， 怕 | 第二十一章中文.txt | 6 | 16 |
| 747 | ， 挺身而出， 与 歹徒 搏斗， 最后 | 身负重伤。 李平乐 这个 平凡 的 人 做出 | 第十一章中文.txt | 20 | 32 |
| 748 | 他 出 教室。 谁 知道 音乐 老师 | 娇小玲珑， 李林 却 是 人高马大， 她 伸 | 第十九章中文.txt | 14 | 9 |
| 749 | ’s your | name？ 李林 就 回答 说： 波力。 | 第二十二章中文.txt | 1 | 45 |
| 750 | ， 已 知 什么 条件、 要求 什么 问题， | 清清楚楚 条理分明 的， 可 见 不 是 不 | 第七章中文.txt | 11 | 2 |
| 751 | ， 庄重 威严 地 说："别 跟 老师 | 嬉皮笑脸 来 这 一 套。" 金铃 只 觉 | 第二章中文.txt | 8 | 7 |
| 752 | 看看， 显得 比 杨小丽 自己 还 要 | 提心吊胆。 杨小丽 对 她 的 行为 哭笑不 | 第二十五章中文.txt | 5 | 66 |
| 753 | 我 又 不 想 当 保送生。” 金铃 | 一脸坦然。 杨小丽 想 了 一会儿， 还 | 第十九章中文.txt | 14 | 19 |
| 754 | ， 和 金铃 同学 一起 出席 颁奖会。 俗话说 “ | 好事成双 ”， 果然 是 有些 道理 的。 | 第十七章中文.txt | 16 | 18 |
| 755 | 喷 了 出来。 领奖 是 在 长江 | 百货公司 楼上 的 会议室， 大概 因为 顺带 | 第十七章中文.txt | 16 | 23 |
| 756 | 在 林中 快乐 地 做 巢、 鸣叫、 | 生儿育女、 歌唱 春天。 那里 有 世界 | 第十九章中文.txt | 14 | 65 |
| 757 | 内， 看见 校长 制作 的 那 座 “ | 倒计时钟 ” 正 显示 出 “ 36 天 ” 这 | 第二十三章中文.txt | 3 | 36 |
| 758 | 表演 了， 逗 得 全班 同学 | 哈哈大笑。 正好 那天 市 电视台 的 记者 | 第十一章中文.txt | 20 | 43 |
| 759 | 几 倍， 卉紫 想 起来 心里 还是 | 隐隐作痛。 此时 卉紫 明白 了 金铃 的 | 第十七章中文.txt | 16 | 48 |
| 760 | 弹 错， 越 错 越 要 挨骂， | 恶性循环， 母女 俩 如同 钻进 了 魔圈。 | 第十七章中文.txt | 16 | 41 |
| 761 | ， 也 不 | 图谋泄愤。 比如 她 给 杨小丽 的 作文 | 第十九章中文.txt | 14 | 50 |
| 762 | 的 男老师 之一， 前年 才 从 | 师范学校 毕业， 一 张 娃娃 脸上 还 残留 | 第六章中文.txt | 10 | 20 |
| 763 | 不 是 要 让 做 母亲 的 | 痛悔终生？ 毕竟 她 是 个 12 岁 的 | 第二十四章中文.txt | 4 | 17 |
| 764 | 问题， 赵卉紫 就 对 张老师 的 话 | 耿耿于怀， 没 事 就 总 想 金铃 智力 | 第七章中文.txt | 11 | 1 |

| | | | | | |
|---|---|---|---|---|---|
| 765 | ，肩膀 | 一抽一抽，泪水 糊 得 一 鼻子 一 脸， | 第二十五章中文.txt | 5 | 71 |
| 766 | 在 自己 的 被子 下面， 怕 蚕宝宝 | 不见天日 活活闷死， 还 仿照 游泳 时 潜水 | 第二十一章中文.txt | 6 | 34 |
| 767 | 。 金铃 每 碰到 这样 的 题目 总是 | 头昏眼花 、 浑身出汗 。 她 偷眼 看 一下 | 第六章中文.txt | 10 | 25 |
| 768 | 房间 的 墙上 抽泣 着， 泪珠 儿 | 大滴大滴 滚 落 下来 。 卉紫 心里 一 热 | 第九章中文.txt | 9 | 50 |
| 769 | 过 英国 女王 一样 的 生活 。” 赵卉紫 | 扑哧一笑， 满 肚子 的 气 都 消 得 | 第六章中文.txt | 10 | 73 |
| 770 | 出 这 副 架势 … …” 倪志伟 这时候 就 | 龇牙一笑：“演 猪八戒 的 人 有 了， | 第十一章中文.txt | 20 | 23 |
| 771 | 好。 这 与 她 想像 中 的 | 一师一徒 灯下细语 的 情景 差距 太 远， | 第二十章中文.txt | 7 | 45 |
| 772 | ‘七 进 七 出’， 也 就 是 | 早上7点 到 校， 晚上7点 放学 。 师范 | 第二十章中文.txt | 7 | 4 |
| 773 | 都 心痒痒 地 想 看 。 片子 在 | 晚上8点 到 8点 半 之间 播出， 到 | 第二十五章中文.txt | 5 | 3 |
| 774 | 到 校， | 晚上7点 放学 。 师范 附小 我 也 派 | 第二十章中文.txt | 7 | 5 |
| 775 | ？ 好像 是 有 这么 回事 。” 第二 天 | 早上6点 钟 不 到， 卉紫 就 起 | 第二十二章中文.txt | 1 | 16 |
| 776 | 应该 合适 。 卉紫 是 在 | 下午3点 钟 的 时候 按 响 铁栅门 | 第二十四章中文.txt | 4 | 56 |
| 777 | 二十五 最后 一个 儿童节 5月31日 的 | 下午5点 钟， 邢老师 收 齐 了 有关 | 第二十五章中文.txt | 5 | 1 |
| 778 | ？” 话音 刚 落， 全班 同学 欢呼 起来， | 拍手跺脚， 热闹非凡 。 金铃 想： 可 | 第二十五章中文.txt | 5 | 9 |
| 779 | 做 。” 他 随手 写 了 个 式子： 48×（ | 40-1 24）。 然后 他 用 48分 | 第六章中文.txt | 10 | 63 |
| 780 | 不 过 妈妈， 大 叫 一 声：“ | 欺人太甚！” 然后 就 把 自己 关 到 | 第七章中文.txt | 11 | 15 |
| 781 | 上 。 下 课 后 一 帮 男生 | 兴师动众 爬 房顶 帮 尚海 拿 文具盒， 导 | 第六章中文.txt | 10 | 23 |
| 782 | 不 敢 有 | 丝毫疏忽 。 片刻， 铁门 打开 了， 开 | 第二十四章中文.txt | 4 | 34 |
| 783 | 米面 油盐 一 类 生活 必需品， 其余 | 一概不买 。 牙膏 肥皂 都 要 省 着 用 | 第十章中文.txt | 21 | 26 |
| 784 | 到 了 脑科 医院， 赵卉紫 又 是 | 不容分说， 牵 着 金铃 的 手 就 往 | 第七章中文.txt | 11 | 19 |
| 785 | 。 卉紫 觉得 金铃 这 孩子 有 点 | 与众不同， 特别 喜欢 同情 和 帮助 弱小 | 第九章中文.txt | 9 | 24 |
| 786 | 不能 容忍 男生 对 她 的 好朋友 | 品头论足， 狠狠 地 瞪 了 尚海 一眼， | 第二十五章中文.txt | 5 | 52 |
| 787 | 一切 。 很多 人 围 着 在 看， | 七嘴八舌 猜测 有 什么 奖品， 是 东西 还 | 第十七章中文.txt | 16 | 14 |
| 788 | 打 过 几 次 "98" 的 高 分 。 | 好景不长， 王老师 教 了 金铃 不到 一 年 | 第二章中文.txt | 8 | 3 |
| 789 | 是 学习， 学习， 连 游戏机 都 没有 | 痛痛快快 玩 过 一 次 。 我 不 知道 | 第二十五章中文.txt | 5 | 72 |
| 790 | 有趣 的 事 吧 。” 老太太 要求 。 金铃 | 心事重重， 生怕 回去 晚 了 妈妈 骂 她 | 第二十二章中文.txt | 1 | 42 |
| 791 | 回家 的 时候 心里 还 有 点 | 忐忑不安， 生怕 妈妈 会 责怪 她 买 这么 | 第十章中文.txt | 21 | 44 |
| 792 | 了 。 奶奶 对 金铃 是 典型 的 | 百依百顺， 用 一 句“要上天拿梯子” | 第十二章中文.txt | 13 | 24 |
| 793 | 召开 班会 讨论 这 件 事， 决定 | 元旦那天 用 班费 买 些 彩纸 什么的 把 | 第十一章中文.txt | 20 | 5 |
| 794 | ， 这个 数 是 （ ）。 金铃 先 填 一个 “20 | .005” 。 用 笔尖 数数 小数点， 觉 | 第十八章中文.txt | 12 | 55 |
| 795 | 使 自己 看上去 苗条 一点 的 打算， | 大摇大摆 甩开 膀子 走路 了 。 校门口 挤 | 第二十一章中文.txt | 6 | 3 |
| 796 | ：“明天 是 一 号， 下 个 月 | 可不可以 由 我 当家？” 卉紫 惊讶 地 | 第十章中文.txt | 21 | 13 |

| | | | | | |
|---|---|---|---|---|---|
| 797 | 心里 对 金铃 有愧， 也 就 只好 | 装聋作哑， 由 着 金铃 。 星期六 学校 | 第七章中文.txt | 11 | 20 |
| 798 | ： 我 最 崇拜 谁？ 结果 当然 是 | 热闹非凡 。 男孩子 们 大都 选择 了 时下 | 第十六章中文.txt | 15 | 26 |
| 799 | 绝对 是 个 局外人 。 当时 她 正 | 全神贯注 画 一 张 美女 肖像 。 尚海 的 | 第五章中文.txt | 22 | 6 |
| 800 | 准确， 每 一个 错别字 都 分辨 得 | 清清楚楚 。 病句 、 读 不 通顺 的 句子 | 第十九章中文.txt | 14 | 47 |
| 801 | 的 小 蚕 活像 在 她 心里 | 爬来爬去， 痒丝丝 的 。 她 感觉 有 两 | 第二十一章中文.txt | 6 | 23 |
| 802 | 杨主任 此时 再 也 无法 保持 冷静， | 慌慌张张 登 上 讲台， 连声 地 喊 道： | 第十八章中文.txt | 12 | 66 |
| 803 | 数学 从来 就 不 进入 状态！ ” 金铃 | 懵懵懂懂 的， 不 知道 什么 叫 “ 进入 | 第六章中文.txt | 10 | 29 |
| 804 | … … 忽然 之间 才 觉得 彼此 都 是 | 标标准准 的 中年人 了 。 最 有趣 的 是 | 第4章中文.txt | 25 | 6 |
| 805 | 听 金铃 的 。 这时候 过来 了 一个 | 大腹便便 的 中年人， 他 一 来 就 惊讶 | 第十七章中文.txt | 16 | 88 |
| 806 | 失礼， 我 可 不 能 做 这样 | 无情无义 的 事 。 ” 结果 卉紫 还是 执 | 第二十四章中文.txt | 4 | 54 |
| 807 | 仗义 的， 危难 时候 不 会 做 | 落井下石 的 事 。 金铃 重 感情 。 邢 | 第十九章中文.txt | 14 | 17 |
| 808 | ， 是 | 心高气傲 的 人， 不 会 满足 于 让 | 第二十章中文.txt | 7 | 17 |
| 809 | 点 高兴 。 金铃 平常 不 是 个 | 爱出风头 的 人， 可 是 好事 既然 找上门 | 第十七章中文.txt | 16 | 60 |
| 810 | 不能不 警惕 。 倪志伟 就 是 这么 个 | 阴阳怪气 的 人， 奸坏奸坏 的， 杨小丽 | 第十一章中文.txt | 20 | 10 |
| 811 | 得 不 轻 。 我 倒 成 了 | 见钱眼开 的 人！ ” 金亦鸣 偏袒 女儿 | 第十六章中文.txt | 15 | 39 |
| 812 | 一点 什么， 就 没 必要 对 她 | 横眉竖目 的 。 你们 姐弟 几 个 读 小学 | 第十二章中文.txt | 13 | 17 |
| 813 | 里 的 洗 发 水 广告 有着 | 异乎寻常 的 兴趣， 再 加上 王老师 脾气 | 第二章中文.txt | 8 | 1 |
| 814 | 大 将军， 指挥 起来 颇 有 点 | 雷厉风行 的 军事家 气派 。 金铃 这时候 | 第十五章中文.txt | 19 | 40 |
| 815 | 着， 祝愿 女儿 能 拿 到 一个 | 漂漂亮亮 的 分数 。 3点 钟 一 过， | 第九章中文.txt | 9 | 31 |
| 816 | 钥匙 开 了 门， 走 进 稿件 | 堆积如山 的 办公室 。 “ 说说 罢了， | 第二十章中文.txt | 7 | 28 |
| 817 | 直 尾巴 、 摆 出 一 副 恶狠狠 | 不顾一切 的 劲儿， 卉紫 有 好几 次 伸 | 第十四章中文.txt | 18 | 33 |
| 818 | 大海 里 航行， 肯定 觉得 船 是 | 一动不动 的 。 ” 医生 不 高兴 地 扭头 | 第七章中文.txt | 11 | 26 |
| 819 | 钩 的 小 指头， 表示 她 是 | 说到做到 的 。 卉紫 这 一 天 在 杂志社 | 第九章中文.txt | 9 | 28 |
| 820 | 布置 的 作业 吗？ ” 妈妈 基本上 是 | 无话可说 的 。 即使 妈妈 不 同意， 爸爸 | 第二十五章中文.txt | 5 | 4 |
| 821 | ， 都 是 金铃 喜欢 去 的 地方 。 | 直截了当 的 原因 有 两 个： 去 的 | 第十二章中文.txt | 13 | 1 |
| 822 | 最 肥 的 鸡腿， 一 大 块 | 四四方方 的 叉烧肉， 或是 煎 好 的 四 | 第十二章中文.txt | 13 | 9 |
| 823 | 啃 着， 对 着 镜头 说： “ 谢谢 | 百货商店 的 叔叔 阿姨 送 我 这 台 | 第十七章中文.txt | 16 | 25 |
| 824 | 的 含量 以及 大脑 细胞 的 生长 、 | 肾上腺素 的 变化 等等， 使 读 它 的 | 第七章中文.txt | 11 | 34 |
| 825 | 12 岁 的 女孩子， 又 长 得 珠圆玉润 、 | 人见人爱 的 。 只 有 金亦鸣 没有 察觉 | 第二十四章中文.txt | 4 | 18 |
| 826 | | 条理分明 的， 可 见 不 是 不 懂 。 | 第七章中文.txt | 11 | 3 |
| 827 | 妈妈， 冷酷 的 妈妈， 比 灰姑娘 和 | 白雪公主 的 后妈 还要 后妈 的 妈妈！ | 第二十一章中文.txt | 6 | 44 |
| 828 | 身体 的 各种 指标 测定 、 血液 中 | 微量元素 的 含量 以及 大脑 细胞 的 生长 | 第七章中文.txt | 11 | 33 |
| 829 | ！ ” 医生 问 他： “ 你 知道 地球 是 | 一动不动 的 呢， 还是 转 个 不 停 | 第七章中文.txt | 11 | 21 |
| 830 | 孩子 的 口中， 带 着 那 种 | 铭心刻骨 的 哀伤 幽怨， 构成 了 对 成年 | 第十四章中文.txt | 18 | 44 |

| | | | | | |
|---|---|---|---|---|---|
| 831 | 来 却 是 让 每个 学生 都 | 心惊胆战 的， 因为 他 善于 把 粉笔头 掷 | 第六章中文.txt | 10 | 21 |
| 832 | 买 的 红领巾， 站 在 晨风 里 | 冉冉升起 的 国旗 下， 把 一 只 胳膊 | 第十六章中文.txt | 15 | 41 |
| 833 | 不 能 看！ ” 金铃 拿 出 了 | 从未有过 的 坚决 。 好 在 上课 铃 又 | 第二十一章中文.txt | 6 | 32 |
| 834 | 句子 排列 整齐 得 像 一 队 | 纪律严明 的 士兵 。 她 给 每 篇 作文 | 第十九章中文.txt | 14 | 48 |
| 835 | 阳光， 仿佛 上帝 已 赐予 它们 生命 。 | 一个陌生 的 声音 从 庞大 的 鱼 身后 | 第十六章中文.txt | 15 | 8 |
| 836 | 开 这种 玩笑？ 这时候 大鱼 身后 发出 | 瓮声瓮气 的 声音： “请问 这 是 不 是 | 第十五章中文.txt | 19 | 17 |
| 837 | 自豪 。 她 侧耳 细 听 房间 里 | 窸窸窣窣 的 声音， 转 过 脸 来 问 | 第二十五章中文.txt | 5 | 61 |
| 838 | 又 跟 他们 进 卫生间， 继续 用 | 结结巴巴 的 声音 问道： “这个 … … | 第十五章中文.txt | 19 | 23 |
| 839 | 李林 在 班 上 一向 是 个 | 令人头疼 的 “多动 症” 孩子 。 谁知 | 第十三章中文.txt | 17 | 17 |
| 840 | 的 狼狈 样， 的确 跟 电视 里 | 风度翩翩 的 大亨 形象 相差 过 远 。 钱 | 第二十五章中文.txt | 5 | 32 |
| 841 | 一 种 欢快 的 节奏， 与 门外 | 忧心忡忡 的 大人 的 面孔 正好 形成 反差 | 第二十六章中文.txt | 2 | 28 |
| 842 | 地 朗读： “我 的 灵魂 一度 是 | 果实累累 的 大树 。 那 时候， 人们 看 | 第十七章中文.txt | 16 | 69 |
| 843 | 。 她 想 这 一定 是 全 城里 | 独一无二 的 大鱼， 班 上 无论是 张灵灵 | 第十五章中文.txt | 19 | 25 |
| 844 | 有 点 慌 。 她 一向 怕 那些 | 目光灼灼 的 女人 。 卉紫 说： “真是 | 第十三章中文.txt | 17 | 21 |
| 845 | 有 你 的！ 你 真 是 个 | 标标准准 的 好 爸爸！ ” 晚上 妈妈 一 | 第十六章中文.txt | 15 | 11 |
| 846 | 把 小 脑袋 昂 得 老 高， | 东转西转 的， 好像 在 对 金铃 说： “ | 第二十二章中文.txt | 1 | 11 |
| 847 | ， 是 个 很 乖 的 、 懂事 的 、 | 讨人喜欢 的 好孩子 。 ” 金铃 出 了 一 | 第二十六章中文.txt | 2 | 15 |
| 848 | ” 、 “八宝 鸭” 、 “笋干 烧肉” ， | 都 是 金铃 百吃不厌 的 好菜 。 外婆 会 做 各种各样 | 第十二章中文.txt | 13 | 4 |
| 849 | 件 背心 坐 在 床 上， 肩膀 | 一抽一抽 的 。 妈妈 抱 着 她 的 肩膀 | 第二章中文.txt | 8 | 16 |
| 850 | 坐 下来， 摆 出 一 副 准备 | 长篇大论 的 姿态， 开始 对 金铃 进行 教 | 第八章中文.txt | 0 | 26 |
| 851 | 。 ” 四周 响起 一 片 咂嘴声 和 抱怨声 。 | 大大小小 的 孩子 们 开始 四散， 没有 买 | 第二十一章中文.txt | 6 | 7 |
| 852 | | 善解人意 的 孩子， 怎么 就 偏偏 学 不 | 第七章中文.txt | 11 | 31 |
| 853 | 。 外婆 常 指责 那些 除 学习 之外 | 万事不通 的 孩子， 痛心疾首 地 说： “ | 第十二章中文.txt | 13 | 15 |
| 854 | 金铃 看不起 她们！ 还有 于 胖 儿， | 溜须拍马 的 家伙， 为了 抄 人家 作业， | 第五章中文.txt | 22 | 13 |
| 855 | 没 多少 钱， 整天 编 编 那些 | 家长里短 的 小 稿子， 时不时 还 得 涎着 | 第十二章中文.txt | 13 | 18 |
| 856 | 飞快 地 光顾 了 一下 卖 日本 | 卡通画书 的 小摊子， 就 熟门熟路 地 拐 | 第二十四章中文.txt | 4 | 31 |
| 857 | 。 卉紫 的 脸 霎时 通红， 感觉 到 | 无处藏身 的 尴尬 。 她 狠狠 地 瞪 金铃 | 第七章中文.txt | 11 | 18 |
| 858 | 记忆， 元旦 是 不 是 组织 一个 | 热热闹闹 的 庆祝会， 大家 尽兴 狂欢 一 | 第十一章中文.txt | 20 | 3 |
| 859 | 斧头 干吗， 以为 我 家 来 了 | 打家劫舍 的 强盗 呢 。 ” 奶奶 年纪 大 | 第十五章中文.txt | 19 | 33 |
| 860 | 。 大家 眼巴巴 地 盯 在 倪志伟 脸上， | 忐忑不安 的， 心存戒备 的， 幸灾乐祸 的 | 第十一章中文.txt | 20 | 18 |
| 861 | 的 、 秃顶 的 、 花白 了 头发 的 、 | 疾病缠身 的 … … 忽然 之间 才 觉得 彼此 | 第4章中文.txt | 25 | 5 |
| 862 | 前 必 在 人 后， 这 是 | 毫无疑问 的， 思想 上 一点 都 不能 放松 | 第二十章中文.txt | 7 | 3 |

| | | | | |
|---|---|---|---|---|
| 863 | 甩 一 甩头 ， 甩掉 刚才 脑子 里 乱七八糟 的 思想 ， 苦 笑笑 说 ： “ 还 | 第二十章中文.txt | 7 | 15 |
| 864 | 的 食谱 … … 她 心里 有 一 种 欲哭无泪 的 悲伤 。 她 不 知道 孩子 生 | 第二十章中文.txt | 7 | 13 |
| 865 | 一样 淌 出去 ， 金铃 心如刀割 ， 实在 有种 欲哭无泪 的 悲伤 。 此时 的 金铃 对 “ | 第十章中文.txt | 21 | 38 |
| 866 | ， 她 受不了 那种 割肉剜心 的 悲痛 。 卉紫 就 跟 金亦鸣 | 第十章中文.txt | 21 | 41 |
| 867 | 灯下细语 的 情景 差距 太 远 ， 过去 的 | 第二十章中文.txt | 7 | 46 |
| 868 | 特写 ， 伴随 着 一 声 令 人 毛骨悚然 的 惨叫 ， 活像 要 伸出 玻璃门 | 第十七章中文.txt | 16 | 85 |
| 869 | ， 简直 就 有 把 人 抛 进 万丈深渊 的 感觉 呢 ！ 她 勉强 挣 出 | 第六章中文.txt | 10 | 51 |
| 870 | 这 才 止步 ， 摆 出 一 脸 上当受骗 的 愤怒 。 这 一 耽搁 ， 卉紫 | 第二十四章中文.txt | 4 | 38 |
| 871 | ？ 因为 没 得到 一等奖 ？ ” 金铃 忽然 问 ： “ ‘ 揠苗助长 ’ 的 成语 你 听说 过 吗 ？ ” | 第十七章中文.txt | 16 | 29 |
| 872 | 。 ” 男孩子 开始 兴奋 ， 跺脚 ， 吹 口哨 ， 做 各种各样 的 手势 。 邢老师 问 ： “ 是 | 第二十五章中文.txt | 5 | 26 |
| 873 | 金铃 吃 ， 这 就 变成 一 场 明目张胆 的 抵抗 减肥 运动 了 。 卉紫 | 第八章中文.txt | 0 | 38 |
| 874 | 模特 儿 而 自豪 。 每年 好莱坞 举办 奥斯卡奖 的 授奖 仪式 时 ， 我 的 办公室 | 第二十三章中文.txt | 3 | 15 |
| 875 | 进 教室 门 ， 看见 前后 黑板 上 密密麻麻 的 排名 ， 心里 就 紧张 起来 。 | 第二十章中文.txt | 7 | 9 |
| 876 | 了 ， 东倒西歪 的 ， 前仰后合 的 ， 揉 肚子 的 ， 擦 眼泪 的 | 第二十五章中文.txt | 5 | 37 |
| 877 | ” 正 显示 出 “ 36 天 ” 这个 让 人 心惊肉跳 的 数字 。 卉紫 三步并作两步 地 | 第二十三章中文.txt | 3 | 37 |
| 878 | 。 一 堂 普普通通 的 数学 课 ， 被 张老师 弄 得 | 第十八章中文.txt | 12 | 25 |
| 879 | 了 胸口 上 。 整整 一个 春节 ， 还有 春节过后 的 整个 春天 ， 金铃 的 家里 总 | 第十五章中文.txt | 19 | 43 |
| 880 | 。 在 主课 考试 尚未 提 上 议事日程 的 时候 ， 卉紫 先 要 为 金铃 | 第十三章中文.txt | 17 | 6 |
| 881 | ， 也 该 有 个 “ 一鸣惊人 ” 的 时候 。 “ 她 笨 吗 ？ | 第二十三章中文.txt | 3 | 21 |
| 882 | 间 把头 扭来扭去 的 时候 ， 我 和 你 的 目光 | 第十九章中文.txt | 14 | 61 |
| 883 | 买 菜 。 正 低头 跟 一个 鱼贩子 讨价还价 的 时候 ， 眼角 里 忽然 瞥见 一 | 第二十四章中文.txt | 4 | 8 |
| 884 | ” 的 厉害 ， 懂得 了 如今 在 世上 大行其道 的 是 什么 。 到 这 一 代 | 第十六章中文.txt | 15 | 23 |
| 885 | 来 得 特别 早 。 我 被 这个 美妙绝伦 的 景象 吸引 住 了 ， 痴痴 地 | 第4章中文.txt | 25 | 28 |
| 886 | 充满 热望 地 盯住 金铃 ， 一 副 深思熟虑 的 智者 的 模样 。 妈妈 在 厨 | 第十五章中文.txt | 19 | 13 |
| 887 | 申老师 、 教 自然 的 任老师 吓坏 了 ， 大呼小叫 的 ， 有的 扶 她 起来 ， 有的 | 第十九章中文.txt | 14 | 2 |
| 888 | 不 愿 放 过 这个 显示 自己 知识渊博 的 机会 ， 赶紧 插嘴 说 ： “ | 第七章中文.txt | 11 | 24 |
| 889 | 的 人 ， 奸坏奸坏 的 ， 杨小丽 背地里 给 他 取 了 | 第十一章中文.txt | 20 | 11 |
| 890 | 倪志伟 偏 要 摆 出 一 副 与众不同 的 架势 ， 说 他 最 崇拜 特工 | 第十六章中文.txt | 15 | 27 |
| 891 | 在 路上 的 时候 ， 自己 也 感觉 大腹便便 的 样子 不 太 雅观 。 她 使劲 | 第二十一章中文.txt | 6 | 2 |
| 892 | 、 丢三落四 的 样子 ， 不 是 算错 题 就 | 第十八章中文.txt | 12 | 34 |
| 893 | 着 一 双 手 ， 故意 装 出 满不在乎 的 样子 ， 似 听 非 听 。 上 | 第二章中文.txt | 8 | 18 |

894 看 她 说： “ 卉紫 你 怎么 啦？ 五心烦躁 的 样子。” 卉紫 说： “ 第二十章中文.txt 7 37
895 耳朵， 鼻子 皱 起来， 做出 一 副 不堪忍受 的 样子： “ 妈呀， 色 死 了 第十七章中文.txt 16 71
896 着 妈妈 为 她 的 复习 材料 心疼不已 的 样子， 心里 忽然 产生 一 丝 第十四章中文.txt 18 22
897 受宠 过 多， 弄 成 一 副 张牙舞爪 的 样子。 直到 9点 钟 睡觉 第九章中文.txt 9 23
898 人 打交道。 她 鄙视 他 那 副 小人得志 的 样子。 至于 猪八戒 的 角色 第十一章中文.txt 20 41
899 这 段 时间 社会 上 流窜犯 很多， 入室打劫 的 案件 频频发生， 此时 家里 只 第十五章中文.txt 19 14
900 ， 其余 都 是 雪白 的。 有的 是 标标准准 的 椭圆； 有的 两头 鼓 中间 第二十四章中文.txt 4 1
901 里 冷冷 地 看 他， 一 副” 无可奉告 ” 的 模样。 倪志伟 便 不 敢 第十七章中文.txt 16 89
902 ， 牙关 咬 起来， 一 副 为 捍卫真理 誓死拼斗 的 模样。 卉紫 说： “ 你 第十六章中文.txt 15 16
903 三分 瞧不起 对方 的 语气， 一 副 自命不凡 的 模样， 所以 他 在 班 上 第十一章中文.txt 20 15
904 ， 绝对 是 一 副 操心过度 的 模样。 谈 的 自然 第4章中文.txt 25 18
905 有些 得意。 倪志伟 左顾右盼 的， 一 副 眉飞色舞 的 模样。 金铃 就 不 服气， 第十八章中文.txt 12
20
906 知识， 羞 于 谈 钱， 对 那些 腰缠万贯 的 “ 款爷 ” 们 总是 抱 着 偏 第十六章中文.txt 15 5
907 材料 隔 成 的 小 单间， 光线 昏昏暗暗 的， 每 个 小 单间 里 都 第十七章中文.txt 16 83
908 魔圈。 每 星期 的 回 课 是 雷打不动 的。 每 次 回 课， 卉紫 比 第十七章中文.txt 16 42
909 ， 清 朗朗 的， 一串串 的， 溅 出 晶莹剔透 的 水花。 卉紫 心惊胆战 地 踮 第十八章中文.txt 12 27
910 的 表盘， 里面 还有 一 颗 可以 转来转去 的 流星 似的 小 红珠珠。 张灵 第十三章中文.txt 17 52
911 的、 涩涩 的， 和 馨兰 那 张 容光焕发 的 滋润 面孔 成 对比。 “ 好 第二十章中文.txt 7 35
912 搽 了 胭脂 口红 翘 着 嘴唇 走来走去 的 爱美 的 小 女孩子。 低 年 第二十五章中文.txt 5 42
913 天 哪！ 这 老太太 不 是 早先 赫赫有名 的 特级 教师 孙淑云 吗？ 去年 第二十四章中文.txt 4 42
914 学生 胡梅、 刘娅如、 倪志伟 …… 他们 就 是 讨人喜欢 的 猫！” 卉紫 一下子 愣 在 第十四章中文.txt 18
43
915 她 面前 打架 争 宠， 她 对 各种各样 的 猫 叫声 可 算是 熟悉 到 第十一章中文.txt 20 8
916 一下 一下 戳 着 鞋 盒子 里 酣睡不醒 的 猫， 觉得 这 猫 的 皮色 第十四章中文.txt 18 28
917 ？ 想当年， 学生 时代 的 赵卉紫 也 曾 雄心勃勃 的。 班 上 的 女生 曾经 讨论 第3章中文.txt 24
1
918 有 巴掌 那么 大， 结 的 桑果 黑紫黑紫 的， 甜 得 要命 ……” 金 第二十二章中文.txt 1 12
919 不 被 看重、 不 被 称赞 的 平淡无奇 的 生活， 其实 是 深深 刺伤 了 第十四章中文.txt 18 45
920 上 谈话。 张老师 是 新华 街 小学 为数极少 的 男老师 之一， 前年 才 从 师 第六章中文.txt 10 19
921 ， 两 个 孩子 还 有 一 场 生离死别 的 痛苦， 再 好 的 东西 吃 第九章中文.txt 9 32
922 干巴巴 的 寓言 故事 吗？” 金铃 用 悲天悯人 的 目光 看 着 他们： “ 你们 第十七章中文.txt 16 78
923 站 在 她们 面前。 裙子 有点 大， 松松垮垮 的， 看上去 杨小丽 像 个 少年老 第二十五章中文.txt 5 63
924 包 眼泪。 她 把 这 一 包 欲滴不滴 的 眼泪 努力 送 进 卉紫 的 第二十一章中文.txt 6 38

| | | | | | |
|---|---|---|---|---|---|
| 925 | ， 水往低处流 啊！ ” 脸上 不免 就 有 些 | 怅然若失 的 神气 。 傍晚 下班 ， 卉紫 | 第二十四章中文.txt | 4 | 7 |
| 926 | 凝固 在 脸上 ， 变成 了 一 种 | 似哭似笑 的 窘迫 。 金铃 不 想 笑 ， | 第二十五章中文.txt | 5 | 23 |
| 927 | ， 手里 拎 的 正是 那 袋 蚕茧 ， | 满脸欢喜 的 笑容 非常 尴尬 地 凝固 在 | 第二十四章中文.txt | 4 | 11 |
| 928 | 的 ， 心存戒备 的 ， | 幸灾乐祸 的 ， 等 着 看 笑话 的 ， 什么 | 第十一章中文.txt | 20 | 19 |
| 929 | 最后 才 出场 ， 期望 着 有 个 | 震撼人心 的 结局 ， 期望 在 孩子 和 家长 | 第十三章中文.txt | 17 | 4 |
| 930 | 垃圾车 ， 一点儿 也 不 像 人家 那些 | 精打细算 的 老太太 们 ， 废品 能 攒 得 | 第十章中文.txt | 21 | 10 |
| 931 | 腰 爬 上 摩托车 。 真 是 个 | 童心未泯 的 老太太 。 校长 、 邢老师 、 | 第二十六章中文.txt | 2 | 27 |
| 932 | 。 卉紫 惊讶 地 发现 那 是 个 | 白发苍苍 的 老太太 的 背影 ， 老人 因为 | 第二十四章中文.txt | 4 | 39 |
| 933 | 老太太 们 站 到 了 一起 。 一个 | 慈眉善目 的 老太太 站 得 无聊 ， 主动 找 | 第二十四章中文.txt | 4 | 21 |
| 934 | 两 只 眼睛 盯住 老师 都 是 | 一动不动 的 ， 老师 喂 多少 就 能 吃 | 第4章中文.txt | 25 | 38 |
| 935 | 书本 作业 ， 不 跟 妈妈 谈及 那些 | 令人头疼 的 考试 成绩 问题 ， 而且 还 可 | 第十二章中文.txt | 13 | 2 |
| 936 | 下来 的 日子 就 变 得 狰狞可怕： | 大大小小 的 考试 接踵而至 了 。 先 是 | 第十三章中文.txt | 17 | 1 |
| 937 | 现在 上学 读书 太 不 开心 了 ， | 没完没了 的 考试 ， 满 耳朵 的 分数 ， | 第十二章中文.txt | 13 | 22 |
| 938 | ， 显出 了 | 从未有过 的 肃穆 和 庄重 。 李小娟 的 | 第二十五章中文.txt | 5 | 12 |
| 939 | 来得及 点 了 个 头 ， 后面 响起 | 啪嗒啪嗒 的 脚步声 。 于胖儿 把 书包 的 | 第十八章中文.txt | 12 | 43 |
| 940 | 妈妈 带 她 去 吃 “ 肯德基 ” 时 | 笑容满面 的 脸 。 她 小心翼翼 朝 尚海伸 | 第六章中文.txt | 10 | 36 |
| 941 | 开 眼睛 ， 马 上 看到 一 张 | 圆圆胖胖 的 脸蛋 挤 在 门 和 门框 | 第十九章中文.txt | 14 | 28 |
| 942 | 。 “ 哎呀 ， 我 都 差点儿 忘 了 问 ， | 今天下午 的 自习课 ， 你们 是 怎么 安排 | 第十九章中文.txt | 14 | 32 |
| 943 | 的 礼品 玩具 ， 其中 有 一 排 | 金发碧眼 的 芭比 娃娃 。 金铃 趴 在 柜 | 第九章中文.txt | 9 | 41 |
| 944 | 咬住 嘴唇 ， 眼巴巴 地 看 着 那些 | 娇艳欲滴 的 花 ， 舍不得 走开 。 摊主 | 第十九章中文.txt | 14 | 23 |
| 945 | 做出 了 不 平凡 的 事情 ， 是 “ | 见义勇为 ” 的 英雄 ， 是 我们 大家 应该 | 第十一章中文.txt | 20 | 33 |
| 946 | 的 串串红 ， 淡 粉色 娇滴滴 的 凤仙花 ， | 探头探脑 的 菖蒲 ， 躲 在 草丛 中 窃窃娇 | 第二十二章中文.txt | 1 | 25 |
| 947 | 的 菖蒲 ， 躲 在 草丛 中 | 窃窃娇笑 的 蝴蝶兰 。 再 顺着 墙角 看 | 第二十二章中文.txt | 1 | 26 |
| 948 | 的 人 ， 特别 不能 容忍 学生 中 | 小偷小摸 的 行为 ， 马上 就 在 班 里 | 第十一章中文.txt | 20 | 38 |
| 949 | 买 回来 送 她 的 ， 大大 的 、 | 五颜六色 的 表盘 ， 里面 还有 一 颗 可以 | 第十三章中文.txt | 17 | 51 |
| 950 | 在 杨小丽 穿 的 是 一 条 | 白底紫花 的 裙子 ， 不 留意 的 话 不 | 第二十五章中文.txt | 5 | 50 |
| 951 | 人缘 并 不好 ， 是 个 高高在上 、 不 | 讨人喜欢 的 角色 。 等 男生 们 开心 过 | 第十一章中文.txt | 20 | 16 |
| 952 | 像 都 很 投入 ， 容 不 得 | 一丝一毫 的 败笔 和 失误 ， 跟 对待 作业 | 第五章中文.txt | 22 | 8 |
| 953 | 黄昏 的 时刻 ， 也 是 在 这 | 车水马龙 的 路边 ， 可 你 还 不 像 | 第十九章中文.txt | 14 | 55 |
| 954 | ， 眼角 里 忽然 瞥见 一个 胖乎乎 的 | 跳跳蹦蹦 的 身影 。 卉紫 赶快 抬头 ， | 第二十四章中文.txt | 4 | 9 |
| 955 | 。 俗话说 “ 身大力不亏 ” ， 金铃 长 着 一 副 | 人高马大 的 身材 ， 多多少少 总 还是 有 | 第十三章中文.txt | 17 | 8 |
| 956 | 发 试卷 的 窸窣声 ， 再就是 监考 老师 | 走来走去 的 轻响 。 金铃 牢记 老师 交待 | 第十八章中文.txt | 12 | 50 |

| | | | | | |
|---|---|---|---|---|---|
| 957 | ， 抱 到 马路 上 看 街景 ， | 南来北往 的 过路人 都 忍不住 凑 上来 逗 | 第八章中文.txt | 0 | 4 |
| 958 | 碰到 中意 的 题目 时 是 能够 | 超常发挥 的 。 这 篇 作文 的 题 | 第4章中文.txt | 25 | 27 |
| 959 | 上 不 可 ？ 你 看看 我们 国家 | 各行各业 的 这些 顶尖 人才 ， 科技 领域 | 第4章中文.txt | 25 | 11 |
| 960 | 加上 外公 外婆 ， 简直 是 一 支 | 浩浩荡荡 的 送考 队伍 。 有些 路 远 的 | 第二十六章中文.txt | 2 | 23 |
| 961 | 。 但是 外婆 有 一个 观点 是 金铃 | 大加赞赏 的 ， 那 就是 对 孩子 的 分数 | 第十二章中文.txt | 13 | 14 |
| 962 | 真 是 要 命 。 签字 简直 是 | 没完没了 的 酷刑 ， 过 了 一 回 接下来 | 第六章中文.txt | 10 | 1 |
| 963 | 是 抱怨 ， 言语 中 的 快乐 是 | 显而易见 的 。 金铃 在 一旁 听 着 ， | 第六章中文.txt | 10 | 46 |
| 964 | 没 必要 把 她 弄 得 整天 | 灰头土脸 的 。 ” 金铃 在 外面 偷听 到 | 第十章中文.txt | 21 | 45 |
| 965 | 。 星期天 ， 金铃 向 卉紫 要 了 坐 | 公共汽车 的 钱 ， 和 刘娅如 两 个 人 | 第十七章中文.txt | 16 | 79 |
| 966 | 。 ” 他 就 开始 发 考卷 。 考卷 是 | 地地道道 的 铅印卷 ， 这 就 使 本 次 | 第十八章中文.txt | 12 | 49 |
| 967 | 大 堆 人 拥 上来 问 金铃 | 各种各样 的 问题 。 “ 摄像机 镜头 对 | 第十七章中文.txt | 16 | 52 |
| 968 | 同学 面前 的 时候 ， 就 俨然 一个 | 珠光宝气 的 阔 太太 ， 出入 有 豪华 轿车 | 第4章中文.txt | 25 | 8 |
| 969 | ， 是 冲 不 破 这 城市 里 | 高楼林立 的 陷阱 吗 ？ 或者 把 路边 耷 | 第十九章中文.txt | 14 | 57 |
| 970 | 胳膊 高高 举 过 头顶 ， 心里 涌出 | 从未有过 的 骄傲 和 自豪 。 她 那天 高 | 第十六章中文.txt | 15 | 42 |
| 971 | 、 正直 、 宽容 、 大度 ， 又 有 一 颗 | 悲天悯人 的 高贵 心灵 ， 艺术 上 的 感觉 | 第十九章中文.txt | 14 | 66 |
| 972 | 的 金黄 的 鱼子 、 雪白 的 鱼油 、 | 花花绿绿 的 鱼肚肠 和 鲜红 的 心肝 ， 觉 | 第十五章中文.txt | 19 | 35 |
| 973 | 发 白 ， 他们 被 李林 这 句 | 惊世骇俗 的话 深深 地 震撼 了 、 惊 呆 | 第二十五章中文.txt | 5 | 22 |
| 974 | 得胜 回 朝 的 将军 ； 金铃 则 | 垂头丧气 ， 皱 着 鼻子 苦 着 脸 ， 像 | 第二十二章中文.txt | 1 | 33 |
| 975 | 庄严 的 中年人 ， 终年 紧 闭 嘴角 | 不苟言笑 ， 目光 偶尔 对 人 一 瞥 ， 尖 | 第十八章中文.txt | 12 | 48 |
| 976 | 减肥 ， 先 减 瘦 了 爸爸 妈妈 ， | 歪打正着 ， 省 得 以后 有 人 逼 我 | 第八章中文.txt | 0 | 33 |
| 977 | 进 了 自己 被窝 里 。 卉紫 真是 | 哭笑不得 ， 看看 两 个 孩子 呼噜 呼噜 正 | 第九章中文.txt | 9 | 26 |
| 978 | 两 分 。 ” 金铃 被 他 说 得 | 又羞又恼 ， 真 恨不得 揪 住 他 的 领子 | 第六章中文.txt | 10 | 41 |
| 979 | 不 错 ！ 娇 得 像 现在 的 | 独生子女 。 真 是 有 什么样 的 孩子 就 | 第二十二章中文.txt | 1 | 20 |
| 980 | ， 整 小时 地 坐 在 琴凳 上 | 一动不动 真 是 难 为 了 她 。 逃避 | 第十七章中文.txt | 16 | 36 |
| 981 | 不 会 是 你 吧 ？ ” 倪志伟 平时 | 神气活现 ， 真 碰 到 事情 马上 就 腿 | 第十一章中文.txt | 20 | 37 |
| 982 | 胶带纸 粘 上去 的 “ 赵卉紫 ” 3 个 字 | 妥妥帖帖 ， 真是 天衣无缝 。 那天 回家 | 第六章中文.txt | 10 | 42 |
| 983 | ， 一 看到 这些 复杂 的 数字 就 | 头昏脑涨 ， 眼冒金星 。 她 咽 一 口 唾 | 第十八章中文.txt | 12 | 51 |
| 984 | ， 慢 晃 着 ， 在 教室 半空 中 | 飞舞摇荡 。 眼尖 的 于胖儿 首先 叫 起来 | 第十八章中文.txt | 12 | 60 |
| 985 | 礼品 又 要 花钱 …… 哎呀 呀 ， 简直 | 没完没了 。 眼见 钱 像 流水 一样 淌 出去 | 第十章中文.txt | 21 | 37 |
| 986 | 书包 兜 着 一 大 抱 东西 ， | 嚎啕大哭 着 回家 。 后来 还是 卉紫 尝试 | 第十七章中文.txt | 16 | 4 |
| 987 | 死 ？ 分明 是 瞧不起 人 嘛 ！ 正 | 胡思乱想 着 ， 忽然 尚海 捅 她 的 胳膊 | 第十八章中文.txt | 12 | 21 |
| 988 | 叫 他 不 要 出声 。 “ 让 孩子 | 踏踏实实 睡 一 觉 吧 。 ” 她 用 哈气 | 第二十六章中文.txt | 2 | 18 |
| 989 | 大 公鸡 变成 金凤凰 ， 在 世界 上 | 展翅飞翔 ！ ” 瞧 ！ 我 爸爸 是 不 是 | 第十六章中文.txt | 15 | 12 |
| 990 | 老鼠 一 夜 之间 咬 了 个 | 七零八落 ， 破碎 的 纸片 撒 了 一 地 ， | 第十四章中文.txt | 18 | 18 |

| | | | | |
|---|---|---|---|---|
| 991 | 过去， 轻轻 在 床 边 坐下， 生怕 一不小心 碰 疼 了 老师。 邢老师 小声 | 第十九章中文.txt | 14 | 29 |
| 992 | 都 没 心思 做 生意 了 似的， 三五成群 站 着， 面色 严峻 地 议论 着 | 第九章中文.txt | 9 | 3 |
| 993 | 了 手 心里 回家， 一路 走 一路 东张西望， 竟 把 手里 的 橡皮 走 忘 | 第1章中文.txt | 23 | 26 |
| 994 | 得 直 不 起 腰 来。 奶奶 莫名其妙： “ 笑 什么 呀？ 送 猪脑子 | 第二十六章中文.txt | 2 | 14 |
| 995 | 猪脑子 来 了！ ” 金铃 就 和 卉紫 相视而笑， 笑 得 直 不 起 腰 来。 | 第二十六章中文.txt | 2 | 13 |
| 996 | ， 不 那么 光明正大。 别人 上课 时 东倒西歪 笑 成 一 团 时， 她 绷 | 第十九章中文.txt | 14 | 16 |
| 997 | 直 不 起 腰。 金铃 也 笑。 一老一少 笑 成 了 一 团。 “ 还有 呢 | 第二十二章中文.txt | 1 | 47 |
| 998 | 小 石头 雕像 举 在 邢老师 面前 晃荡晃荡， 笑嘻嘻 地 问： " 老师 你 | 第二章中文.txt | 8 | 6 |
| 999 | 的 一 声 惊讶 或者 赞叹。 她 挨家挨户 笑眯眯 解释： “ 这 是 我 爸爸 | 第十五章中文.txt | 19 | 41 |
| 1000 | ！ ” 卉紫 当着 一 街 人 笑 得 前仰后合。 第二 天 到 学校， 金铃 一 | 第十七章中文.txt | 16 | 51 |
| 1001 | 会 嚎啕大哭。 第二 天 她 提 早 10 分钟 | 第六章中文.txt | 10 | 8 |
| 1002 | 。 谁 料到 快乐 就 像 肥皂泡 一样 转瞬即逝， 第二 天 数学 卷子 发 下来 的 | 第六章中文.txt | 10 | 16 |
| 1003 | 话： “ 笑 什么？ ‘ 士 别 三日， 当 刮目相看 ’。 等 于胖儿 以后 真 的 有 | 第二十五章中文.txt | 5 | 33 |
| 1004 | 的 牛肉 味。 她 很 精， 先 不动声色， 等 出 了 书房 门 后 又 | 第八章中文.txt | 0 | 35 |
| 1005 | 的 脑袋 挤 在 一 处， 都 心急火燎 等 着 看 奇迹。 奇迹 却 没有 | 第二十二章中文.txt | 1 | 17 |
| 1006 | 上， 一 动 都 不 敢 动。 好不容易 等 脚步声 走 过去 了， 金铃 慌 | 第十八章中文.txt | 12 | 58 |
| 1007 | 。 转念 一 想 又 不 对： 为 “ 希望工程 ” 筹钱 才 能 募捐， 要不 就 | 第十三章中文.txt | 17 | 49 |
| 1008 | 有 一 次 扬眉吐气 签字 的 机会， 还 活生生 让 老 | 第六章中文.txt | 10 | 45 |
| 1009 | 分数 的， 她 一 看 就 头疼， 稀里糊涂 算 下来， 10 题 起码 错 6 | 第1章中文.txt | 23 | 11 |
| 1010 | 送 进 卉紫 的 视线 里。 卉紫 不为所动： “ 算 了， 别 对 我 来 这 | 第二十一章中文.txt | 6 | 39 |
| 1011 | 里 穿过， 金铃 的 理由 非常 充足。 管天管地， 管不住人拉屎撒尿， 妈妈 总 不 | 第二十二章中文.txt | 1 | 3 |
| 1012 | 本市 新华街 小学 读 六 年级。 身高 1.55 米， 体重 50 公斤， 标准 的 | 第1章中文.txt | 23 | 1 |
| 1013 | 那么 多 孩子 的？ 简直 就 是 流水作业 线。” | 第二十章中文.txt | 7 | 48 |
| 1014 | 的 节目 才 能 又 好玩 又 出奇制胜？ 组 里 讨论 了 一个 中午， | 第十一章中文.txt | 20 | 9 |
| 1015 | ： 一个 数 是 由 2 个 10 和 5 个 0.01 组成 的， 这个 数 是 （ ） | 第十八章中文.txt | 12 | 54 |
| 1016 | 笨！ 既 是 邻居， 早 就 该 想方设法 结 上 关系 了！ ” 金亦鸣 替 | 第二十章中文.txt | 7 | 21 |
| 1017 | 发挥 她 的 语言 才能 和 想像力， 添油加醋， 绘声绘色， 说 得 邢老师 笑眯 | 第十九章中文.txt | 14 | 31 |
| 1018 | 也 不 跟 你们 算 了， 你们 痛痛快快 给 个 大头 就 成。” 听 她 | 第十三章中文.txt | 17 | 32 |
| 1019 | 一 层 浅浅的 灰白， 眼角 的 皱纹 密密麻麻， 绝对 是 一 副 操心过度 的 模 | 第4章中文.txt | 25 | 17 |
| 1020 | 啊！ 第二 天 风和日丽， 老天爷 仿佛 故意 要 送给 大家 | 第十八章中文.txt | 12 | 38 |
| 1021 | 把 倪志伟 推 了 出来。 倪志伟 同样 一脸无辜： “ 老师 没 宣布 考试 不 准 | 第十八章中文.txt | 12 | 73 |
| 1022 | 学 文科 的， 可 中学 数学 一直 出类拔萃， 考 大学 就 因为 数学 比 别人 | 第1章中文.txt | 23 | 12 |
| 1023 | 二十二 种瓜得豆， 而且 是 颗 金 豆 金铃 很 | 第二十二章中文.txt | 1 | 1 |

| | | | | | |
|---|---|---|---|---|---|
| 1024 | 开始 “ 污肚 ”， 很快 将 所有 的 储存 | 倾吐一空， 而后 颇为 空落 地 等待 来 年 | 第十五章中文.txt | 19 | 5 |
| 1025 | 不 懂 你 不 懂！” 忽然 她 | 嚎啕大哭， 肩膀 一抽一抽， 泪水 糊 得 | 第二十五章中文.txt | 5 | 70 |
| 1026 | 不 懂得 美！ 杨贵妃 胖 不 胖？ | 蒙娜丽莎 胖 不 胖？ 还有 美神 维纳斯， | 第八章中文.txt | 0 | 10 |
| 1027 | 猜猜 是 谁 得了 100 分。 大家 就 | 乱猜一气： 胡梅、 刘娅如、 倪志伟 … | 第十八章中文.txt | 12 | 30 |
| 1028 | 工程， 每次 卉紫 都 要 累 得 | 头昏眼花、 胳膊 发 沉。 金铃 一 到 | 第十六章中文.txt | 15 | 4 |
| 1029 | 、 一 套 泳装、 一 套 冬装， 可以 | 换来换去。 胳膊 腿 也 是 软 的， 让 | 第九章中文.txt | 9 | 46 |
| 1030 | 想 不 明白， 你 的 考试 成绩 | 忽高忽低 能 相差 这么 多。 看 你 学习 | 第二十三章中文.txt | 3 | 7 |
| 1031 | 不灵便 的 孙 老太太。 一时间 她 竟 | 目瞪口呆， 脑子 有点 转 不 过来。 孙 | 第二十六章中文.txt | 2 | 26 |
| 1032 | 什么， 金铃 笑 得 仰 起 了 | 圆鼓鼓的 脸。 她 几乎 是 跳 着 蹦 | 第二十四章中文.txt | 4 | 35 |
| 1033 | 住 了， 眼睛 睁 得 老大， 嘴巴 | 半张不张， 脸颊 肌肉 微微 有些 抽动。 | 第二十一章中文.txt | 6 | 14 |
| 1034 | 不 记得 请 妈妈 缝 上？” 金铃 | 支支吾吾， 自己 也 不 知道 自己 答 了 | 第二十一章中文.txt | 6 | 22 |
| 1035 | 的 炒面 香味 弥漫 开 来。 金铃 | 迫不及待 舀 一 勺 进口， 却 立刻 “ 呸 | 第八章中文.txt | 0 | 25 |
| 1036 | ， 马上 对 妈妈 郑重 宣布： 坚决 杜绝 | 大手大脚 花钱 的 现象， 除了 米面 油盐 | 第十章中文.txt | 21 | 25 |
| 1037 | 提高 节约 观念， 以后 不 会 再 | 大手大脚 花钱， 这 也 是 你 希望 我 | 第十章中文.txt | 21 | 14 |
| 1038 | 。 杨小丽 对 她 的 行为 | 哭笑不得， 苦 了 脸 求 她：“ 金铃 | 第二十五章中文.txt | 5 | 67 |
| 1039 | 对 金亦鸣 说：“ 妈妈 今天 有 没有 | 吃错什么 药？” 金亦鸣 责备 她：“ | 第二十四章中文.txt | 4 | 48 |
| 1040 | ， 有 几 样 还是 可以 的， 比如 “ | 仰卧起坐 ”， 虽然 起 得 吃力 了 些， | 第十三章中文.txt | 17 | 7 |
| 1041 | 生命 更 重要。” 卉紫 笑话 他：“ 简直 | 对牛弹琴！ 蚕 儿 能 有 人 的 思维？ | 第二十二章中文.txt | 1 | 21 |
| 1042 | 命运 就 是 南辕北辙 了 呢！” 金亦鸣 | 举手投降：“ 行 了 行 了， 你 别 再 | 第六章中文.txt | 10 | 60 |
| 1043 | 了 看， 一下子 放心 了： 是 个 | 白发苍苍 行动 不便 的 老太太， 胳膊 下 | 第二十二章中文.txt | 1 | 30 |
| 1044 | 齐 腰 “ 哗啦 ” 一 撕， 罚 学生 | 从头到尾 补 做 一 遍。 还有 一 次 | 第六章中文.txt | 10 | 22 |
| 1045 | 的 角色， 金铃 在 元旦 庆祝会 上 | 认认真真 表演 了， 逗 得 全班 同学 哈哈 | 第十一章中文.txt | 20 | 42 |
| 1046 | ：“ 如果 你 是 说 着 笑 着 | 不知不觉 被 他们 拐走 的 呢？ 如果 他们 | 第1章中文.txt | 23 | 5 |
| 1047 | 去 玩？” 金铃 低头 剥 着 指甲， | 一声不响。 被 外婆 问 得 急 了， 她 | 第十四章中文.txt | 18 | 6 |
| 1048 | 妈妈 买 来 的 这些 习题册， 金铃 | 完完全全 被 淹没 在 题海 中。 每 天 | 第十四章中文.txt | 18 | 9 |
| 1049 | 起来 是 开窍 了， 知道 用功 了， | 一心一意 要 做 好 孩子 了。 妈妈 等 | 第六章中文.txt | 10 | 14 |
| 1050 | 的 语文 老师 是 市 优秀 教师， | 三天两头 要 在 班 上 开 “ 公开 课 ” | 第1章中文.txt | 23 | 22 |
| 1051 | 跟 着 她 就 跑， 一 副 | 见义勇为 要 帮 她 追回 钱包 的 架势。 | 第二十四章中文.txt | 4 | 37 |
| 1052 | 答应 着， 心里 很 感激。 她 想， | 无论如何 要 把 金铃 的 学习 成绩 提 | 第十三章中文.txt | 17 | 39 |
| 1053 | 数学 总是 考 不 到 优秀。 卉紫 | 下定决心 要 采取 非常 手段， 务必 在 女 | 第八章中文.txt | 0 | 12 |
| 1054 | 天 尚海 妈妈 就 找 了 邢老师， | 直截了当 要求 参加 补课。 邢老师 自然 是 | 第五章中文.txt | 22 | 3 |
| 1055 | 谁！ 别 总 以为 自己 成绩 好， | 高人一头， 见 了 别人 得奖 心里 就 妒忌 | 第十七章中文.txt | 16 | 55 |
| 1056 | 上 都 接受 些 什么 呀！ 宣传 | 一心助人、 见义勇为、 发奋图强 的 事迹 | 第十章中文.txt | 21 | 1 |

| | | | | | |
|---|---|---|---|---|---|
| 1057 | 次 金铃 对 眼镜 叔叔 佩服 得 | 五体投地， 觉得 他 比 电视 上 的 美国 | 第九章中文.txt | 9 | 7 |
| 1058 | 。” 话说 出去， 倪志伟 才 意识 到 自己 | 一不小心 触犯 了 众怒， 因为 动画片 是 | 第十六章中文.txt | 15 | 28 |
| 1059 | 影子！ 原来 小 东西 用 的 是 “ | 金蝉脱壳” 计。 卉紫 心里 一时 又 好 | 第二十四章中文.txt | 4 | 12 |
| 1060 | 好。 金铃 听见 屋里 的 大人 在 | 七嘴八舌 讨论 什么， 听 了 半 天 才 | 第九章中文.txt | 9 | 9 |
| 1061 | 太 短 了， 要是 路 长 得 | 没完没了 该 多 好 啊， 她 可以 永远 | 第六章中文.txt | 10 | 2 |
| 1062 | 不 能 成 眠， 而 后 就 | 苦思冥想 该 怎么 帮帮 金铃， 而 后 就 | 第十二章中文.txt | 13 | 29 |
| 1063 | 跑 回 家， 气 得 赵卉紫 | 翻着白眼 说 不 出 一 句 话。 | 第3章中文.txt | 24 | 5 |
| 1064 | 回家 后， 金铃 照例 又 跟 妈妈 | 絮絮叨叨 说 些 学校 里 的 事。 说 | 第二章中文.txt | 8 | 23 |
| 1065 | 并 不 想 无偿奉献。 金铃 有些 不好意思， | 支支吾吾 说： “其实 也 没什么…… | 第十章中文.txt | 21 | 17 |
| 1066 | 一 声， 用 唱歌 一样 的 声音 | 拖长腔调 说： “我 -- 也 -- 喜 - | 第二十二章中文.txt | 1 | 52 |
| 1067 | 花 200 块 钱 买 的 呀！” 金铃 | 一字一句 说： “我 只是 想 要 帮 你 | 第十三章中文.txt | 17 | 56 |
| 1068 | 你 所 说 的 任何 话。” 金铃 | 哭丧着脸 说： “我 怕 你 知道 了 会 | 第二十三章中文.txt | 3 | 6 |
| 1069 | ， 卉紫 白 用 了 半天 劲。 金铃 | 自告奋勇 说： “我 来 吧！” 当然 | 第十五章中文.txt | 19 | 29 |
| 1070 | 站 起来， 两手 死命 捂住 裤裆， 一边 | 哭丧着脸 说： “我 要 上 厕所！ 我 | 第十八章中文.txt | 12 | 8 |
| 1071 | 一 揪， 情绪 马上 就 松弛 了， | 龇牙一笑 说： “是 不 一样。” 医 | 第七章中文.txt | 11 | 28 |
| 1072 | 身上， 哪里 还 肯 放下 呢？ 嘴里 | 不好意思 说 要， 眼睛 里 却 是 热切 | 第十七章中文.txt | 16 | 9 |
| 1073 | 神 来。 金铃 蹭 到 卉紫 身边， | 哭丧着脸 说： “都 怪 我 跌 了 跟头 | 第十三章中文.txt | 17 | 33 |
| 1074 | ； 外国语 学校 的 卷子 常常 出 得 | 刁钻古怪， 说不定 倒 能 对 了 她 的 | 第4章中文.txt | 25 | 41 |
| 1075 | 什么， 所以 才 赶来 帮忙， 帮 她 | 解脱困境。 说不定 它 就 是 动画片 里 | 第十四章中文.txt | 18 | 23 |
| 1076 | 出 一个 香喷喷 的 煎饼 夹 油条， | 装模作样 读 几 句 书， 俯 下 头 | 第十九章中文.txt | 14 | 6 |
| 1077 | 丈夫 谁 当 谁 的 底色 呢？ | 也就是说， 谁 退 居 家庭 操持 家务 管理 | 第3章中文.txt | 24 | 2 |
| 1078 | 听 着 前后 左右 家长 们 的 | 窃窃私语： 谈论 自己 孩子 的 分数， 预 | 第二十章中文.txt | 7 | 12 |
| 1079 | 脸 愁苦： “是 啊， 我 怎么 就 | 一不小心 赚 了 大钱 呢？ 弄 得 秘书 | 第二十三章中文.txt | 3 | 17 |
| 1080 | ， 才 开始 有 六 年级 的 学生 | 陆陆续续 走 出 校门。 他们 没有 整 队 | 第二十四章中文.txt | 4 | 28 |
| 1081 | 的 重点 中学 校长， 哪个 不 是 | 身价百倍？ 走 出去 比 大学 校长 都 风光 | 第二十章中文.txt | 7 | 22 |
| 1082 | 胖 孩子 和 憨 憨 的 熊猫、 | 摇摇摆摆 走路 的 企鹅 及 爱 睡觉 的 | 第七章中文.txt | 11 | 35 |
| 1083 | 到 学校 去 过？ 老师 都 喜欢 | 大惊小怪。” 赵卉紫 一个劲 地 说： | 第六章中文.txt | 10 | 48 |
| 1084 | 闹 个 准确 结论， 以后 也 好 | 对症下药。 赵卉紫 不 知道 “学习 障碍 | 第七章中文.txt | 11 | 9 |
| 1085 | ， 好好 一道 题目 就 这么 错 得 | 不明不白。 赵卉紫 打 定 主意 要 带 金 | 第七章中文.txt | 11 | 7 |
| 1086 | 听 一 盘 外语 磁带， 嘴里 还 | 念念有词。 赵卉紫 用劲 推开 门， 故意 | 第六章中文.txt | 10 | 57 |
| 1087 | 一 堆 人， 不断 地 有 人 | 匆匆忙忙 赶 过来 挤 进去， 又 不断 地 | 第九章中文.txt | 9 | 5 |
| 1088 | 到 10 分钟， 奶奶 已经 握 着 斧子 | 气喘吁吁 赶到。 奶奶 说： “真是 好 | 第十五章中文.txt | 19 | 32 |

| | | | | | |
|---|---|---|---|---|---|
| 1089 | 的 望 着 房顶， 过 一会儿 忽然 | 哈哈大笑 起来。 卉紫 在 外面 听见 金铃 | 第十八章中文.txt | 12 | 26 |
| 1090 | 了 没有 多 长 时间， 又 重新 | 趾高气扬 起来， 因为 他 参加 区 里 的 | 第十一章中文.txt | 20 | 39 |
| 1091 | 看 了 第一 行 字， 马上 又 | 头晕目眩 起来， 连忙 把 本子 合 上。 | 第十九章中文.txt | 14 | 53 |
| 1092 | 组 同学 目光 的 逼视 下， 只得 | 轻手轻脚 趴 到 金铃 的 背 上， 当 | 第十一章中文.txt | 20 | 24 |
| 1093 | 决定 向 夫人 汇报 了 再说。 他 | 两手空空 跑 回 家， 气 得 赵卉紫 翻着白 | 第3章中文.txt | 24 | 4 |
| 1094 | ？ ” 金亦鸣 说： “ 怎么 不 会 这样 呢？ | 3923 24 跟 40 相差 多少？ 1 | 第六章中文.txt | 10 | 66 |
| 1095 | ， 仍然 会 | 心甘情愿 跟着 广告 走， 不撞南墙不回头。 | 第八章中文.txt | 0 | 17 |
| 1096 | 还是 那么 要 强。” 两 个 人 | 一前一后 跨 进 门 去。 “ 梦娜 ” 的 | 第二十章中文.txt | 7 | 36 |
| 1097 | “ 50 米 跑 ” 的 水平， 书包 在 背后 | 哗啦哗啦 跳 得 像 炒 豆子。 路 上 | 第十三章中文.txt | 17 | 43 |
| 1098 | ， 要 允许 保密！ " 写 完 信， 又 | 涎着脸皮 蹭 过来， 求 妈妈 替 她 写 | 第二章中文.txt | 8 | 10 |
| 1099 | 皮肤 很 白， 长 得 也 是 | 眉清目秀， 身材 高高的， 腿 长长的。 | 第十一章中文.txt | 20 | 12 |
| 1100 | 金铃 的 家 了， 就 好像 它 | 那天晚上 躲 在 黑暗 处 听 懂 了 | 第十四章中文.txt | 18 | 49 |
| 1101 | 很 聪明 的 孩子。 " 金铃 一 愣， | 迟迟疑疑 转 过 身 问 老师： " 你 是 | 第二章中文.txt | 8 | 21 |
| 1102 | 上 不足 比 下 有余， 活 得 | 快快乐乐 轻轻松松， 叫 人 气 也 不 是 | 第1章中文.txt | 23 | 16 |
| 1103 | 最 喜欢 老鼠？ ” 卉紫 嘀咕 一 声： | “ 莫名其妙。 ” 过 了 一会儿， 金铃 终 | 第十四章中文.txt | 18 | 34 |
| 1104 | 些， 改 成 一个 “ 7 ” 字， 报名 时 | 鱼目混珠 过 了 关。 可是 金铃 妈妈 后 | 第1章中文.txt | 23 | 19 |
| 1105 | 碰到 了 主编 余老太， 她 是 挤 | 公共汽车 过来 的。 大约 因为 个子 矮 | 第二十章中文.txt | 7 | 25 |
| 1106 | | 活活闷死， 还 仿照 游泳 时 潜水 的 做法 | 第二十一章中文.txt | 6 | 35 |
| 1107 | 话头 一 转， “ 有 件 事， 我 | 想来想去 还 是 推荐 你。 ” 金铃 听 | 第十七章中文.txt | 16 | 57 |
| 1108 | ， 除了 按 月 准时 开 工资 外， | 或多或少 还 能 发 些 奖金， 逢年过节 都 | 第3章中文.txt | 24 | 9 |
| 1109 | ”， 卉紫 自己 也 害怕 人家 | 胡搅蛮缠， 还是 先 送 了 钱 拉倒。 | 第十三章中文.txt | 17 | 25 |
| 1110 | 不足 半 个 小时。 原来， 只要 她 | 全神贯注， 还是 可以 把 作业 完成 得 快 | 第十九章中文.txt | 14 | 46 |
| 1111 | 带 出 去 见 见世面。 外婆 说： “ | 治国之道 还有 个 一张一弛 呢， 孩子 苦学 | 第十四章中文.txt | 18 | 2 |
| 1112 | ， 外国语 学校 简直 比 北大 清华 剑桥 | 牛津哈佛 还要 神气百倍。 卉紫 在 心里 恨 | 第4章中文.txt | 25 | 21 |
| 1113 | 远， 要 坐 三 个 小时 的 | 长途汽车， 还要 过 一 条 轮渡 … … ” | 第二十二章中文.txt | 1 | 14 |
| 1114 | 。 | 总的说来， 这 一 家 的 经济 情况 是 | 第3章中文.txt | 24 | 13 |
| 1115 | ， 你 懂 不 懂？ ” 金铃 被 妈妈 | 劈头盖脸 这 一 顿 骂， 委屈 得 流出 | 第八章中文.txt | 0 | 28 |
| 1116 | 踩 车， 两 位 老人 配合 得 | 得心应手。 这 以后， 他们 就 骑 着 | 第十二章中文.txt | 13 | 21 |
| 1117 | 金铃 的 胳膊。 金铃 趁 这 机会 | 拔腿就跑。 这 回 她 是 真正 跑 出 | 第十三章中文.txt | 17 | 42 |
| 1118 | 是 我 呀！ 金铃 同学 嘛！ ” 卉紫 | 哭笑不得。 这 孩子 一个 人 躺 在 床 | 第十八章中文.txt | 12 | 31 |
| 1119 | 写 这些 文章 的 人 总 喜欢 | 危言耸听， 这 跟 作家 们 对 生活 素材 | 第七章中文.txt | 11 | 36 |
| 1120 | 似的， 跑 到 班主任 邢老师 办公室 里 | 大喊大叫： “ 这 还 了 得！ 和 苏美 | 第六章中文.txt | 10 | 47 |
| 1121 | 把 金铃 叫 过 来 询问。 金铃 | 一口咬定 这些 蚕 是 自己 捡 的。 “ | 第二十一章中文.txt | 6 | 37 |
| 1122 | ， 还 是 金铃 自己 太 不 用功， | 得过且过。 这样 的 孩子 真是 烂泥 巴 | 第1章中文.txt | 23 | 13 |

1123 数： 39 和 23 24 。 结果 式子 就 变成 这样： 48 ×39+48×23 24 。 这样 算 起来 第六章中文.txt 10
70
1124 原谅。 好 的 是 数学 成绩 一直 平稳上升， 进入 了 班 上 前 10 名。 第二十四章中文.txt 4 61
1125 发生 了 何事， 依旧 在 努力 地 爬来爬去， 连 带 着 整 只 口袋 都 第二十一章中文.txt 6 21
1126 嗓门 喊： “ 金铃 你 又 干什么？ ” 金铃 灵机一动， 连忙 回答： “ 我 上 厕所 第二十二章中文.txt 1
2
1127 进 位， 又 错 一 道。 张老师 哭丧着脸， 连连 摆手： “ 罢了 罢了， 第六章中文.txt 10 30
1128 ， 金铃 终于 还是 不 忍心 看 妈妈 气喘吁吁 追 猫 的 样子， 慢吞吞 上前 帮 第十四章中文.txt 18 35
1129 桌上。 李小娟 慌忙 拿 着 粉笔 下位， 恭恭敬敬 送 回 到 讲台。 尚海 又 惊 第二十一章中文.txt 6 28
1130 惯性， 那 就 像 火箭 已经 把 人造卫星 送 进 了 轨道 一样， 你 让 第八章中文.txt 0 6
1131 笔 钱 便 存 下来， 在 金铃 周岁那天 送 进 长江 百货公司， 拖 回家 第十七章中文.txt 16 32
1132 ， 心跳 如 鼓， 拔腿就逃。 逃 到 厕所 时， 忽然 里面 第十七章中文.txt 16 87
1133 实际 地 问。 卉紫 想 了 想， 咬牙切齿 道： “ 交 钱！ ” 第4章中文.txt 25 13
1134 骚扰， 骚扰！ ” 卉紫 一 屁股 坐 下来， 自言自语 道： “ 看 起来 不 给 是 不行 第十三章中文.txt 17
46
1135 舌头， 慌忙 转 过 身 坐 得 端端正正。 邢老师 伸出 一 根 手指， 第二十五章中文.txt 5 6
1136 把 老师 们 说 得 一个 个 毛骨悚然。 邢老师 和 数学 张老师 联合 第十八章中文.txt 12 71
1137 。 因为 他 聪明， 成绩 好， 又 口齿伶俐 能言善辩， 邢老师 就 让 他 当 了 副班长 第十一章中文.txt 20
13
1138 小 红萝卜， 谁 看 着 都 觉得 心惊胆战。 邢老师 急急忙忙 地 赶 了 过 第二十五章中文.txt 5 53
1139 什么样 的 家教 才 合适 呢？ ” 卉紫 虚心讨教。 邢老师 笑笑： “ 这 不 容 第二十章中文.txt 7 18
1140 ， 痴痴 地 望 着 草地 … … 忽然， 我 眼睛一亮！ 那、 那 不 是 刚刚 抽 嫩芽 第4章中文.txt 25 29
1141 作 这样 的 允诺， 一时间 高兴 得 语无伦次： “ 那么 … … 那么 … … 补 第4章中文.txt 25 40
1142 ， 真是 天衣无缝。 那天 回家 后 妈妈 问 金铃 第六章中文.txt 10 43
1143 杨小丽。 半 天 没 见面， 她 仿佛 久别重逢 那样 的 兴奋， 高声 喊 着 他们 第二十六章中文.txt 2 25
1144 面前 就 变 得 像 个 刺猬， 随时随地 都 会 把 满身 硬刺 竖起 来， 第六章中文.txt 10 5
1145 ， 想 找 个 外语 学校 的 家教， 钻壁打洞 都 找 不 着。 馨兰 忽然 叫 第二十章中文.txt 7 41
1146 接近 于 沸腾。 外国语 学校， 外国语 学校！ 口口声声 都 是 外国语 学校， 简直 像 强 第4章中文.txt
25 20
1147 。 杨小丽 脸色 煞白， 因为 惊慌 和 害怕， 满头满脸 都 是 汗水。 邢老师 安慰 她 第二十五章中文.txt 5
59
1148 还 能 发 些 奖金， 逢年过节 都 有 实物 发放， 算是 过得去 第3章中文.txt 24 10
1149 个 读 文科 的 料， 怎么 就 随时随地 都 有 这些 感慨 生 出 来？ 第二十五章中文.txt 5 77
1150 不好？ 发展 了 生产， 搞活 了 经济， 于国于民 都 有利。 就是 要 鼓励 孩子 第十章中文.txt 21 3

1151 似的， 把 厨房 里 瓶瓶罐罐 都 移 开， 到处 搜寻 老鼠 留下 第十四章中文.txt 18 13
1152 天 到 学校， 金铃 发现 全班 同学 不约而同 都 穿 上 了 自己 最 好 第二十五章中文.txt 5 17
1153 遍 又 一 遍， 连 每 一个 标点符号 都 背 出来 了。 然后 她 把 第二章中文.txt 8 13
1154 妈妈 真诚 的 道歉， 心软 的 金铃 无论如何 都 要 给予 原谅。 好 的 是 第二十四章中文.txt 4 60
1155 金铃 最 崇拜 的 影星， 他 的 一招一式 都 那么 逗人， 金铃 不时 发出 第十四章中文.txt 18 24
1156 就 回到 家， 吊 着 胳膊 在 大街小巷 里 东游西荡。 他 对 于胖儿 说 第十三章中文.txt 17 57
1157 ！” 卉紫 和 金亦鸣 紧急 行动 起来， 在 角角落落 里 寻找 老鼠洞。 卉紫 奇怪 的 第十四章中文.txt 18 14
1158 路边 污泥 糟蹋 得 不 成 样子， 自动铅笔 里 的 铅 统统 断 了， 钢笔 第十七章中文.txt 16 3
1159 了 吗？” 金铃 回答： “我 尽量 吧。” 不一会儿， 金亦鸣 下班 回家。 卉紫 把 第十三章中文.txt 17 23
1160 卖弄 新 学 的 成语： “这 叫 ‘ 牛刀小试’！” 金亦鸣 刚 摘 下 耳机 第十七章中文.txt 16 21
1161 上 前 抓住 了 爸爸 的 手。 人赃俱获， 金亦鸣 只好 跟 女儿 分享 美味 第八章中文.txt 0 36
1162 呢？” “ 砸锅卖铁！” 金亦鸣 叹 了 一 第4章中文.txt 25 15
1163 药 吃 错 了 会 产生 幻觉， 行为古怪。” 金亦鸣 扑哧 笑 出来， 第二十四章中文.txt 4 49
1164 洞 的 本领？ 寻找 的 结果 是 一无所获。 金亦鸣 把 睡觉 的 大床 都 掀 第十四章中文.txt 18 15
1165 。 否则 只好 对不起 了。” 金铃 跳 起来： “ 一言为定？” 金亦鸣 说： “一言为定 第九章中文.txt 9 20
1166 朗读 一 遍 后， 金亦鸣 和 卉紫 面面相觑。 金亦鸣 说： “这 有 什么 第十七章中文.txt 16 77
1167 ？” 金亦鸣 说： “ 一言为定。” 金铃 不 放心， 跟 爸爸 第九章中文.txt 9 21
1168 吃 了 苍蝇 一样 难过。 她 想 无论如何 金铃 不 该 去 那个 倒霉 的 第七章中文.txt 11 11
1169 。 因为 不 到 上学 时间， 校园 里 冷冷清清。 金铃 不好意思 将 花束 举 在 第十九章中文.txt 14 24
1170 解释： “这 是 我 爸爸 的 大鱼。” 当天晚上 金铃 做 了 个 梦， 梦 到 第十五章中文.txt 19 42
1171 一个 准。 比如 有 这么 一 条： 48×（ 40-1 24） 。 金铃 先 把 第六章中文.txt 10 62
1172 5 个 不同 的 答案， 简直 就 是 一塌糊涂。 金铃 冲 到 厨房 里， 对 第十四章中文.txt 18 40
1173 很 满意， 认为 金铃 睡 得 不错， 精力充沛。 金铃 吃 过 外婆 送来 的 糕 第二十六章中文.txt 2 19
1174 多少 火， 生 了 多少 气， 已经 难以计数。 金铃 呢， 原本 活泼 的 天 第十七章中文.txt 16 46
1175 ” 过来。 马路 上 车 鸣 人 叫， 热闹非凡。 金铃 在 人群 中 一 眼 看见 第二十六章中文.txt 2 24
1176 ， 桌上 意外 地 摆 了 一 碗 红烧排骨。 金铃 大概 是 饿 得 慌 了， 第十章中文.txt 21 33
1177 响 了， 老师 挥 挥手， 表示 谈话 到此为止。 金铃 如释重负， 拔腿 就 想 第二章中文.txt 8 19
1178 自己 也 有 一 份 责任， 心里 不是滋味。 金铃 对 杨小丽 说： “我 第十九章中文.txt 14 18
1179 去 金铃 外婆 家。 结果 一 上 公共汽车， 金铃 就 大 叫： “妈妈 你 第七章中文.txt 11 17
1180 色狼！” 金亦鸣 在 旁边 早已 笑 得 前仰后合。 金铃 干脆 拒绝 了 爸爸 妈妈 第十七章中文.txt 16 75
1181 过来 面向 正前方， 面孔 微微 带笑， 双目 炯炯有神。 金铃 平常 是 个 随意 惯 了 第十七章中文.txt 16

61
1182 课程 加深， 满 纸 的 小 蝌蚪 密密麻麻， 金铃 年龄 小， 认 读 能力 第十七章中文.txt 16 37
1183 题库 啦， 英语 初级 辅导 教程 啦， 五花八门。 金铃 很 冷静 地 翻 着 这些 第十四章中文.txt 18 7
1184 太 美， 等 一会儿 做 习题 又 错误百出。” 金铃 很 有 把握 地 说 第二十三章中文.txt 3 19
1185 ， 热闹非凡。 金铃 想： 可 真 是 的， 第二十五章中文.txt 5 10
1186 ， 你 这个 坏东西！ 又 在 我 跟前 先斩后奏。” 金铃 把 一 根 食指 竖 第九章中文.txt 9 34
1187 地 问：“是 吗？ 让 我 看看， 可不可以？” 金铃 抽出 自己 的 作文本 第十九章中文.txt 14 52
1188 ， 两边 都 没 有 金铃 的 名字。 这就是说， 金铃 既 不 是 最 好， 也 第4章中文.txt 25 24
1189 出 钱 嘛！” 金铃 却 是 摇头， 死活不肯。 金铃 有 一 块 很 漂亮 的 第十三章中文.txt 17 50
1190 委员 对 了 得 数。 结果 真是 出人意外， 金铃 每 一 题 都 对 了。 第六章中文.txt 10 12
1191 ， 有 好几 天 都 闷闷不乐。 金铃 的 钢笔字 一向 非常 糟 第二章中文.txt 8 15
1192 无 一 不 起 了 一 身 鸡皮疙瘩。 金铃 看 不 过 了， 奋勇 第十一章中文.txt 20 21
1193 ， 逗 那个 笑 几 声， 忙 得 不亦乐乎。 金铃 站 在 教室 门口 定 一 第十九章中文.txt 14 35
1194 嘴 望 着 金铃， 一时 真 有点 哭笑不得。 金铃 自顾自 地 站起身 来， 第二十一章中文.txt 6 29
1195 个 数， 很 快 得 出 数字： 1918。 金铃 莫名其妙 地 看 着 爸爸 第六章中文.txt 10 64
1196 ， 有些 大惊小怪 地 围 着 女儿 问长问短。 金铃 被 问 得 烦 了， 说 第十九章中文.txt 14 45
1197 ， 弯 着 腰， 直 吐 得 浑身抽搐， 涕泪横流。 金铃 走 到 她 身边， 很 第二十一章中文.txt 6 18
1198 改变 主意。 可是 妈妈 在 房间 里 一声不响。 金铃 走 到 阳台， 打开 鞋 第二十一章中文.txt 6 42
1199 抽出 手指， 做 一个 鬼脸， 坐 得 毕恭毕敬。 金铃 选 了 一 支 红色 粉笔 第十九章中文.txt 14 39
1200 盯 着 那个 芭比 娃娃 看 得 目不转睛， 金铃 那时 就 有 了 要 送 第十章中文.txt 21 43
1201 冲 出 门 去。 全班 哄堂大笑。 金铃 鄙夷 地 撇 撇嘴 说： 第十八章中文.txt 12 10
1202 的 身影。 卉紫 赶快 抬头， 大喝一声：“金铃！” 金铃 没有 想 第二十四章中文.txt 4 10
1203 林立 的 大楼、 高耸 的 烟囱 和 高速公路、 铁路 破坏 了 你 和 你 家人 第十九章中文.txt 14 63
1204 个 男老师， 那样 的 话 可 就 糟糕透顶。 门 打开 了， 杨小丽 穿着 第二十五章中文.txt 5 62
1205 ， 她 就 笑眯眯 地 围 着 金铃 团团直转， 问 女儿 渴 不 渴， 又 问 第二十四章中文.txt 4 47
1206 她 发 这么 大 的 火。 她 小心翼翼 问 她：“是 不 是 来 例假 第二十五章中文.txt 5 68
1207 的， 还有 橘黄 的， 漂亮 极了！” 金铃 睁大眼睛 问：“真 的？ 不 会 是 老 第二十一章中文.txt 6
11
1208 呢！ 她 勉强 挣 出 个 笑容， 小心翼翼 问：“金铃 不是 应用题 全 对 第六章中文.txt 10 52
1209 就 把 电话 打 过 来 了， 小心翼翼 问 金铃：“考 了 多少 分？ 第十二章中文.txt 13 25
1210 吃 着 青菜， 觉得 味道 很 好， 不知不觉 间 一 筷 接着 一 筷， 一 第十章中文.txt 21 30
1211 十二分 顺当， 大学 时代 的 才华 在 字斟句酌 间 又 回到 了 她 身上。 晚上 第二十章中文.txt 7 42
1212 玩物。 也 就 在 那 时候， 你 走投无路 间 把头 扭来扭去 的 时候， 我 第十九章中文.txt 14 60
1213 一 看， 原来 教室 里 的 人 不知不觉 间 都 走 光 了， 只 剩 第4章中文.txt 25 34

| | | | | | |
|---|---|---|---|---|---|
| 1214 | 要 带 金铃 看 一 次 医生 ， | 是红是白 闹 个 准确 结论 ， 以后 也 好 | 第七章中文.txt | 11 | 8 |
| 1215 | 是 报警 又 是 到 电视台 发 | 寻人启事 ， 闹 得 一 条 街上 人心惶惶 。 | 第九章中文.txt | 9 | 11 |
| 1216 | 太 放在 心上 ， 这边 母女 两 个 | 哭哭笑笑 闹 得 惊天动地 ， 他 老先生 稳 | 第六章中文.txt | 10 | 55 |
| 1217 | 办 ？ ” 余老太 在 杂志社 里 一向 以 | 心慈手软 闻名 的 ， 所以 虽说 在 这里 工 | 第二十章中文.txt | 7 | 26 |
| 1218 | 了 。 最 有趣 的 是 ， 所有 话题 | 不约而同 集中 在 自己 的 儿女 们 身上 ， | 第4章中文.txt | 25 | 7 |
| 1219 | 、 浑身出汗 。 她 偷眼 看 一下 张老师 ， 张老师 | 正襟危坐 ， 面容 严肃 ， 看上去 她 不 是 | 第六章中文.txt | 10 | 26 |
| 1220 | ， 差点儿 跌进 李林 的 怀里 。 全班 | 哄堂大笑 。 音乐 老师 白嫩 白嫩 的 脸上 | 第十九章中文.txt | 14 | 12 |
| 1221 | 地上 蹒跚 地 挣扎 挪动 ， 却 是 | 无论如何 飞 不 到 天空 。 我 不 知道 | 第十九章中文.txt | 14 | 56 |
| 1222 | 这个 月 的 用 钱 计划 已经 | 了然在胸 ， 马上 对 妈妈 郑重 宣布 ： 坚 | 第十章中文.txt | 21 | 24 |
| 1223 | 肉 ， 你 这 是 虐待 父母 。” 金铃 | 洋洋得意 ， 马上 就 摆 出 了 当家人 的 | 第十章中文.txt | 21 | 27 |
| 1224 | 吐 吐 舌头 ， 心想 还 是 妈妈 | 老奸巨猾 ， 马上 就 猜 出 她 并 不 | 第十章中文.txt | 21 | 16 |
| 1225 | 成 了 “ 6 ” 。 金铃 眼见 妈妈 的 脸色 | 多云转阴 ， 马上 申明 说 ： “ 这 回 卷 | 第七章中文.txt | 11 | 14 |
| 1226 | 喘气 ， 一句话 不 说 。 卉紫 实在 觉得 | 莫名其妙 ， 高声 喊 书房 里 的 金亦鸣 。 | 第十六章中文.txt | 15 | 18 |
| 1227 | 同时 松 了 一口气 。 卉紫 有时候 想想 | 心犹不甘 ， 鼓励 金铃 说 ： “ 没 事 再 | 第十七章中文.txt | 16 | 47 |
| 1228 | 呢 ！ 金铃 的 语文 老师 气 得 | 面红耳赤 ， 鼻子 都 歪 到 了 旁边 。 | 第1章中文.txt | 23 | 23 |
| 1229 | ！ 作文 也 是 文学 ！ ” 金铃 激动 得 | 面红耳赤 ， 鼻孔 张开 来 ， 牙关 咬 起来 | 第十六章中文.txt | 15 | 15 |
