# Supplementary material for: Exploring translator’s style in children’s literature: A case study of Nicky Harman’s English translations of Huang Beijia’s two works
Source: PLoS One. 2026 Jun 2;21(6):e0350245. doi: 10.1371/journal.pone.0350245 (PMC13229296; doi:10.1371/journal.pone.0350245)
Supplement: S6 File — (PDF) [file pone.0350245.s009.pdf]

1 街上/s 还有/v 拉/v 藤/n 瓜/n 在/p 卖/v ，/wd 树/n 上/f 的/ude1 蝉儿叫成/nr —/m 条/q 声/ng ，/wd 到/p 晚上/t 还/d 得/ude3 泼/v 水/n ¥������.txt

2 收/vi 是/vshi 了不起/a 的/ude1 事情/n ，/wd 那个/rz 年代/n 肯/v 老老实实/z 去/vf 学/v 农/ng 的/ude1 人/n ，/wd 说/v 句/q 真话/n ，/wd ¥������.txt

3 wd 说/v 句/q 真话/n ，/wd 都/d 是/vshi 存/v 了/ule —/m 颗/q 救国救民/vl 的/ude1 大/a 爱/v 之/uzhi 心/n 的/ude1 人/n 。/wj 抗战/v ¥������.txt

4 ，/wd 出/vf 粉/n 率/k 又/d 高/a ，/wd 当年/t 的/ude1 亩产/vn 二百三十/m 斤/q ，/wd 报纸/n 上/f 称为/v 中国/ns 的/ude1 "/wyz 绿色/n ¥������.txt

5 d 他/rr 老人家/n 是/vshi 个/q 了不起/a 的/ude1 人/n ，/wd 为/p 中华民族/n 的/ude1 生存/vn 和/cc 繁衍/v 做出/v 了/ule 大/a 贡献/n 的/ud ¥������.txt

6 n 就/d 发/v 了/ule 动员令/n ，/wd 宣布/v "/wyz 地/n 不/d 分/v 东西南北/nl ，/wd 人/n 不/d 分/v 男女老幼/nl ，/wd 必须/d 全民/n 奋起/v ，¥������.txt

7 d 宣布/v "/wyz 地/n 不/d 分/v 东西南北/nl ，/wd 人/n 不/d 分/v 男女老幼/nl ，/wd 必须/d 全民/n 奋起/v ，/wd 不惜/v 牺牲/v 焦土/n 抗战/vi ¥������.txt

8 /wj 命令/n 一下/mq ，/wd 全国/n 的/ude1 工/n 农/ng 商/vg 学/v 各行各业/nl 都/d 响应/v 起来/vf 啦/y ，/wd 抛/v 家/n 别/d 舍/v ，/wd 离 ¥������.txt

9 业/nl 都/d 响应/v 起来/vf 啦/y ，/wd 抛/v 家/n 别/d 舍/v ，/wd 离乡背井/vl ，/wd 上/f 前线/s 的/ude1 上/f 前线/s ，/wd 去/vf 后/f 方/ ¥������.txt

10 大学/n 内/f 迁/v ，/wd 租/v 了/ule 三/m 只/q 轮船/njtgj 从/p 南京下关/ns 码头/n 出发/vi ，/wd 先/d 抵/v 汉口/ns ，/wd 又/d 换上/v 小/ ¥������.txt

11 /wj
那个/rz 小院/n 有/vyou 个/q 好听/a 的/ude1 也/d 是/vshi 名副其实/vl 的/ude1 名字/n ：/wm 榴/ng 园/ng 。/wj 两/m 层/qv 的/ude ¥������.txt

12 /n 地/ude2 挑衅/vi 小/a 女孩/n ，/wd 一会儿/mq 爹/n 开/v 翅膀/n 冤家对头/n 一样/uyy 斗/v 得/ude3 昏天黑地/al ，/wd 简直/d 就/d 是/vshi ¥������.txt

13 ，/wd 一会儿/mq 爹/n 开/v 翅膀/n 冤家对头/n 一样/uyy 斗/v 得/ude3 昏天黑地/al ，/wd 简直/d 就/d 是/vshi 榴/ng 园/ng 里/f 的/ude1 —/m ¥������.txt

14 学校/n 辙/n 退/v 到/v 成都/ns ，/wd 头/m —/m 件/q 大事/n 便是/v 想方设法/dl 找到/v 他/rr 。/wj 爸爸/n 把/pba 他/rr 从/p 川/b 西/b 乡下/ ¥������.txt

15 /a 紫/a ，/wd 石榴花/n 红/a 的/ude1 季节/n 。/wj
出生/vi 在/p 成都华西/ns 坝上/s 的/ude1 小弟/n 四/m 个/q 月/n 大/a 了/y 。/wj 四/m
¥������.txt

16 de1 上帝/n ！/wt "/wyz
因为/p 我/rr 娘/n 在/p 逃难/vi 路上/s 颠沛流离/vl ，/wd 严重/a 营养/n 不足/an ，/wd 才/d 导致/v 小弟/n 生/v 出/ ¥������.txt

17 地/n ，/wd 让/v 楼上/s 楼下/s 的/ude1 每/rz 户/ng 人家/rr 都/d 蹑手蹑脚/z 地/ude2 走路/vi ，/wd 蹑手蹑脚/z 地/ude2 做/v 事/n ，/wd 生怕 ¥������.txt

18 ude1 每/rz 户/ng 人家/rr 都/d 蹑手蹑脚/z 地/ude2 走路/vi ，/wd 蹑手蹑脚/z 地/ude2 做/v 事/n ，/wd 生怕/v 惊扰/v 了/ule 那样/rzv —/m ¥������.txt

19 有/vyou 人/n 坐/v 在/p 台下/s 鼓掌/vi ，/wd 这/rzv 才/d 叫/vi 各得其所/vl 。/wj
还是/c 说/v 回/qv 小弟/n 。/wj 小弟/n 四/m 个/q 月/n ¥������.txt

20 后/f ，/wd 他/rr 就/d 带/v 着/uzhe 他/rr 的/ude1 学生/n 们/k 马不停蹄/vl 地/ude2 出门/vi ，/wd 跟/p 当地/s 政府/n 合办/v "/wyz 农业/ ¥������.txt

21 n —/d 喊/v ，/wd 他/rr 就/d 咧/v 嘴/n 笑/v ，/wd 大/a 眼睛/n 忽闪忽闪/z ，/wd 转/v 着/uzhe 脑袋/n 循声/d 找/v 人/n ，/wd 口水/n 湿/a ¥������.txt

22 yz 有/vyou 用/v 没用/vi 呢/y ？/ww "/wyz
邢/nr1 姆/x 妈/n 大包大揽/vl ：/wm "/wyz 有/vyou 用/v 没用/vi ，/wd 总归/d 试/v 了/ul
¥������.txt

23 /ule 才/d 晓得/v 哦/e 。/wj "/wyz
我/rr 娘/n 回家/vi ，/wd 迟迟疑疑/z 告诉/v 我/rr 姐/n ，/wd 意思/n 要/v 我/rr 姐/n 帮/v 她/rr 写
¥������.txt

24 积极/a 的/ude1 参与者/n ，/wd 我/rr 赶紧/d 地/ude2 冲/v 上去/vf 自告奋勇/vl ：/wm "/wyz 娘/n ，/wd 我会/r 写字/vi ，/wd 我/rr 来/vf ¥������.txt

25 p 娘/n 的/ude1 吩咐/vn ，/wd 在/p 每/rz 张/q 纸条/n 上/f 都/d 歪歪扭扭/z 写/v 上/f 同样/d —/m 句/q 话/n ：/wm 天/n 皇皇/z ，/wd 地/n ¥������.txt

26 ：/wm "/wyz 呵呵/e ，/wd 上学/vi 考/v 头名/n 没/v 本事/n ，/wd 装神弄鬼/vl —/d 学/v 就/d 会/v 啊/y ，/wd 你/rr 看看/v 你/rr 这/rzv ¥������.txt

27 i 往/p 纸/n 上/f 写/v 。/wj "/wyz
我/rr 埋/v 着/uzhe 头/n 得意洋洋/al 写/v ，/wd 根本/d 不/d 理会/v 她/rr 。/wj 我/rr 知道/v 她/r
¥������.txt

28　le 我/rr 爸/n 的/ude1 一/m 支/q 秃/a 毛笔/n 当/p 刷子/n ，/wd 趁热打铁/vl 地/ude2 带/v 上/vf
小/a 素/dg 出门/vi 贴/v 纸条/n 儿/ng 。　¥�������.txt
29　就/d 拉/v 着/uzhe 小/a 素/dg 躲/v 在/p 树荫/n 里/f ，/wd 看/v 南来北往/vl 过路/v 的/ude1
有/vyou 没/d 有/vyou 人/n 肯/v 站/vi 下/f　¥�������.txt
30　/vi 跑/v 野/ng 玩/v ，/wd 她/rr 都/d 是/vshi 我/rr 的/ude1 坚定不移/vl 的/ude1 追随者/n 。/wj 我
们/rr 顺着/p 陕西/ns 街/n 的/ude1　¥�������.txt
31　uzhe 几/m 丛/q 红色/n 美人蕉/n 的/ude1 小/a 客栈/n ，/wd 还有/v 零零落落/z 几/m 户/q 家居/n
小院/n 门/n 。/wj 才/c 五月/t ，/wd 天/qt 已　¥�������.txt
32　d 路上/s 总/d 不/d 见/v 行人/n ，/wd 石缝/n 里/f 的/ude1 杂草/n 探头探脑/al 钻/v 出来/vf ，/wd
锯齿/n 状/ng 的/ude1 叶片/n 擦/v 着/uzh　¥�������.txt
33　r 收/v 声/n 了/y ，/wd 眼泪/n 还/d 没/d 干/v ，/wd 眼睛/n 便/d 东张西望/vl ：/wm "/wyz 哪儿/rys
呢/y ？/ww 桑果/n 在/p 哪儿/rys 呢/y　¥�������.txt
34　r 带/v 到/v 河边/s 。/wj 我/rr 让/v 她/rr 坐/v 在/p 一/m 截/q 疙疙瘩瘩/z 的/ude1 老/a 树桩/n
子/ng 上/f 等/v 着/uzhe 我/rr ，/wd 别　¥�������.txt
35　没有/v ，/wd 被/pbei 我们/rr 这些/rz 天/qt 里/f 吃/v 得/ude3 干干净净/z 。/wj 唉/e 唉/e ，/wd
早/ad 知道/v 就/d 留下/v 几/m 颗/q 了/　¥�������.txt
36　d 没/v 哪/ry 棵/q 树/n 难倒/v 过/uguo 我/rr 。/wj 桑树/n 更/d 不在话下/vl ，/wd 枝繁叶茂/vl
的/ude1 ，/wd 树干/n 长/a 不/d 高/a ，/wd　¥�������.txt
37　q 树/n 难倒/v 过/uguo 我/rr 。/wj 桑树/n 更/d 不在话下/vl ，/wd 枝繁叶茂/vl 的/ude1 ，/wd 树
干/n 长/a 不/d 高/a ，/wd 树皮/n 又/d 糙/a　¥�������.txt
38　碰/v 破/v 了/ule 果皮/n ，/wd 紫/a 黑色/n 的/ude1 甜/a 汁/ng 噼噼啪啪/o 迸/vi 出来/vf ，/wd
沾/v 到/v 衣服/n 上/f ，/wd 那/rzv 就是/　¥�������.txt
39　ude1 一/m 顿/qv 打/v 。/wj
我/rr 踩/v 着/uzhe 树干/n ，/wd 小心翼翼/dl 探/v 出/vf 身子/n ，/wd 轻/ad 拿/v 轻/ad 放/v 地/ude2 在/
¥�������.txt
40　颗/q ，/wd 窝/v 在/p 手/n 心里/s 。/wj 几乎/d 在/p 同时/n ，/wd 条件反射/nl 一样/uyy ，/wd
口/q 水/n 不/d 争气/a 地/ude2 从/p 我/rr 的　¥�������.txt
41　d 垂/v 着/uzhe 两/m 眼/q 往/p 地/n 上/f 看/v ，/wd 一/m 副/q 事不关己/vl 爱/v 搭/v 不/d 理/v
的/ude1 样/ng 儿/ng 。/wj
那边/rzs ¥�������.txt
42　ude1 。/wj 他/rr 一定/d 不/d 喜欢/vi 看/v 我们/rr 和/cc 爸爸/n 亲密无间/vl 的/ude1 情景/n 。/wj
"/wyz 橙子/n ！/wt "/wyy 我/rr 爸　¥�������.txt
43　s ，/wd 手/n 扬/vg 起来/vf ，/wd 啪/o 的/ude1 一下子/mq ，/wd 出其不意/nl 地/ude2 糊/v 到/v
我/rr 的/ude1 膝盖/n 上/f 。/wj
我/rr　¥�������.txt
44　ude2 糊/v 到/v 我/rr 的/ude1 膝盖/n 上/f 。/wj
我/rr 简直/d 目瞪口呆/al 。/wj 如果/c 我/rr 事先/d 知道/v 他/rr 会/v 把/pba 唾液/n 糊
¥�������.txt
45　/wd 居然/d 驮/v 着/uzhe 我/rr 不/d 带/v 喘气/vi 。/wj
我/rr 心惊胆战/al 地/ude2 趴/v 在/p 他/rr 背/v 上/f ，/wd 一瞬间/t 心里/s 闪/　¥�������.txt
46　/a 糊/v 的/ude1 ，/wd 全都/d 是/vshi 。/wj 那/rzv 一/m 片/q 触目惊心/vl 、/wn 淋漓尽致/al
的/ude1 红/a ，/wd 瞥/v 一/m 眼/q 都/d 让　¥�������.txt
47　，/wd 全都/d 是/vshi 。/wj 那/rzv 一/m 片/q 触目惊心/vl 、/wn 淋漓尽致/al 的/ude1 红/a ，/wd
瞥/v 一/m 眼/q 都/d 让/v 人/n 心/n 惊/　¥�������.txt
48　我/rr 妹/n ，/wd 都/d 显得/v 那么/rz 陌生/a ，/wd 遥远/a ，/wd 格格不入/vl 。/wj
他/rr 长/v 得/ude3 矮小/a ，/wd 又/d 精瘦/z ，/wd　¥�������.txt
49　，/wd 扮/v 个/q 鬼脸/n ，/wd 算是/v 回答/v 。/wj
沈天/nr 路/n 面红耳赤/al ，/wd 一/m 双/q 大脚/n 用劲/vi 地/n 踢/n 着/uzhe 地面/n ，/
¥�������.txt
50　不可/v ，/wd 弄/v 得/ude3 校长/n 老师/n 个个/q 宠/v 她/rr ，/wd 一来二去/dl ，/wd 宠/v 得/ude3
她/rr 家里/s 家/n 外/f 都/d 是/vshi 鼻　¥�������.txt
51　家里/s 家/n 外/f 都/d 是/vshi 鼻孔/n 朝天/ns 。/wj 今天/t 爸/n 毫不留情/vl 地/ude2 呵斥/v 了/ule
她/rr ，/wd 还是/c 当着/p 沈/nr1 天/　¥�������.txt
52　当着/p 沈/nr1 天/n 路/n 的/ude1 面/n ，/wd 我/rr 立刻/d 觉得/v 扬眉吐气/vl ，/wd 还/d 有/vyou
点/n 幸灾乐祸/vl 。/wj
我/rr 姐/n 狠狠/　¥�������.txt
53　n ，/wd 我/rr 立刻/d 觉得/v 扬眉吐气/vl ，/wd 还/d 有/vyou 点/n 幸灾乐祸/vl 。/wj
我/rr 姐/n 狠狠/d 地/ude2 瞪/v 我/rr 一/m 眼/q 。/　¥�������.txt
54　虽然/c 只/d 大/a 了/ule 一/m 岁/qt ，/wd 却/d 完全/ad 是/vshi 地地道道/bl 大哥/n 的/ude1 派头/n
。/wj 他/rr 个子/n 高高的/z ，/wd 肩膀/　¥�������.txt
55　vi 搭/v 床/n 就是/d 仁厚/a 啊/y ？/ww 我/rr 不/d 是/vshi 也/d 大大方方/z 腾出/v 我/rr 的/ude1

小/a 桌子/n 给/p 沈天路/ns 用/v 了/ule ¥�������.txt
56 v 趴/v 在/p 床/n 边/k 上/f 写/v ，/wd 字/n 都/d 写/v 得/ude3 歪歪扭扭/z ，/wd 娘/n 就/d 没/d 看到/v 啊/y ？/ww
沈天路/ns 是/vshi 个 ¥�������.txt
57 /vi 。/wj 有/vyou 段/q 时间/n ，/wd 我/rr 觉得/v 好玩/a ，/wd 有意无意/dl 地/ude2 仿/v 他/rr 左撇子/n 的/ude1 习惯/n ，/wd 仿/v 到/v ¥�������.txt
58 he 了/y ，/wd 沈/nr1 天/n 路上/s 到/v 高中/n 后/f ，/wd 成绩/n 突飞猛进/vl ，/wd 差点儿/d 就/d 把/pba 我/rr 姐/n 甩/v 到/v 了/ule 身后 ¥�������.txt
59 /n 发现/v ，/wd 呵斥/v 了/ule 几/m 次/qv 后/f ，/wd 不/d 敢/v 明目张胆/nl 学/v 他/rr 的/ude1 口音/n 了/y ，/wd 背地里/d 却/d 拿/v "/ ¥�������.txt
60 字/n 对口/vn 型/k ，/wd 做/v 鬼脸/n ，/wd 弄/v 出/vf 一/m 副/q 心照不宣/vl 的/ude1 鬼祟/a 样/u 。/wj
沈天路/ns 明白/v 我们/rr 之间/f 的 ¥�������.txt
61 就/d 体会/v 不/d 到/v 一个/mq 初/d 涉/vg 人世/n 的/ude1 男孩/n 举目无亲/vl 的/ude1 心境/n 的/ude1 呢/y ？/ww 全/a 因为/c 我们/rr 的/u ¥�������.txt
62 语/nz 一/m 句/q 不/d 会/v ，/wd 数学/n 还/d 没/d 学/v 过/uguo 因式分解/n ，/wd 初三/t 的/ude1 功课/n 肯定/d 是/vshi 跟不上/v ，/wd 给/ ¥�������.txt
63 都/d 是/vshi 最/d 会/v 念/v 书/n 的/ude1 人/n ，/wd 是/vshi 中华民族/n 最/d 优秀/a 的/ude1 学子/n ，/wd 天/qt 路/n 一定/d 是/vshi ¥�������.txt
64 wd 金陵/ns 大学/n 从/p 战火/n 中/f 的/ude1 南京/ns 撤退/vi 到/v 成都华西/ns 坝/n 之后/f ，/wd 跟/p 北平燕京/ns 大学/n 、/wn 山东齐鲁/ns 大学 ¥�������.txt
65 的/ude1 南京/ns 撤退/vi 到/v 成都华西/ns 坝/n 之后/f ，/wd 跟/p 北平燕京/ns 大学/n 、/wn 山东齐鲁/ns 大学/n 、/wn 南京金陵/ns 女子/n 大学/n ¥�������.txt
66 i 到/v 成都华西/ns 坝/n 之后/f ，/wd 跟/p 北平燕京/ns 大学/n 、/wn 山东齐鲁/ns 大学/n 、/wn 南京金陵/ns 女子/n 大学/n 这/rzv 几/m 所/usuo 教 ¥�������.txt
67 之后/f ，/wd 跟/p 北平燕京/ns 大学/n 、/wn 山东齐鲁/ns 大学/n 、/wn 南京金陵/ns 女子/n 大学/n 这/rzv 几/m 所/usuo 教会/v 大学/n 同时/c 挤/v ¥�������.txt
68 /wyz
我/rr 攥/v 着/uzhe 那/rzv 半/m 块/q 红/a 橡皮/n ，/wd 迷迷糊糊/z 的/ude1 ，/wd 心思/n 还/d 在/p 小/a 兔子/n 身上/s 。/wj
"/ ¥�������.txt
69 在/p 小/a 兔子/n 身上/s 。/wj
"/wyz 哎/e ，/wd "/wyy 她/rr 老气横秋/al 地/ude2 说/v ，/wd "/wyz 如果/c 你/rr 真/d 喜欢/vi 那个/r ¥�������.txt
70 r 的/ude1 心爱/b 的/ude1 铅笔盒/n 的/ude1 时候/n ，/wd 禁不住/v 大惊失色/vl ：/wm 兔子/n 不见/v 了/y ！/wt 放/v 铅笔盒/n 的/ude1 那/rzv ¥�������.txt
71 j
恨/v 恨/v 地/ude2 瞪/v 他/rr 一/m 眼/q 之后/f ，/wd 我/rr 摇摇晃晃/z ，/wd 深/d 一/m 脚/q 浅/a 一/m 脚/n 地/ude2 走/v 出/vf 门/ ¥�������.txt
72 e1 ，/wd 又/d 黑/a 又/d 沉/v ，/wd 一个/mq 劲儿/n 下坠/v ，/wd 处心积虑/dl 要/v 把/pba 我/rr 拖/v 进/vf 悲哀/a 无边/z 的/ude1 黑夜/n ¥�������.txt
73 我/rr 哥/n 。/wj 娘/n 是/vshi 怕/v 粮食/n 糟蹋/v 了/y 。/wj
意想不到/vl 的/ude1 打击/vn 接踵而至/vl 。/wj 我/rr 爸/n 吃/v 完/vi 饭/ ¥�������.txt
74 vshi 怕/v 粮食/n 糟蹋/v 了/y 。/wj
意想不到/vl 的/ude1 打击/vn 接踵而至/vl 。/wj 我/rr 爸/n 吃/v 完/vi 饭/n ，/wd 放下/v 饭碗/n ，/wd ¥�������.txt
75 n 刷/v 出来/vf 的/ude1 一样/uyy ，/wd 板/ng 板正/a 正/a ，/wd 平平淡淡/z ，/wd 唉/e 唉/e ，/wd 真是/d 没劲/a 。/wj
他/rr 在/p 笨/a ¥�������.txt
76 wd 包/v 口/n 敞/v 着/uzhe ，/wd 露出/v 那个/rz 崭新/b 的/ude1 金光闪闪/vl 的/ude1 文具盒/n 。/wj 不/d 知道/v 怎么/ryv 的/ude1 ，/wd ¥�������.txt
77 /vl 的/ude1 文具盒/n 。/wj 不/d 知道/v 怎么/ryv 的/ude1 ，/wd 鬼使神差/vl 一样/uyy ，/wd 我/rr 发现/v 我/rr 心里/s 冒/v 出/vf 一个/mq ¥�������.txt
78 里/s 冒/v 出/vf 一个/mq 狰狞/a 的/ude1 魔鬼/n ，/wd 它/rr 在/p 龇牙咧嘴/vl 地/ude2 怂恿/v 我/rr 去/vf 做/v 坏事/n ，/wd 很/d 坏/a 很/ ¥�������.txt
79 /v 坏事/n ，/wd 很/d 坏/a 很/d 坏/a 的/ude1 事/n 。/wj 我/rr 迷迷糊糊/z 地/ude2 走/v 过去/vf 抽出/v 文具盒/n ，/wd 掩/v 在/p 怀里/s ， ¥�������.txt
80 /v 了/ule 几/m 根/q 枯枝/n 木条/n 什么的/r 遮/v 上/f ，/wd 又/d 心惊肉跳/al 地/ude2 回家/vi ，/wd 接着/c 扫地/vi ，/wd 抹/v 桌子/n ，/w ¥�������.txt
81 /ude1 异常/a 。/wj 通常/d 早晨/t 是/vshi 一/m 天/qt 里/f 最/d 手忙脚乱/al 的/ude1 时辰/n ，/wd 连/ulian 万事/n 不/d 管/v 的/ude1 我 ¥�������.txt

82 及格/vi ，/wd 已然/d 拉/v 了/ule 全班/n 后腿/n ，/wd 此时/r 又/d 雪上加霜/vl 地/ude2 不/d 带/v
文具/n ，/wd 先生/n 从/p 心里/s 就/d 认为/ ¥�������.txt
83 vf 咧/y 。/wj
当时/t 都/d 没/d 在意/v 。/wj 学校/n 嘛/y ，/wd 大大小小/z 两百/m 多/m 个/q 学生/n 呢/y ，/wd
有/vyou 用功/a 的/ude1 ， ¥�������.txt
84 /v 一/m 圈/qv 没/d 找/v 着/uzhe 人/n 。/wj 我/rr 娘/n 开始/v 心惊胆战/al ，/wd 怕/v 沈天路/ns
出/vf 了/ule 什么/ry 事/n 。/wj 我/rr ¥�������.txt
85 e1 实验室/n 里/f 找/v 他/rr 禀告/v 实情/n 。/wj 我/rr 爸/n 也/d 大惊失色/vl ，/wd 赶紧/d 叫/vi
上/f 实验室/n 里/f 的/ude1 两/m 个/q 学生 ¥�������.txt
86 /n 。/wj 跑/v 出去/vf 才/d 知道/v 自己/rr 不/d 认/v 路/n ，/wd 转来转去/vl 还是/d 围/v 着/uzhe
锦/ag 秀/ag 河边/s 转圈/vi 圈/v ，/wd ¥�������.txt
87 /s 转圈/vi 圈/v ，/wd 转/v 得/ude3 又/d 累/v 又/d 饿/v ，/wd 好不容易/dl 挨/v 到/p 晚上/t ，/wd
偷/d 啃/v 了/ule 人家/n 地/n 里/f 两 ¥�������.txt
88 wj 他/rr 沉默/v 了/ule 好/a 一会儿/mq ，/wd 调整/v 气息/n ，/wd 和颜悦色/vl 地/ude2 说/v ：/wm
"/wyz 行/vi 吧/y ，/wd 天/qt 路/n ， ¥�������.txt
89 我/rr ，/wd 我/rr 心里/s 的/ude1 那个/rz 魔鬼/n 才/d 是/vshi 罪魁祸首/nl 。/wj
那个/rz 漂亮/a 的/ude1 文具盒/n ，/wd 是/vshi 我/rr ¥�������.txt
90 d 是/vshi 我/rr 从/p 后园/n 墙/n 洞/n 里/f 扒/v 出来/vf ，/wd 痛哭流涕/al 交/v 给/p 爸爸/n 的/ude1
。/wj 我/rr 本来/d 准备/v 好/a 了/ ¥�������.txt
91 无视/v 我/rr 存在/v 。/wj 整整/d 一个/mq 星期/n 里/f ，/wd 我/rr 谨小慎微/vl 地/ude2 约束/v 自
己/rr ：/wm 吃/v 菜/n 只/d 吃/v 我/rr 平常 ¥�������.txt
92 r 主动/ad 加/v 量/n ，/wd 别人/rr 抄/v 十/m 遍/qv ，/wd 我/rr 一丝不苟/vl 地/ude2 抄/v 上/vf 二
十/m 遍/qv 。/wj 我/rr 还/d 抢/v 着/ ¥�������.txt
93 d 他/rr 头/n 一个/mq 明白/v 了/ule 我/rr 的/ude1 意思/n ，/wd 真心诚意/dl 夸奖/v 我/rr 说/v
：/wm "/wyz 橙子/n 不/d 简单/a ，/wd 懂得 ¥�������.txt
94 我/rr 姐/n 笑眯眯/z 地/ude2 拿走/v 了/y 。/wj 我/rr 姐/n 还/d 甜言蜜语/nl 对/p 我/rr 娘/n 保证/v
，/wd 等/udeng 小/a 素/dg 念/v 了/ ¥�������.txt
95 儿/n ，/wd 怎么/ryv 就/d 没有/d 长/v 出/vf 我/rr 姐/n 的/ude1 花花肠子/nl 呢/y ？/ww
第三/m 章/q ·/w 倒霉/a 的/ude1 大/a 公鸡/n
十 ¥�������.txt
96 t 哪个/ry 男/b 娃/ng 会/v 要/v 你/rr ？/ww "/wyz 我/rr 娘/n 长吁短叹/vl ，/wd 在/p 心里/s 设想/v
了/ule 一百/m 遍/qv 我/rr 嫁/v 不/ ¥�������.txt
97 ule 三/m 条/q 红/a 杠杠/n 。/wj 她/rr 一边/d 打/v ，/wd 一边/d 声泪俱下/vl 地/ude2 控诉/v 我/rr
：/wm "/wyz 哪/ry 见/v 过/uguo 你/ ¥�������.txt
98 弟弟/n 的/ude1 脑/n 勺/ng 磕/v 出/vf 一个/mq 包/n 啦/y ；/wf 好不容易/dl 穿/v 件/q 新/a 衣服/n
上/f 学/v ，/wd 却/d 兴奋/a 过头/a 洒/ ¥�������.txt
99 ，/wd 梅/nr1 教授/n 抱/v 着/uzhe 他/rr 的/ude1 那/rzv 只/d 威风凛凛/al 的/ude1 红/a 羽毛/n 爱尔
兰/nsf 良种/n 鸡/n ，/wd 悲恸/a 欲/v ¥�������.txt
100 /d 是/vshi "/wyz 农/ng 科学/n "/wyy ，/wd 彼此/rr 有/vyou 共同语言/n 。/wj 梅/nr1 教授/n 本来/d
在/p 美国/nsf 威斯康星/nsf 大学/n 学 ¥�������.txt
101 /rr 有/vyou 共同语言/n 。/wj 梅/nr1 教授/n 本来/d 在/p 美国/nsf 威斯康星/nsf 大学/n 学生/n 物/ng
，/wd 博士/n 毕业/v 后/f 回国/vi ，/wd ¥�������.txt
102 /rr 后脚/n 起身/vi 招呼/v ，/wd 然后/c 跟/v 着/uzhe 我/rr 爸/n 咯吱咯吱/o 爬/v 上/vf 楼/n ，/wd
两/m 人/n 泡/v 壶/ng 茶叶末/n ，/wd 坐 ¥�������.txt
103 的/ude1 解释/vn 。/wj
不过/c 大多数/m 时间/n ，/wd 他们/rr 会/v 心平气和/vl 地/ude2 讨论/v 跟/p 教学/vn 有关/vn 的/ude1 问
题/n 。/wj 有 ¥�������.txt
104 v 梅/nr1 教授/n 共同/d 担任/v 指导/vn 老师/n ，/wd 梅/nr1 教授/n 二话不说/vl 答应/v 了/y 。/wj
还有/v 一/m 年/qt 春天/t ，/wd 我/rr 爸/n ¥�������.txt
105 n 都/d 起/vf 了/ule 疱/n 。/wj 梅/nr1 教授/n 衔/v 个/q 烟斗/n 心平气和/vl 地/ude2 开导/v 他/rr
：/wm "/wyz 偷/v 就/d 偷/v 了/ule ¥�������.txt
106 /v 。/wj "/wyz
"/wyz 不/d 坐/v ！/wt "/wyy 梅/ng 教授/n 义正词严/vl ，/wd "/wyz 子/ng 不/d 教/v ，/wd 父/ng 之/uzhi
过/ng ¥�������.txt
107 沈/nr1 天/n 路/n ，/wd 兄弟/n 姐妹/n 有/vyou 六/m 个/q ，/wd 防不胜防/vl ，/wd 不/d 当/p 心/n
就/d 会/v 有/vyou 哪个/ry 闯/v 下/vf ¥�������.txt
108 /v 我/rr 这/rzv 对/p 种鸡/n 多/a 宝贝/n ？/ww 我/rr 千/m 里/q 迢迢万里/nl 迢迢/z 从/p 南京/ns 带/v
过来/vf 的/ude1 啊/y ，/wd 我们/rr ¥�������.txt
109 /d 认定/v 是/vshi 我家/n 的/ude1 ？/ww "/wyz
梅/nr1 教授/n 掷地有声/vl ：/wm "/wyz 我/rr 这/rzv 两/m 只/q 鸡/n ，/wd 没/v 人/n¥�������.txt
110 鸡/n 身上/s 走/v 一/m 圈/qv 。/wj 公鸡/n 死活/d 不/d 从/p ，/wd 怒目圆睁/vl ，/wd 脖子/n 上/f

的/ude1 毛一根根/nr 都/d 竖/v 起来/vf ，/w ￥�������.txt
111 /n 死活/d 不/d 从/p ，/wd 怒目圆睁/vl ，/wd 脖子/n 上/f 的/ude1 毛一根根/nr 都/d 竖/v 起来/vf
，/wd 伸/v 着/uzhe 脖颈/n 往/p 我/rr 面前 ￥�������.txt
112 /d 可惜/v 那/rzv 只/q 猫/n 野/ng 怂/vg 了/y ，/wd 趁/p 我/rr 手忙脚乱/al 时/ng ，/wd “/wyz 啊/y
呜/o ”/wyy 一/m 声/qv 惨叫/vi ， ￥�������.txt
113 时/ng ，/wd “/wyz 啊/y 呜/o ”/wyy 一/m 声/qv 惨叫/vi ，/wd 落荒而逃/al 。/wj
也/d 怪/v 我/rr 自己/rr 太/d 大意/a ，/wd 昨天/t 偷/ ￥�������.txt
114 ，/wd 肯定/v 被/pbei 人/n 看见/v 了/y ，/wd 而且/c 这/rzv 人/n 居心不良/vl 地/ude2 向/p 梅/nr1 教
授/n 告状/vi 了/y 。/wj 没/v 准儿/n ￥�������.txt
115 vi 了/y 。/wj 没/v 准儿/n 就是/v 我/rr 姐/n ，/wd 她/rr 总是/d 处心积虑/dl 地/ude2 要/v 看/v 我/rr
笑话/n ，/wd 好/d 显得/v 自己/rr 多 ￥�������.txt
116 错/n ，/wd 对不住/v 对不住/v 。/wj "/wyz
梅/nr1 教授/n 依旧/z 痛心疾首/vl ：/wm "/wyz 三/m 根/q 尾/ng 羽/ng ！/wt 你/rr 女儿/n 是/
￥�������.txt
117 q 尾/ng 羽/ng 的/ude1 宝贝/n 补/v 身体/n 。/wj
梅/nr1 教授/n 怒气冲冲/vl 走/v 了/ule 之后/f ，/wd 我/rr 关/v 紧/a 房门/n ，/wd 屁股/
￥�������.txt
118 去/vf 抢/v 我/rr 姐/n 手里/s 的/ude1 毽子/n 。/wj 我/rr 甚至/d 不顾一切/nl 地/ude2 咬/vi 了/ule
她/rr 一/m 口/q ，/wd 咬/vi 得/ude ￥�������.txt
119 uo 小孩子/n ，/wd 他/rr 的/ude1 手/n 一直/d 在/p 哆嗦/vi ，/wd 犹豫不决/vl ，/wd 也许/d 是/vshi
拿/v 不/d 准/a 该/rz 用/p 多/m 大/a ￥�������.txt
120 r 是/vshi 想/v 拿/v 个/q 头/m 名/q 。/wj "/wyz
我/rr 爸/n 莫名其妙/al ：/wm "/wyz 什么/ry 头名/n ？/ww "/wyz
"/wyz 踢/v 毽子 ￥�������.txt
121 。/wj
"/wyz 那/rzv 好/a ，/wd "/wyy 我/rr 爸/n 做/v 个/q 不容置疑/vl 的/ude1 手势/n ，/wd "/wyz 比赛/v 完
了/vi ，/wd 拿/v 着/u ￥�������.txt
122 我/rr 也/d 不/d 知道/v 我/rr 为什么/ryv 要/v 哭/v ，/wd 有点/d 莫名其妙/al 。/wj
还有/v ，/wd 我/rr 死/v 活/n 都/d 没/d 想到/v ，/wd ￥�������.txt
123 天路/ns 跟/p 我/rr 不/d 在/p 一个/mq 学校/n ，/wd 又/d 是/vshi 不声不响/vl 独/d 往/p 独/d 来/vf
的/ude1 一个/mq 人/n ，/wd 他/rr 怎么 ￥�������.txt
124 ，/wd 他/rr 有/vyou 一/m 本/q 英文版/n 的/ude1 《/wkz 少年/n 百科全书/n 》/wky ，/wd 听说/v
里面/f 写/v 尽/vi 了/ule 世界/n 上/f 所有 ￥�������.txt
125 做/v 了/ule 一/m 件/q 冬衣/n 。/wj 我/rr 和/cc 姐姐/n 是/vshi 阴丹士林/n 布/vg 的/ude1 ，/wd 布
料/n 不怎么/vl 够/v ，/wd 衣领/n 用/v ￥�������.txt
126 nr1 丝绸/n 。/wj 我/rr 姐/n 认为/v 这个/rz 花色/n 衣领/n 称得上/v 神来之笔/nl ，/wd 瞬间/t 让/v 我
们/rr 的/ude1 普通/a 棉衣/n 变/v 得/ude ￥�������.txt
127 t 不/d 伤/v 老百姓/nt ，/wd 不/d 打/v 自己/rr 人/n 。/wj 维护/v 中华民族/n ，/wd 永/d 做/v 自由/a
人/n ！/wt 他们/rr 班级/n 在/p 操场/n ￥�������.txt
128 v 的/ude1 时候/n ，/wd 鼓声/n 震天/vi ，/wd 歌声/n 嘹亮/a ，/wd 气势磅礴/vl ，/wd 可/c 真/d
让/v 别的/rzv 班级/n 羡慕/v 到/v 眼睛/n 发绿/ ￥�������.txt
129 wj
我们/rr 四/m 年级/n 班/n 的/ude1 老师/n 不/d 服气/vi ，/wd 一门心思/dl 要/v 在/p 气势/n 上/f 压倒/v
五/m 年级/n 。/wj 怎么/ryv 压/v ￥�������.txt
130 d 都/d 要/v 哭/v 出来/vf 。/wj 闹/v 半天/mq ，/wd 我/rr 即便/c 赴汤蹈火/vl 完成/v 了/ule 任务/n
，/wd 也/d 不过/d 是/vshi 老师/n 口中/s ￥�������.txt
131 j
忘/v 了/ule 说/v 了/y ，/wd 从/p 我们/rr 榴/ng 园/ng 到/v 金女大琴/nr 房/n ，/wd 路程/n 有点/d 远/a
，/wd 步行/vi 起码/d 半/m 小时/ ￥�������.txt
132 ude1 弄堂/n 、/wn 一/m 座/q 烛光/n 闪动/v 气息/n 幽远/a 的/ude1 天主教堂/n ，/wd 如果/c 我/rr
一个/mq 人/n 独自/d 行走/vi 的/ude1 话/n ￥�������.txt
133 而/cc 找/v 我/rr 哥/n 。/wj 我/rr 哥/n 是/vshi 绅士/n ，/wd 一般来说/vl 他/rr 很/d 少/ad 拒绝/v 女
孩子/n 。/wj 果然/c ，/wd 我/rr 一 ￥�������.txt
134 是/d 好/a 的/ude1 。/wj
很多/m 年/qt 之后/f 我/rr 还/d 记得/v 那天晚上/t 的/ude1 星空/n 。/wj 那么/rz 多/a 的/ude1 星星/n
，/wd 排山倒 ￥�������.txt
135 天晚上/t 的/ude1 星空/n 。/wj 那么/rz 多/a 的/ude1 星星/n ，/wd 排山倒海/bl 一样/uyy ，/wd
一/m 声/qv 吆喝/v 就/d 会/v 从/p 天上/s 哗啦啦 ￥�������.txt
136 d 是/vshi 躲/v 在/p 黑影/n 里/f 的/ude1 。/wj "/wyz
我/rr 无话可说/vl 。/wj
树/n 林子/n 越来越/d 密/a 。/wj 白天/t 的/ude1 时候/n ￥�������.txt

137 wn 油葫芦/n 、/wn 铁砂/n 青/a 、/wn 藤/n 花/v 紫/a 。/wj 他/rr 绘声绘色/al 地/ude2 解释/v ，/wd 哪/ry 种/q 蟋蟀/n 头/n 型/k 什么样/ryv ¥�������.txt
138 劲头/n ，/wd 饲养/v 又/d 该/v 注意/v 什么/ry ，/wd 说/v 得/ude3 头头是道/al 。/wj 我/rr 惊奇/a 地/ude2 发现/v 他/rr 其实/d 是/vshi 个/ ¥�������.txt
139 vi 。/wj 他/rr 也许/d 听见/v 我/rr 叫/vi 得/ude3 凄厉/a ，/wd 于心不忍/vl ，/wd 这/rzv 才/d 回/v 转/v 来/vf ，/wd 摸/v 到/v 我/rr ¥�������.txt
140 wd 转而/c 乖乖/d 求/v 他/rr ：/wm "/wyz 天/n 路/n 哥/n ，/wd 可不可以/v 拉/v 住/vi 你/rr 的/ude1 手/n ？/ww "/wyz
那/rzv 是/vs ¥�������.txt
141 /y 。/wj 他/rr 瓮/n 声/qv 瓮/n 气/n 对/p 我/rr 下/vf 了/ule 最后通牒/nl ：/wm "/wyz 走/v 还是/c 不/d 走/v ？/ww "/wyz
"/wyz ¥�������.txt
142 /uzhe 他/rr 的/ude1 棍子/n 走/v ，/wd 一路/mq 心虚/a 地/ude2 东张西望/vl 着/uzhe ，/wd 生怕/v 横/dg 刺/v 里/f 杀/v 出/vf 一个/mq 同 ¥�������.txt
143 n 练/v 琴/n ，/wd 弹/v 那/rzv 首/m 简单/a 无比/z 却/d 是/vshi 铿锵有力/al 的/ude1 《/wkz 铲/v 东/f 铲/v 东/f 铲/v 》/wky 。/wj 不/ ¥�������.txt
144 路/n 无处/d 可/v 待/vi ，/wd 只好/d 坐/v 在/p 门槛/n 上/f ，/wd 百无聊赖/vl 地/ude2 看/v 远处/s 的/ude1 夜空/n ，/wd 看/v 琴/n 房/n 对 ¥�������.txt
145 /n 对面/f 大片/n 黑黝黝/z 的/ude1 草地/n ，/wd 和/cc 草地/n 上/f 三三两两/al 卧/vi 着/uzhe 的/ude1 、/wn 梅/ng 教授/n 从/p 南京/ns 千辛 ¥�������.txt
146 两两/al 卧/vi 着/uzhe 的/ude1 、/wn 梅/ng 教授/n 从/p 南京/ns 千辛万苦/nl 带来/v 的/ude1 荷兰/nsf 花/n 奶牛/n 。/wj 偶尔/d 我/rr 停/v ¥�������.txt
147 堂/n 时/ng ，/wd 他/rr 不/d 知道/v 触发/v 了/ule 什么/ry ，/wd 没头没脑/al 来/vf 了/ule 一/m 句/q ：/wm "/wyz 你/rr 弹/v 得/ude3 ¥�������.txt
148 /n 。/wj
之后/f 的/ude1 好几/m 天/qt ，/wd 我/rr 对/p 他/rr 耿耿于怀/vl ，/wd 坚决/ad 不/d 跟/p 他/rr 说/v 一/m 句/q 话/n 。/wj 这 ¥�������.txt
149 de1 下巴/n 。/wj
这个/rz 念头/n 让/v 我/rr 无比/z 兴奋/a ，/wd 不知不觉/dl 我/rr 开始/v 认真/a 地/ude2 上课/vi ，/wd 很/d 勤奋/a 地/ud ¥�������.txt
150 1 萧瑟/z 。/wj 落叶/n 乔木/nrf 留下/v 了/ule 满/a 地/n 的/ude1 金碧辉煌/al 之后/f ，/wd 几乎/d 一夜间/t 变成/v 了/ule 光/n 裸/ag 的/ude ¥�������.txt
151 几乎/d 一夜间/t 变成/v 了/ule 光/n 裸/ag 的/ude1 流浪汉/n ，/wd 有气无力/al 地/ude2 摇晃/v 着/uzhe 枝头/n 仅/d 剩/v 的/ude1 几/m 片/q ¥�������.txt
152 n 。/wj
这/rzv 一/m 年/qt 的/ude1 年底/t ，/wd 前方/s 战事/n 不容乐观/vl 。/wj 日军/n 由/p 海路/n 登陆/vi 攻陷/v 广州/ns ，/wd 据说/v ¥�������.txt
153 退/vi 告/v 全国/n 军民/n 书/vg 》/wky ，/wd 誓言/n 全国/n 人民/n 万众一心/vl ，/wd 抗战/vi 到底/vi ，/wd 决不/d 投降/v 。/wj
我/rr 姐姐/ ¥�������.txt
154 /v 时/ng 向/p 天皇/n 狂言/n 三/m 个/q 月/n 占领/v 中国/ns ，/wd 目前过去/t 多/m 久/a 了/y ？/ww 一/m 年/qt 零三/m 个/q 月/n 了/y ！/wt ¥�������.txt
155 e1 肌肉/n 上/f ，/wd 晦涩/an 而/cc 干/v 糙/a 。/wj 我/rr 在/p 白天夜晚/t 总是/d 饥饿/n ，/wd 渴望/v 有/vyou 更/d 多/a 的/ude1 荤腥/n ¥�������.txt
156 n 写/v 作业/vi ，/wd 嘴巴/n 嗫/n 嚅/x 着/vi 背/v 英语/nz ，/wd 昏天黑地/al 应付/v 考试/vn ，/wd 除了/p 我/rr 娘/n ，/wd 没/v 人/n 能/v ¥�������.txt
157 娘/n 夸奖/v 他/rr 懂得/v 要/v 好/a ，/wd 有/vyou 股子/q "/wyz 磨杵成针/vl "/wyy 的/ude1 劲儿/n 。/wj 还/d 说/v 他/rr 在/p 我们/rr ¥�������.txt
158 里/s ，/wd 一/m 准/a 是/vshi 砌/v 墙/n 的/ude1 砖头/n ——/wp 后来居上/vl ，/wd 不/d 信/v 走着瞧/vl 。/wj 我们/rr 都/d 明白/a 娘/n 说/ ¥�������.txt
159 de3 很/d 成功/a ，/wd 校/ng 里/f 校外/s 人心/n 鼓/v 荡/v ，/wd 热气腾腾/vl 。/wj 到/p 圣诞节/t 的/ude1 时候/n ，/wd 社团/n 活跃/a 分子/n ¥�������.txt
160 /t 的/ude1 时候/n ，/wd 社团/n 活跃/a 分子/n 们/k 更/d 是/vshi 跃跃欲试/vl ，/wd 各/rz 校/ng 都/d 攒/v 足/a 劲儿/n 要/v 借/v 着/uzhe ¥�������.txt
161 1 模样/n 跟/p 他们/rr 家/n 的/ude1 爱尔兰/nsf 良种/n 鸡/n 简直/d 一模一样/al ，/wd 都/d 是/vshi 那种/r 目/ng 不/d 斜视/v 气势/n 逼/v 人/ ¥�������.txt
162 j ./wj ./wj "/wyy 一/m 开口/vi ，/wd 美妙/a 的/ude1 音色/n 千回百转/vl ，/wd 楼上/s 楼下/s 的/ude1 邻居/n 们/k 寂静/a 无/v 声/ng ， ¥�������.txt
163 。/wj 每/rz 回/v 排练/v ，/wd 演员/n 们/k 唱/v 念/v 做/v 打/v 一丝不苟/vl ，/wd 加上/v 锣鼓/n 铙/nr1 钹/n 京胡/n 响/a 板/n 什么/ry ，/ ¥�������.txt
164 咣/o 啷/x 很/d 是/vshi 热闹/a 。/wj 看/v 排练/v 的/ude1 观众/n 人山人海/nl ，/wd 比/p 正式/ad 演

出/v 还要/d 火爆/a 。/wj 我/rr 跟/v 着/¥�������.txt
165 排练/v 时/ng 一直/d 藏/v 着/uzhe 掖/v 着/uzhe 不/d 让/v 人/n 先睹为快/vl ，/wd 弄/v 得/ude3 大家/rr 心急火燎/al ，/wd 恨/v 不/d 能/v ¥�������.txt
166 着/uzhe 不/d 让/v 人/n 先睹为快/vl ，/wd 弄/v 得/ude3 大家/rr 心急火燎/al ，/wd 恨/v 不/d 能/v 睡/v 一夜/n 就/d 能/v 看到/v 海报/n 张贴 ¥�������.txt
167 /d 搞/v 就/d 搞/v 了/ule 个/q 大/a 的/ude1 ，/wd 要/v 排演/v 莎士比亚/nrf 的/ude1 《/wkz 仲夏夜/t 之/uzhi 梦/n 》/wky 。/wj 因为/p ¥�������.txt
168 派/nms 了/ule 一个/mq 仙/ng 后/f 的/ude1 角色/n ，/wd 正在/d 心满意足/vl 地/ude2 盘算/v 她/rr 是/vshi 披散/v 着/uzhe 头发/n 上台/vi ¥�������.txt
169 是/vshi 好/a 还是/d 坏/a 。/wj
沈天/nr 路/n 屋里/s 屋/n 外/f 进进出出/v 地/ude2 帮/v 我/rr 娘/n 准备/vn 晚饭/n ，/wd 一会儿/mq 拿/v ¥�������.txt
170 就/d 又/d 开心/a 起来/vf ，/wd 在/p 台上/s 很/d 卖力/a 地/ude2 拿腔拿调/dl ，/wd 倒是/d 精彩/a 。/wj
我/rr 也/d 挺/d 失落/v 的/ude1 ¥�������.txt
171 /vf 了/y ，/wd 不/d 出场/vi 的/ude1 时候/n ，/wd 小伙伴/n 们/k 叽叽喳喳/o 像/v 群/q 麻雀/n 子/ng ，/wd 别提/v 多/m 热闹/a 。/wj 不过/c ¥�������.txt
172 a 段/q 台词/n 要/v 在/p 台上/s 说/v ，/wd 别的/rzv 小孩/n 只能/v 摇头晃脑/vl 在/p 我/rr 身后/f 当/v 背景/n 。/wj 嗯/e ，/wd 说/v 真话/n ¥�������.txt
173 张开/v ，/wd 灯光/n 一/d 打/v ，/wd 油漆/n 闪闪/z 发亮/vi ，/wd 马马虎虎/z 也/d 混/v 得/ude3 过去/vf 了/y 。/wj
我/rr 姐/n 笑话/n 我/ ¥�������.txt
174 么着/ryv ？/ww 沈天路/ns 顶/v 上去/vf 了/y 。/wj 是/vshi 他/rr 自告奋勇/vl 请求/v 的/ude1 。/wj
我/rr 哥/n 我/rr 姐/n 都/d 没/d 想到/ ¥�������.txt
175 a ：/wm 早/ad 知道/v 这样/rzv 的/ude1 话/n ，/wd 我/rr 干吗/v 辛辛苦苦/z 背/v 那些/rz 台词/n 呢/y ？/ww 还/d 白/d 让/v 沈天/nr 路/n 笑 ¥�������.txt
176 d 又/d 哐/o 里/f 哐啷/o ，/wd 竹竿/n 人/n 一样/uyy ，/wd 尤其/d 冬日清早/t 上学/vi ，/wd 寒风/n 飕飕/o 地/ude2 灌/v 进/vf 衣襟/n ，/wd ¥�������.txt
177 时间/n 我们/rr 每天/r 走/v 在/p 路上/s 都/d 是/vshi 目光/n 炯炯/z 东张西望/vl ，/wd 渴望/v 路边/s 看见/v 一个/mq 瞎子/n 或者/c 跛子/n ，/wd ¥�������.txt
178 。/wj 但是/c 在/p 行善/vi 这/rzv 件/q 事/n 上/f ，/wd 小学生/n 无论如何/dl 争/v 不过/c 中学生/n 。/wj 比如/v 我/rr 姐/n 她们/rr 在/p 学校 ¥�������.txt
179 rzv 几/m 天/qt ，/wd 我/rr 哥/n 看/v 够/v 了/ule 医院/n 里/f 人间地狱/n 一般/uyy 的/ude1 悲惨/a 情景/n ，/wd 每天/r 回家/vi 都/d 吃/v ¥�������.txt
180 。/wj 石板/n 地/ude2 滑/v ，/wd 水桶/n 沉/v ，/wd 老太太/n 们/k 颤颤巍巍/z 拎/v 水/n 走路/vi 的/ude1 样子/n 让/v 人/n 提/v 着/uzhe 心/ ¥�������.txt
181 嘛/y ，/wd 提/v 水/n 要不得/vl ，/wd 你/rr 看看/v 你/rr ，/wd 小可怜儿/n ，/wd 麻/n 秆/ng 儿/ng 细/a 的/ude1 膀子/n ，/wd 啷/x 个/q ¥�������.txt
182 砖/n 缝/n ，/wd 每/rz 根/n 木/ng 栏杆/n ，/wd 都/d 擦/v 到/v 一尘不染/vl 。/wj
我/rr 娘/n 说/v ：/wm "/wyz 女/b 大/a 十八/m 变/v ¥�������.txt
183 ule 我/rr 娘/n 也/d 不/d 懂/v ，/wd 她/rr 就/d 是/vshi 个/q 家庭妇女/nl 嘛/y 。/wj
开春/t 后/f ，/wd 童子军/n 还/d 搞/v 了/ule 一/ ¥�������.txt
184 /a 学生/n 来说/uls ，/wd 称得上/v 壮举/n 。/wj
出发/vi 的/ude1 那天早晨/t ，/wd 阵势/n 很/d 大/a ，/wd 排/v 了/ule 浩浩荡荡/al 的/ude1 ¥�������.txt
185 vi 的/ude1 那天早晨/t ，/wd 阵势/n 很/d 大/a ，/wd 排/v 了/ule 浩浩荡荡/al 的/ude1 长/a 队/n ，/wd 鼓乐/n 齐鸣/vi ，/wd 大家/rr 扯/v ¥�������.txt
186 rr 扯/v 开/v 喉咙/n 高唱/v ：/wm "/wyz 我们/rr ，/wd 是/vshi 中华民族/n 的/ude1 少年/n 兵/n ，/wd 年纪/n 虽/c 小/a ，/wd 志气/n 高/a ¥�������.txt
187 /ude1 男生/n 拉肚子/v ，/wd 路上/s 没/d 找到/v 厕所/n ，/wd 又/d 不好意思/a 请假/vi 出列/v 就地/d 解决/v ，/wd 实在/d 憋/v 不/d 住/vi ，/w ¥�������.txt
188 爸爸/n 在/p 重庆/ns 政府/n 当/p 参议员/n ，/wd 本人/rr 一/m 副/q 弱不禁风/al 的/ude1 瓷/ng 娃娃/n 模样/n ，/wd 走/v 了/ule 不/d 到/v 十 ¥�������.txt
189 n 和/cc 白糖/n 的/ude1 炒/v 熟/a 的/ude1 麦粉/n 。/wj 要不是/c 野营拉练/vl ，/wd 我/rr 不/d 可能/v 一下子/mq 拥有/v 这么/rz 多/a 好吃/a ¥�������.txt
190 塞/v 进/vf 口中/s 。/wj 怕/v 老师/n 发现/v ，/wd 还/d 不/d 敢/v 大张旗鼓/dl 地/ude2 嚼/v ，/wd 先/d 拿/v 唾沫/n 洇/vi 湿/a ，/wd 再/d ¥�������.txt
191 q 没/d 吃/v 过/uguo 这么/rz 好吃/a 的/ude1 东西/n 。/wj 真是/d 妙不可言/vl ，/wd 简直/d 让/v 人/n 幸福/an 到/v 头晕/vi 。/wj
到/v 了/ ¥�������.txt
192 /wj 所有/b 人/n 都/d 坐/v 在/p 路边/s 野餐/n 时/ng ，/wd 我/rr 大大方方/z 地/ude2 打开/v 干粮/n

袋/ng ，/wd 把/pba 咸鸭蛋/n 分/qt 给/v ¥�������.txt
193 我们/rr 的/ude1 蚕食/vn 速度/n ，/wd 那/rzv 一/m 袋子/q 东西/n 无论如何/dl 都/d 剩/v 不/d 到/v 第二/m 天/qt 早上/t 。/wj
那/rzv 一夜/ ¥�������.txt
194 wd 不/d 怀/v 好意/n 地/ude2 瞪视/v 着/uzhe 我/rr ，/wd 仿佛/d 随时随地/dl 会/v "/wyz 唰/n "/wyy 地/ude2 伸/v 出/vf 一/m 只/q 手/ ¥�������.txt
195 这样/rzv 服装/n 统一/vn 又/c 队列/n 整齐/a 的/ude1 小学生/n ，/wd 三三两两/al 走/v 出来/vf 看热闹/vi 。/wj 我们/rr 趁机/d 列队/vi 高唱/v ：/ ¥�������.txt
196 中国/ns 一定/d 强/a ！/wt 你/rr 看/v 那/rzv 八百/m 壮士/n ，/wd 孤军奋战/vl 东/f 战场/n ./wj ./wj ./wj ./wj ./wj ./wj "/wyz 还 ¥�������.txt
197 的/ude1 晨雾/n 中/f 灰/ag 突/vg 突/vg 一/m 团/q ，/wd 显得/v 死气沉沉/al 。/wj
接/v 下去/vf 我们/rr 开始/v 参观/v 天王/n 殿/ng 、/wn ¥�������.txt
198 拜/v 了/ule 一下/mq 弥勒佛/n 、/wn 四/m 大/a 天王/n ，/wd 还有/v 龇牙咧嘴/vl 面相/n 狰狞/a 的/ude1 十八罗汉/nl 。/wj 我/rr 从小/d 就/d 不/ ¥�������.txt
199 n 四/m 大/a 天王/n ，/wd 还有/v 龇牙咧嘴/vl 面相/n 狰狞/a 的/ude1 十八罗汉/nl 。/wj 我/rr 从小/d 就/d 不/d 喜欢/vi 去/vf 佛/n 庙/n ，/wd ¥�������.txt
200 。/wj 但是/c 返程/v 的/ude1 路上/s 再/d 也/d 保持/v 不/d 了/ule 整齐划一/vl 的/ude1 队形/n ，/wd 有/vyou 脚/n 上/f 打/v 泡/v 一/m 瘸/ ¥�������.txt
201 you 早早/d 吃/v 完/vi 了/ule 干粮/n 然后/c 饿/v 着/uzhe 肚子/n 无精打采/vl 的/ude1 ，/wd 不/d 到/v 二百/m 人/n 的/ude1 队伍/n ，/wd ¥�������.txt
202 /vl 的/ude1 ，/wd 不/d 到/v 二百/m 人/n 的/ude1 队伍/n ，/wd 拖拖拉拉/z 竟然/d 绵延/vi 了/ule 几/m 里/q 路/n 。/wj 负责/v 收容/v 的/u ¥�������.txt
203 心/a 了/y 。/wj
范/nr1 伯伯/n 一/m 家/q 都/d 对/p 小/a 头虱/n 手足无措/al ，/wd 范玛丽/nrf 特地/d 跑/v 上/vf 楼/n ，/wd 向/p 我/rr 娘 ¥�������.txt
204 /vf 楼/n ，/wd 向/p 我/rr 娘/n 讨教/v 办法/n 。/wj 我/rr 娘/n 斩钉截铁/al 告诉/v 她/rr ：/wm "/wyz 先/d 把/pba 娃儿/n 的/ude1 头发/ ¥�������.txt
205 /ude1 。/wj 那/rzv 时候/n 的/ude1 人/n ，/wd 真的/d 是/vshi 稀里糊涂/z 地/ude2 活/v ，/wd 稀里糊涂/z 地/ude2 死/v 。/wj
后来/t 范/ ¥�������.txt
206 /ude1 人/n ，/wd 真的/d 是/vshi 稀里糊涂/z 地/ude2 活/v ，/wd 稀里糊涂/z 地/ude2 死/v 。/wj 后来/t 范/nr1 伯伯/n 受/v 我/rr 娘/n 启 ¥�������.txt
207 /n 的/ude1 味道/n 也/d 难/ad 闻/v ，/wd 熏/v 得/ude3 我们/rr 五脏六腑/nl 翻腾/vi ，/wd 碰上/v 刚刚/d 吃/v 饱/a 了/ule 肚子/n ，/wd 简 ¥�������.txt
208 ule 一/m 只/q 黑乎乎/z 、/wn 毛茸茸/z 的/ude1 小/a 东西/n ，/wd 七嘴八舌/vl 兴奋/v 到/v 不行/a 。/wj
饼子/n 也/d 不/d 要/v 吃/v 了/y ，¥�������.txt
209 条/q 浅/a 灰色/n 开司米/n 的/ude1 薄/a 围巾/n 。/wj 这/rzv 身/q 不伦不类/al 的/ude1 打扮/n 真是/d 逗/v ，/wd 然而/c 放在/v 风度翩翩/vl 的/ ¥�������.txt
210 v 身/q 不伦不类/al 的/ude1 打扮/n 真是/d 逗/v ，/wd 然而/c 放在/v 风度翩翩/vl 的/ude1 陶/nr1 伯伯/n 身上/s ，/wd 非但/c 不/d 违/vg 和/cc ¥�������.txt
211 s ，/wd 非但/c 不/d 违/vg 和/cc ，/wd 还/d 透/v 着/uzhe 点/n 落拓不羁/vl 的/ude1 帅/nr1 劲儿/n 。/wj
小/a 熊/nr1 崽/n 真是/d 藏民/ ¥�������.txt
212 也/d 没/d 想到/v 这/rzv 东西/n 养/v 大/a 了/ule 会/v 是/vshi 定时炸弹/nl 。/wj
整个/b 四月/t 和/cc 五月/t ，/wd 熊/nr1 崽/n 和/cc ¥�������.txt
213 和/cc 五月/t ，/wd 熊/nr1 崽/n 和/cc 我们/rr 相处/vi 得/ude3 其乐融融/al 。/wj
一/m 开始/v 大家/rr 争相/d 喂/v 食/ng ，/wd 荤腥/n 瓜 ¥�������.txt
214 。/wj
一/m 开始/v 大家/rr 争相/d 喂/v 食/ng ，/wd 荤腥/n 瓜果/n 一股脑儿/dl 都/d 上/f ，/wd 熊/nr1 崽/n 吃/v 伤/v 了/y ，/wd 拉稀/vi ¥�������.txt
215 ，/wd 拉稀/vi ，/wd 掉/v 毛/nr1 ，/wd 眼角/n 糊/v 屎/n ，/wd 萎靡不振/vl ，/wd 要/v 不/d 是/vshi 我们/rr 央求/v 畜牧/n 系/v 的/ude1 ¥�������.txt
216 t 陶/nr1 伯伯/n 召集/v 全院/n 小孩子/n 开会/vi ，/wd 跟/p 大家/rr 约法三章/vl ：/wm 每/rz 家/n 负责/v 喂/v 食/v 一/m 天/qt ，/wd 投/v 喂 ¥�������.txt
217 r ：/wm "/wyz 看见/v 没有/v ？/ww 一/m 家/q 一/m 天/qt ，/wd 各负其责/vl ，/wd 喂/v 得/ude3 不好/a ，/wd 全民/n 声讨/v ！/wt "/wyz ¥�������.txt
218 多/m 大/a 的/ude1 祸/n 。/wj
这/rzv 事/n 一/m 出/vf ，/wd 家家户户/nl 都/d 觉得/v 害怕/v ，/wd 小/a 点/qt 的/ude1 孩子/n 从此/d 再 ¥�������.txt
219 们/rr ，/wd 把/pba 熊/n 送/v 去/vf 哪里/rys 了/y ？/ww 对方/n 支支吾吾/z ，/wd 始终/d 不/d 肯/v

透露/v 确切/a 的/ude1 去向/n 。/wj 范舒 ¥�������.txt
220 们/rr 二/m 楼/n 的/ude1 西北/s 角/n 上/f ，/wd 住/vi 着/uzhe 赫赫有名/vl 的/ude1 物理学家/n 徐方训/nr ，/wd 就/d 是/vshi 那个/rz 被/p ¥�������.txt
221 ude1 西北/s 角/n 上/f ，/wd 住/vi 着/uzhe 赫赫有名/vl 的/ude1 物理学家/n 徐方训/nr ，/wd 就/d 是/vshi 那个/rz 被/pbei 熊/nr1 宝宝/n ¥�������.txt
222 /d 早/a 他/rr 从/p 美国哥伦比亚/nsf 大学/n 毕业/v 后/f ，/wd 在/p 苏州东吴/ns 大学/n 任教/vi ，/wd 一九三七年/t 日本/nsf 军队/n 攻占/v 上海/ns ¥�������.txt
223 p 榴/ng 园/ng 大院/n 里/f 是/vshi 出/vf 了/ule 名/q 的/ude1 不修边幅/vl 。/wj
一九三七年/t 抗战/vi 开始/v 时/ng ，/wd 国军/nr2 的/ud ¥�������.txt
224 /vi ，/wd 起身/vi 叹息/vi ，/wd 在/p 屋里/s 转圈/vi 圈/v ，/wd 大步流星/dl 地/ude2 穿过/v 走廊/n 找/v 我/rr 爸/n ，/wd 高声/d 地/ude2 ¥�������.txt
225 /d 叹气/vi ，/wd 面色/n 凝重/z ：/wm "/wyz 国家/n 贫弱/a ，/wd 积重难返/vl 啊/y 。/wj "/wyz 徐/nr1 伯伯/n 拼命/d 摇头/vi ，/wd 眼泪/ ¥�������.txt
226 军/n 的/ude1 飞机/njtgj 居然/d 深入/v 内地/s ，/wd 频繁/ad 在/p 重庆成都/ns 扔下/v 炸弹/n 。/wj 战火/n 蔓延/vi ，/wd 整个/b 中国/ns 陷入/v ¥�������.txt
227 /n 蔓延/vi ，/wd 整个/b 中国/ns 陷入/v 深渊/n ，/wd 无数/m 家庭/n 颠沛流离/vl ，/wd 那/rzv 真是/d 一/m 段/q 特别/d 绝望/a 的/ude1 日子/n ¥�������.txt
228 书斋/n 了/y ，/wd 他/rr 联合/v 了/ule 坝上/s 的/ude1 几/m 位/q 物理学家/n 和/cc 化工/n 学/v 家/q ，/wd 成立/vi 起/vf 一个/mq "/wyz 技 ¥�������.txt
229 p 抗战/vi 的/ude1 意义/n 讲/v 到/v 国人/n 的/ude1 责任/n ，/wd 滔滔不绝/vl 讲/v 了/ule 一个/mq 小时/n 。/wj 老/a 军阀/n 居然/d 感动/v 到 ¥�������.txt
230 珍藏/v 的/ude1 宝贝/n 双手/n 奉/v 上/f ，/wd 并且/c 言/vg 明/ag 分文不取/vl 。/wj
原料/n 收集/v 得/ude3 差不多/al 时/ng ，/wd 徐/nr1 ¥�������.txt
231 从/p 他/rr 的/ude1 老/a 同学/n 手中/s 觅/vg 得/ude3 一/m 本/q 马瑟尔氏/nrf 的/ude1 《/wkz 高级/a 火药/n 学/v 》/wky 。/wj 这/rzv 本 ¥�������.txt
232 /rzv 本/q 书/n 对/p 他/rr 的/ude1 研制/vn 工作/vn 可/v 算是/v 如虎添翼/vl 。/wj 用/p 徐/nr1 伯伯/n 自己/rr 的/ude1 话/n ，/wd 现在/t ¥�������.txt
233 用/p 徐/nr1 伯伯/n 自己/rr 的/ude1 话/n ，/wd 现在/t 是/vshi 万事俱备/vl 只/d 欠/v 东风/n 了/y 。/wj 东风/n 是/vshi 什么/ry 呢/y ？/ ¥�������.txt
234 楼/n 的/ude1 走廊/n 上/f ，/wd 远远/d 看/v 着/uzhe 炉/ng 口/n 忽明忽暗/vl 的/ude1 火焰/n ，/wd 看/v 着/uzhe 徐/nr1 伯伯/n 和/cc 他/ ¥�������.txt
235 wd 早日/d 研制/v 出/vf 能/v 把/pba 日本/nsf 人/n 炸/v 得/ude3 人仰马翻/al 的/ude1 火药/n 。/wj 我们/rr 每个/r 人/n 都/d 想/v 过去/vf ¥�������.txt
236 争抢/v 打架/vi ，/wd 怒/vg 目/ng 圆/vg 瞪/v ，/wd 摆/v 出/vf 你死我活/al 的/ude1 架势/n ，/wd 喉咙/n 里/f 发出/v 可怕/a 的/ude1 低/a¥�������.txt
237 睡/v 在/p 走廊/n 上/f ，/wd 让/v 蚊子/n 咬/vi 得/ude3 我们/rr 千疮百孔/vl 。/wj 我/rr 特别/d 心疼/v 我/rr 的/ude1 小/a 弟弟/n ，/wd ¥�������.txt
238 我/rr 的/ude1 小/a 弟弟/n ，/wd 他/rr 那/rzv 张/q 小/a 脸上/s 密密麻麻/z 布满/v 了/ule 蚊虫/n 叮/v 咬/vi 的/ude1 红包/n ，/wd 看上去/v ¥�������.txt
239 时候/n 不/d 懂得/v 抓/v ，/wd 只/d 晓得/v 哭/v ，/wd 哭/v 到/v 声嘶力竭/dl 气/n 都/d 喘/v 不/d 上来/vf ，/wd 真/d 叫/vi 人/n 崩溃/vi ¥�������.txt
240 ！/wt "/wyz
男生/n 苦/v 了/ule 脸/n 坐下/vi ，/wd 却/d 又/d 坐立不安/al ，/wd 看看/v 左边/f 的/ude1 窗外/s ，/wd 再/d 看看/v 右边/f ¥�������.txt
241 /m 响/q ，/wd 弹/v 到/v 地上/s 。/wj 全/a 教室/n 的/ude1 人/n 哈哈大笑/al 。/wj
自从/p 我们/rr 来到/v 华西/ns 坝上/s ，/wd 还/d 没有/d¥�������.txt
242 d 记/v 吃/v 不/d 记/v 打/v 的/ude1 ，/wd 所以/c 那天/r 我们/rr 嘻嘻哈哈/z ，/wd 一点/mq 没/d 有/vyou 意识/n 到/v 地狱/n 之/uzhi 祸/n ¥�������.txt
243 老/a 校长/n 一/m 只/q 手/n 把/pba 衣角/n 撩/v 在/p 手里/s ，/wd 声嘶力竭/dl 地/ude2 在/p 走廊/n 里/f 跑/v 着/uzhe 喊/v 着/uzhe ，/wd ¥�������.txt
244 vf 了/y ，/wd 跳/vi 起来/vf 往/v 外/f 就/d 跑/v ，/wd 把/pba 桌椅板凳/n 带/v 得/ude3 东倒西歪/vl 。/wj 人/n 多/ad 拥挤/vi ，/wd 一些/ ¥�������.txt
245 来/vf 往/v 外/f 就/d 跑/v ，/wd 把/pba 桌椅板凳/n 带/v 得/ude3 东倒西歪/vl 。/wj 人/n 多/ad 拥挤/vi ，/wd 一些/mq 男生/n 干脆/d 从/p 窗 ¥�������.txt
246 跳/vi ，/wd 一/d 回头/d 看见/v 范/nr1 舒/vg 文/ng 穿着/n 裙子/n 手足无措/al 的/ude1 模样/n ，/wd 赶快/d 折返/v 身/ng ，/wd 抓住/v 她/rr ¥�������.txt
247 /wj
我们/rr 用/v 最/d 快/a 的/ude1 速度/n 越过/v 操场/n ，/wd 三三两两/al 躲/v 进/vf 树林/n ，/wd 大/a 口/n 地/ude2 喘气/vi 。/wj 范 ¥�������.txt
248 v 反/vi 身/ng 回去/v 捡/v ，/wd 恰/d 在/p 此时/r ，/wd 爆炸声/n 震耳欲聋/vl 又/d 排山倒海/bl 地/ude2 扑/v 了/ule 过来/vf ，/wd 我们/rr ¥�������.txt

249 回去/v 捡/v ，/wd 恰/d 在/p 此时/r ，/wd 爆炸声/n 震耳欲聋/vl 又/d 排山倒海/bl 地/ude2 扑/v 了/ule 过来/vf ，/wd 我们/rr 忙不迭/d 地/ude2 ¥�������.txt
250 没有/v 防空/vn 设施/n ，/wd 所以/c 日本/nsf 人/n 飞/vi 得/ude3 肆无忌惮/vl ，/wd 机身/n 压/v 到/v 很/d 低/a 很/d 低/a ，/wd 飞行员/n 端 ¥�������.txt
251 de1 翅膀/n 上/f ，/wd 那个/rz 鲜红/z 的/ude1 太阳/n 旗/n 标志/n 清晰可见/vl ，/wd 触目惊心/vl 。/wj
—/m 架/qv 又/d —/m 架/qv ，/wd ¥�������.txt
252 ，/wd 那个/rz 鲜红/z 的/ude1 太阳/n 旗/n 标志/n 清晰可见/vl ，/wd 触目惊心/vl 。/wj
—/m 架/qv 又/d —/m 架/qv ，/wd 飞机/njtgj 得意/a ¥�������.txt
253 死/a 的/ude1 狗/n ，/wd 血肉/n 模糊/a ，/wd 肝胆/n 肚/ng 肠/n 花花绿绿/z 流/v 了/ule —/m 地/n 。/wj 所/usuo 幸/vg 两/m 家/q 都/d ¥�������.txt
254 wyz
我/rr 挺/d 佩服/v 我/rr 娘/n ，/wd 虽说/c 她/rr 是/vshi 家庭妇女/nl ，/wd 不/d 识字/vi ，/wd 可/c 是/vshi 关键/n 时刻/d 能够/v ¥�������.txt
255 了/ule —/m 声/qv ：/wm "/wyz Shit/n ！/wt "/wyz
工程/n 半途而废/vl 。/wj 我/rr 哥/n 觉得/v 对不起/v 娘/n 和/cc 小弟/n ，/wd 转而 ¥�������.txt
256 /wd 看/v 起来/vf 像/v 是/vshi 大师/n 制作/v 的/ude1 几/m 个/q 现代主义/n 的/ude1 装饰品/n ，/wd —/m 副/q 意味深长/vl 的/ude1 样子/n 。 ¥�������.txt
257 v 的/ude1 几/m 个/q 现代主义/n 的/ude1 装饰品/n ，/wd —/m 副/q 意味深长/vl 的/ude1 样子/n 。/wj 有/vyou —/m 天/qt 生/v 炉子/n 找/v ¥�������.txt
258 烧/vi 。/wj 树枝/n 干/v 得/ude3 很/d 透/v ，/wd 烧/vi 起来/vf 噼噼啪啪/o ，/wd 还/d 真/d 是/vshi 好/a 使/v 。/wj
听/v 雨/n 茶馆/n ¥�������.txt
259 日本/nsf 注意/v 。/wj
我们/rr 学会/v 了/ule 在/p 空袭/vn 中/f 从容不迫/vl 地/ude2 生活/vi 和/cc 学习/vn 。/wj 有/vyou 几/m 堂/q 国文 ¥�������.txt
260 事/n 处处/d 总/d 要/v 压/v 我/rr —/m 头/q 。/wj
不过/c ，/wd 未雨绸缪/vl 果真/d 是/vshi 有用/a 的/ude1 ，/wd 在/p 这/rzv 一点/mq 上
¥�������.txt
261 ，/wd 然后/c 冷不防/d 地/ude2 俯冲/vi ，/wd 炸/v 我们/rr 一个/mq 措手不及/al 。/wj 在/p 那/rzv 种/q 时候/n ，/wd 防空/vn 警报/n 就/d 形同 ¥�������.txt
262 不及/al 。/wj 在/p 那/rzv 种/q 时候/n ，/wd 防空/vn 警报/n 就/d 形同虚设/vl 。/wj
我/rr 娘/n 当时/t 正/d 在/p 烧/vi 晚饭/n ，/wd 就手/ ¥�������.txt
263 胳膊/n ：/wm "/wyz 娘/n 你/rr 跟/p 我/rr 走/v ！/wt "/wyz
与此同时/c ，/wd 我/rr 拦腰/d 抱/v 起/vf 小弟/n ，/wd 我/rr 姐/n 已经/d¥�������.txt
264 小/a 素/dg 背/v 在/p 背/v 上/vf 。/wj 我们/rr 六/m 个/q 人/n 跌跌撞撞/z 地/ude2 下/vf 楼/n ，/wd 穿过/v 院落/n ，/wd 直奔/v 河/n 坎/ ¥�������.txt
265 探/v 身/ng 往来/vn 路/n 上/f 看/v ，/wd 看到/v 沈天路/ns 已经/d 不顾一切/nl 地/ude2 背/v 起/vf 了/ule 我/rr 娘/n ，/wd 身子/n 很/d 吃 ¥�������.txt
266 /m 次/qv 。/wj 我们/rr 趴/v 在/p 河/n 坎/ng 上/f ，/wd 能够/v 清清楚楚/z 看见/v 飞机/njtgj 肚皮/n 底下/f 那个/rz 投弹/v 的/ude1 舱口/n ¥�������.txt
267 /d 带/v 我们/rr 三/m 个/q ）/wky 领到/v 街/n 子/ng 上/f ，/wd 慷慨解囊/vl ，/wd 请/v 我们/rr 每/rz 人/n 吃/v 了/ule —/m 碗/q 辣/a ¥�������.txt
268 光/n 闪烁/v ，/wd 炫/vg 得/ude3 我们/rr 无法/v 睁/v 眼/n 。/wj 不一会儿/bl ，/wd 有/vyou 日本/nsf 飞机/njtgj 被/pbei 打/v 中/f ，/w ¥�������.txt
269 /n 倒/d 栽/v 葱/n 似的/uyy 坠落/v ，/wd 撞/v 到/v 地面/n ，/wd 惊天动地/vl 炸/v 响/a ，/wd 还/d 腾出/v 黑/a 红色/n 的/ude1 蘑菇云/n 。/ ¥�������.txt
270 我/rr 是/vshi 男孩子/n 。/wj
我/rr 又/d 羞/v 又/d 恼/v ，/wd 好不容易/dl 熬/v 到/p 周末/t ，/wd 回家/vi 头/m —/m 桩/q 事/n 就是/v 对 ¥�������.txt
271 手/n 上街/vi ，/wd 扯/v 了/ule —/m 块/q 那年/t 流行/v 的/ude1 阴丹士林/n 布/vg ，/wd 还有/v —/m 块/q 浅/a 蓝/a 竹布/n ，/wd 熬/v —/ ¥�������.txt
272 /n 酸/a 胀/a ，/wd 想/v 流泪/vi 。/wj
四/m 间/q 宿舍/n ，/wd 东西南北/nl 各/rz —/m 间/q ，/wd 团团/d 围/v 成/v 一个/mq 天井/n ，/wd
¥�������.txt
273 子/n 和/cc 黏/a 黏/a 虫/n 的/ude1 天堂/n 。/wj 黏/a 黏/a 虫/n 无处不在/bl ，/wd 那种/r 灰/n 黄色/n 布满/v 隐形/b 花纹/n 的/ude1 缓慢/ad ¥�������.txt
274 /v 的/ude1 那些/rz 廊/ng 柱/ng 和/cc 门窗/n ，/wd 便/d 总是/d 影影绰绰/al 若明若暗/vl ，/wd 令/v 人/n 疑惑/v 是否/v 有/vyou 怪物/n 藏身/ ¥�������.txt
275 1 那些/rz 廊/ng 柱/ng 和/cc 门窗/n ，/wd 便/d 总是/d 影影绰绰/al 若明若暗/vl ，/wd 令/v 人/n 疑惑/v 是否/v 有/vyou 怪物/n 藏身/vi 其中/rz ¥�������.txt

276 f 一个/mq 可怕/a 的/ude1 陷阱/n 。/wj 所以/c 我/rr 一般/ad 在/p 下午四点/t 钟/n 以后/f 就/d 拒绝/v 喝/vg 水/n ， /wd 实在/d 需要/v 起/vf ¥�������.txt
277 一个/mq 月/n 左右/m ， /wd 下午/t 放/v 了/ule 学/v ， /wd 我/rr 急急忙忙/z 奔/v 回/qv 宿舍/n 读/v 一/m 本/q 翻译/n 小说/n 。/wj 这/rzv ¥�������.txt
278 已经/d 在/p 我们/rr 房间/n 里/f 传阅/v 了/ule 一/m 圈/qv ， /wd 好不容易/dl 传/v 到/v 我/rr 的/ude1 手里/s 。/wj 而/cc 对面/f 宿舍/n 里 ¥�������.txt
279 ， /wd 确定/v 房间/n 里/f 没有/v 别的/rzv 女孩子/n 在/p ， /wd 才/d 小心翼翼/dl 跨/v 进/vf 门/n 边/k 。/wj
他/rr 真/d 是/vshi 长/a 得/u ¥�������.txt
280 /rr 有/vyou 点/qt 感动/v 。/wj
我/rr 抓/v 着/uzhe 发糕/nms 狼吞虎咽/vl 。/wj 说实话/vl ， /wd 自从/p 住校/vi ， /wd 饥饿/n 的/ude1 感 ¥�������.txt
281 。/wj 说实话/vl ， /wd 自从/p 住校/vi ， /wd 饥饿/n 的/ude1 感觉/n 无时无刻/dl 不/d 在/p 压倒/v 一切/rz 。/wj
"/wyz 橙子/n 唉/e ， /wd " ¥�������.txt
282 d 在/p 那/rzv 几/m 只/q 橘子/n 上/f ， /wd 便/d 无聊/a 地/ude2 东张西望/vl 。/wj 只/d 一/m 眼/q ， /wd 他/rr 已经/d 瞥见/v 了/ule 我/r ¥�������.txt
283 不/d 住/vi "/wyz 的/ude1 人/n ， /wd 憋/v 了/ule 一/m 肚子/q 稀奇古怪/al 的/ude1 事/n ， /wd 好不容易/dl 见到/v 沈天路/ns ， /wd 赶快/d ¥�������.txt
284 n ， /wd 憋/v 了/ule 一/m 肚子/q 稀奇古怪/al 的/ude1 事/n ， /wd 好不容易/dl 见到/v 沈天路/ns ， /wd 赶快/d 噼/n 里/f 啪/o 啦/y 竹筒/n 倒/v ¥�������.txt
285
"/wyz 可/v 你/rr 不/d 是/vshi 基督徒/n 。/wj "/wyz 他/rr 慢条斯理/vl 。/wj
"/wyz 我/rr 当然/d 不/d 是/vshi 基督徒/n 。/wj "/ ¥�������.txt
286 r 的/ude1 数学/n 月/n 考/v 只/d 得/v 了/ule 七/m 十分/d ， /wd 一心一意/dl 要/v 看/v 我/rr 出洋相/v 。/wj
我/rr 偏/d 不/d 让/v 他们/r ¥�������.txt
287 s 莫名/v 地/ude2 起/vf 了/ule 红晕/n ， /wd 神情/n 慌张/a ， /wd 三三两两/al 半/m 背/v 了/ule 身/ng ， /wd 咬/vi 耳朵/n ， /wd 辫/ng 梢/ ¥�������.txt
288 伙/n 呢/y ， /wd 有/vyou 这么/rz 多/a 的/ude1 女生/n 都/d 在/p 心慌意乱/vl 啊/y ！ /wt 这么/rz 一/m 想/v ， /wd 我/rr 可/v 骄傲/a 了/y ¥�������.txt
289 /rr 可/v 骄傲/a 了/y ， /wd 一路/mq 走/v 着/uzhe ， /wd 一路/mq 神气活现/vl 地/ude2 仰/v 着/uzhe 头/n ， /wd 眼睛/n 几乎/d 都/d 要/v 望 ¥�������.txt
290 了/y 。/wj 我/rr 实在/d 憋/v 不/d 住/vi ， /wd 弯/v 下/vf 腰/n 哈哈大笑/al 。/wj 沈天路/ns 被/pbei 我/rr 笑/v 得/ude3 实在/d 难为情/a ¥�������.txt
291 我/rr 笑/v 得/ude3 实在/d 难为情/a ， /wd 小声/d 地/ude2 、/wn 咬牙切齿/dl 地/ude2 说/v ： /wm "/wyz 你/rr 个/q 小/a 疯子/n ！ /wt " ¥�������.txt
292 c ， /wd 弹/v 你们/rr 先生/n 教/v 的/ude1 ？ /ww "/wyz
我/rr 一门心思/dl 要/v 显/v 摆/v ： /wm "/wyz 那/rzv 多/m 没意思/a 。/wj 才/ ¥�������.txt
293 n 的/ude1 乐谱/n 柜/ng 里/f 寻找/v 到/v 了/ule 俄国/ns 作曲家/n 柯萨科夫/nrf 的/ude1 这/rzv 首/m 钢琴曲/n 。/wj 这/rzv 原本/d 是/vshi ¥�������.txt
294
不过/c ， /wd 我/rr 那/rzv 时候/n 弹琴/vi 的/ude1 水平/n 跟/p 范舒文真/nr 是/vshi 没/v 法/b 比/n ， /wd 乐曲/n 一/m 开头/n ， /wd 勉强/ ¥�������.txt
295 1 学校/n 里/f 熬/v 过/vf 了/ule 一/m 周/qt ， /wd 我/rr 已经/d 迫不及待/dl 地/ude2 要/v 想/v 回家/vi ， /wd 想/v 看看/v 榴/ng 园/ng 里¥�������.txt
296 朋友/n ， /wd 因为/c 谁/ry 也/d 受/v 不/d 了/ule 她/rr 的/ude1 伶牙俐齿/bl 。/wj
跑/v 进/vf 院子/n ， /wd 三步并作两步/vl 地/ude2 蹦/v ¥�������.txt
297 ， /wd 轻盈/a ， /wd 安逸/an ， /wd 虽然/c 娴熟/a ， /wd 总/d 觉得/v 蜻蜓点水/vl 一样/uyy 飘/v 着/uzhe ， /wd 少/a 一股劲儿/n 。/wj 用/p 她/r ¥�������.txt
298 ， /wd 总/d 觉得/v 蜻蜓点水/vl 一样/uyy 飘/v 着/uzhe ， /wd 少/a 一股劲儿/n 。/wj 用/p 她/rr 妈妈/n 范玛丽/nrf 的/ude1 话/n 说/v ： /wm ¥�������.txt
299 。/wj 范玛丽/nrf 的/ude1 琴声/n 要/v 更加/d 丰满/a 激荡/vn ， /wd 一泻千里/vl 。/wj 这个/rz 弹琴/vi 者/k 明显/a 指法/n 生疏/a ， /wd 和声/n ¥�������.txt
300 n ， /wd 一个/mq 年轻/a 又/d 帅气/a 的/ude1 小伙子/n ， /wd 至多/d 二十四五/m 岁/qt 吧/y ， /wd 长/v 着/uzhe 一/m 双/q 跟/p 范舒文/nr 一样/¥�������.txt
301 e 应该/v 跟/p 勇敢/a 的/ude1 马克/n 打/v 个/q 招呼/v ， /wd 只/d 一心一意/dl 打量/v 他/rr 的/ude1 黄/nr1 头发/n 、/wn 蓝/a 眼睛/n 、/wn ¥�������.txt
302 nsf 乡村/ns 口音/n 的/ude1 英文/nz 。/wj
范/nr1 舒/vg 文/ng 善解人意/al 地/ude2 凑/v 到/v 我/rr 的/ude1 耳朵/n 上/f ： /wm "/wyz
¥�������.txt

303 娘/n 拧/v 衣服/n ，/wd 带/v 着/uzhe 肥皂/n 气味/n 的/ude1 水/n 滴滴答答/o 拧/v 在/p 走廊/n 砖/n 地上/s ，/wd 涌出/v 细小/a 闪光/n 的/ude ￥�������.txt
304 ，/wd 因为/p 片刻/m 之后/f ，/wd 马克/n 拿/v 着/uzhe 一/m 副/b 羽毛球拍/n 走/v 到/v 院子/n 里/f ，/wd 弯腰/vi 拿/v 棍子/n 画/v 出/vf 一 ￥�������.txt
305 yy 的/ude1 手/n 可/c 真/d 是/vshi 大/a 得/ude3 吓人/a ，/wd 羽毛球拍/n 抓/v 在/p 他/rr 手里/s ，/wd 轻飘飘/z 像/v 拈/v 着/uzhe 个/q ￥�������.txt
306 f 窜/vi 去/vf 。/wj
沈天/nr 路/n 趴/v 在/p 栏杆/n 上/f ，/wd 目不转睛/dl 地/ude2 往/p 下/f 看/v 。/wj 他/rr 脸上/s 的/ude1 神情/n ￥�������.txt
307 n 一个/mq 劲儿/n 给/p 她/rr 喂/v 球/n ，/wd 无奈/d 她/rr 还是/d 不堪一击/vl 。/wj
我/rr 捅/v 捅/v 沈/nr1 天/n 路/n 的/ude1 胳膊/n ， ￥�������.txt
308 m 天/qt 是/vshi 星期天/t ，/wd 范/nr1 舒/vg 文/ng 一早/t 就/d 挨家挨户/dl 通知/n ，/wd 她/rr 妈妈/n 晚上/t 要/v 请/v 邻居/n 们/k 聚餐/v ￥�������.txt
309 手/v 准备/vn ，/wd 每家/r 都/d 费尽/v 心思/n 做/v 了/ule 一个/mq 像模像样/al 的/ude1 大/a 菜/n 。/wj 我/rr 娘/n 做/v 的/ude1 是/vshi ￥�������.txt
310 r 就/d 差/a 帮/v 人/n 喂/v 饭/n 喂/v 菜/n 了/y 。/wj "/wyz
那天晚上/t 月光/n 特别/d 好/a ，/wd 风/n 特别/d 柔/a ，/wd 墙/n 外/f 的/ ￥�������.txt
311 1 问题/n ，/wd 马克/n 耸/vg 耸肩/v ，/wd 摊/ng 开/v 手/n ，/wd 面红耳赤/al 的/ude1 ，/wd 说/v 他/rr 可能/v 答/v 不/d 好/a 。/wj 几/m ￥�������.txt
312 他们/rr 在/p 中国/ns 战场/n 跟/p 日/b 机/ng 作战/vi ，/wd 更加/d 绘声绘色/al ，/wd 说/v 到/v 兴起/v ，/wd 还/d 起/vf 身/ng 离/v 座/q ， ￥�������.txt
313 n 。/wj
沈天路/ns 听/v 得/ude3 也/d 吃力/a ，/wd 但是/c 他/rr 全神贯注/dl ，/wd 眼睛/n 一直/d 盯/v 着/uzhe 马克/n 的/ude1 嘴/n ，/wd ￥�������.txt
314 吃/v 进/vf 肚子/n 里/f 。/wj 他/rr 那/rzv 晚/a 的/ude1 眼睛/n 前所未有/vl 地/ude2 亮/vi ，/wd 连/ulian 前额/n 、/wn 鼻尖/n 、/wn 嘴 ￥�������.txt
315 /wd 欢乐/a 时/ng ，/wd 悲痛/a 时/ng ，/wd 哀伤/an 时/ng ，/wd 随时随地/dl 都/d 可以/v 唱/v 起来/vf 。/wj 几/m 位/q 教授/n 们/k 从/p 《 ￥�������.txt
316 ，/wd 放开/v 喉咙/n ，/wd 激情/n 澎湃/vi 地/ude2 朗诵/v 了/ule 一段济慈/nr 的/ude1 名/q 篇/q 《/wkz 夜莺/n 颂/vg 》/wky 。/wj 陶/nr ￥�������.txt
317 篇/q 《/wkz 夜莺/n 颂/vg 》/wky 。/wj 陶/nr1 伯伯/n 是/vshi 英国牛津/ns 大学/n 毕业生/n ，/wd 他/rr 的/ude1 那/rzv 一/m 口/q 漂亮/a ￥�������.txt
318 桌子/n 板凳/n 抬/v 进/vf 主人家/n 中/f ，/wd 院子/n 扫/v 得/ude3 干干净净/z 。/wj
一切/rz 结束/v 后/f ，/wd 夜/tg 已经/d 深/a 了/y ，/w ￥�������.txt
319 是/vshi 一/m 盆/q 糨糊/n 。/wj
他/rr 看/v 我/rr 那/rzv 副/q 晕晕乎乎/z 的/ude1 傻/a 样/u ，/wd 有点/d 扫兴/a ，/wd 伸手/vi 从/p 裤/
￥�������.txt
320 g 了/ule 转/v 一个/mq 圈/n ，/wd 才/d 放/v 下/vf 。/wj 他/rr 真心诚意/dl 地/ude2 邀请/v 我/rr ：/wm "/wyz 橙子/n ，/wd 以后/f 我们/ ￥�������.txt
321 不/d 及格/vi ，/wd 那/rzv 就/p 洋相/n 出/vf 大/a 了/y 。/wj
战战兢兢/dl 了/ule 两/m 天/qt 。/wj 虽然/c 无/v 事/n 可/v 做/v ，/wd ￥�������.txt
322 /wd 从/p 早/ad 到/v 晚/tg 纹/ng 丝/n 不/d 乱/a 。/wj
她/rr 痛心疾首/vl 责怪/v 我/rr ：/wm "/wyz 黄/nr1 橙子/n ，/wd 真/d 真/d 要 ￥�������.txt
323 不/d 到/v 国文/n 课/n 就/d 一定/d 挂/v 科/n 啊/y 。/wj
"/wyz 下不为例/vl ！/wt "/wyz 她/rr 又/d 一/m 次/qv 严肃/v 了/ule 面孔/n ， ￥�������.txt
324 v 我/rr 黄/nr1 橙子/n 了/y 。/wj
拿/v 着/uzhe 成绩单/n ，/wd 欢天喜地/vl 回/v 了/ule 家/n ，/wd 才/d 上楼/vi 就/d 听/v 见/v 我/rr
￥�������.txt
325 "/wyz 你/rr 真/d 是/vshi 冬瓜/n 爬/v 在/p 葫芦/n 上/f ，/wd 胡搅蛮缠/vl 哩/y 。/wj 人家/rr 服务团/n 收/v 的/ude1 都/d 是/vshi 大学生 ￥�������.txt
326 ，/wd 谁/ry 是/vshi 人民/n 的/ude1 敌人/n 。/wj "/wyz
娘/n 一败涂地/al 。/wj 我/rr 姐/n 的/ude1 那/rzv 张/q 嘴/n ，/wd 不/d 让/ ￥�������.txt
327 /wd 干吗/v 还要/d 挖苦/v 我/rr 一下/mq ？/ww 我/rr 心里/s 实在/d 愤愤不平/vl 。/wj 后来/t 趁/p 她/rr 收拾/v 行李/n ，/wd 我/rr 把/pba 她 ￥�������.txt
328 q 脸/n 不/d 皱/v 成/v 个/q 柿饼/n 才/d 怪/a 。/wj
我/rr 姐/n 意气风发/vl 地/ude2 走/v 了/y 。/wj 头/m 两/m 天/qt 娘/n 还/d 念叨/v ￥�������.txt
329 纪/n 的/ude1 人/n 参加/v 社团/n ，/wd 一/d 感动/v ，/wd 差点儿/d 热泪盈眶/al 。/wj 待/vi 到/v 去/vf 了/ule 之后/f 才/d 发现/v ，/wd 原来 ￥�������.txt
330 /rr 耶稣/nrf 会/v 的/ude1 人/n ！/wt "/wyz
范舒文/nr 倒是/d 大大咧咧/z ：/wm "/wyz 你/rr 管/v 别人/rr 呢/y ，/wd 反正/d 都/d 是/v
￥�������.txt

331 /n 。/wj 教会/n 的/ude1 组织性/n 很/d 好/a ，/wd 分工/vd 合作/v 井井有条/al ：/wm 有人/r 理/n 麻绳/n ，/wd 有人/r 专事/d 裁剪/v ，/wd 有人 ¥�������.txt
332 来/vf 就/d 是/vshi 一/m 只/q 袖管/n ，/wd 看/v 得/ude3 我/rr 目瞪口呆/al 。/wj
回/v 家/q 告诉/v 我/rr 娘/n ，/wd 娘/n 说/v 她/rr ¥�������.txt
333 教堂/n 见识/n 一/m 回/qv 。/wj
我/rr 对/p 范/nr1 舒/vg 文/ng 信誓旦旦/vl 说/v ，/wd 一定/d 要/v 学会/v 踩/v 缝纫机/n 的/ude1 技术/n 。 ¥�������.txt
334 裹/n ，/wd 居然/d 就是/v 我/rr 要/v 的/ude1 东西/n ！/wt 我/rr 欣喜若狂/vl 地/ude2 送/v 到/v 范舒文/nr 家/n ，/wd 请/v 她/rr 转交/v 给 ¥�������.txt
335 /d 安稳/a 。/wj 范/nr1 舒/vg 文/ng 绣/v 出来/vf 的/ude1 字/n 端端正正/z ，/wd 干净/a 秀气/a ，/wd 我/rr 的/ude1 字/n 总是/d 歪/v 七/ ¥�������.txt
336 k 做成/v 前线/s 用品/n 总是/d 不/d 合适/a 吧/y ？/ww 大家/rr 都/d 七嘴八舌/vl 地/ude2 犯/v 了/ule 难/a 。/wj 我/rr 想/v 了/ule 个/q 好 ¥�������.txt
337 wd 路面/n 总/d 是/vshi 夜里/t 上冻/v ，/wd 白天/t 化冻/vi ，/wd 坑坑洼洼/bl 泥泞/n 不堪/v 。/wj 街/n 子/ng 上/f 出门/vi 闲逛/vi 的/ude1¥�������.txt
338 ，/wd 头巾/n 裹/v 脸/n ，/wd 棉袄/n 棉裤/n 棉鞋/n 穿/v 得/ude3 严严实实/z ，/wd 一/m 人/n 挎/v 一个/mq 敞/v 口/q 小/a 篮子/n ，/wd 街/ ¥�������.txt
339 又/d 不/d 实用/a 的/ude1 小/a 玩意/n 儿/ng 。/wj 我们/rr 都/d 不好意思/a 张口/vi 吆喝/v ，/wd 连/ulian 抬头/vi 看/v 人/n 的/ude1 勇气 ¥�������.txt
340 /wj 还有/v 拖/v 着/uzhe 鼻涕/n 的/ude1 顽皮/a 小/a 孩子/n 们/k 跑前跑后/vl ，/wd 拿/v 土块/n 砸/v 她/rr ，/wd 大喊大叫/vl ：/wm "/wyz ¥�������.txt
341 小/a 孩子/n 们/k 跑前跑后/vl ，/wd 拿/v 土块/n 砸/v 她/rr ，/wd 大喊大叫/vl ：/wm "/wyz 小/a 洋人/n ！/wt 快/d 看/v 小/a 洋人/n ！/wt¥�������.txt
342 时/ng ，/wd 她/rr 把/pba 一/m 头/q 金发/n 编成/v 辫子/n ，/wd 严严实实/z 地/ude2 兜/vi 进/vf 头巾/n 里/f ，/wd 还/d 特意/d 戴/v 一/m ¥�������.txt
343 交差/vi ，/wd 一个/mq 荷包/n 也/d 没/d 卖/v 出去/vf ，/wd 太/d 不好意思/a 了/y 。/wj 而且/c ，/wd 就/d 这样/rzv 无/v 功/n 而/cc 返/v ¥�������.txt
344 u 效果/n 。/wj "/wyz
我们/rr 连连/d 点头/vi ，/wd 佩服/v 到/v 无话可说/vl 。/wj
他/rr 有点/d 忸怩/a 地/ude2 抠/v 着/uzhe 手指头/n 上 ¥�������.txt
345 z 一个/mq 心里/s 有/vyou 主意/n 的/ude1 人/n 呢/y ？/ww 他/rr 不声不响/vl ，/wd 却/d 是/vshi 什么/ry 都/d 看/v 在/p 眼里/s ，/wd 什么 ¥�������.txt
346 wj
天/qt 已经/d 有/vyou 点/n 晚/tg 了/y ，/wd 即便/c 我们/rr 雄心勃勃/vl ，/wd 义卖/vn 活动/vn 也/d 得/v 第二/m 天/qt 开始/v 。/wj
¥�������.txt
347 qv 漂亮/a 的/ude1 白/a 纱/n ，/wd 远处/s 的/ude1 树木/n 河流/n 影影绰绰/al 。/wj 娘/n 猜/v 到/v 我们/rr 的/ude1 紧张/a ，/wd 让/v 我们 ¥�������.txt
348 们/rr 从/p 榴/ng 园/ng 出发/vi 时/ng ，/wd 是/vshi 一/m 支/q 意气风发/vl 的/ude1 小小/z 队伍/n 。/wj 最/d 前面/f 的/ude1 沈天路/ns 头 ¥�������.txt
349 着/uzhe 糨糊/n 碗/n 的/ude1 我/rr 。/wj 范/nr1 舒/vg 文/ng 左右开弓/vl 挎/v 着/uzhe 我/rr 和/cc 她/rr 的/ude1 两/m 只/q 货/n 篮 ¥�������.txt
350 尾巴/n --/wp 小/a 素/ag ，/wd 她/rr 坚持/v 要/v 去/vf ，/wd 可怜巴巴/z 求/v 了/ule 我/rr 一/m 早上/t 。/wj 我/rr 同意/v 她/rr 帮/v ¥�������.txt
351 wd 走/v 不/d 多/a 远/a 脖子/n 就/d 受不了/al 了/y 。/wj 我们/rr 当机立断/vl ，/wd 在/p 路边/s 卸/v 了/ule 货/n ，/wd 就地/d 摆开/v 阵势/ ¥�������.txt
352 /vi 了/ule 把/pba 桌子/n 放在/v 横幅/n 前/f ，/wd 我/rr 和/cc 范舒文一/nr 左/f 一/m 右/f 哼哈二将/n 似的/uyy 分列/v 两侧/f 。/wj 桌上/s ¥�������.txt
353 放在/v 横幅/n 前/f ，/wd 我/rr 和/cc 范舒文一/nr 左/f 一/m 右/f 哼哈二将/n 似的/uyy 分列/v 两侧/f 。/wj 桌上/s 的/ude1 绣品/n 也/d 摆/v ¥�������.txt
354 n 加/v 了/ule 金边/nsf 糊/v 成/v 的/ude1 收款/vn 箱/ng ，/wd 小巧玲珑/al ，/wd 四/m 四方/n 方/q ，/wd 太阳/n 一/m 照/ng ，/wd 明晃晃/ ¥�������.txt
355 /vf 大声/d 地/ude2 念/v 出/vf 对联/n 上/f 的/ude1 字/n ，/wd 啧啧称赞/vl 。/wj 几/m 个/q 小商贩/n 开口/vi 询/vg 价/n ，/wd 似乎/d 觉得 ¥�������.txt
356 i 。/wj 还有/v 一个/mq 肥/a 头/n 肥/a 脑/n 穿/v 马褂/n 的/ude1 中年男子/n ，/wd 拿/v 了/ule 一个/mq 烟袋/n ，/wd 硬是/d 给/v 了/ule 两 ¥�������.txt
357 r 说/v 。/wj
我们/rr 垂/v 手/n 恭立/nr2 ，/wd 兴奋/v 得/ude3 面红耳赤/al 。/wj
他/rr 俯身/v 跟/p 勤务兵/n 耳语/vi 一/m 句/q 。/wj 勤 ¥�������.txt
358 行/vi 渐/d 远/a ，/wd 消失/vi 在/p 路/n 的/ude1 尽头/f ，/wd 范舒文才/nr 反应/vi 过来/vf ，/wd 惊呼/v 一/m 声/qv ：/wm "/wyz 橙子/n¥�������.txt

359 ，/wd 烟袋/n 荷包/n 已经/d 卖/v 完/vi ，/wd 我们/rr 已经/d 在/p 一鼓作气/vl 准备/v 下面/f 一/m 轮/qv 的/ude1 义卖/vn 活动/vn 了/y ，/wd ¥�������.txt
360 d 邓/nr1 将军/n ，/wd 打/v 过/uguo 长沙/ns 保卫/v 战/ng ，/wd 赫赫有名/vl 的/ude1 抗战/vn 英雄/n ！/wt "/wyz
大/a 冬天/t 里/f ，/w ¥�������.txt
361 抗战/vn 英雄/n 。/wj "/wyz
他/rr 咬/vi 着/uzhe 嘴唇/n ，/wd 若有所思/vl 的/ude1 模样/n 。/wj
我/rr 忽然/d 想到/v 一个/mq 问题/n ：/ ¥�������.txt
362 /n 呢/y ，/wd 还/d 是/vshi 女儿/n ？/ww "/wyz
沈天/nr 路/n 目瞪口呆/al 地/ude2 看/v 着/uzhe 我/rr ，/wd 死活/d 都/d 不/d 明白/a
¥�������.txt
363 v 个/q 生意人/n 。/wj
那/rzv 一个/mq 寒假/t 中/f ，/wd 我们/rr 欲罢不能/vl 地/ude2 陷入/v 义卖/vi 疯狂/a 时/ng ，/wd 我/rr 姐/n 却/d ¥�������.txt
364 /ude2 陷入/v 义卖/vi 疯狂/a 时/ng ，/wd 我/rr 姐/n 却/d 一直/d 出门在外/vl ，/wd 实践/v 着/uzhe 另外/rz 一/m 种/q 抗战/vi 救国/vi 之/u ¥�������.txt
365 shi 不/d 少/a 。/wj 开学/vi 前/f 一/m 天/qt ，/wd 她/rr 才/d 风尘仆仆/vl 衣裳/n 褴褛/z 地/ude2 回/v 了/ule 家/n 。/wj 到/p 家/n 那天 ¥�������.txt
366 清水/n 煮/v 野菜/n 。/wj 也/d 看/v 到/v 医院/n 的/ude1 伤病员/n 缺医少药/nl 活生生/z 疼/v 死/v 。/wj 她/rr 说/v 着/uzhe 说/v 着/uzhe ¥�������.txt
367 /wj 她/rr 说/v 着/uzhe 说/v 着/uzhe 流/v 了/ule 泪/n ，/wd 忧心忡忡/vl 地/ude2 叹息/vi ：/wm "/wyz 中国/ns 太/d 苦/a 了/y ，/wd ¥�������.txt
368 z 放心/v ，/wd 中国/ns 还有/v 我们/rr 这/rzv 一代人/n 在/p ，/wd 前赴后继/vl 也/d 要/v 灭/v 了/ule 小/a 日本/nsf 。/wj "/wyz
沈天路/n ¥�������.txt
369 v 决/d 绝/ag ，/wd 让/v 我/rr 隐隐/z 觉得/v 不祥/z ，/wd 觉得/v 心惊胆战/al 。/wj
我/rr 说/v 不/d 好/a 。/wj 可/c 我/rr 当时/t 心里/s ¥�������.txt
370 i 不安/a 。/wj
第十二/m 章/q ·/w 恋爱/vi 中/f 的/ude1 女孩/n
周六下午/t ，/wd 我/rr 照例/d 拎/v 一个/mq 小/a 包袱/n 回家/vi 。/wj 包袱 ¥�������.txt
371 /ude1 小/a 粉蝶/n 也/d 来/vf 了/y ，/wd 连/ulian 鸟雀/n 都/d 欢欣鼓舞/vl ，/wd 嘤嘤/o 嗡嗡/o 一/m 世界/n 的/ude1 热闹/a 。/wj
我/rr ¥�������.txt
372 下/vf 短/a 一下/mq ，/wd 风铃/n 乱/d 摇/v 似的/uyy ，/wd 一发/d 不可收拾/al 。/wj
我/rr 猛地/d 转/v 过/uguo 身/ng ，/wd 望/v 着/uzh ¥�������.txt
373 我/rr 姐/n ，/wd 又/d 羞/v 又/c 气/n 。/wj 我/rr 气/v 她/rr 不声不响/vl 跟/v 着/uzhe 我/rr 走/v 了/ule 这么/rz 久/ng ，/wd 活生生/ ¥�������.txt
374 前面/f 出/vf 尽/vi 了/ule 洋相/n ，/wd 还/d 如此/rzv 地/ude2 乐不可支/vl 。/wj 如果/c 不/d 是/vshi 她/rr 身边/s 还/d 走/v 着/uzhe ¥�������.txt
375 帅气/a 的/ude1 男孩/n 儿/ng 啊/y ，/wd 连/ulian 我/rr 这种/r 粗枝大叶/al 的/ude1 小/a 女生/n 都/d 明白/v 他/rr 长/v 得/ude3 好看/a ¥�������.txt
376 有/vyou 话/n 慢慢/d 说/v ，/wd 不/d 急/a 。/wj "/wyz
我/rr 呼哧呼哧/o 地/ude2 ：/wm "/wyz 娘/n ，/wd 你/rr 想/v 不/d 想/v 知道/ ¥�������.txt
377 地/ude2 ：/wm "/wyz 你们/rr 两/m 个/q 打/v 小/a 不/d 就是/v 冤家对头/n 吗/y ？/ww 你/rr 姐/n 走/v ，/wd 你/rr 不/d 高兴/a ？/ww " ¥�������.txt
378 v 过/uguo 政府/n 嘉奖/vn 。/wj
我/rr 爸/n 静静地/z 听/v ，/wd 不置可否/vl ，/wd 不/d 说/v "/wyz 哦/e "/wyy ，/wd 也/d 不/d 说/v
¥�������.txt
379 觉得/v 好/a 还是/c 不/d 好/a 呢/y ？/ww 真/d 急/a 人/n 。/wj
此时此刻/nl ，/wd 我/rr 有/vyou 那么/rz 点/qt 同情/v 我/rr 姐/n 。/wj ¥�������.txt
380 /wd 偏/d 等/v 着/uzhe 我/rr 姐/n 领会/v 。/wj 大人/n 的/ude1 花花肠子/nl 就/d 是/vshi 多/a ，/wd 麻烦/an 。/wj
姐/n 想/v 去/vf ， ¥�������.txt
381 别人/rr 知道/v 了/ule 怎么/ryv 想/v ？/ww "/wyz
我/rr 爸/n 心平气和/vl 解释/v 给/p 娘/n 听/v ：/wm 现在/t 的/ude1 时代/n 要/v 讲究/
¥�������.txt
382 特意/d 把/pba 我/rr 姐/n 喊/v 到/v 身边/s 谈话/vi 。/wj 我/rr 断断续续/dl 听到/v 几/m 句/q ：/wm ./wj ./wj ./wj ./wj ./wj ./w ¥�������.txt
383 uzhe 绣花鞋/n ，/wd 走/v 起/vf 路/n 来/vf 像/v 鸭子/n 一样/uyy 摇摇摆摆/v 。/wj 我/rr 看/v 着/uzhe 她/rr 的/ude1 模样/n 想/v 笑/v ， ¥�������.txt
384 鸡/n ，/wd 还有/v 他/rr 家/n 从/p 云南/ns 带/v 回来/v 的/ude1 各种各样/bl 的/ude1 菌/n 干/v

。/wj 在/p 华西/ns 坝上/s 住/vi 了/ule ¥������.txt
385 /ude1 饭食/n ，/wd 一下子/mq 弄/v 得/ude3 我/rr 头昏/vi ，/wd 眼花缭乱/al 都/d 不/d 知道/v 在/p 哪个/ry 碗/n 里/f 下/vf 筷子/n 才/d 好 ¥������.txt
386 、/wn 灯影/n 牛肉/n 几/m 样/q 小/a 食/ng ，/wd 也/d 让/v 我/rr 惊喜万分/vl 。/wj 躺/v 上/vf 床/n 之后/f ，/wd 我/rr 一边/d 高高/z 地/u ¥������.txt
387 天天/d 都/d 能/v 吃/v 到/v 好/a 东西/n 。/wj "/wyz 我/rr 姐/n 咬牙切齿/dl 扑/v 上来/vf 揪/v 我/rr 的/ude1 嘴/n ，/wd 还/d 气/v 我/r ¥������.txt
388 ms 。/wj 端/v 到/v 手里/s ，/wd 我/rr 跟/p 我/rr 姐/n 忍不住/v 面面相觑/vl 。/wj 程渝/nr 生/v 偷偷/d 告诉/v 我们/rr 说/v ，/wd 他们/rr ¥������.txt
389 d 料/v 归/v 料/n ，/wd 石/ng 槽/ng 陶器/n 都/d 打扫/v 得/ude3 干干净净/z 。/wj 程渝生/nr 说/v ，/wd 他家/r 马/n 儿/k 最/d 多/a 的/ude ¥������.txt
390 那/rzv 几/m 天/qt ，/wd 马队/n 出门/vi 了/y ，/wd 马厩/n 里/f 空空荡荡/z ，/wd 倒是/d 门外/s 草场/n 上/f 拴/v 了/ule 一/m 匹/q 尚/nr1 ¥������.txt
391 十足/z 的/ude1 眼睛/n 滴溜溜/z 地/ude2 看/v 我们/rr ，/wd 一边/d 悠闲自在/al 地/ude2 甩/v 着/uzhe 长/a 尾巴/n ，/wd 漂亮/a 得/ude3 宛如 ¥������.txt
392 v 事/n ，/wd 不/d 停/vi 地/ude2 抬/v 起/vf 前/f 蹄/ng ，/wd 欢蹦乱跳/bl ，/wd 还/d 拿/v 脑袋/n 去/vf 拱/v 程渝生/nr 的/ude1 胳膊/n ¥������.txt
393 /v 。/wj
我/rr 赶快/d 在/p 旁边/f 插嘴/v ：/wm "/wyz 小孩子/n 可不可以/v 骑/v ？/ww "/wyz
程渝生/nr 说/v ：/wm "/wyz 可以/v 啊/y ¥������.txt
394 3 一/d 抬/v 手/n 就/d 能够/v 扯/v 下/vf 几/m 片/q 。/wj 我/rr 迫不及待/dl 地/ude2 要/v 想/v 打/v 马/n 飞跑/vi ，/wd 程/nr1 渝/b 生死 ¥������.txt
395 r1 渝/b 生死/n 活/v 抓/v 着/uzhe 缰绳/n 不/d 放/v ，/wd 一边/d 惊慌失措/vl 地/ude2 喊/v ：/wm "/wyz 小/a 妹/n 小妹/n 小妹/n ！/wt " ¥������.txt
396 各色/r 野花/n 在/p 山坡/n 上/f 随/v 风/n 摇曳/vi ，/wd 近/v 看/v 万紫千红/vl ，/wd 远/ad 看/v 却/d 隐/v 入/v 绿色/n 不/d 见/v 。/wj 阳光 ¥������.txt
397 de1 西南/s 战场/n 。/wj
我/rr 爸爸/n 心/n 痛/dg 至极/vi ，/wd 万般无奈/dl 地/ude2 送/v 走/v 他/rr 的/ude1 那些/rz 宝贝/n 学生/n 之后/¥������.txt
398 把/pba 这些/rz 年轻人/n 往/p 前线/s 送/v ？/ww 这/rzv 是/vshi 杀鸡取卵/vl 啊/y 老兄/n ！/wt 战争/n 再/d 残酷/a ，/wd 终归/d 有/vyou 结 ¥������.txt
399 n ！/wt 农业/n 一定/d 要/v 发展/v ！/wt 国/n 军/n 征兵/vn ，/wd 无论如何/dl 不/d 该/v 征/v 到/v 我们/rr 农学院/n 学生/n 的/ude1 头/n 上/ ¥������.txt
400 几/m 岁/qt 年纪/n ，/wd 碰上/v 机会/n ，/wd 一样/a 会上/t 战场/n 舍身取义/vl 。/wj "/wyz 我/rr 爸爸/n 嗫/n 嚅/x ：/wm "/wyz 道理/n ¥������.txt
401 点/qt 人才/n 不/d 容易/a ，/wd 将来/t 他们/rr 都/d 该/v 是/vshi 国家栋梁/nl 的/ude1 ，/wd 可惜/v 了/y ./wj ./wj ./wj ./wj ./wj ¥������.txt
402 喊/v 的/ude1 人/n ，/wd 听/v 的/ude1 人/n ，/wd 似乎/d 都/d 习以为常/vl 。/wj 沈天路/ns 能/v 干/v ，/wd 也/d 肯干/a ，/wd 家里/s 琐碎 ¥������.txt
403 v 。/wj 一/m 尺/q 多/a 高/a 的/ude1 水桶/n ，/wd 他/rr 能够/v 左右开弓/vl 地/ude2 拎/v 起来/vf ，/wd 一口气/d 奔/v 上/vf 楼梯/n ，/wd ¥������.txt
404 小伙子/n 的/ude1 模样/n ：/wm 宽/a 肩/n ，/wd 细/a 腰/n ，/wd 浓眉大眼/al ，/wd 目光/n 坚定/a ，/wd 说话/vi 有/vyou 分量/n ，/wd 也/d ¥������.txt
405 vi 起/vf 扬/vg 灰/ag ，/wd 一样/a 一样/uyy 都/d 做/v 得/ude3 像模像样/al 。/wj 我/rr 觉得/v 我们/rr 家/q 兄弟/n 姐妹/n 当中/f ，/wd 爸 ¥������.txt
406 给/p 他/rr 加/v 缝/n 的/ude1 两/m 只/q 大/a 口袋/n 里/f ，/wd 鼓鼓囊囊/z 不/d 知道/v 塞/v 了/ule 些/q 什么/ry 东西/n 。/wj 时不时/d 地/¥������.txt
407 个/rz 时候/n ，/wd 他/rr 显得/v 特别/d 专注/vi ，/wd 又/d 特别/d 犹豫不决/vl ：/wm 先/d 左右/v 看看/v ，/wd 再/d 退后/vi 两/m 步/qv 看看/ ¥������.txt
408 v 我/rr 爸/n 是/vshi 在/p 选种/vi ，/wd 要/v 在/p 麦地/n 里/f 数以万计/vl 的/ude1 麦穗/n 中/f 找/v 出/vf 一/m 株/q 超凡脱俗/al 的/ude ¥������.txt
409 麦地/n 里/f 数以万计/vl 的/ude1 麦穗/n 中/f 找/v 出/vf 一/m 株/q 超凡脱俗/al 的/ude1 群体/n 优胜者/n 。/wj
我/rr 爸/n 身后/f 亦步亦趋/vl ¥������.txt
410 株/q 超凡脱俗/al 的/ude1 群体/n 优胜者/n 。/wj
我/rr 爸/n 身后/f 亦步亦趋/vl 地/ude2 跟/v 着/uzhe 我/rr 姐/n 。/wj 她/rr 穿/v 的/ude
¥������.txt
411 篮子/n 里/f 取出/v 一/m 只/q 小/a 纸袋/n ，/wd 吹/v 开/v ，/wd 轻手轻脚/dl 套/v 在/p 那/rzv 株/q 麦穗/n 上/f ，/wd 袋/q 口/n 折叠/v ， ¥������.txt
412 远处/s 的/ude1 棉花/n 地/n 里/f 扬/vg 了/ule 一/m 扬/vg 。/wj 影影绰绰/al 看见/v 那/rzv 块/q 地/n 里/f 有/vyou 几/m 个/q 蹲/v 着/uz ¥������.txt

413 v 只/d 盛/v 放/v 纸袋/n 卡片/n 的/ude1 篮子/n 。/wj 跟/p 姐姐/n 朝夕相处/vl 这么/rz 多/m 年/qt ，/wd 我/rr 是/vshi 头/m 一/m 回/qv 心 ¥�������.txt
414 处/vl 这么/rz 多/m 年/qt ，/wd 我/rr 是/vshi 头/m 一/m 回/qv 心甘情愿/vl 为/p 她/rr 服务/v 。/wj 那/rzv 一刻/mq ，/wd 初夏/t 的/ud ¥�������.txt
415 成/v 了/ule 小孩子/n 涂抹/v 出/vf 来/vf 的/ude1 蜡笔画/n ，/wd 五颜六色/bl ，/wd 绚丽/a 到/v 让/v 人/n 眼花/vi 。/wj 田野/n 上/f 倏忽/d ¥�������.txt
416 我/rr 想起/v 一/m 年/qt 前/f 还/d 在/p 我们/rr 头/m 顶/q 上/f 狂轰滥炸/vl 的/ude1 小/a 日本/nsf 的/ude1 飞机/njtgj 。/wj
世界/n 若 ¥�������.txt
417 /n ，/wd 我/rr 的/ude1 哥哥/n 和/cc 姐姐/n ，/wd 他们/rr 会/v 一心一意/dl 做/v 自己/rr 喜欢/vi 的/ude1 事/n ，/wd 成就/n 自己/rr 想/v ¥�������.txt
418 d 选出/v 又/d 套/v 上/vf 纸袋/n 的/ude1 种/q 穗/n ，/wd 他/rr 呕心沥血/vl 培育/v 了/ule 半/m 年/qt 之/uzhi 多/a 的/ude1 宝贝/n ，/w¥�������.txt
419 以上/f 被/pbei 人/n 砍/v 了/ule 头/n ，/wd 只/d 剩/v 一根根/m 参差不齐/al 竖/v 在/p 田里/s 的/ude1 可怜/a 的/ude1 麦秆/n ，/wd 惊慌失措 ¥�������.txt
420 参差不齐/al 竖/v 在/p 田里/s 的/ude1 可怜/a 的/ude1 麦秆/n ，/wd 惊慌失措/vl 地/ude2 对/p 着/uzhe 他/rr 呼救/vi 哭泣/vi 。/wj
我/rr ¥�������.txt
421 良种/n ，/wd 让/v 我/rr 不/d 要/v 追究/v 。/wj 好/a 嘛/y ！/wt 姑息养奸/nl ，/wd 这/rzv 回/qv 瞄/v 上/vf 我/rr 的/ude1 麦种/n 啦/y ¥�������.txt
422 /wd 一/m 大半/m ！/wt 老梅/n 啊/y ，/wd 一/m 大半/m 啊/y ，/wd 不翼而飞/vl ！/wt "/wyz 梅/nr1 教授/n 衣衫/n 零乱/an ，/wd 胡子/n 拉/ ¥�������.txt
423 声/n 地/ude2 嘀咕/v ：/wm "/wyz 是/vshi 真/a 的/ude1 ？/ww 匪夷所思/vl ，/wd 匪夷所思/vl 。/wj "/wyz
我/rr 爸/n 陀螺/n 一样/uyy ¥�������.txt
424 /v ：/wm "/wyz 是/vshi 真/a 的/ude1 ？/ww 匪夷所思/vl ，/wd 匪夷所思/vl 。/wj "/wyz
我/rr 爸/n 陀螺/n 一样/uyy 在/p 屋里/s 打转/v ¥�������.txt
425 种子/n 没/v 了/ule 这/rzv 是/vshi 事实/n ，/wd 又/d 不/d 能/v 挨家挨户/dl 去/vf 农民/n 家里/s 搜查/v ，/wd 逼/v 着/uzhe 他们/rr 交出/v¥�������.txt
426 v 选/v 出来/vf 的/ude1 麦种/n ，/wd 我/rr 爸/n 不/d 敢/v 再/d 掉以轻心/vl ，/wd 下/vf 命令/n 说/v ，/wd 在/p 麦子/n 收割/vn 之前/f 的/ ¥�������.txt
427 /wd 怕/v 你/rr 哥/n 他们/rr 遭/v 人/n 暗算/v 呢/y 。/wj 我/rr 自告奋勇/vl 说/v ，/wd 要/v 不/d 我/rr 替/p 你/rr 去/vf 看看/v ？/ww ¥�������.txt
428 真的/d 能/v 让/v 国家/n 富强/an ，/wd 能/v 让/v 老百姓/nt 过上/v 丰衣足食/vl 的/ude1 好日子/n ？/ww
一路/mq 上/f 七/m 想/v 八/m 想/v ， ¥�������.txt
429 一/m 声/qv 打开/v 一/m 扇/q 柴门/n ，/wd 背/v 个/q 布/n 口袋/n 蹑手蹑脚/z 走/v 出来/vf ，/wd 一路/mq 小跑/vi 着/uzhe 蹿/vi 进/vf 我/r ¥�������.txt
430 /ule 他/rr 去/vf 见/v 我/rr 爸/n ，/wd 那/rzv 才/d 是/vshi 大快人心/nl 的/ude1 事/n 。/wj
想/v 着/uzhe ，/wd 自己/rr 就/d 嘻/o ¥�������.txt
431 /wj
一会儿/mq ，/wd 就/d 看到/v 远远/d 的/ude1 地方/n 有/vyou 忽明忽暗/vl 的/ude1 光/n ，/wd 似乎/d 还/d 有/vyou 青色/n 的/ude1 一/ ¥�������.txt
432 /ude3 很/d 远/a ，/wd 更/d 远/a 处/n 还/d 有/vyou 一/m 条/q 隐约可见/vl 的/ude1 银白色/n 的/ude1 带子/n ，/wd 那/rzv 是/vshi 银河/ ¥�������.txt
433 /ww 银河/n 两边/f 有/vyou 没/d 有/vyou 翘首/vd 相望/v 的/ude1 牛郎织女/nl 呢/y ？/ww 我/rr 觑/v 起/vf 眼睛/n 努力/ad 往/p 天空/n 中/f ¥�������.txt
434 子/n 读书/vi ，/wd 拿/v 退婚/v 做/v 威胁/vn ，/wd 女孩子/n 父母/n 思来想去/vl ，/wd 觉得/v 婚姻/n 要/v 紧/a ，/wd 期末/t 过来/vf 把/pba 她 ¥�������.txt
435 v 回家/vi 的/ude1 ，/wd 女孩子/n 对/p 着/uzhe 两/m 个/q 下人/n 拳打脚踢/vl ，/wd 还是/c 没/d 用/v 。/wj
一下子/mq 走/v 掉/v 四/m 个/q ¥�������.txt
436 了/ule 暑假/t ，/wd 在家/vi 还是/c 不/d 自由/a ，/wd 所以/c 要/v 争分夺秒/nl 享受/v 两/m 人/n 世界/n 。/wj
我们/rr 仰面/d 朝天/ns 地/ude ¥�������.txt
437 /n 地/ude2 躺/v 在/p 范玛丽/nrf 的/ude1 大/a 床/n 上/f ，/wd 惊慌失措/vl 地/ude2 叫/vi 起来/vf :/wm “/wyz 哎呀/e 侬/ng 格/n 两/m ¥�������.txt
438 /ng 姆/x 妈/n 看见/v …/ws …/ws ”/wyy
范/nr1 舒/vg 文/ng 嗲声嗲气/dl 学/v 她/rr 的/ude1 腔调/n ：/wm “/wyz 阿拉姆/nrf 妈/n 不/
¥�������.txt
439 可是/c 书名/n 就/d 把/pba 她/rr 难/ad 住/vi 了/y ，/wd 她/rr 结结巴巴/z 读/v :/wm “/wyz 在/p 所

有/b 的/ude1 .../ws .../ws 之上/f ？ ￥�������.txt
440 本/q 书/n 你/rr 看/v 到/v 哪儿/rys 了/y ?/ww ”/wyy
我/rr 不好意思/vl 回答/v 说/v 一/m 页/q 没/d 看/v 过/uguo ，/wd 含含糊糊/z 答/ ￥�������.txt
441 我/rr 不好意思/vl 回答/v 说/v 一/m 页/q 没/d 看/v 过/uguo ，/wd 含含糊糊/z 答/v :/wm “/wyz 我/rr 姐/n 也/d 在/p 看/v 。/wj ”/wyy ￥�������.txt
442 写/v 的/ude1 是/vshi 世界/n 上/f 最/d 最/d 美好/a 、/wn 最/d 罗曼蒂克/a 、/wn 最/d 让/v 人/n 向往/vn 憧憬/vn 的/ude1 事情/n 啊/y !/ ￥�������.txt
443 脑瓜/n 。/wj ”/wyy
过/vf 了/ule 一会儿/mq ，/wd 她/rr 又/d 悲天悯人/vl 地/ude2 替/p 我/rr 发愁/v :/wm “/wyz 什么/ry 时候/n 你/r ￥�������.txt
444 ？/ww 手术刀/n 嘛/y ！/wt 你们/rr 中国/ns 的/ude1 观音/n 菩萨/n 救苦救难/vl ，/wd 医生/n 就/d 是/vshi 观音/n 菩萨/n 嘛/y 。/wj "/wyz ￥�������.txt
445 就/d 弄/v 到/v 了/ule 大学/n 四/m 年/qt 的/ude1 课程表/n ，/wd 从头到尾/dl 研究/v 了/ule 一/m 遍/qv ，/wd 他/rr 发现/v 一个/mq 很/d 重 ￥�������.txt
446 题/n ：/wm 新闻学/n 当中/f 摄影/vi 是/vshi 重头戏/n ，/wd 一个/mq 新闻记者/n 如果/c 不/d 会/v 摄影/vi ，/wd 他/rr 就/d 是/vshi 个/q "/w ￥�������.txt
447 wd 显影液/n ，/wd 定影液/n ./wj ./wj ./wj ./wj ./wj ./wj 林林总总/vl ，/wd 是/vshi 一/m 笔/q 吓人/a 的/ude1 费用/n 。/wj
我/r ￥�������.txt
448 摊/ng ，/wd 回家/vi 后/f 拿/v 纸/n 笔算/v 了/ule 算账/vi ，/wd 如释重负/vl 地/ude2 告诉/v 我们/rr 说/v ，/wd 价钱/n 不/d 贵/a ，/wd 吓 ￥�������.txt
449 逃难/vi 到/v 成都/ns ，/wd 又/d 苦/ad 熬/v 这些/rz 年/qt ，/wd 坐吃山空/vl ，/wd 已经/d 靠/v 典当/v 为生/vi ，/wd 好/a 东西/n 也/d 是/v ￥�������.txt
450 v 一定/b 的/ude1 表演/vn ，/wd 竟然/d 把/pba 主编/v 感动/v 到/v 热泪盈眶/al ，/wd 当即/d 拍板/vi 要/v 尽快/d 地/ude2 连载/v 这/rzv 本/q￥�������.txt
451 要/v 尽快/d 地/ude2 连载/v 这/rzv 本/q 小说/n 。/wj 主编/n 还/d 信心百倍/vl 地/ude2 说/v ，/wd 等/udeng 刊物/n 连载/v 完了/vi ，/wd 马 ￥�������.txt
452 /vn 来说/uls ，/wd 简直/d 就/d 是/vshi 及时雨/n ，/wd 是/vshi 天时地利/nl 人和/n ！/wt "/wyz 主编/n 紧紧/d 握住/v 我/rr 哥/n 的/ude1 ￥�������.txt
453 跟/v 着/uzhe 兴奋/a ，/wd 一/m 把/pba 抱/v 起/vf 小弟/n ，/wd 大喊大叫/vl 地/ude2 转/v 了/ule 一/m 圈/qv 又/d 一/m 圈/qv ，/wd 吓/ ￥�������.txt
454 /d 骂/v 她/rr "/wyz 疯子/n "/wyy 。/wj 沈天路/ns 则/d 已经/d 默不作声/vl 地/ude2 忙/v 前/f 忙/v 后/f ，/wd 为/p 我/rr 哥/n 腾出/v ￥�������.txt
455 n 去/vf 送/v 稿/ng ，/wd 沈天路/ns 跟/p 我/rr 守/v 在/p 家里/s 心急火燎/al 等/udeng 结果/n 。/wj 等待/v 中/f 我们/rr 闲聊/v ，/wd 沈天路 ￥�������.txt
456 数/v 了/ule 十/m 来/m 部/q 。/wj
我/rr 怀疑/v 他/rr 是/vshi 道听途说/bl ，/wd 因为/c 我/rr 从来/d 没/d 见/v 他/rr 读/v 过/uguo 这些 ￥�������.txt
457 e 一个/mq 秃头/n 老师/n 给/p 沈天路/ns 他们/rr 上课/vi ，/wd 还/d 津津有味/al 讲述/v 《/wkz 茶花女/n 》/wky 的/ude1 情景/n ，/wd 更/d 是/ ￥�������.txt
458 ule 嘴/n 。/wj
"/wyz 脸红/vi 了/y 吧/y ？/ww "/wyz 我/rr 不依不饶/vl ，/wd "/wyy 承认/v 你/rr 喜欢/vi 我/rr 姐/n 了/y 吧/y ？/ ￥�������.txt
459 长/a 大/a 。/wj
我/rr 哥/n 我/rr 姐/n 回来/v 的/ude1 时候/n 喜气洋洋/vl ，/wd 我/rr 在/p 楼上/s 都/d 听到/v 姐姐/n 跟/v 邢/nr1 姆/x ￥�������.txt
460 d 不用/d 问/v 啦/y ，/wd 肯定/d 是/vshi 一/m 炮/n 打响/v ，/wd 旗开得胜/vl 。/wj
奇怪/v 的/ude1 是/vshi 他们/rr 还/d 带/v 回来/v 一个 ￥�������.txt
461 得胜/vl 。/wj
奇怪/v 的/ude1 是/vshi 他们/rr 还/d 带/v 回来/v 一个陌生/n 人/n ，/wd 这/rzv 人/n 个头/n 不/d 高/a ，/wd 细/a 胳膊/n 细 ￥�������.txt
462 长/a 得/ude3 紧凑/a ，/wd 说/v 起/vf 话/n 来/f 却/d 是/vshi 手舞足蹈/al ，/wd 表情/n 也/d 透/v 着/uzhe 古/a 灵/a 精怪/n 。/wj 我/r ￥�������.txt
463 wyz 可/v 我们/rr 还/d 没有/v 胜利/vi 呢/y 。/wj "/wyz
他/rr 一本正经/al ：/wm "/wyz 总/d 有/vyou 这/rzv 一/m 天/qt ，/wd 胜利/v ￥�������.txt
464 举/ng ，/wd 做出/v 一个/mq 飞翔/v 的/ude1 姿态/n ，/wd "/wyy 人民战争/n 是/vshi 大海/n ，/wd 乌云/n 是/vshi 可恶/a 的/ude1 小/a 日本 ￥�������.txt
465 丈/q 的/ude1 人物/n 。/wj 我/rr 看见/v 沈天路/ns 听/v 得/ude3 目瞪口呆/al ，/wd 手里/s 的/ude1 冰棒/n 一时间/t 忘/v 了/ule 吮吸/v ，/w ￥�������.txt
466 /m 多/m 岁/qt 了/y ，/wd 吟/v 哦/e 古诗词/n 也/d 会/v 这样/rzv 摇头晃脑/vl ，/wd 陶醉/vi 不已/vi 。/wj 不过/c 老师/n 是/vshi 自己/rr 让 ￥�������.txt

467　看/v 《/wkz 高于/v 一切/rz 》/wky 的/ude1 原文/n 。/wj 他/rr 热情洋溢/nl 地/ude2 说/v ，/wd 这/rzv 本/q 书/n 真/d 是/vshi 写/v 得　¥�������.txt
468　根/q 冰棒/n 仔细/ad 吮/v 完/vi 。/wj 他/rr 的/ude1 话语/n 像/v 山洪暴发/vl 一样/uyy 滔滔不绝/vl ，/wd 有/vyou 一/m 种/q 泥沙俱下/vl 裹挟/　¥�������.txt
469　吮/v 完/vi 。/wj 他/rr 的/ude1 话语/n 像/v 山洪暴发/vl 一样/uyy 滔滔不绝/vl ，/wd 有/vyou 一/m 种/q 泥沙俱下/vl 裹挟/v 一切/rz 的/ude1　¥�������.txt
470　/n 像/v 山洪暴发/vl 一样/uyy 滔滔不绝/vl ，/wd 有/vyou 一/m 种/q 泥沙俱下/vl 裹挟/v 一切/rz 的/ude1 气势/n 。/wj
完/vi 了/ule 他/rr 提　¥�������.txt
471　/v 一切/rz 》/wky 这个/rz 书名/n 有点/d 概念化/v ，/wd 不/d 够/v 罗曼蒂克/a ，/wd 换/v 一个/mq 怎么样/ryv ？/ww 《/wkz 生死/n 之/uzhi 恋　¥�������.txt
472　要/v 他/rr 留下/v 吃饭/vi 。/wj 娘/n 为了/p 招待/v 贵客/n ，/wd 手忙脚乱/al 地/ude2 炒/v 了/ule 一/m 盘/qv 腊肉/nms ，/wd 还/d 蒸/v　¥�������.txt
473　v 不/d 怎么/ryv 在/p 意/ng ，/wd 他/rr 在/p 饭桌/n 上/f 一直/d 滔滔不绝/vl 地/ude2 跟/v 我/rr 爸/n 讲话/vi ，/wd 分析/v 各种/rz 战场/n　¥�������.txt
474　nrf 的/ude1 失败/vn ，/wd 日本/nsf 支持/v 缅甸/ns 独立/v ，/wd 共产国际/nl 解散/v 。/wj 他/rr 还/d 提到/v 了/ule 延安/ns ，/wd 提到/v　¥�������.txt
475　rr 更/d 深刻/a 的/ude1 了/y 。/wj "/wyz 他/rr 说/v 得/ude3 斩钉截铁/al 。/wj
他/rr 走/v 了/ule 之后/f ，/wd 我/rr 爸/n 背/v 着/　¥�������.txt
476　便/d 已经/d 脱销/vi 。/wj 加印/v 了/ule 一/m 次/qv ，/wd 依然/d 供不应求/vl 。/wj 第二/m 期/qv 连载/v 时/ng ，/wd 主编/v 一/m 咬牙/vi ，　¥�������.txt
477　wd 他/rr 专门/d 为/v 她/rr 拍/v 了/ule 几/m 张/q 特写/n ，/wd 信誓旦旦/vl 说/v ：/wm "/wyz 肯定/v 比/p 明星/n 照/v 还/d 高级/a 。/wj　¥�������.txt
478　/ng 儿/ng 。/wj 我/rr 哥/n 觉得/v 这样/rzv 用/v 起来/vf 可以/v 细水长流/vl ，/wd 万一/d 拍/v 坏/a 了/ule 或者/c 冲洗/v 坏/a 了/y ，/wd　¥�������.txt
479　是/vshi 一个/mq 很/d 有/vyou 钱/n 的/ude1 庄园主/n ，/wd 在/p 马来西亚/nsf 拥有/v 大片/n 的/ude1 橡胶/n 园/ng ，/wd 五十/m 岁/qt 之后/　¥�������.txt
480　qt 之后/f 产业/n 交/ng 给/p 儿子/n 们/k 打/v 理/n ，/wd 自己/rr 告老还乡/vl 。/wj 因为/p 祖上/n 曾/d 是/vshi 举人/n ，/wd 自己/rr 又/d　¥�������.txt
481　/n ，/wd 没/v 事/n 就/d 过去/vf 看/v 书/n ，/wd 喝茶/vi ，/wd 谈天说地/vl ，/wd 一/d 坐/v 大/a 半/m 天/qt 时间/n 。/wj 书店/n 在/p 任　¥�������.txt
482　半/m 天/qt 时间/n 。/wj 书店/n 在/p 任何/rz 时候/n 都/d 是/vshi 热热闹闹/z 。/wj
我/rr 头/m 一/m 回/qv 跟/p 姐姐/n 去/vf "/wyz 天地/　¥�������.txt
483　。/wj 既然/c 是/vshi 哥哥/n 给/u 的/ude1 钱/n ，/wd 沈天路/ns 不好意思/vl ，/wd 我们/rr 两/m 个/q 好/a 意思/n 。/wj 我/rr 想/v 用/p　¥�������.txt
484　v 了/ule 一/m 扇/q 又/d 一/m 扇/q 的/ude1 窗口/s 。/wj 我/rr 模模糊糊/z 意识/n 到/v ，/wd 我/rr 生活/vi 的/ude1 这个/rz 国家/n 和/cc　¥�������.txt
485　/rr 生活/vi 的/ude1 这个/rz 国家/n 和/cc 社会/n 其实/d 是/vshi 动荡不安/vl 的/ude1 ，/wd 有/vyou 一/m 种/q 崭新/b 的/ude1 东西/n 正在　¥�������.txt
486　ude1 潮/n ，/wd 总之/c 它/rr 会/v 让/v 我们/rr 的/ude1 世界/n 天翻地覆/al 。/wj
鲁迅/nr 先生/n 的/ude1 书/n 我/rr 不久/m 之前/f 也/d　¥�������.txt
487　/wj 推开/v 那/rzv 扇/q 黑/a 漆/n 门/n ，/wd 才/d 发现/v 里面/f 别有洞天/vl 。/wj 大白天/n 的/ude1 ，/wd 店堂/n 里/f 居然/d 灯火/n 通明/z　¥�������.txt
488　半/m 倚/v 半/m 靠/v 的/ude1 ，/wd 两两/m 促膝/v 的/ude1 ，/wd 交头接耳/vl 的/ude1 ，/wd 全都/d 是/vshi 十几/m 二十/m 岁/qt 的/ude1　¥�������.txt
489　vshi 凑热闹/vi ，/wd 居然/d 盘腿/vd 坐/v 在/p 角落/n 里/f ，/wd 全神贯注/dl 翻/v 着/uzhe 绷/v 绷/v 绳/ng 。/wj
我/rr 姐/n 小声/vd 惊　¥�������.txt
490　先生/n ！/wt "/wyz
想/v 也/d 没有/d 想/v ，/wd 我们/rr 就/d 急急忙忙/z 赶/v 过去/vf 。/wj 进/vf 门/n 发现/v 人/n 很/d 多/a ，/wd 横　¥�������.txt
491　wd 站/vi 在/p 北/f 首/m 的/ude1 一/m 张/q 靠背椅/n 上/f ，/wd 汗流浃背/al 地/ude2 给/v 大家/rr 介绍/v 他/rr 手里/s 拿/v 着/uzhe 的/u　¥�������.txt
492　f 都/d 跟着/v 毛刺/n 刺/v 的/ude1 难受/a 。/wj
李胜利/nr 正在/d 绘声绘色/al 地/ude2 朗读/v 书/n 中/f 的/ude1 一/m 段/q 话/n ：/wm "/
¥�������.txt
493　/rr 小说/n 里/f 的/ude1 词句/n 也/d 不/d 一样/uyy 。/wj 我/rr 似懂非懂/vl ，/wd 还/d 特别/d 盼/v 着/uzhe 能/v 懂/v ，/wd 心里/s 揣/v　¥�������.txt
494　/f 窜/vi 了/ule 过来/vf ，/wd 当着/p 所有/b 人/n 的/ude1 面/n 亲亲热热/z 地/ude2 喊/v ：/wm "/wyz 两/m 位/q 密/a 斯/dg 黄/a ，/wd¥�������.txt
495　rr 再/d 读/v 一/m 段/q 给/p 你们/rr 听/v 。/wj "/wyz

他/rr 急急忙忙/z 地/ude2 反/vi 身/ng 回去/v ，/wd 嗖/o 的/ude1 —/m 下/f 再 ¥�������.txt
496 wd 不致/d 因/p 虚度/v 年华/n 而/cc 悔恨/v ，/wd 也/d 不致/d 因/p 碌碌无为/vl 而/cc 羞愧/vn 。/wj 在/p 他/rr 临死/vi 的/ude1 时候/n ，/w¥�������.txt
497 d 把/pba 小/a 日本/nsf 赶/v 出/vf 中国/ns ，/wd 建立/v 我们/rr 梦寐以求/bl 的/ude1 民主/a 社会/n ！/wt 同学/n 们/k ，/wd 在/p 二十/m 世 ¥�������.txt
498 走/v 出/vf "/wyz 天地/n "/wyy 书店/n 时/ng ，/wd 我/rr 姐/n 激动不已/vl ，/wd 忍不住/v 问/v 我/rr ：/wm "/wyz 你/rr 说/v ，/wd 李 ¥�������.txt
499 d 她/rr 完全/ad 不/d 能/v 明白/an ，/wd 保/v 尔/y 既然/c 那么/c 爱冬妮娅/nrf ，/wd 冬/tg 妮/ng 娅/x 又/d 是/vshi 那么/rz 美丽/a 善良/a¥�������.txt
500 /pba 秋/tg 气/n 猛/d 吹/v ，/wd 不/d 露脸/vi 便/d 将/p 落叶/n 一扫而空/vl ，/wd 犹如/v 法师/n 赶走/v 了/ule 群/q 鬼/n ./wj ./wj ./ ¥�������.txt
501 v 再/d 去/vf 。/wj 我/rr 哥/n 说/v ，/wd 他/rr 比较/d 喜欢/vi 心平气和/vl 地/ude2 讨论/v 问题/n ，/wd 理智/n 地/ude2 分析/v 形势/n 和/ ¥�������.txt
502 vn 时间/n ，/wd 暑假/t 过/vf 后/f 他/rr 读/v 高三/n ，/wd 要/v 全力以赴/vl 准备/v 考/v 大学/n 。/wj 他/rr 现在/t 想/v 考/v 金陵/ns 大学/ ¥�������.txt
503 /rr 可以/v 自由/ad 选择/v 人生/n 道路/n 。/wj "/wyz 我/rr 姐/n 真心诚意/dl 地/ude2 对/p 他/rr 说/v 。/wj
"/wyz 不/d ，/wd 我/rr ¥�������.txt
504 之下/f ，/wd 我/rr 姐/n 只/d 能/v 抓住/v 我/rr 不/d 放/v ，/wd 三天两头/dl 拽/v 着/uzhe 我/rr 去/vf 书店/n 。/wj 整个/b 暑假/t 的/ude ¥�������.txt
505 rr 完全/ad 听/v 不/d 懂/v 。/wj 我/rr 姐/n 也/d 有/vyou 点/n 坐立不安/al ，/wd 不/d 知道/v 是/vshi 不/d 是/vshi 李胜利/nr 不/d 在场/¥�������.txt
506 /p 窗口/s 望/v 向/p 对面/f 的/ude1 屋顶/n ，/wd 仿佛/d 有/vyou 千军万马/nl 横扫/v 而/cc 过/vf ，/wd 扬/vg 起/vf 的/ude1 水烟/n —/m ¥�������.txt
507 /ryv 知道/v 我/rr 在/p 书店/n 啊/y ？/ww "/wyz
程渝/nr 生/v 老老实实/z 答/v ：/wm "/wyz 听/v 同学/n 说/v 的/ude1 ，/wd 他们/rr 都
¥�������.txt
508 。/wj 雨点/n 落/v 在/p 程渝生/nr 的/ude1 油/n 布伞/n 面/q ，/wd 噼噼啪啪/o 地/ude2 响/v ，/wd 我/rr 尖/a 起/vf 耳朵/n 也/d 听/v 不/d ¥�������.txt
509 什么/ry 。/wj 最后/f 我/rr 姐/n 伸/v 进/vf 一个/mq 头/n ，/wd 怒气冲冲/vl 招呼/v 我/rr ：/wm "/wyz 橙子/n ，/wd 还/d 不/d 走/v ？/w ¥�������.txt
510 /a ，/wd 潺潺/o 不/d 断/v 。/wj 蛙/ng 声/ng 和/cc 虫/n 鸣/vg 此起彼伏/vl ，/wd 引来/v —/m 只/q 浑身/n 湿淋淋/z 的/ude1 狗/n ，/wd 怒 ¥�������.txt
511 伏/vl ，/wd 引来/v —/m 只/q 浑身/n 湿淋淋/z 的/ude1 狗/n ，/wd 怒气冲冲/vl 地/ude2 奔/v 上/vf 田埂/n ，/wd 对/p 着/uzhe 看/v 不/d 见 ¥�������.txt
512 秋天/t ，/wd 华西/ns 坝上/s 的/ude1 学生/n 运动/vn 会/v 是/vshi 万人空巷/vl 的/ude1 盛事/n 。/wj 不光/c 五/m 所/q 大学/n 的/ude1 学生/n ¥�������.txt
513 n 。/wj
仲/nr1 老师/n 特别/d 要/v 强/ng ，/wd 工作/vn 起来/vf 一丝不苟/vl ，/wd 每年/r —/m 次/qv 的/ude1 秋季/t 运动会/n 便/d 是/vsh ¥�������.txt
514 /ude1 口令/n ，/wd 光/d 这/rzv 阵势/n 和/cc 形式/n 就/d 能够/v 先声夺人/vl 。/wj
我们/rr 列队/vi 在/p 操场/n ，/wd 听/v 仲/nr1 老师/n ¥�������.txt
515 成熟/a 了/y ，/wd 腰/n 是/vshi 腰/n 腿/n 是/vshi 腿/n ，/wd 亭亭玉立/vl 。/wj 我/rr 往/p 她/rr 面前/f —/m 站/q ，/wd 细溜溜/z 的/u ¥�������.txt
516 i 后/f 退/v 还/d 是/vshi 左/f 转/v 右/f 转/v ，/wd 总/d 能/v 准确无误/al 地/ude2 回到/v 原位/n 。/wj 我/rr 的/ude1 搭档/n 不行/a ，/ ¥�������.txt
517 de3 别提/v 有/vyou 多/m 来/vf 劲/n 。/wj 有/vyou —/m 天/qt 乐极生悲/vl ，/wd 头/n 上/f 的/ude1 篮筐/n 刮/v 到/v 锅台/n 上/f 的/ud ¥�������.txt
518 a 素/ag ，/wd 做/v 了/ule —/m 回/qv 和/cc 事/n 佬/ng 。/wj 那天中午/t 真/a 的/ude1 就/d 没有/v 碗/n 盛饭/v 了/y ，/wd 我/rr 娘/n ¥�������.txt
519 提/v 水/n ，/wd —/m 手/n —/m 只/q 水桶/n ，/wd 提/v 起来/vf 轻轻松松/z 玩儿/v 似的/uyy ，/wd 掷/v 个/q 铅球/n 肯定/v 不在话下/vl 。/wj ¥�������.txt
520 v 起来/vf 轻轻松松/z 玩儿/v 似的/uyy ，/wd 掷/v 个/q 铅球/n 肯定/v 不在话下/vl 。/wj 我/rr 哥/n 提醒/v 他/rr 说/v ，/wd 应该/v 不/d 是/vs ¥�������.txt
521 姐/n 在/p 学校/n 一直/d 参加/v 体操/n 训练/vn ，/wd 运动会/n 上/f 顺理成章/vl 地/ude2 报/v 了/ule 两/m 个/q 体操/n 项目/n ：/wm 平衡木/n ¥�������.txt
522 wj 她/rr 一直/d 认为/v 自己/rr 这/rzv 两/m 个/q 项目/n 是/vshi 十拿九稳/al ，/wd 却/d 不料/d 第—/m 天/qt 比赛/vn 平衡木/n ，/wd 风/n — ¥�������.txt
523 rr 那个/rz 体育/n 老师/n 戴/v —/m 副/q 黑/a 框/ng 眼镜/n ，/wd 文质彬彬/vl 的/ude1 样子/n ，/wd 在/p 国外/s 得/v 了/ule 体育/n 硕士/n ¥�������.txt
524 到位/vi ，/wd 将/p 整个/b 场上/s 的/ude1 节奏/n 控制/v 得/ude3 恰到好处/al 。/wj —/m 场/qv

球/n 赛/vn ，/wd 只要/c 我/rr 哥/n 上场/v ￥�������.txt
525 /n 穿行/vi 在/p 水底/s 一样/uyy ，/wd 左冲/nr 右/f 突/vg ，/wd 见缝插针/vl ，/wd 把/pba 比赛/vn
踢/v 得/ude3 行云流水/nl 一般/uyy 。/w ￥�������.txt
526 右/f 突/vg ，/wd 见缝插针/vl ，/wd 把/pba 比赛/vn 踢/v 得/ude3 行云流水/nl 一般/uyy 。/wj 进
燕/nr2 大/a 足球队/n 没有/v 多久/ryt ，/wd ￥�������.txt
527 f 最/d 瞩目/vi 的/ude1 一/m 颗/q 新星/n ，/wd 女/b 学生/n 们/k 趋之若鹜/vl ，/wd 看到/v 比赛/vn
通告/n 就/d 会/v 奔走相告/vl ，/wd 蜂拥/v ￥�������.txt
528 女/b 学生/n 们/k 趋之若鹜/vl ，/wd 看到/v 比赛/vn 通告/n 就/d 会/v 奔走相告/vl ，/wd 蜂拥/vi
去/vf 看/v ，/wd 还/d 争/v 着/uzhe 端/v 茶/ ￥�������.txt
529 /v 有/vyou 希望/n ，/wd 只能/v 把/pba 心态/n 放/v 平和/a ，/wd 听天由命/vl 就/d 好/a 。/wj
开幕式/n 那天/r ，/wd 我们/rr 全家/n 齐齐/nr ￥�������.txt
530 ule 新闻稿/n ，/wd 对/p 着/uzhe 话筒/n 念/v ，/wd 后来/t 干脆/d 赤膊上阵/vl ，/wd 一/m 群/q
人/n 涌/v 上/f 播音/vi 台/q ，/wd 在/p 话筒 ￥�������.txt
531 人/n ，/wd 因为/c 他/rr 的/ude1 相机/n 终于/d 有/vyou 了/ule 堂堂正正/vl 的/ude1 用武之地/nl
。/wj 除去/v 自己/rr 的/ude1 比赛/vn 时间 ￥�������.txt
532 他/rr 的/ude1 相机/n 终于/d 有/vyou 了/ule 堂堂正正/vl 的/ude1 用武之地/nl 。/wj 除去/v 自己/rr
的/ude1 比赛/vn 时间/n 之外/f ，/wd 他/ ￥�������.txt
533 燕京/nz 大学/n 的/ude1 队/n 伍一/m 字/n 长/a 蛇/n 阵/ng ，/wd 浩浩荡荡/z 直奔/v 运动场/n 而/cc
来/vf 。/wj 自行车/n 部队/n 作/v 前导/v ， ￥�������.txt
534 /wd 步伐/n 整齐/a ，/wd 啦啦队/n 各/rz 部门/n 全体/n 出动/v ，/wd 大队人马/nl 声威/n 极/d 盛/v
。/wj 待/p 战报/n 传出/v ，/wd 必将/d 显赫/a ￥�������.txt
535 战报/n 传出/v ，/wd 必将/d 显赫/a 。/wj 燕/ng 大/a 健儿/n ，/wd 名列前茅/vl ，/wd 各项/r 冠
军/n ，/wd 非/b 我/rr 莫/d 属/v ，/wd 锦标/n ￥�������.txt
536 wyz
新闻稿/n 由/p 一/m 位/q 燕/ng 大/a 女生/n 在/p 话筒/n 里/f 字正腔圆/al 地/ude2 播出/v 之后/f ，/wd
场/qv 中/f 一/m 片/q 嘘声/n ，/ ￥�������.txt
537 /wd 燕大/nr2 ，/wd 我/rr 最/d 强大/a ！/wt "/wyz 我/rr 爸/n 针锋相对/al ：/wm "/wyy 金/ng 大/a
努力/an ，/wd 永/d 争/v 第一/m ！/ ￥�������.txt
538 shi 由/p 欢乐/an 开始/v ，/wd 不/d 知道/v 中国/ns 古谚/n "/wyz 乐极生悲/vl "/wyy 是/vshi 不/d
是/vshi 说/v 的/ude1 这个/rz 意思/n ￥�������.txt
539 wyy 是/vshi 不/d 是/vshi 说/v 的/ude1 这个/rz 意思/n 。/wj
一九四三/m 的/ude1 秋天/t ，/wd 给/p 苦难/n 中/f 的/ude1 中国/ns 带来/v ￥�������.txt
540 /v ，/wd 盟军/n 已经/d 攻/v 入/v 法西斯/nz 占领/v 之下/f 的/ude1 西西里岛/nsf 。/wj 我/rr 哥/n
拿/v 出/vf 世界/n 地图/n 查找/v 给/p 我们/ ￥�������.txt
541 当中/f 绝对/d 具有/v 权威性/n ，/wd 我们/rr 当即/d 佩服/v 得/ude3 一塌糊涂/al 。/wj
结果/n 是/vshi ，/wd 不到/v 两/m 个/q 月/n 的/ude1 ￥�������.txt
542 vi ；/wf 五/m 天/qt 之后/f ，/wd 意大利/nsf 宣布/v 投降/v ；/wf 十月中旬/t ，/wd 意大利/nsf 反
过来/d 对/p 德国/nsf 宣战/vi 。/wj
报纸/n ￥�������.txt
543 /wd 意大利/nsf 反过来/d 对/p 德国/nsf 宣战/vi 。/wj
报纸/n 上/f 连篇累牍/dl 地/ude2 讲述/v 一些/mq 关于/p 意大利/nsf 军队/n 的/ude1 笑话/
￥�������.txt
544 ，/wd 金发/n 碧眼/n 的/ude1 小伙子/n 们/k 一/d 进/vf 校园/n 就/d 眉飞色舞/vl 地/ude2 趴/v 在/p
车厢/n 两边/f ，/wd 不/d 停/vi 地/ude2 ￥�������.txt
545 十一/m 名/q 上场/vi 队员/n 却/d 是/vshi 满/a 脸/n 紧张/a ，/wd 严阵以待/vl ，/wd 连/ulian 赛
前/t 准备/v 活动/vn 都/d 做/v 得/ude3 一丝 ￥�������.txt
546 以待/vl ，/wd 连/ulian 赛前/t 准备/v 活动/vn 都/d 做/v 得/ude3 一丝不苟/vl 。/wj
华西/ns 坝/n 的/ude1 足球场/n 边/k 里/f 外/f 三/m 层 ￥�������.txt
547 其次/c ，/wd 爬/v 到/v 场/qv 边/n 的/ude1 一个/mq 篮球架/n 上/f 居高临下/vl 看/v 比赛/vn 。/wj
范舒文/nr 爬/v 上/f 去/vf 之后/f 累/v 得/ ￥�������.txt
548 n 在/p 即将/d 终场/n 时/ng 光荣/a 负伤/vi ，/wd 被/pbei 一个/mq 人高马大/al 的/ude1 英国/ns 队
员/n 一/m 脚/q 绊倒/v ，/wd 连续/ad 翻滚/v ￥�������.txt
549 连续/ad 翻滚/vi 了/ule 几/m 圈/qv ，/wd 坐/v 在/p 地上/s ，/wd 龇牙咧嘴/vl 地/ude2 抱/v
着/uzhe 一/m 只/q 脚/n 。/wj 几/m 个/q 预备/ ￥�������.txt
550 wj "/wyz
他们/rr 从/p 成都/ns 坐船/vi ，/wd 沿/p 岷/ng 江/n 顺流而下/vl 至/p 宜宾/ns ，/wd 溯/vg 金沙江/ns 抵/v
屏山/ns ，/wd 再/d ￥�������.txt
551 v 了/ule 两/m 个/q 当地/s 彝族/nz 人/n 当/p 向导/n ，/wd 进入/v 大小凉山/n 交界处/n 的/ude1 原
始/a 森林/n ，/wd 一路/mq 险象环生/vl ，/wd ￥�������.txt
552 /wd 进入/v 大小凉山/n 交界处/n 的/ude1 原始/a 森林/n ，/wd 一路/mq 险象环生/vl ，/wd 终于/d
到达/v 彝/nz 区/n 。/wj

陶/nr1 伯伯/n 在/p 彝 ￥�������.txt
553 1 彝/nz 人/n 朋友/n 拿/v 青/a 藤/n 扎/v 一/m 副/b 担架/n ，/wd 千辛万苦/nl 地/ude2 把/pba 他/rr 抬/v 回/v 成都/ns 。/wj
请/v 了/ul ￥�������.txt
554 de1 苦味/n 。/wj
几/m 天/qt 工夫/n ，/wd 陶/nr1 伯伯/n 已经/d 奄奄一息/vl ，/wd 原来/d 高/a 大/a 快乐/a 妙语连珠/al 的/ude1 一个/mq 人/ ￥�������.txt
555 wd 陶/nr1 伯伯/n 已经/d 奄奄一息/vl ，/wd 原来/d 高/a 大/a 快乐/a 妙语连珠/al 的/ude1 一个/mq 人/n ，/wd 消瘦/a 到/v 躺/v 在/p 床/n 上/f ￥�������.txt
556 z 值/n 不/d 值/v 的/ude1 话/n ！/wt 这/rzv 世界/n 上/f ，/wd 从古到今/dl ，/wd 为/p 科学/n 为/p 真理/n 牺牲/v 性命/n 的/ude1 不止/v 一 ￥�������.txt
557 ，/wd 说/v 老陶/nr 这个/rz 病/n ，/wd 只有/c 弄/v 到/v "/wyz 金鸡纳霜/n "/wyy 才/d 有/vyou 希望/n 。/wj 我/rr 爸/n 问/v "/wyz 金 ￥�������.txt
558 霜/n "/wyy 才/d 有/vyou 希望/n 。/wj 我/rr 爸/n 问/v "/wyz 金鸡纳霜/n "/wyy 是/vshi 什么/ry 东西/n ，/wd 范/nr1 伯伯/n 说/v 是/v ￥�������.txt
559 疟疾/n 的/ude1 特效药/n ，/wd 也/d 叫/vi 奎宁/n ，/wd 产/v 在/p 热带雨林/n 的/ude1 金鸡/n 纳/v 树上/s ，/wd 中国/ns 是/vshi 没有/v 这/r ￥�������.txt
560 vf 水/n ，/wd 但是/c 也/d 慢/a 不/d 过/uguo 三/m 天/qt 。/wj 无论如何/dl ，/wd 五/m 天/qt 之内/f 总/d 能/v 取回/v 药品/n 。/wj
我/r ￥�������.txt
561 期/n ，/wd 七/m 天/qt 。/wj 他/rr 坐/v 上/vf 船/njtgj ，/wd 顺流而下/vl ，/wd 到/v 宜宾/ns 找到/v 救护/vn 分队/n ，/wd 出/vf 示范/vn ￥�������.txt
562 久/m 之后/f 被/pbei 另/rz 一/m 艘/q 船/njtgj 救/v 下/f ，/wd 几经周折/vl 才/d 回/v 到/v 成都/ns 。/wj 可惜/v ，/wd 他/rr 一路/mq 奔跑 ￥�������.txt
563 /rr 娘/n 却/d 一直/d 对/p 他/rr 翻船/vi 落水/vi 的/ude1 遭遇/n 心有余悸/vl ，/wd 我/rr 不/d 止/v 一/m 次/qv 听/v 她/rr 对/p 我/rr 爸 ￥�������.txt
564 娘/n 对/p 沈天路/ns 更加/d 怜惜/v 了/y ，/wd 我/rr 从/p 他们/rr 进进出出/v 看/v 向/p 沈天路/ns 的/ude1 眼神/n 里/f 能够/v 察觉/v 出来/vf ￥�������.txt
565 /wj 当时/t 他/rr 运送/v 的/ude1 是/vshi 宝贵/a 无比/z 的/ude1 战略物资/nl --/wp 作战/vn 飞机/njtgj 所/usuo 需要/v 的/ude1 特殊/a 油 ￥�������.txt
566 n 军/n 军官/n 陪同/vn 马克/n 在/p 飞虎队/nz 的/ude1 同事/n ，/wd 千里迢迢/dl 把/pba 一/m 包/q 遗物/n 从/p 云南昆明/ns 送/v 到/v 了/ule 成 ￥�������.txt
567 z 的/ude1 同事/n ，/wd 千里迢迢/dl 把/pba 一/m 包/q 遗物/n 从/p 云南昆明/ns 送/v 到/v 了/ule 成都/ns 范/nr1 伯伯/n 家里/s 。/wj 那/rzv ￥�������.txt
568 v 五/m 千/m 米/q 的/ude1 驼峰/n 航线/n ，/wd 是/vshi 怎样/ryv 惊心动魄/vl 的/ude1 死亡/vn 之/uzhi 旅/ng 。/wj
第十九/m 章/q ·/w 抓 ￥�������.txt
569 wd 陶/nr1 伯伯/n 病逝/vi ，/wd 马克/n 牺牲/v ，/wd 榴/ng 园/ng 不知不觉/dl 中/f 笼罩/v 着/uzhe 一/m 层/qv 悲哀/a 的/ude1 阴影/n 。/wj ￥�������.txt
570 1 人/n 都/d 领/v 走/v 了/y 。/wj "/wyz
范/nr1 舒/vg 文/ng 大惊失色/vl 地/ude2 看/v 我/rr ，/wd 脸/n 白/d 成/v 一/m 张/q 纸/n ：
￥�������.txt
571 》/wky 《/wkz 中央/n 日报/n 》/wky ，/wd 也/d 有/vyou 《/wkz 新华日报/ntjs 》/wky ，/wd 包括/v 没有/d 太/d 多/a 人/n 知晓/v 的/ude1 ￥�������.txt
572 哥/n 说/v 《/wkz 国难/n 三日/t 刊/vg 》/wky 应该/v 跟/p 《/wkz 新华日报/ntjs 》/wky 一样/uyy ，/wd 都/d 是/vshi 共产党人/n 办/v 的/ud ￥�������.txt
573 跟/p 《/wkz 新华日报/ntjs 》/wky 一样/uyy ，/wd 都/d 是/vshi 共产党人/n 办/v 的/ude1 报纸/n ，/wd 因为/c 报/n 上/f 最/d 常/d 刊登/v ￥�������.txt
574 的/ude1 巨大/a 的/ude1 废墟/n ，/wd 在/p 废墟/n 之中/f 有/vyou 生气勃勃/vl 的/ude1 力量/n ，/wd 有/vyou 破土/vi 而/cc 出/vf 茁壮/ad ￥�������.txt
575 ng 就/d 是/vshi 他/rr 开/v 的/ude1 ，/wd 为了/p 方便/v 结交/v 南来北往/vl 的/ude1 人/n ，/wd 替/p 日本/nsf 军队/n 收集/v 情报/n 。/wj ￥�������.txt
576 v 学校/n ，/wd 一路/mq 走/v ，/wd 一路/mq 上/f 我/rr 都/d 会/v 左顾右盼/vl 留意/v 每/rz 一个/mq 迎面/d 过来/vf 的/ude1 人/n ，/wd 特别/ ￥�������.txt
577 ude1 眼睛/n ，/wd 是/vshi 躲闪/vi 着/uzhe 我/rr ，/wd 还是/c 鬼鬼祟祟/al 地/ude2 研究/v 我/rr 。/wj 我/rr 期盼/v 有/vyou 那么/rz 一 ￥�������.txt
578 研究/v 我/rr 。/wj 我/rr 期盼/v 有/vyou 那么/rz 一个/mq 间谍/n 一不小心/dl 被/pbei 我/rr 发现/v ，/wd 我/rr 赶快/d 喊/v 路/n 人/n 帮忙 ￥�������.txt
579
入冬/vi ，/wd 从/p 战场/n 上/f 传来/v 的/ude1 消息/n 让/v 人/n 忧心忡忡/vl 。/wj 远征军/n 第一/m 次/qv 入/v 缅/b 被/pbei 日军/n 击败/v ￥�������.txt

580 /a 。/wj
而/cc 在/p 日军/n 大本营/n 中/f ，/wd 日本/nsf 首相/n 东条英机/nrj 因为/p 急于/vd 结束/v 中国/ns 战争/n ，/wd 不惜/v 代价/n 地/u ¥�������.txt
581 中国/ns 战争/n ，/wd 不惜/v 代价/n 地/ude2 从/p 日本/nsf 运送/v 战略物资/nl ，/wd 从/p 南方/s 各/rz 占领区/n 抽调/v 部队/n ，/wd 派遣/v 大 ¥�������.txt
582 /v 了/ule 几/m 年/qt 之前/f 战争/n 爆发/v 时/ng 的/ude1 那种/r 茫然无措/al 。/wj
学校/n 里/f 新/a 来/vf 了/ule 一个/mq 伙夫/n ，/wd ¥�������.txt
583 z ，/wd 眼皮/n 总是/d 耷拉/v 着/uzhe ，/wd 从/p 眼/n 缝/n 里/f 偷偷摸摸/dl 看/v 人/n ，/wd 鬼祟/a 得/ude3 很/d 。/wj 还有/v ，/wd 他/ ¥�������.txt
584 /v 得/ude3 古怪/a ，/wd 手艺/n 却/d 好/a ，/wd 随便/ad 一个/mq 家常豆腐/nms 或者/c 蚂蚁上树/nms ，/wd 能/v 把/pba 我们/rr 吃/v 得/ude3 ¥�������.txt
585 ，/wd 手艺/n 却/d 好/a ，/wd 随便/ad 一个/mq 家常豆腐/nms 或者/c 蚂蚁上树/nms ，/wd 能/v 把/pba 我们/rr 吃/v 得/ude3 嗞/o 哈/v 嗞/o 哈 ¥�������.txt
586 /ng 瞪/v 着/uzhe 一/m 双/q 碧蓝/z 澄澈/z 的/ude1 眼睛/n ，/wd 目不转睛/dl 地/ude2 看/v 我/rr ，/wd 嘴里/s 发出/v "/wyz 嗯/e 嗯/e " ¥�������.txt
587 /nz 长成/v 一个样/nl ？/ww
"/wyz 对不起/v ，/wd "/wyy 她/rr 小心翼翼/dl 道歉/vi ，/wd "/wyz 我/rr 只是/d 说/v ，/wd 有/vyou 那么/
¥�������.txt
588 师傅/n "/wyy ，/wd 他/rr 必然/b 浑身/n 一/m 震/vi ，/wd 而后/d 毕恭毕敬/al 地/ude2 站/vi 下/f ，/wd 垂/v 手/n 而/cc 立/v ，/wd 偷偷/ ¥�������.txt
589 是/vshi 铁/n 打/v 的/ude1 ！/wt "/wyz
他/rr 以为/v 我/rr 处心积虑/dl 是/vshi 要/v 进/vf 窝棚/n 偷/v 他/rr 的/ude1 东西/n 吧/y
¥�������.txt
590 ，/wd 住/vi 这么/rz 破/v 一个/mq 窝棚/n ，/wd 有/vyou 什么/ry 见不得人/vl 的/ude1 家什/n 值得/v 他/rr 如此/rzv 警惕/v ？/ww
还有/v ， ¥�������.txt
591 会/v 一/m 步/qv 三/m 回头/vi 地/ude2 溜/v 出/vf 学校/n ，/wd 急急忙忙/z 不/d 知道/v 去/vf 了/ule 哪儿/rys 。/wj 两/m 点/qt 钟/n 正是 ¥�������.txt
592 ，/wd 没/v 办法/n 跟/v 在/p 他/rr 身后/f 一/m 看/v 究竟/n 。/wj 好不容易/dl 挨/v 到/v 下/vf 课/n ，/wd 奔/v 去/vf 后面/f 伙房/n ，/wd ¥�������.txt
593
他/rr 耸/vg 耸肩/v ，/wd 不再/d 理/v 我/rr 。/wj
我/rr 姐/n 幸灾乐祸/vl （/wkz 她/rr 最/d 喜欢/vi 幸灾乐祸/vl ）/wky 说/v ：/wm "/ ¥�������.txt
594 我/rr 。/wj
我/rr 姐/n 幸灾乐祸/vl （/wkz 她/rr 最/d 喜欢/vi 幸灾乐祸/vl ）/wky 说/v ：/wm "/wyz 橙子/n ，/wd 姐/n 给/p 你/rr 出/ ¥�������.txt
595 r 陪/v 你/rr 走/v 一/m 趟/qv ？/ww "/wyz
我/rr 姐/n 有点/d 阴阳怪气/al ：/wm "/wyz 最/d 好/a 不/d 过/uguo 啦/y ，/wd 弄/v 不好/
¥�������.txt
596 rr 故意/d 上/f 去/vf 拉/v 住/vi 沈天路/ns 的/ude1 胳膊/n ，/wd 亲密无间/vl 地/ude2 往/p 外/f 走/v 。/wj 沈天/nr 路/n 窘/a 得/ude3 脸 ¥�������.txt
597 他/rr 现在/t 应该/v 跟/v 上/vf 。/wj 我们/rr 跨过/v 矮墙/n ，/wd 悄无声息/vl 地/ude2 上/vf 了/ule 大/a 路/n 。/wj 沈天/nr 路/n 步子/n ¥�������.txt
598 显/ad 是/vshi 怕/v 有/vyou 人/n 跟踪/vn 。/wj 如果/c 是/vshi 正大光明/bl 走路/vi 办事/vi ，/wd 他/rr 干吗/v 这么/rz 心虚/a 鬼祟/a ？/w ¥�������.txt
599 我/rr 看见/v 董/nr1 师傅/n 走/v 到/v 其中/rz 一/m 家门口/n ，/wd 迫不及待/dl 地/ude2 抬/v 手/n 拍/v 一/m 拍/q 门/n 。/wj 片刻/m 门/q 就 ¥�������.txt
600 "/wyz 快/a 呀/y ！/wt "/wyy 我/rr 催/v 他/rr 。/wj
他/rr 若有所思/vl ：/wm "/wyz 你/rr 闻/v 见/v 什么/ry 味道/n 没有/v ？/ww " ¥�������.txt
601 怕/v 你们/rr 学校/n 知道/v 了/ule 要/v 开除/v ，/wd 自然/d 就/d 偷偷摸摸/dl ，/wd 人之常情/nl 。/wj "/wyz
老/a 天/qt ，/wd 明明/d 一个 ¥�������.txt
602 /n 知道/v 了/ule 要/v 开除/v ，/wd 自然/d 就/d 偷偷摸摸/dl ，/wd 人之常情/nl 。/wj "/wyz
老/a 天/qt ，/wd 明明/d 一个/mq 大/a 烟鬼/n ¥�������.txt
603 外/f 飞/vi 。/wj "/wyz
我/rr 爸/n 埋头/vd 扒/v 饭/n ，/wd 不置可否/vl 。/wj
娘/n 看看/v 沈天路/ns ，/wd 再/d 转头/v 看看/v 我/rr ¥�������.txt
604 ude1 时候/n 。/wj 生/v 在/p 战争/n 年代/n 的/ude1 小孩/n ，/wd 颠沛流离/vl ，/wd 缺/v 吃/v 少/ad 穿/v ，/wd 站/vi 出来/vf 一个/mq 比/ ¥�������.txt
605 不能/v 再/d 丑/a 的/ude1 麻/n 母鸡/n ，/wd 小弟/n 玩/v 得/ude3 忘乎所以/vl 时/ng ，/wd 麻/n 母鸡/n 正在/d 林子/n 里/f 撅/v 着/uzhe 屁股 ¥�������.txt

606 裤子/n ，/wd 从/p 屁股/n 到/v 脚/n ，/wd —/m 条/q 棉/ng 裤腿/n 热气腾腾/vl 。/wj
我/rr 娘/n 先/d 还/d 不/d 肯/v 承认/v ：/wm "/wyz ¥������.txt
607 d 还/d 不/d 肯/v 承认/v ：/wm "/wyz 学校/n 放假/vi 呢/y ，/wd 家家户户/nl 的/ude1 小/a 孩子/n 都/d 在/p 外面/f 耍/v 呢/y ，/wd 妹妹/n ¥������.txt
608 路/n 我/rr 都/d 难/ad 认错/vi 。/wj "/wyz
我/rr 娘/n 就/d 无话可说/vl 了/y 。/wj 不过/c 她/rr 拿/v 脚尖/n 踢/v —/m 下/f 那/rzv
¥������.txt
609 里/s 还/d 真/d 是/vshi 拿/v 不/d 出/vf 钱/n 。/wj 几/m 番/qv 讨价还价/vl ，/wd 女人/n 同意/v 我/rr 娘/n 脱/v 下/vf 她/rr 身上/s 的/u ¥������.txt
610 邻居/n 看见/v ，/wd 像/v 什么/ry 话/n 呢/y ？/ww "/wyz
我/rr 无话可说/vl ，/wd 只好/d 朝/p 小弟/n 跺脚/vi ：/wm "/wyz 哭/v ！/wt 哭 ¥������.txt
611 /ag 和/cc 小弟/n 嫌/ng 不/d 好吃/a 剩下/v 的/ude1 ，/wd 他/rr 二话不说/vl ，/wd 统统/d 划/v 拉/v 过去/vf ，/wd 拨/v 进/vf 自己/rr 碗/ ¥������.txt
612 磨/v 着/uzhe 沈天路/ns 的/ude1 秘密/n 。/wj 他/rr 对/p 别人/rr 瞒天过海/vl 可以/v ，/wd 对/p 我/rr 也/d 守口如瓶/vl ，/wd 这/rzv 让/v ¥������.txt
613 /wj 他/rr 对/p 别人/rr 瞒天过海/vl 可以/v ，/wd 对/p 我/rr 也/d 守口如瓶/vl ，/wd 这/rzv 让/v 我/rr 心里/s 实在/d 郁闷/a 。/wj 我们/rr ¥������.txt
614 d 把/pba 衣服/n 拉/v 好/a ，/wd 坐/v 直/d 了/ule 身体/n ，/wd 不声不响/vl 地/ude2 瞪视/v 我/rr ，/wd 等/udeng 我/rr 发问/vi 。/wj ¥������.txt
615 钱/n 啊/y ？/ww "/wyz 我/rr 对/p 家里/s 的/ude1 事情/n 一向/d 糊里糊涂/z 。/wj
他/rr 摇摇/v 手/n ：/wm "/wyz 娘/n 的/ude1 皮/n 坎 ¥������.txt
616 腿/n ，/wd 谁/ry 要/v 你/rr 谁/ry 倒霉/a 。/wj "/wyz
我/rr 想想也是/vl ，/wd 这/rzv 一点/mq 我/rr 有/vyou 自知之明/nl 。/wj 我/rr ¥������.txt
617 "/wyz
我/rr 想想也是/vl ，/wd 这/rzv 一点/mq 我/rr 有/vyou 自知之明/nl 。/wj 我/rr 帮/v 他/rr 穿/v 好/a 衣服/n ，/wd 坐/v 下来/vf ¥������.txt
618 q 婶娘/n 。/wj 那个/rz 婶婶/n 真是/d 不好惹/a ，/wd 知道/v 我们/rr 志在必得/vl ，/wd 狮子/n 大/a 开口/vi ，/wd 要/v 了/ule 个/q 吓/v 死/v ¥������.txt
619 ule 个/q 吓/v 死/v 人/n 的/ude1 价/n 。/wj 我们/rr 两/m 个/q 据理力争/vl ，/wd 最后/f 还是/d 掏/v 出/vf 了/ule 能/v 买/v 到/v 五/m ¥������.txt
620 庆/ns 寄/v 来/vf 的/ude1 —/m 封/q 信/n ，/wd 真正/d 是/vshi 晴天霹雳/nl ：/wm 她/rr 已经/d 跟/v 着/uzhe 李胜利/nr 去/vf 了/ule 延安 ¥������.txt
621 颤抖/vi 得/ude3 像/v —/m 片/q 风/n 中/b 树叶/n ：/wm "/wyz 好不容易/dl 从/p 南京/ns 逃/v 出/vf 来/vf 了/y ，/wd 她/rr 又/d 要/v ¥������.txt
622 f 饭碗/n ：/wm "/wyz 行/vi 了/y ，/wd 吃饭/vi 。/wj "/wyz
第二十一/m 章/q ·/w 参军/vi
一九四四年/t ，/wd 抗战/vi 七/m 年/qt 之后/f ¥������.txt
623 战役/n 中/f 打/v 了/ule 一个/mq 大/a 大/a 的/ude1 败仗/n ，/wd 恼羞成怒/vl ，/wd 遂/d 制订/v 出/vf 疯狂/a 的/ude1 作战/vn 计划/n ，/wd ¥������.txt
624 南/ns 重镇/ns 独山/ns ，/wd 直接/ad 威逼/v 陪都/n 重庆/ns 。/wj
举国上下/bl ，/wd 人人/n 皆/d 惊/v 。/wj
华西/ns 坝上/s 的/ude1 年轻人/ ¥������.txt
625 v 着/uzhe 这些/rz 学生/n 去/vf 当/v 炮灰/n ，/wd 否则/c 我们/rr 千辛万苦/nl 办/v 大学/n 是/vshi 为了/p 什么/ry ？/ww 学生/n 们/k 读/v 一 ¥������.txt
626 rz 不/d 争/v ；/wf 哥哥/n 如果/c 报了名/v ，/wd 那/rzv 是/vshi 生离死别/nl ，/wd 胜利/vd 返家/vi 的/ude1 可能性/n 微乎其微/vl 。/wj
那/ ¥������.txt
627 /rzv 是/vshi 生离死别/nl ，/wd 胜利/vd 返家/vi 的/ude1 可能性/n 微乎其微/vl 。/wj
那/rzv 几/m 天/qt 真/d 是/vshi 我们/rr 家里/s 沉重/ ¥������.txt
628 他/rr ./wj ./wj ./wj ./wj ./wj ./wj "/wyy 我/rr 娘/n 忧心忡忡/vl 看/v 我/rr 爸/n 。/wj 她/rr 不/d 敢/v 把/pba "/wyz 参军/ ¥������.txt
629 vf 的/ude1 肌肉/n ：/wm "/wyz 瞧瞧/v 我/rr 这样/rzv 的/ude1 运动健将/n ，/wd 英雄/n 终于/d 有/vyou 了/ule 用武之地/nl 。/wj "/wyz ¥������.txt
630 这样/rzv 的/ude1 运动健将/n ，/wd 英雄/n 终于/d 有/vyou 了/ule 用武之地/nl 。/wj "/wyz
我/rr 哥/n 一直/d 仰慕/v 远征军/n 的/ude1 英名 ¥������.txt
631 入伍/vi 之后/f 即/v 被/pbei 派/nms 往/p 印度/nsf 受训/vn 。/wj 同年七月/t ，/wd "/wyz 七七/m "/wyy 抗战/vi 七/m 周年/q 纪念日/n ，/wd ¥������.txt
632 /rr 的/ude1 大哥/n ，/wd 十九/m 岁/qt ，/wd 英魂/n 埋葬/v 在/p 异国他乡/nl 的/ude1 土地/n 。/wj
同一个/b 八月/t ，/wd 沈天路/ns 被/pbe ¥������.txt
633 离/v 家/n 去/vf 延安/ns 的/ude1 信件/n 之后/f ，/wd 我/rr 爸/n 处之泰然/vl 。/wj 哥哥/n 报名/vi 参军/vi ，/wd 我/rr 爸/n 觉得/v 理所应当/ ¥������.txt

634 处之泰然/vl 。/wj 哥哥/n 报名/vi 参军/vi ， /wd 我/rr 爸/n 觉得/v 理所应当/dl 。/wj 唯有/v 沈天路/ns 报考/v 航校/n ， /wd 仿佛/d 最后/f 击中/v ¥�������.txt
635 /d 开口/vi 。/wj
我/rr 爸/n 于是/cc 明白/v 一切/rz 都/d 已经/d 板上钉钉/vl 。/wj 他/rr 是/vshi 明白/v 沈/nr1 天/n 路/n 的/ude1 脾气/ ¥�������.txt
636 穿/v 心/n ， /wd 这/rzv 一/m 份/q 无言/z 的/ude1 爱意/n 他/rr 无论如何/dl 不/d 能/v 辜负/v 。/wj 而/cc 我/rr 娘/n ， /wd 算/v 起来/v ¥�������.txt
637 ， /wd 他/rr 双手/n 插/v 袋/ng 默默/d 地/ude2 走/v ， /wd 我/rr 不知所措/vl 地/ude2 跟/v 在/p 他/rr 身后/f ， /wd 时不时/d 地/ude2 偷看/ ¥�������.txt
638 rr 娘/n 总是/d 对/p 他/rr 另/rz 眼/n 相/d 看/v 。/wj
我们/rr 不知不觉/dl 走/v 到/v 了/ule 河边/s 的/ude1 杂/a 树林/n 。/wj 沈天路/ns ¥�������.txt
639 的/ude1 杂/a 树林/n 。/wj 沈天路/ns 站/vi 了/ule 一下/mq ， /wd 若有所思/vl 地/ude2 朝/p 树林/n 里/f 看/v 了/ule 一/m 眼/q 。/wj 我/r ¥�������.txt
640 曾经/d 被/pbei 小/a 日本/nsf 飞机/njtgj 炸/v 塌/v 一角/n 又/d 马马虎虎/z 修复/v 起来/vf 的/ude1 天主教堂/n 。/wj 九/m 岁/qt 那年/t ， /w ¥�������.txt
641 机/njtgj 炸/v 塌/v 一角/n 又/d 马马虎虎/z 修复/v 起来/vf 的/ude1 天主教堂/n 。/wj 九/m 岁/qt 那年/t ， /wd 沈天路/ns 陪/v 我/rr 走/v 夜/t ¥�������.txt
642 /n 钩/n 住/vi 我/rr 的/ude1 头发/n ， /wd 吓/v 得/ude3 我/rr 魂飞魄散/al ， /wd 我/rr 想/v 拉/v 住/vi 他/rr 的/ude1 手/n ， /wd 他/ ¥�������.txt
643 f 这个/rz 家/n 就/d 交/v 给/p 你/rr 了/y 。/wj "/wyz
我/rr 急急忙忙/z 抗议/v ： /wm "/wyz 不/d 可以/v 这样/rzv ！ /wt 你/rr 和/cc ¥�������.txt
644 说话/vi 算数/vi ， /wd 一定/d 一定/d 要/v 回家/vi ！ /wt "/wyz
第二十二/m 章/q ·/w 两地/n 书/n
亲爱的/n 橙子/n ： /wm
到/p 昆明/ns 一/¥�������.txt
645 是/vshi 每天/r 的/ude1 一万/m 米/q 跑/v ， /wd 就/d 让/v 他/rr 精疲力尽/vl 。/wj 现在/t 教官/n 允许/v 他/rr 减/v 为/p 五/m 千/m 米/q 跑¥�������.txt
646 f 我/rr 不/d 应该/v 这么/rz 写/v ， /wd 这/rzv 会/v 让/v 你/rr 担惊受怕/vl 。/wj 我/rr 承认/v 我/rr 有时候/d 喜欢/vi 胡/nr1 思/v 乱/d ¥�������.txt
647 生/n 也好/y 大好/a 开阔/a 。/wj 我/rr 看到/v 了/ule 机场/n 两旁/f 一望无际/vl 的/ude1 茅草/n 地/ude2 ， /wd 它们/rr 被/pbei 河流/n 湖泊/n ¥�������.txt
648 。/wj
飞越/v 驼峰/n 的/ude1 时候/n ， /wd 我/rr 看到/v 了/ule 连绵不断/vl 的/ude1 雪山/n ， /wd 太/d 壮观/a 了/y 呀/y ！ /wt 像/p 什么/ ¥�������.txt
649 /v 一下/mq ， /wd 想/v 了/ule 半/m 天/qt 也/d 找/v 不/d 出/vf 称心如意/vl 的/ude1 词句/n 。/wj 你/rr 看/v 我/rr 多/d 无/v 趣/ng ， / ¥�������.txt
650 /v 有/vyou 马克/n 这样/rzv 的/ude1 幸运/a ？ /ww
啊啊/e ， /wd 一不小心/dl 又/d 说/v 起/vf 这个/rz ， /wd 打/v 嘴/n 打/v 嘴/n 。/wj
¥�������.txt
651 吃/v ？ /ww 我/rr 稍微/d 嚼/v 一下/mq 就/d 闭/v 着/uzhe 眼睛/n 囫囵吞枣/vl 地/ude2 咽/v ， /wd 而后/d 猛/d 喝/vg 水/n ， /wd 压/v 住/v ¥�������.txt
652 这/rzv 只/q 羊/n ！ /wt
爸爸/n 让/v 我/rr 告诉/v 你/rr ， /wd 无论如何/dl 要/v 学会/v 吃/v 牛排/n ， /wd 把/pba 身体/n 养/v 得/ude3 更¥�������.txt
653 到处/d 都/d 是/vshi 飞机/njtgj 的/ude1 话/n ， /wd 就/d 好像/v 时时刻刻/dl 都/d 能/v 看见/v 你/rr 。/wj 她/rr 还/d 给/p 小弟/n 折/vi ¥�������.txt
654 jtgj 睡觉/vi ， /wd 醒/v 来/vf 一/m 看/v 压/v 扁/a 了/y ， /wd 号啕大哭/vl ， /wd 好/a 伤心/a 呢/y ， /wd 小/a 素/ag 只好/d 又/d 给/p 他 ¥�������.txt
655 大家/rr 都/d 很/d 想/v 你/rr 。/wj 所有/b 的/ude1 人/n ， /wd 每时每刻/bl 。/wj
黄/nr1 橙子/n
亲爱的/n 橙子/n ： /wm
我/rr 给/p 小弟 ¥�������.txt
656 a 地/ude2 看/v 我/rr 脚下/f 的/ude1 大地/n ， /wd 看/v 那些/rz 五颜六色/bl 的/ude1 土地/n 河流/n 山川/n 。/wj 世界/n 真/d 美/a 。/wj 如¥�������.txt
657 le 再/d 去/vf ， /wd 那/rzv 时候/n 他/rr 才/d 是/vshi 一个/mq 扬眉吐气/vl 的/ude1 中国/ns 人/n 。/wj
黄/nr1 橙子/n
亲爱的/n 橙子/n ¥�������.txt
658 而且/c ， /wd 我们/rr 都/d 已经/d 写/v 好/a 了/ule 遗书/n ， /wd 迫不及待/dl 地/ude2 盼望/v 投入/vn 战斗/vn ， /wd 誓死/d 报国/vi 。/wj 所¥�������.txt
659 ude1 英勇/a ， /wd 鬼子/n 的/ude1 飞机/njtgj 在/p 我/rr 眼前/s 灰飞烟灭/vl ； /wf 也/d 说不定/vl

我/rr 已经/d 中弹/vi 牺牲/v ，/wd 呼应/v ¥������.txt
660 wn 飞机/njtgj 和/cc 炸弹/n ，/wd 当/v 与/p 敌人/n 兵舰/n 阵地/n 同归于尽/vl 。/wj
橙子/n 小妹/n --/wp 请/v 允许/v 我/rr 像/p 你/rr 的 ¥������.txt
661 曾经/d 多么/d 快乐/a 地/ude2 生活/vi 过/uguo 。/wj
沈天路/ns
第二十三/m 章/q ·/w 欢乐/a 时光/n
在/p 我/rr 又/d 一/m 次/qv 见到/v 沈 ¥������.txt
662 d 弹琴/vi ，/wd 种/q 花/n ，/wd 儿孙/n 绕/v 膝/ng ，/wd 享受/v 天伦之乐/nl ；/wf 也许/d 她/rr
早/ad 已经/d 离开/v 人世/n ，/wd 骨/ng 殖 ¥������.txt
663 一直/d 是/vshi 那个/rz 梳/v 两/m 条/q 金/b 黄色/n 发辫/n 、/wn 大呼小叫/vl 地/ude2 看/v 着/uzhe
我/rr 爬树/v 下/vf 河/n 的/ude1 美丽 ¥������.txt
664 m 天/qt 。/wj 这/rzv 三/m 天/qt 里/f 沈/nr1 天/n 路/n 简直/d 手忙脚乱/al ，/wd 不/d 知道/v 该/v
怎么/ryv 招待/v 我/rr 。/wj 他/rr 攒 ¥������.txt
665 的/ude1 嘴巴/n 。/wj "/wyz 橙子/n 橙子/n ，/wd "/wyy 他/rr 可怜巴巴/z 地/ude2 哀求/v 我/rr
，/wd "/wyz 别/d 这么/rz 顽皮/a 了/ul ¥������.txt
666 /v 。/wj 钱/n 沪/b 生/v 穿/v 着/uzhe 一/m 身/q 飞行服/n ，/wd 一本正经/al 地/ude2 对/p 我/rr 敬
礼/vi ，/wd 称呼/v 我/rr "/wyz 王/n ¥������.txt
667 t 的/ude1 。/wj 钱/n 沪/b 生/v 很/d 为/v 自己/rr 羞愧/v ，/wd 面红耳赤/al ，/wd 不/d 敢/v 再/d 开
口/vi 讲/v 沪/b 语/ng 了/y ，/wd 转 ¥������.txt
668 /a 睫毛/n ，/wd 瞳仁/n 特别/d 黑/a ，/wd 看/v 人/n 时/ng 显得/v 一往情深/vl 。/wj 云南/ns 高原/n
那么/rz 大/a 的/ude1 太阳/n ，/wd 也/d ¥������.txt
669 m 回/qv 对/p 长官/n 提出/v 非分/b 要求/n ，/wd 当时/t 就/d 觉得/v 无地自容/al 。/wj 跟/p 着/uzhe
他/rr 又/d 笑嘻嘻/z 地/ude2 说/v ，/wd ¥������.txt
670 /p 那个/rz 空军/n 营地/n 的/ude1 三/m 天/qt 时间/n ，/wd 我/rr 时时刻刻/dl 都/d 沐浴/vi 着/uzhe
这样/rzv 的/ude1 温暖/an ，/wd 让/v ¥������.txt
671 。/wj "/wyz 起码/d 不/d 下/vf 五/m 次/qv ，/wd 沈天/nr 路/n 唉声叹气/vl 地/ude2 表达/v 了/ule
他/rr 的/ude1 担忧/vn 。/wj
我/rr ¥������.txt
672 rr 想/v 说/v 什么/ry ，/wd 又/d 不/d 忍心/v 说/v 出来/vf ，/wd 忧心忡忡/vl 地/ude2 盯住/v 我/rr
的/ude1 眼睛/n 。/wj 我/rr 问/v 他/r ¥������.txt
673 很/d 业余/b 。/wj "/wyz
我/rr 想/v 拿到/v 美女/n 画/n ，/wd 甜言蜜语/nl 无/v 师/ng 自/p 通/v 地/ude2 往/p 外/f 冒/v ：/wm
"/wyz¥������.txt
674 yy 地/ude2 笑/v 起来/vf ，/wd 嘴角/n 居然/d 还/d 现出/v 一个/mq 隐隐约约/z 的/ude1 酒窝/n
。/wj
慰问/v 团里/n 的/ude1 男生/n 跟/p 飞行员 ¥������.txt
675 /wyz 你们/rr 是/vshi 天使/n 。/wj "/wyz
我/rr 和/cc 同伴/n 勾肩搭背/vl 坐/v 在/p 新/a 铺/v 的/ude1 床单/n 上/f ，/wd 笑/v 得/ude
¥������.txt
676 l 坐/v 在/p 新/a 铺/v 的/ude1 床单/n 上/f ，/wd 笑/v 得/ude3 哗啦哗啦/o 。/wj
"/wyz 真/a 的/ude1 ，/wd 你们/rr 一/d 来/vf ，/wd ¥������.txt
677 我/rr 在/p 沈天路/ns 的/ude1 眼睛/n 里/f 还/d 是/vshi 一个/mq 傻里傻气/z 的/ude1 瓷/ng 娃娃/n
，/wd 他/rr 小心翼翼/dl 地/ude2 呵护/v ¥������.txt
678 /d 是/vshi 一个/mq 傻里傻气/z 的/ude1 瓷/ng 娃娃/n ，/wd 他/rr 小心翼翼/dl 地/ude2 呵护/v 我/rr
，/wd 爱惜/v 我/rr ，/wd 捧/v 着/uzh ¥������.txt
679 下/vf 眼皮/n ，/wd 自己/rr 都/d 能/v 看见/v 自己/rr 鼻尖/n 上/f 星星点点/bl 的/ude1 细/a 汗/n
。/wj 在/p 我/rr 从/p 幕后/s 走/v 上前/v ¥������.txt
680 d 听/v 不/d 见/v ，/wd 我/rr 像/v 是/vshi 被/pbei 一/m 片/q 五彩斑斓/al 的/ude1 祥云/nr2 支/q 托/v
着/uzhe 一样/a ，/wd 迷迷糊糊/z ¥������.txt
681 /q 五彩斑斓/al 的/ude1 祥云/nr2 支/q 托/v 着/uzhe 一样/a ，/wd 迷迷糊糊/z 坐/v 到/v 了/ule 钢
琴/n 前面/f 。/wj
在/p 我/rr 坐/v 下来/v ¥������.txt
682 ！/wt 我/rr 被/pbei 音乐/n 陶醉/vi ，/wd 被/pbei 礼堂/n 里/f 异乎寻常/al 的/ude1 寂静/a 陶醉/vi
，/wd 也/d 被/pbei 我/rr 自己/rr 的 ¥������.txt
683 哗哗/o 地/ude2 响起/v 。/wj 我/rr 起立/vi ，/wd 傻笑/vi ，/wd 不知所措/vl 。/wj 负责/v 舞台/n 监
督/vn 的/ude1 同学/n 假装/v 上台/vi 搬动 ¥������.txt
684 /d 找到/v 。/wj 他/rr 会/v 去/vf 了/ule 哪儿/rys ？/ww 我/rr 心慌意乱/vl 。/wj
奔/v 下/vf 舞台/n ，/wd 一/m 出/vf 边门/n ，/wd 我/ ¥������.txt
685 /rr 彼此/rr 的/ude1 气息/n 送/v 到/v 对方/n 耳朵/n 里/f 。/wj
好不容易/dl ，/wd 我/rr 都/d 要/v 被/pbei 他/rr 的/ude1 肃穆/a 和/cc ¥������.txt
686 ，/wd 他/rr 才/d 吐/v 一/m 口/q 气/n ，/wd 伸出/v 臂膀/n ，/wd 小心翼翼/dl 地/ude2 揽/v 我/rr

到/v 他/rr 胸前/s 。/wj
"/wyz 橙子/n ¥�������.txt
687 的/ude1 反/vi 转/v 。/wj 中国/ns 战区/n 的/ude1 形势/n 同样/d 令人鼓舞/vl ：/wm 湘西/ns 大捷/n ，/wd 福州/ns 收复/v ，/wd 南宁/ns 已/d ¥�������.txt
688 ，/wd 去年/t 的/ude1 这个/rz 时候/n ，/wd 石榴花/n 开/v 得/ude3 七零八落/al ，/wd 结果/n 我/rr 姐/n 去/vf 了/ule 延安/ns 之后/f 再/d 无 ¥�������.txt
689 le 延安/ns 之后/f 再/d 无/v 音信/n ，/wd 我/rr 哥/n 牺牲/v 在/p 异国他乡/nl 的/ude1 陌生/a 之/uzhi 地/n 。/wj
小素/nr2 在/p 走廊/n 上 ¥�������.txt
690 的/ude1 陌生/a 之/uzhi 地/n 。/wj
小素/nr2 在/p 走廊/n 上/f 满头大汗/al 地/ude2 跳绳/vi ，/wd 听见/v 娘/n 在/p 自言自语/nl ，/wd 撇/ ¥�������.txt
691 p 走廊/n 上/f 满头大汗/al 地/ude2 跳绳/vi ，/wd 听见/v 娘/n 在/p 自言自语/nl ，/wd 撇/v 撇嘴/vi 批评/v 她/rr ：/wm "/wyz 这/rzv 是/vs ¥�������.txt
692 /wm "/wyz 你/rr 小孩子/n 家家/q ，/wd 懂/v 个/q 什么/ry ？/ww 自古以来/dl 草木/n 人命/n 都/d 是/vshi 有/vyou 定/v 数/n 的/ude1 ，/w ¥�������.txt
693 发起/v 进攻/vn ，/wd 要/v 争夺/v 南阳/ns 及/cc 老河口/n 两/m 个/q 军事基地/nl 。/wj 第五/m 战区/n 的/ude1 中国/ns 军队/n 抵抗/vn 不/d 住/v ¥�������.txt
694 j 日军/n 攻陷/v 南阳/ns 和/cc 老河口/n 之后/f ，/wd 中国/ns 军队/n 重整旗鼓/vl ，/wd 又/d 开始/v 组织/n 反攻/vn ，/wd 一点/mq 一点/mq 地/ud ¥�������.txt
695 /wj 一/m 场/qv 接/v 一/m 场/qv 的/ude1 战斗/vn 打/v 得/ude3 艰苦卓绝/bl ，/wd 彼此/rr 都/d 难/a 有/vyou 喘息/vn 之/uzhi 时/ng 。/ ¥�������.txt
696 又/d 俯冲/vi ，/wd 一头/d 撞/v 了/ule 上去/vf ，/wd 跟/p 敌机/n 同归于尽/vl 。/wj
沈天路/ns 的/ude1 遗书/n 和/cc 遗物/n 在/p 八月份/t 才¥�������.txt
697 一/m 位/q 军容/n 整齐/a 神情/n 肃穆/a 的/ude1 空军/n 上尉/n 手/n 捧沈天路/ns 的/ude1 遗物/n 站/vi 在/p 我/rr 娘/n 面前/f 时/ng ，/wd 娘 ¥�������.txt
698 然/ad 之间/f 都/d 变/v 得/ude3 懂事/a 了/y ，/wd 他们/rr 一直/d 提心吊胆/vl 地/ude2 围/v 在/p 她/rr 身边/s ，/wd 不/d 停歇/vi 地/ude2 ¥�������.txt
699 徐/nr1 伯伯/n 两/m 家/q 跟/p 我们/rr 同船/n 而/cc 行/vi 。/wj 范舒文一/nr 家/n 更/d 早/a 之前/f 绕道/v 越南/nsf 回/v 了/ule 美国/nsf ¥�������.txt
700 d 我们/rr 在/p 傅厚岗/nr 的/ude1 家里/s 来/vf 了/ule 一/m 位/q 不速之客/nl ，/wd 小小的/z 个子/n ，/wd 穿/v 灰色/n 中山装/n ，/wd 留/v 着 ¥�������.txt
701 m 年/qt 时间/n ，/wd 没有/d 收到/v 过/uguo 我/rr 姐/n 的/ude1 一丁点儿/mq 消息/n ，/wd 我/rr 娘/n 的/ude1 心/n 早/tg 已经/d 被/pbei¥�������.txt
702 那/rzv 时候/n 救护/vn 系统/n 很/d 不/d 发达/a ，/wd 学生/n 们/k 急急忙忙/z 从/p 学校/n 食堂/n 借/v 来/vf 平板车/n 拖/v 他/rr 去/vf 医院/n ¥�������.txt
703 z 汤普森/nrf "/wyy 弹起/v 。/wj 一/m 年/qt 之后/f 我/rr 能够/v 断断续续/dl 弹/v 出/vf 《/wkz 野/b 蜂/ng 飞舞/v 》/wky 。/wj 到/p 现在 ¥�������.txt
704 》/wky 是/vshi 我/rr 的/ude1 保留/vn 曲目/n ，/wd 我/rr 在/p 教职员工/n 联欢会/n 上/f 弹/v 过/uguo ，/wd 在/p 退休/vn 工作者/n 协会/n ¥�������.txt
705 d 都/d 是/vshi 这个/rz 年龄/n ，/wd 美好/a ，/wd 绚烂/an ，/wd 热气腾腾/vl 。/wj
¥�������.txt
