# Supplementary material for: Exploring translator’s style in children’s literature: A case study of Nicky Harman’s English translations of Huang Beijia’s two works
Source: PLoS One. 2026 Jun 2;21(6):e0350245. doi: 10.1371/journal.pone.0350245 (PMC13229296; doi:10.1371/journal.pone.0350245)
Supplement: S7 File — (ZIP) [file pone.0350245.s010.zip › data/NIPLR-based Word Segmentation and Part-of-Speech (POS) Tagging.docx]

计算所汉语词性标记集

Version 3.0

制订人：刘群 张华平 张浩

1.          名词  (1个一类，7个二类，5个三类)

名词分为以下子类：

n 名词

nr 人名

nr1 汉语姓氏

nr2 汉语名字

nrj 日语人名

nrf 音译人名

ns 地名

nsf

 音译地名

nt 机构团体名

nz 其它专名

nl 名词性惯用语

ng 名词性语素

2.          时间词(1个一类，1个二类)

t 时间词

tg 时间词性语素

3.          处所词(1个一类)

s 处所词

4.          方位词(1个一类)

f 方位词

5.          动词(1个一类，9个二类)

v 动词

vd 副动词

vn 名动词

vshi 动词“是”

vyou 动词“有”

vf 趋向动词

vx 形式动词

vi 不及物动词（内动词）

vl 动词性惯用语

vg 动词性语素

6.          形容词(1个一类，4个二类)

a 形容词

ad 副形词

an 名形词

ag 形容词性语素

al 形容词性惯用语

7.          区别词(1个一类，2个二类)

b 区别词

bl 区别词性惯用语

8.          状态词(1个一类)

z 状态词

9.          代词(1个一类，4个二类，6个三类)

r 代词

rr 人称代词

rz 指示代词

rzt 时间指示代词

rzs 处所指示代词

rzv 谓词性指示代词

ry 疑问代词

ryt 时间疑问代词

rys 处所疑问代词

ryv 谓词性疑问代词

rg 代词性语素

10.     数词(1个一类，1个二类)

m 数词

mq 数量词

11.     量词(1个一类，2个二类)

q 量词

qv 动量词

qt 时量词

12.     副词(1个一类)

d 副词

13.     介词(1个一类，2个二类)

p 介词

pba 介词“把”

pbei 介词“被”

14.     连词(1个一类，1个二类)

c 连词

    cc 并列连词

15.     助词(1个一类，15个二类)

u 助词

uzhe 着

ule 了 喽

uguo 过

ude1 的 底

ude2 地

ude3 得

usuo 所

udeng 等 等等 云云

uyy 一样 一般 似的 般

udh 的话

uls 来讲 来说 而言 说来

uzhi 之

ulian 连 （“连小学生都会”）

16.     叹词(1个一类)

e 叹词

17.     语气词(1个一类)

y 语气词(delete yg)

18.     拟声词(1个一类)

o 拟声词

19.     前缀(1个一类)

h 前缀

20.     后缀(1个一类)

k 后缀

21.     字符串(1个一类，2个二类)

x 字符串

    xe  Email字符串

xs 微博会话分隔符

xm 表情符合

xu 网址URL

22.     标点符号(1个一类，16个二类)

w 标点符号

wkz 左括号，全角：（ 〔  ［  ｛  《 【  〖 〈   半角：( [ { <

wky 右括号，全角：） 〕  ］ ｝ 》  】 〗 〉 半角： ) ] { >

wyz 左引号，全角：“ ‘ 『

wyy 右引号，全角：” ’ 』

wj 句号，全角：。

ww 问号，全角：？ 半角：?

wt 叹号，全角：！ 半角：!

wd 逗号，全角：， 半角：,

wf 分号，全角：； 半角： ;

wn 顿号，全角：、

wm 冒号，全角：： 半角： :

ws 省略号，全角：……  …

wp 破折号，全角：――   －－   ――－   半角：---  ----

wb 百分号千分号，全角：％ ‰   半角：%

wh 单位符号，全角：￥ ＄ ￡  °  ℃  半角：$
